# Rapid protein sequence evolution via compensatory frameshift is widespread in RNA virus genomes

**DOI:** 10.1186/s12859-021-04182-9

**Published:** 2021-05-17

**Authors:** Dongbin Park, Yoonsoo Hahn

**Affiliations:** Department of Life Science, Chung-Ang University, Seoul, 06794 South Korea

**Keywords:** RNA virus, Compensatory frameshift, Viral genome, Protein evolution

## Abstract

**Background:**

RNA viruses possess remarkable evolutionary versatility driven by the high mutability of their genomes. Frameshifting nucleotide insertions or deletions (indels), which cause the premature termination of proteins, are frequently observed in the coding sequences of various viral genomes. When a secondary indel occurs near the primary indel site, the open reading frame can be restored to produce functional proteins, a phenomenon known as the compensatory frameshift.

**Results:**

In this study, we systematically analyzed publicly available viral genome sequences and identified compensatory frameshift events in hundreds of viral protein-coding sequences. Compensatory frameshift events resulted in large-scale amino acid differences between the compensatory frameshift form and the wild type even though their nucleotide sequences were almost identical. Phylogenetic analyses revealed that the evolutionary distance between proteins with and without a compensatory frameshift were significantly overestimated because amino acid mismatches caused by compensatory frameshifts were counted as substitutions. Further, this could cause compensatory frameshift forms to branch in different locations in the protein and nucleotide trees, which may obscure the correct interpretation of phylogenetic relationships between variant viruses.

**Conclusions:**

Our results imply that the compensatory frameshift is one of the mechanisms driving the rapid protein evolution of RNA viruses and potentially assisting their host-range expansion and adaptation.

**Supplementary Information:**

The online version contains supplementary material available at 10.1186/s12859-021-04182-9.

## Background

RNA viruses are one of the most common virus types infecting a wide range of hosts including animals, fungi, and plants [1–4]. Their remarkable success is due to their exceptional evolvability. The evolution of RNA viruses is driven by several molecular mechanisms, including the high rate of sequence substitutions [5], recombination of genomic RNAs [6], and reassortment of genome segments [7].

RNA virus genomes are replicated by an RNA-dependent RNA polymerase (RdRp), which has a low to no capacity for efficient proofreading activity [8]. Therefore, RNA viruses exhibit higher mutation rates than other replication units [9]. Their mutation rates were calculated to be within the range of 1 × 10^–6^ to 1 × 10^–4^ substitutions per nucleotide site per cell infection [10–12]. The ineffective proofreading activity of RdRp also results in a high frequency of insertions and deletions (indels) in protein-coding open reading frames (ORFs) [13]. Indels can also occur as a result of various flaws in viral RNA synthesis, including the secondary structure of templates, misalignment of growing strands, and template switching [14–16]. A previous study on the tobacco mosaic virus revealed that a considerable proportion of indel mutations occurred spontaneously. Specifically, of 35 total mutations, 24 were insertions or deletions of a single or multiple nucleotide(s) [17]. An indel mutation can cause a frameshift, which may lead to a disrupted ORF thereby producing truncated proteins [18, 19].

A defective genome containing indel mutations could be maintained in a quasispecies population, which is a mixture of a substantial number of variant genomes [20, 21]. RNA viruses usually infect a host as a quasispecies rather than as a clonal population with the same genome sequence [22, 23]. Intrinsic features of RNA viruses, such as error-prone replication and the ability to produce a substantial number of progenies in a short time period, allow for the maintenance of quasispecies after selection and bottleneck events. In a quasispecies population, continuous complementation and/or interference occur within individual variant genomes, having a positive or negative effect on viral fitness [19, 24–26]. Of note, defective viral genomes producing truncated proteins can propagate in a host with the help of functional genomes (helpers) [27]. While a defective genome is being replicated by helpers, a secondary indel may occur and revert the defective genome to a functional one. A pair of indels may restore the downstream ORF, resulting in a short segment using an altered reading frame. This phenomenon is known as a “compensatory frameshift” [28, 29].

With the advancement of sequencing technology, the number and diversity of identified viral genome sequences have rapidly increased [30]. High-throughput RNA sequencing data obtained from infected samples are a powerful and cost-effective resource for the identification and characterization of novel RNA virus genome sequences [31–33]. Further, the analysis of such large viral genome sequence resources may yield novel insights into virus evolution [34, 35].

As RNA viruses exhibit a rapid mutation rate and a non-negligible frequency of spontaneous indels, compensatory frameshifts may occur at a high frequency. In this study, we systematically analyzed known RNA virus genome sequences to identify compensatory frameshifts. The biological and experimental implications of compensatory frameshift events were also discussed.

## Results

### Identification of compensatory frameshift cases in RNA virus genomes

**Fig. 1:**
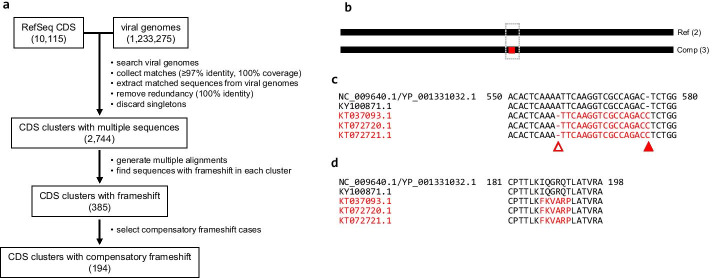
The schematic and an example of the identification of compensatory frameshift cases in RNA virus genomes. **a** A procedure was developed to identify compensatory frameshift cases in the RNA virus genome. By analyzing 10,115 RefSeq CDS sequences and 1,233,275 viral genome sequences, a total of 194 CDS clusters were identified as having at least one compensatory frameshift form. **b** An example compensatory frameshift case found in the PorPV matrix protein is presented. Five PorPV matrix protein CDS sequences were classified into the reference form (“Ref”; two sequences) and a compensatory frameshift form (“Comp”; three sequences). A box indicates the region where the compensatory frameshift was identified. The red line indicates the frameshifted segment using an alternative reading frame in the compensatory frameshift form compared to the reference form. **c** A part of the multiple alignment of PorPV matrix protein CDS sequences is shown. The 1-nt deletion (open triangle) and 1-nt insertion (filled triangle) in the compensatory frameshift form sequences (accession numbers in red) are marked. The frameshifted segments are in red. **d** A part of multiple alignment of PorPV matrix protein sequences is shown. The 6-aa frameshifted segment is in red

We developed a procedure for the identification of compensatory frameshift cases in RNA virus genomes (Fig. 1a). A total of 10,115 reference protein-coding sequences (CDSs) of RNA viruses were collected from the National Center for Biological Information (NCBI) RefSeq database: 1759 from double-stranded RNA (dsRNA) viruses, 5385 from positive-sense (+) single-stranded RNA (ssRNA) viruses, 2885 from negative-sense (−) ssRNA viruses, and 86 from unclassified RNA viruses (Table 1). For each reference CDS sequence, a MEGABLAST search of non-RefSeq viral genomes was performed to collect variant CDS sequences with highly stringent conditions (97% minimum identity and 100% coverage). Subsequently, the reference CDS and matched variant CDS sequences extracted from viral genome sequences were combined into a CDS cluster. After removal of identical sequences, CDS clusters with two or more sequences, including the reference sequence, were retained. There were 2744 CDS clusters with multiple sequences (Additional file 1: Table S1).

**Table 1:** Compensatory frameshift cases identified in viral proteins

Classification	RefSeq genomes	CDSs	CDS clusters^a^	CDS clusters with a frameshift	CDS clusters with a compensatory frameshift
dsRNA	1517	1759	345	45 (13.04%^b^)	25 (7.25%)
(+) ssRNA	2757	5385	1345	209 (15.54%)	110 (8.18%)
(−) ssRNA	1344	2885	1047	131 (12.51%)	59 (5.64%)
Unclassified	69	86	7	0	0
Total	5687	10,115	2744	385 (14.03%)	194 (7.07%)

^a^Number of CDS clusters containing a reference and variant sequences

^b^Percentages are relative to the number of CDS clusters

For each CDS cluster, sequences were multiply aligned. Thereafter, variant CDS sequences were compared to the reference CDS sequence and classified as either a reference or frameshift form (see Methods). Of 2744 CDS clusters, 385 clusters had at least one frameshift form. These frameshift forms were further examined to determine if the frameshift event resulted in protein truncation or not. When an indel event resulted in protein truncation, the variant CDS sequence was classified as a truncating frameshift form. On the other hand, if multiple indel events restored the original ORF without a stop codon, the variant CDS was classified as a compensatory frameshift form.

As a result, 194 CDS clusters were identified to contain at least one compensatory frameshift form (Table 1 and Additional file 1: Table S1). In some clusters, two or more different compensatory forms were observed. Of note, two or more independent frameshifted segments were present in some compensatory frameshift form sequences. There was a total of 301 different compensatory frameshift cases, where boundaries of the frameshifted segment were unique (Additional file 2: Table S2). Lengths of frameshifted segments using an alternative reading frame ranged from 5 to 87 amino acids (aa) (Additional file 3: Fig. S1). The most frequent length was 8 aa, which was observed in 30 cases. Most cases (293 cases; 97.34%) had a frameshifted segment of 40 aa or shorter.

A frameshift mutation in a sequence could be due to experimental artifacts rather than a natural product. In this study, it was postulated that a compensatory frameshift case would be genuine when the same case was observed in multiple different sequence records. Among the 301 compensatory frameshift cases, 80 cases were supported by at least two different sequences, suggesting that they were naturally originated. However, it cannot be ruled out that at least some of the rest 221 cases, which were supported by only one sequence, were resulted from an experimental artifact.

Compensatory frameshift cases were observed in all three types of RNA viruses: 25 clusters from dsRNA viruses, 110 from (+) ssRNA viruses, and 59 from (−) ssRNA viruses. Compared to the initial number of CDS clusters, these accounted for 7.25%, 8.18%, and 5.64% for dsRNA, (+) ssRNA, and (−) ssRNA viruses, respectively.

As an example, the compensatory frameshift case identified in the matrix protein of porcine rubulavirus (PorPV) is presented in Fig. 1b. PorPV is a member of the genus *Rubulavirus* (family *Paramyxoviridae*) and was responsible for the porcine “blue eye disease” outbreak in Mexico [36]. The reference CDS sequence of the PorPV matrix protein was 1110 nt including the stop codon (accession number NC_009640.1) and encoded a 369-aa protein (accession number YP_001331032.1).

There were five CDS sequences in the PorPV matrix protein CDS cluster. Sequence comparison revealed that three variant sequences had a 1-nt deletion at position 559 of the reference CDS sequence (hereafter, position numbers are based on the reference CDS sequence), which caused a reading frame change (Fig. 1c). However, a 1-nt insertion at position 576 restored the reading frame. This pair of indels resulted in the translation of a small segment from an altered reading frame, leading to the 6-aa sequence change in the protein sequence (Fig. 1d). The 6-aa sequence, 187-IQGRQT-192, of the reference form was replaced by the altered sequence, FKVARP, in the compensatory frameshift form. Except for this segment, protein sequences were almost identical between the two forms as their nucleotide sequences were highly conserved.

Nucleotide sequence identity between the reference CDS sequence (NC_009640.1) and a compensatory frameshift form (KT037093.1) was 99.82% (1109 identical residues over 1111-nt alignment), while protein sequence identity was 98.37% (363 identical residues over 369-aa alignment). Generally, the degree of protein sequence identity is higher than that of nucleotide sequence identity as synonymous substitution rates in the nucleotide sequence are generally higher than non-synonymous substitution rates. However, this was not the case here, probably because of the 6-aa difference caused by the pair of indels. Therefore, the compensatory frameshift event led to a large-scale change in the protein sequence.

### Compensatory frameshift may cause a higher estimation of evolutionary distance in the protein tree

Compensatory frameshifts cause large-scale amino acid changes through multiple indels, even though nucleotide sequences are almost identical. Therefore, phylogenetic analyses based on nucleotide and protein sequences may show discordant results. It is expected that the evolutionary distance calculated from a protein tree would be greater than that based on a nucleotide tree.

**Fig. 2:**
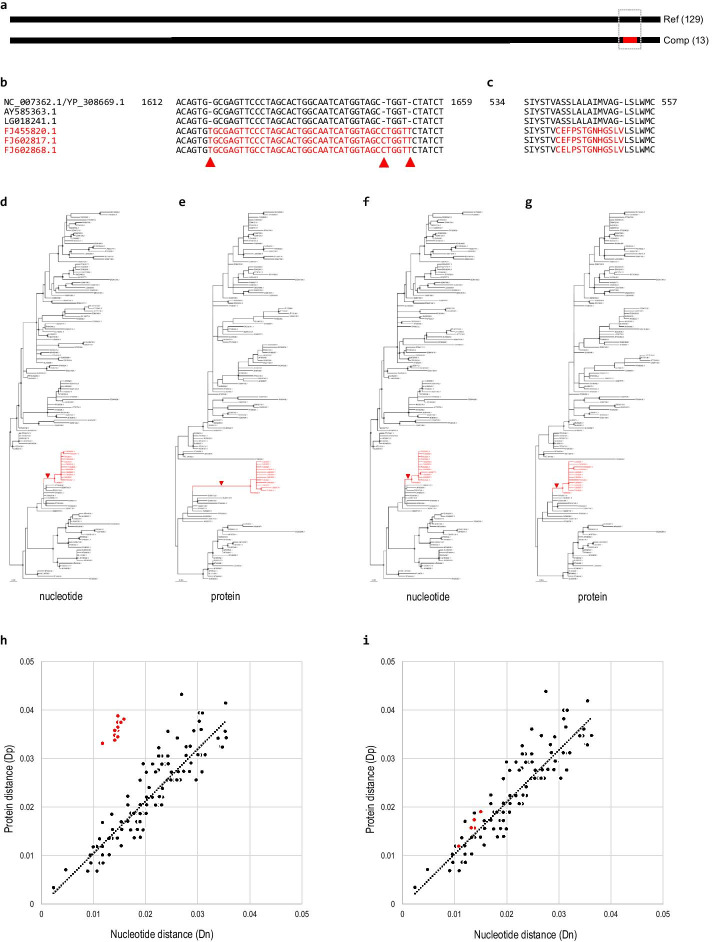
Higher estimation of protein evolutionary distance caused by the compensatory frameshift. **a** A compensatory frameshift case was identified near the C-terminus of the IAV HA protein. The frameshifted segment is marked in red. There were 129 reference and 13 compensatory frameshift form sequences. **b** A part of multiple alignment of representative IAV HA CDS sequences is shown. Three 1-nt insertion positions in the compensatory frameshift form are marked by filled triangles. The frameshifted segment is in red. The alignment of all 142 sequences is presented in Additional file 3: Fig. S2. **c** Comparison of protein sequences revealed that the 12-aa sequence in the reference form was replaced with a 13-aa sequence (in red) in the compensatory frameshift form. **d**, **e** Phylogenetic trees inferred from multiply aligned IAV HA nucleotide (**d**) and protein (**e**) sequences are presented. In both trees, all compensatory frameshift form sequences (in red) were grouped into a single clade, suggesting that the indel event occurred once in the ancestral branch (red arrowhead) of all the compensatory frameshift form sequences. Note that the relative length of the ancestral branch of the compensatory frameshift form sequences is longer in the protein tree than in the nucleotide tree. Branches with bootstrap support values of ≥ 95% are marked by black or red circles on the nodes. **f**, **g** Phylogenetic trees inferred from multiply aligned IAV HA nucleotide (**f**) and protein (**g**) sequences are presented. Note that the length of the ancestral branch (red arrowhead) of the compensatory frameshift form sequences is significantly shorter in the protein tree (**g**) compared to the original tree (**e**). High resolution images of phylogenetic trees (**d**–**g**) are presented in Additional file 3: Fig. S3. **h** Protein distance values (Dp; vertical axis) between the reference sequence and all other sequences deduced from the protein tree were plotted against corresponding nucleotide distance values (Dn; horizontal axis) deduced from the nucleotide tree. Black and red dots indicate the reference and compensatory frameshift form sequences, respectively. The dotted linear regression line was calculated only from data of the reference form sequences. Note the higher Dp values of the compensatory frameshift form sequences compared to those of reference form sequences with similar Dn values. **i** When the frameshifted segments were removed, Dp values of the compensatory frameshift form sequences were similar to those of reference form sequences

To test this hypothesis, we analyzed the hemagglutinin (HA) CDS cluster of the influenza A virus (IAV), which had a case of compensatory frameshift (Fig. 2). IAV is a zoonotic virus that belongs to the genus *Influenzavirus A* (family *Orthomyxoviridae*) [37]. It is the primary cause of seasonal flu epidemics and occasional pandemics worldwide [38]. The reference CDS sequence obtained from the RefSeq genome sequence (accession number NC_007362.1) is 1707 nt and encodes a 568-aa protein (YP_308669.1). The HA CDS cluster contained 145 sequences obtained through a similarity search of viral genome sequences using the reference sequence as a query. Among them, 129 and 13 sequences were identified to be the reference and compensatory frameshift forms, respectively (Fig. 2a). The remaining three sequences were of different types of frameshift forms.

In the compensatory frameshift form sequences, three separate 1-nt insertions at positions 1618, 1650, and 1654 were identified when upon comparison with the reference form (Fig. 2b). When aligned nucleotide sequences were translated, these three insertions resulted in a large-scale aa sequence difference between the reference and compensatory frameshift forms (Fig. 2c). The 12-aa sequence of the reference form was replaced with a 13-aa sequence in the compensatory frameshift form. The compensatory frameshift form had one more amino acid in this segment due to the three 1-nt insertions.

Nucleotide and protein sequences of the IAV HA CDS were used for phylogenetic analyses (Fig. 2d, e). The phylogenetic tree based on nucleotide sequences revealed that the 13 compensatory frameshift form sequences formed a single clade. This implied that the compensatory frameshift event occurred in the ancestral branch (red arrowhead in Fig. 2d) of these 13 viruses. The tree based on protein sequences showed a similar branching pattern to that of the nucleotide tree. However, upon visual inspection, the relative length of the ancestral branch (red arrowhead in Fig. 2e) of the compensatory frameshift form in the protein tree was much longer than that of the corresponding branch of the nucleotide tree.

To test if the increase of the length of the ancestral branch of the compensatory frameshift forms was due to the frameshifted segment, the aligned region corresponding to the frameshifted segment was removed from the nucleotide and protein sequence alignments. Phylogenetic trees were then inferred from these edited alignments. The resulting nucleotide (Fig. 2f) and protein (Fig. 2g) trees were virtually identical to the respective original trees (Fig. 2d, e, respectively). However, the length of the ancestral branch (red arrowhead in Fig. 2g) of the compensatory frameshift forms in the protein tree was significantly reduced compared to that of the original tree. This result strongly suggested that the frameshifted segment caused the overestimation of protein sequence evolution of compensatory frameshift forms in the original protein tree.

To investigate whether protein distances (Dp) between the reference and the compensatory frameshift form sequence were greater than the nucleotide distances (Dn), evolutionary distances in the protein and nucleotide trees were compared. Tip-to-tip distances from the reference CDS sequence (NC_007362.1) to 141 variant sequences (128 reference and 13 compensatory frameshift form sequences) in the inferred protein and nucleotide trees were calculated. When Dp values were plotted against Dn values calculated from the original trees (Fig. 2d, e), a clear distinction between reference and compensatory frameshift form sequences was observed (Fig. 2h). It was expected that Dp and Dn values may be correlated because the protein sequence change is generally proportional to nucleotide substitutions in the CDS sequence. As expected, Dp and Dn values of reference form sequences showed a positive correlation. The linear regression line (dotted line in Fig. 2h) calculated using distances of reference forms indicated a clear correlation. However, compensatory frameshift forms (red dots in Fig. 2h) appeared above the regression line. This suggested that compensatory frameshift forms had higher Dp values than reference forms with similar Dn values. When Dp and Dn values were re-calculated from trees inferred from sequences without the frameshifted segments (Fig. 2f, g), Dp and Dn values for both reference and compensatory frameshift forms showed the same pattern (Fig. 2i), implying that the increase of Dp values in compensatory frameshift forms was due to the frameshifted segment.

To determine if the same phenomenon could be observed in other clusters, we inferred phylogenetic trees and calculated Dp and Dn values from 104 CDS clusters with at least two reference and one compensatory frameshift form sequences (Additional file 4: Data S1). Dp values of the compensatory frameshift forms were higher than those of reference forms with similar Dn values in many clusters.

These results clearly indicated that a compensatory frameshift led to a higher estimation of protein sequence substitutions between viruses with and without the compensatory frameshift. Phylogenetic tree inference programs may treat amino acid sequence changes caused by the compensatory frameshift as accelerated substitutions, resulting in a higher estimation of the evolutionary distance between the two different forms. Therefore, compensatory frameshifts can cause an overestimation of the amino acid substitution rate between sequences with and without compensatory frameshift changes.

### Compensatory frameshift causes discrepancy between nucleotide and protein trees

**Fig. 3:**
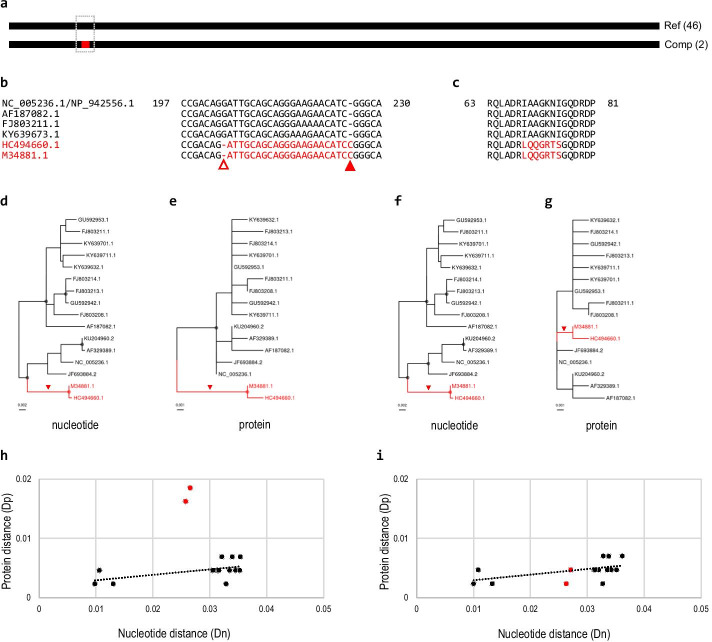
Different branching pattern in the nucleotide and protein trees caused by a compensatory frameshift. **a** A compensatory frameshift was identified in the SEOV nucleocapsid protein. There were 46 reference and two compensatory frameshift form sequences. **b** A part of the multiple alignment of representative SEOV nucleocapsid CDS sequences is shown. The compensatory frameshift form sequences (in red) have a 1-nt deletion (open triangle) and a 1-nt insertion (filled triangle). The alignment of all 48 nucleotide sequences is presented in Additional file 3: Fig. S4. **c** A part of protein sequence alignment shows the 7-aa frameshifted sequences (in red). **d**, **e** Phylogenetic trees inferred from full-length nucleotide (**d**) and protein (**e**) sequences are presented. The ancestral branch of the compensatory frameshift form sequences (in red) is indicated by a red arrowhead. **f**, **g** Phylogenetic trees inferred from nucleotide (**f**) and protein (**g**) sequences without the frameshift segment revealed that the compensatory frameshift form clade (in red) was placed among reference form sequences in both trees. High resolution images of phylogenetic trees (**d**–**g**) are presented in Additional file 3: Fig. S5. **h** Protein distance (Dp) values between the reference sequence and all the other sequences were plotted against corresponding nucleotide distance (Dn) values. Dp values of the compensatory frameshift form sequences (red dots) were higher compared to those of reference form sequences (black dots) albeit their similar Dn values. **i** When the frameshifted segment was removed, Dp values of the compensatory frameshift form sequences were similar to those of the reference form sequences

Compensatory frameshifts result in overestimation of amino acid sequence substitutions compared to nucleotide sequence substitutions. In some circumstances, this overestimation may cause a different branching pattern between the nucleotide and protein trees, which may obscure an accurate phylogenetic relationship among virus variants. An example case was observed in the Seoul orthohantavirus (SEOV) nucleocapsid CDS sequences (Fig. 3).

SEOV is a member of the genus *Orthohantavirus* of the family *Hantaviridae* [39], is carried by black (*Rattus rattus*) or brown rats (*R. norvegicus*), and causes hemorrhagic fever with renal syndrome in humans [40]. The reference SEOV nucleocapsid CDS sequence (1290 nt) was obtained from the RefSeq SEOV S genome segment (NC_005236.1), encoding a 429-aa protein (NP_942556.1). The SEOV nucleocapsid CDS cluster contained 48 sequences: 46 were the reference forms, whereas two were compensatory frameshift forms (Fig. 3a). Sequence comparison revealed that the compensatory frameshift form sequences had a 1-nt deletion at position 204 and a 1-nt insertion at 226 compared to the reference form (Fig. 3b), resulting in the sequence difference in the 7-aa segment (Fig. 3c).

Phylogenetic trees were constructed from 16 selected nucleotide and protein sequences of the SEOV nucleocapsid CDS sequences (Fig. 3d, e). Many reference form CDSs were translated to the same protein sequence although their nucleotide sequences were different. We selected 14 reference and two compensatory frameshift form sequences with non-redundant protein sequences. The nucleotide and protein trees revealed that the two compensatory frameshift form sequences formed a unique clade, implying that the compensatory frameshift event occurred in the ancestral branch (red arrowhead in Fig. 3d, e). Interestingly, the branching patterns of nucleotide and protein trees differed from one another when the midpoint rooting was applied. In the nucleotide tree, the compensatory frameshift form sequences were placed within the reference form sequences. In contrast, in the protein tree, all reference form sequences formed a single clade, excluding the compensatory frameshift form sequences.

Phylogenetic trees were inferred using the nucleotide and protein sequence alignments after the aligned region corresponding to the frameshifted segment of the compensatory frameshift forms was removed. In the nucleotide tree (Fig. 3f), the compensatory frameshift form sequences were placed within the reference form clade as in the original tree (Fig. 3d). Interestingly, the compensatory frameshift form clade was also placed among reference form sequences in the protein tree (Fig. 3g) and the length of the ancestral branch of the compensatory frameshift forms was shorter compared to the original tree (Fig. 3e). This result implied that the 7-aa sequence change caused by the compensatory frameshift could result in an overestimation of amino acid sequence substitution in the original protein tree and placed the two compensatory frameshift form sequences as an early-branching lineage.

Dp and Dn values were calculated from the reference sequence (NC_005236.1) to variant sequences in the inferred protein and nucleotide trees. When Dp values were plotted against Dn values, Dp values from the compensatory frameshift form sequences were displaced from the linear regression line inferred from data of the reference form sequences (Fig. 3h). The plot revealed that Dp values of the reference form sequences were similar to each other although their Dn values were different, suggesting a notable suppression of non-synonymous nucleotide substitutions. When the frameshifted segment was removed from the alignment and Dp and Dn values were re-calculated, the reference and compensatory frameshift form sequences showed similar Dp values (Fig. 3i).

Therefore, a large-scale amino acid change due to a compensatory frameshift could cause an overestimation of amino acid substitution, which may in turn lead to a different pattern of branching for compensatory frameshift forms in the protein and nucleotide trees. This phenomenon could be prominent especially when the protein is under strong negative selection as even a short frameshifted segment may result in a significant protein sequence change compared to the wild type sequences.

## Discussion

We systematically examined 2744 RNA viral CDS clusters, which were composed of a reference sequence derived from the RefSeq database and its closely related variant sequences (non-RefSeq). There were 194 clusters that had at least one compensatory frameshift form. The number of compensatory frameshift cases with a unique frameshifted segment was 301, among which 80 cases were supported by multiple sequences. The compensatory frameshift forms were predicted to produce full-length proteins with a stretch of amino acid sequences translated from an alternative reading frame compared to the reference form. Resulting proteins encoded by CDSs with a compensatory frameshift generally have several to tens of different amino acids compared to the reference form. We suggest that the compensatory frameshift is one of the mechanisms enabling the rapid protein evolution in RNA viruses and potentially facilitating the expansion of their host range as well as host defense system evasion.

Compensatory frameshifts can arise via several different mechanisms. Two or more independent serial frameshift mutation events over several replication cycles can generate a reverted genome. However, multiple indel events can also occur during a single replication cycle. Replicative recombination between genomes with different frameshift mutations could be another mechanism allowing for the generation of a functional genome with a compensatory frameshift.

Interestingly, truncating frameshift forms, which cannot produce functional proteins due to premature termination, were also identified in similar numbers. There were 196 CDS clusters that had at least one truncating frameshift form resulting from a frameshift indel mutation. These sequences could be derived from defective replication by-products that have lost their infectious capability. It is rather surprising that many sequences with a frameshift mutation have been deposited in the database. These defective genomes could be isolated because they were stably propagating in host cells using proteins supplied by complementing helper viruses, which are part of a large pool of viral species variant forms, also known as a quasispecies population [41–44]. A secondary indel may occur in a defective genome during subsequent replications, resulting in restoration of the original reading frame. Therefore, truncating frameshift forms with a single frameshift indel mutation are considered intermediates of a compensatory frameshift form.

However, a secondary indel mutation in the truncating frameshift form may not guarantee restoration of the original reading frame. A stop codon may be present within the frameshifted segment even though the original reading frame is restored after the downstream indel. Such CDS sequences would produce prematurely terminated proteins. In our dataset, 22 CDS clusters had at least one truncating frameshift form with a stop codon within the frameshifted segment. These could be examples of dead-end products that failed to generate a compensatory frameshift form.

A compensatory frameshift causes large-scale amino acid sequence differences between the ancestral and derived compensatory frameshift forms even though their nucleotide sequences are almost identical. Such large-scale amino acid sequence changes can interfere with subsequent phylogenetic analyses. Multiple unmatched amino acid sequences in the frameshifted segment might be counted as substitutions, which would result in a higher estimation of the evolutionary distance between protein sequences with and without the compensatory frameshift. Hence, the evolutionary distance between protein sequences of the two forms could be estimated as greater than that between nucleotide sequences (see Figs. 2d–i, 3d–i).

A substantial change of amino acid sequences with conserved nucleotide sequences could cause a different pattern of branching in the nucleotide and protein trees when analyzing sequences with and without a compensatory frameshift in a single dataset (see Fig. 3d–g). This implies that different evolutionary histories of virus variants can be inferred depending on which sequences are analyzed. Usually, protein sequences alone are used for the phylogenetic analysis of virus groups. If some of the viral sequences experienced a compensatory frameshift event, protein-based analysis could infer an incorrect phylogenetic relationship of these viruses. We suggest that nucleotide sequences rather than protein sequences should be used to infer a genealogy of viruses with highly similar genome sequences.

Because this study was designed as a proof-of-concept study, we applied a very stringent condition: only CDS sequences with at least 97% nucleotide sequence identity to a RefSeq sequence were collected. This condition was employed to avoid spurious alignments. As the nucleotide sequence identity between the reference and compensatory frameshift forms decreases, mismatches within the frameshifted segment will be preferred than gaps at the boundaries, which may conceal the indel events. When the nucleotide sequence similarity is low, gaps can be inserted to maximize the alignment score even if there are no actual historical indel events. Under highly stringent conditions, every alignment gap in nucleotide sequences is likely due to an indel event. Thus, amino acid sequences derived from the same reading frame of two forms would be almost identical to each other, but those from the frameshifted segment could be drastically different.

It is relatively easy to detect a compensatory frameshift event when highly similar sequences are examined as in this study. However, in practice, viral protein sequences from the same genus or even the same species only share a moderate sequence identity. For example, the species demarcation criteria in the genus *Betaflexiviridae* and in various other genera is that different species have less than 80% amino acid identity between coat protein or RdRp sequences [45]. Proteins from the same viral species may have an amino acid sequence identity as low as 80% and much lower nucleotide sequence identity. In cases of such low identity, it could be difficult to detect a compensatory frameshift event, if any. Therefore, it is highly likely that there are more compensatory frameshift cases in available RNA genome sequences, which cannot be detected by conventional sequence comparisons.

## Conclusions

We identified various cases of compensatory frameshifts in available RNA virus genome sequences. The compensatory frameshift is one of the mechanisms driving the rapid protein sequence evolution of RNA viruses with conserved genome sequences. A compensatory frameshift can interfere with the inference of the evolutionary history of related viruses because amino acid sequence mismatches caused by a compensatory frameshift could be misinterpreted as substitutions. Further comprehensive studies of compensatory frameshifts could expand our knowledge on the mechanisms of protein evolution in RNA viruses.

## Methods

### Reference viral CDS sequence data

RefSeq genome sequences of the realm *Riboviria* (taxonomy ID 2559587) including dsRNA viruses and ssRNA viruses were downloaded from the NCBI RefSeq database (https://www.ncbi.nlm.nih.gov/refseq) on April 1st, 2020. There were 1426 dsRNA, 1894 (+) ssRNA, 1278 (−) ssRNA, and 1089 unclassified RNA viruses. Most of unclassified RNA viruses (1054 viruses) were represented by genome sequences identified from invertebrate transcriptome data [2]. The unclassified viruses were re-classified based on their protein sequence similarity to classified viruses, which was performed by BLASTP searches of unclassified virus proteins against classified proteins with an E-value cut-off of 1e-05. As the result, 1089 unclassified viruses were re-classified into 91 dsRNA, 863 (+) ssRNA, 66 (−) ssRNA, and 69 unclassified RNA viruses.

Annotated CDS sequences were extracted from RefSeq viral genome sequences. Sequences shorter than 600 nt or without a start or stop codon were discarded. Obtained RefSeq sequences were designated as “reference” CDS sequences. Information on the major host associated with each virus was obtained from the Virus-Host DB (https://www.genome.jp/virushostdb) [46]. Virus types based on the Baltimore classification were obtained from the ViralZone DB (https://viralzone.expasy.org) [47].

### Variant viral CDS sequence data

Non-RefSeq genome sequences of the realm *Riboviria* were downloaded from the NCBI nucleotide database (https://www.ncbi.nlm.nih.gov/nucleotide) and were converted to BLAST-searchable sequences. MEGABLAST searches were conducted against these non-RefSeq viral genome sequences using the reference CDS sequences as queries [48]. Sequences exhibiting an identity of 97% or greater and 100% coverage were collected. These selected non-RefSeq CDS sequences were extracted from corresponding genome sequences. In cases where a programmed ribosomal frameshift occurred [49, 50], the skipped nucleotide was removed or the repeated nucleotide was added to generate the complete ORF. These sequences were designated as “variant” CDS sequences.

### CDS clusters

For each reference sequence, matched variant sequences were added to form a “CDS cluster”. After removing redundant sequences at 100% identity, CDS clusters containing two or more sequences were retained for further analyses. To obtain a protein sequence, the CDS sequence was translated based on the genetic code designated in its NCBI record. Codons containing an ambiguous nucleotide were translated to X. Nucleotide and protein sequences in a CDS cluster were multiply aligned using the MAFFT program (version 7.453) with the parameter “--auto” to allow the program to choose an optimum strategy for each alignment [51].

### Identification of compensatory frameshift

Gaps in aligned nucleotide sequences were analyzed to find indels in variant sequences relative to the reference sequence. When a gap was found in a variant sequence, it was interpreted as a deletion relative to the reference sequence. When a gap was identified in the reference sequence, an insertion was considered to be present in the variant sequence.

For each variant sequence, all indel events were examined to see if they had caused a frameshift. When the number of inserted or deleted nucleotides at a position was not divisible by three, the indel would cause a frameshift in the downstream region of the variant sequence relative to the reference sequence. When a variant sequence had the same ORF as the reference sequence without any frameshifted segment, it was classified as a “reference form”. When a variant sequence had a frameshifted segment relative to the reference sequence, it was classified as a “frameshift form”. When a frameshift form had premature termination or used a different stop codon compared to the reference form, it was classified as a “truncating frameshift form”. When a frameshift form restored the original (reference) ORF through a downstream indel event, it was classified as a “compensatory frameshift form”.

### Phylogenetic analysis

Multiple sequence alignments of nucleotide or protein sequences were generated using the MAFFT program (version 7.453) with the parameter “--auto” [51]. Maximum likelihood phylogenetic trees were inferred using the IQ-TREE software (version 1.6.12) with parameters “-s <*alignment*> -bb 1000 -keep-ident” [52]. Branch supports were calculated by using the ultrafast bootstrap approximation (UFBoot) method implemented in the IQ-TREE software [53]. Phylogenetic trees were rooted at the midpoint and visualized using the ggtree package [54]. Pairwise evolutionary distances between sequences in the inferred phylogenetic trees were calculated using the nw_distance program of the Newick Utilities suite (version 1.6.0) with the parameter “-n -m m <*treefile*>” [55].

## Supplementary Information

**Additional file 1: Supplementary Table S1.**

**Additional file 2: Supplementary Table S2.**

**Additional file 3: Supplementary Figures S1–S5.**

**Additional file 4: Supplementary Data S1.**

## Data Availability

Supplementary Information containing Supplementary Figures and Supplementary Tables are provided as Additional files. The datasets (multiple sequence alignments of RefSeq and non-RefSeq viral CDS sequences) analyzed during the current study are available in the Figshare repository (https://doi.org/10.6084/m9.figshare.13238810.v1).

## Abbreviations

CDS: Coding sequence
Dn: Nucleotide distance
Dp: Protein distance
dsRNA: Double-stranded RNA
HA: Hemagglutinin
IAV: Influenza A virus
indel: Insertion and deletion
ORF: Open reading frame
PorPV: Porcine rubulavirus
SEOV: Seoul orthohantavirus
ssRNA: Single-stranded RNA
