# Supplementary material for: Rapid protein sequence evolution via compensatory frameshift is widespread in RNA virus genomes
Source: BMC Bioinformatics. 2021 May 17;22:251. doi: 10.1186/s12859-021-04182-9 (PMC8127213; doi:10.1186/s12859-021-04182-9)

Rapid protein sequence evolution via compensatory frameshift is widespread in RNA virus genomes

Dongbin Park, Yoonsoo Hahn

Department of Life Science, Chung-Ang University, Seoul 06794, Republic of Korea

Additional file 3. Supplementary Figures S1–S5.

Supplementary Figure S1. Distribution of lengths of segments using an alternative reading frame

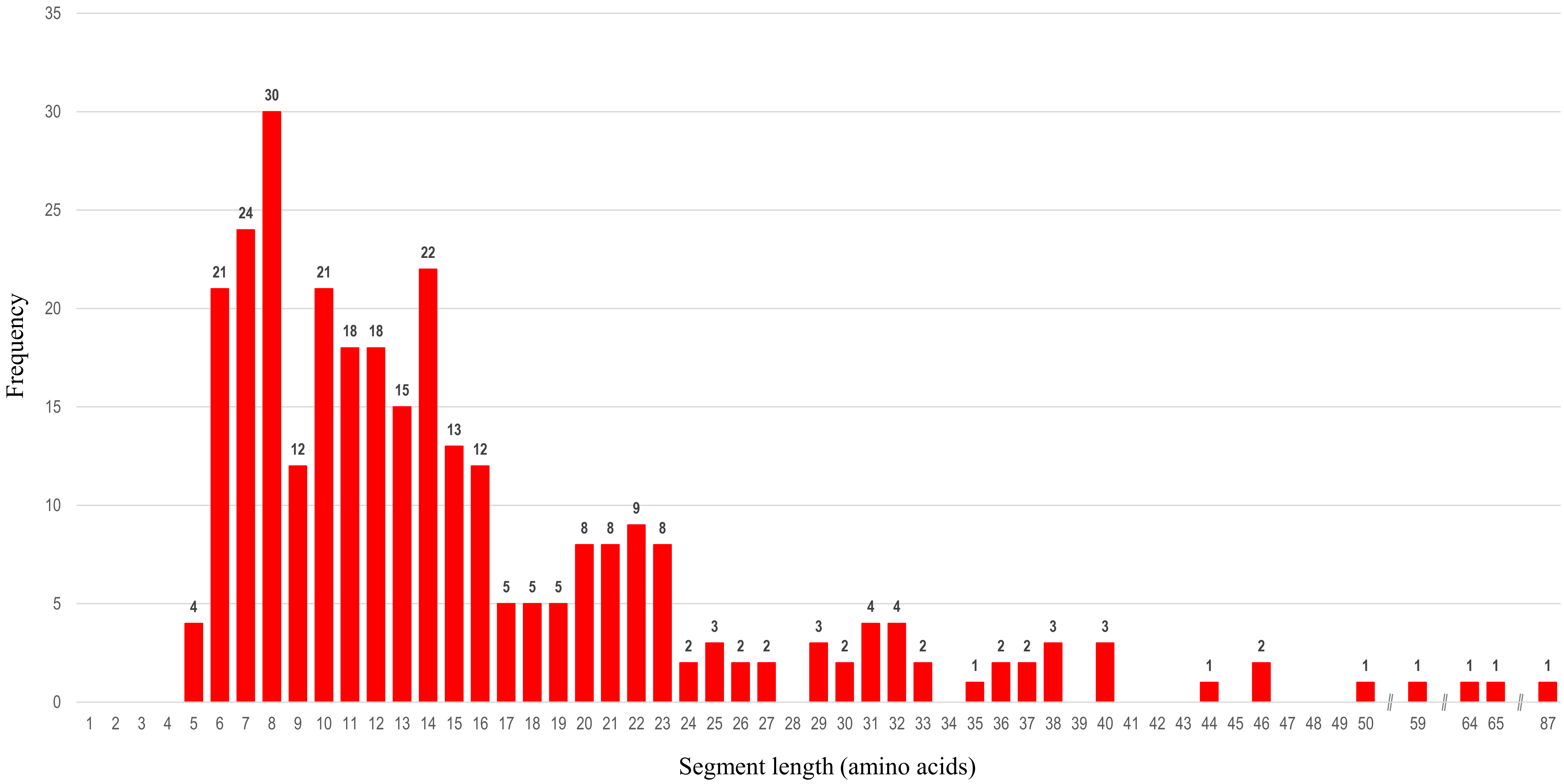

Supplementary Figure S2. Multiple alignment of the IAV HA CDS sequences.

```
NC_007362.1/YP_308669.1   1612 ACAGTG-GCGAGTTCCCTAGCACTGGCAATCATGGTAGC-TGGT-CTATCT 1659
AB557747.1                     ACAGTG-GCGAGTTCCCTAGCACTGGCAATCATGGTAGC-TGGT-CTATCT
AF046080.1                     ACAGTG-GCGAGTTCCCTAGCACTGGCAATCATGGTAGC-TGGT-CTATCT
AF046088.1                     ACAGTG-GCGAGTTCCCTAGCACTGGCAATCATGGTAGC-TGGT-CTATCT
AF046097.1                     ACAGTG-GCGAGTTCCCTAGCACTGGCAATCATGGTAGC-TGGT-CTATCT
AF046099.1                     ACAGTG-GCGAGTTCCCTAGCACTGGCAATCATGGTAGC-TGGT-CTATCT
AF057291.1                     ACAGTG-GCGAGTTCCCTAGCACTGGCAATCATGGTAGC-TGGT-CTATCT
AF082034.1                     ACAGTG-GCGAGTTCCCTAGCACTGGCAATCATGGTAGC-TGGT-CTATCT
AF098541.1                     ACAGTG-GCGAGTTCCCTAGCACTGGCAATCATGGTAGC-TGGT-CTATCT
AF098542.1                     ACAGTG-GCGAGTTCCCTAGCACTGGCAATCATGGTAGC-TGGT-CTATCT
AF098543.1                     ACAGTG-GCGAGTTCCCTAGCACTGGCAATCATGGTAGC-TGGT-CTATCT
AF098545.1                     ACAGTG-GCGAGTTCCCTAGCACTGGCAATCATGGTAGC-TGGT-CTATCT
AF098546.1                     ACAGTG-GCGAGTTCCCTAGCACTGGCAATCATGGTAGC-TGGT-CTATCT
AF148678.1                     ACAGTG-GCGAGTTCCCTAGCACTGGCAATCATGGTAGC-TGGT-CTATCT
AF216713.1                     ACAGTG-GCGAGTTCCCTAGCACTGGCAATCATGGTAGC-TGGT-CTATCT
AF216721.1                     GCAGTG-GCGAGTTCCCTAGCACTGGCAATCATGGTAGC-TGGT-CTATCT
AF216729.1                     ACAGTG-GCGAGTTCCCTAGCACTGGCAATCATGGTAGC-TGGT-CTATCT
AF216737.1                     ACAGTG-GCGAGTTCCCTAGCACTGGCAATCATGGTAGC-TGGT-CTATCT
AF364334.1                     ACAGTG-GCGAGTTCCCTAGCACTGGCAATCATGGTAGC-TGGT-CTATCT
AF468837.1                     ACAGTG-GCGAGTTCCCTAGCACTGGCAATCATGGTAGC-TGGT-CTATCT
AF501234.1                     ACAGTG-GCGAGTTCCCTAGCACTGGCAATCATGGTAGC-TGGT-CTATCT
AY075027.1                     ACAGTG-GCGAGTTCCCTAGCACTGGCAATCATGGTAGC-TGGT-CTATCT
AY075030.1                     ACAGTG-GCGAGTTCCCTAGCACTGGCAATCATGGTAGC-TGGT-CTATCT
AY075033.1                     ACAGTG-GCGAGTTCCCTAGCACTGGCAATCATGGTAGC-TGGT-CTATCT
AY585357.1                     ACAGTG-GCGAGTTCCCTAGCACTGGCAATCATGGTAGC-TGGT-CTATCT
AY585358.1                     ACAGTG-GCGAGTTCCCTAGCACTGGCAATCATGGTAGC-TGGT-CTATCT
AY585359.1                     ACAGTG-GCGAGTTCCCTAGCACTGGCAATCATGGTAGC-TGGT-CTATCT
AY585360.1                     ACAGTG-GCGAGTTCCCTAGCACTGGCAATCATGGTAGC-TGGT-CTATCT
AY585361.1                     ACAGTG-GCGAGTTCCCTAGCACTGGCAATCATGGTAGC-TGGT-CTATCT
AY585362.1                     ACAGTG-GCGAGTTCCCTAGCACTGGCAATCATGGTAGC-TGGT-CTATCT
AY585363.1                     ACAGTG-GCGAGTTCCCTAGCACTGGCAATCATGGTAGC-TGGT-CTATCT
AY585364.1                     ACAGTG-GCGAGTTCCCTAGCACTGGCAATCATGGTAGC-TGGT-CTATCT
AY585365.2                     ACAGTG-GCGAGTTCCCTAGCACTGGCAATCATGGTAGC-TGGT-CTATCT
AY585366.1                     ACAGTG-GCGAGTTCCCTAGCACTGGCAATCATGGTAGC-TGGT-CTATCT
AY585367.1                     ACAGTG-GCGAGTTCCCTAGCACTGGCAATCATGGTAGC-TGGT-CTATCT
AY585368.1                     ACAGTG-GCGAGTTCCCTAGCACTGGCAATCATGGTAGC-TGGT-CTATCT
AY585369.1                     ACAGTG-GCGAGTTCCCTAGCACTGGCAATCATGGTAGC-TGGT-CTATCT
AY585370.1                     ACAGTG-GCGAGTTCCCTAGCACTGGCAATCATGGTAGC-TGGT-CTATCT
AY585371.1                     ACAGTG-GCGAGTTCCCTAGCACTGGCAATCATGGTAGC-TGGT-CTATCT
AY585372.1                     ACAGTG-GCGAGTTCCCTAGCACTGGCAATCATGGTAGC-TGGT-CTATCT
AY585373.1                     ACAGTG-GCGAGTTCCCTAGCACTGGCAATCATGGTAGC-TGGT-CTATCT
AY585374.1                     ACAGTG-GCGAGTTCCCTAGCACTGGCAATCATGGTAGC-TGGT-CTATCT
AY585375.1                     ACAGTG-GCGAGTTCCCTAGCACTGGCAATCATGGTAGC-TGGT-CTATCT
AY585376.1                     ACAGTG-GCGAGTTCCCTAGCACTGGCAATCATGGTAGC-TGGT-CTATCT
AY585377.1                     ACAGTG-GCGAGTTCCCTAGCACTGGCAATCATGGTAGC-TGGT-CTATCT
AY639405.1                     ACAGTG-GCGAGTTCCCTAGCACTGGCAATCATGATAGC-TGGT-CTATCT
AY646424.1                     ACAGTG-GCGAGTTCCCTAGCACTGGCAATCATGGTAGC-TGGT-CTATCT
AY676033.1                     ACAGTG-GCGAGTTCCCTAGCACTGGCAATCATGGTAGC-TGGT-CTATCT
AY684706.1                     ACAGTG-GCGAGTTCCCTAGCACTGGCAATCATGGTAGC-TGGT-CTATCT
AY747609.1                     ACAGTG-GCGAGTTCCCTAGCACTGGCAATCATGGTAGC-TGGT-CTATCT
AY747617.1                     ACAGTG-GCGAGTTCCCTAGCACTGGCAATCATGGTAGC-TGGT-CTATCT
AY854190.1                     ACAGTG-GCGAGTTCCCTAGCACTGGCAATCATGGTAGC-TGGT-CTATCT
AY950230.1                     ACAGTG-GCGAGTTCCCTAGCACTGGCAATCATGGTAGC-TGGT-CTATCT
AY950236.1                     ACAGTG-GCGAGTTCCCTAGCACTGGCAATCATGGTAGC-TGGT-CTATCT
CY028952.1                     ACAGTG-GCGAGTTCCCTAGCACTGGCAATCATGGTAGC-TGGT-CTATCT
CY028957.1                     ACAGTG-GCGAGTTCCCTAGCACTGGCAATCATGGTAGC-TGGT-CTATCT
CY028960.1                     ACAGTG-GCGAGTTCCCTAGCACTGGCAATCATGGTAGC-TGGT-CTATCT
DL141426.1                     ACAGTG-GCGAGTTCCCTAGCACTGGCAATCATGGTAGC-TGGT-CTATCT
DM476494.1                     ACAGTG-GCGAGTTCCCTAGCACTGGCAATCATGGTAGC-TGGT-CTATCT
DQ023145.1                     ACAGTG-GCGAGTTCCCTAGCACTGGCAATCATGGTAGC-TGGT-CTATCT
DQ080022.1                     ACAGTG-GCGAGTTCCCTAGCACTGGCAATCATGGTAGC-TGGT-CTATCT
DQ201829.1                     ACAGTG-GCGAGTTCCCTAGCACTGGCAATCATGGTAGC-TGGT-CTATCT
DQ211925.1                     ACAGTG-GCGAGTTCCCTAGCACTGGCAATCATGGTAGC-TGGT-CTATCT
DQ343151.1                     ACAGTG-GTGAGTTCCCTAGCACTGGCAATCATGGTAGC-TGGT-CTATCT
DQ343152.1                     ACAGTG-GCGAGTTCCCTAGCACTGGCAATCATGGTAGC-TGGT-CTATCT
DQ366314.1                     ACAGTG-GCGAGTTCCCTAGCACTGGCAATCATGGTAGC-TGGT-CTATCT
DQ432037.1                     ACAGTG-GCGAGTTCCCTAGCACTGGCAATCATGGTAGC-TGGT-CTATCT
DQ432045.1                     ACAGTG-GCGAGTTCCCTAGCACTGGCAATCATGGTAGC-TGGT-CTATCT
DQ767725.1                     ACAGTG-GCGAGTTCCCTAGCACTGGCAATCATGGTAGC-TGGT-CTATCT
DQ836043.1                     ACAGTG-GCGAGTTCCCTAGCACTGGCAATCATGGTAGC-TGGT-CTATCT
DQ997094.1                     ACAGTG-GCGAGTTCCCTAGCACTGGCAATCATGGTAGC-TGGT-CTATCT
DQ997102.1                     ACAGTG-GCGAGTTCCCTAGCACTGGCAATCATGGTAGC-TGGT-CTATCT
DQ997122.1                     ACAGTG-GCGAGTTCCCTAGTACTGGCAATCATGGTAGC-TGGT-CTATCT
DQ997133.1                     ACAGTG-GCGAGTTCCCTAGCACTGGCAATCATGGTAGC-TGGT-CTATCT
DQ997147.1                     ACAGTG-GCGAGTTCCCTAGCACTGGCAATCATGGTAGC-TGGT-CTATCT
DQ997163.1                     ACAGTG-GCGAGTTCCCTAGCACTGGCAATCATGGTGGC-TGGT-CTATCT
DQ997182.1                     ACAGTG-GCGAGTTCCCTAGCACTGGCAATCATGGTAGC-TGGT-CTATCT
DQ997218.1                     ACAGTG-GCGAGTTCCCTAGCACTGGCAATCATGGTAGC-TGGT-CTATCT
DQ997219.1                     ACAGTG-GCGAGTTCCCTAGCACTGGCAATCATGGTAGC-TGGT-CTATCT
DQ997253.1                     ACAGTG-GCGAGTTCCCTAGCACTGGCAATCATGGTAGC-TGGT-CTATCT
DQ997262.1                     ACAGTG-GCGAGTTCCCTAGCACTGGCAATCATGGTAGC-TGGT-CTATCT
DQ997268.1                     ACAGTG-GCGAGTTCCCTAGCACTGGCAATCATGGTAGC-TGGT-CTATCT
DQ997283.1                     ACAGTG-GCGAGTTCCCTAGCACTGGCAATCATGGTGGC-TGGT-CTATCT
DQ997308.1                     ACAGTG-GCGAGTTCCCTAGCACTGGCAATCATGGTAGC-TGGT-CTATCT
DQ997318.1                     ACAGTG-GCGAGTTCCCTAGCACTGGCAATCATGGTAGC-TGGT-CTATCT
DQ997325.1                     ACAGTG-GCGAGTTCCCTAGCACTGGCAATCATGGTAGC-TGGT-CTATCT
DQ997355.1                     ACAGTG-GCGAGTTCCCTAGCACTGGCAATCATGGTGGC-TGGT-CTATCT
DQ997377.1                     ACAGTG-GCGAGTTCCCTAGCACTGGCAATCATGGTAGC-TGGT-CTATCT
DQ997392.1                     ACAGTG-GCGAGTTCCCTAGCACTGGCAATCATGGTATC-TGGT-CTATCT
DQ997405.1                     ACAGTG-GCGAGTTCCCTAGCACTGGCAATCATGGTAGC-TGGT-CTATCT
DQ997513.1                     ACAGTG-GCGAGTTCCCTAGCACTGGCAATCATGGTAGC-TGGT-CTATCT
DQ997522.1                     ACAGTG-GCGAGTTCCCTAGCACTGGCAATCATGGTAGC-TGGT-CTATCT
DQ997531.1                     ACAGTG-GCGAGTTCCCTAGCACTGGCAATCATGGTAGC-TGGT-CTATCT
EU195416.1                     ACAGTG-GCGAGTTCCCTAGCACTGGCAATCATGGTGGC-TGGT-CTATCT
EU263345.1                     ACAGTG-GCGAGTTCCCTAGCACTGGCAATCATGGTAGC-TGGT-CTATCT
EU263353.1                     ACAGTG-GCGAGTTCCCTAGCACTGGCAATCATGGTAGC-TGGT-CTATCT
FJ390061.2                     ACAGTG-GCGAGTTCCCTAGCACTGGCAATCATGGTAGC-TGGT-CTATCT
GQ184328.1                     ACAGTG-GCGAGCTCCCTAGCACTGGCAATCATGGTGGC-TGGT-CTATCT
GU052019.1                     ACAGTG-GCGAGTTCCCTAGCACTGGCAATCATGGTAGC-TGGT-CTATCT
GU052027.1                     ACAGTG-GCGAGTTCCCTAGCACTGGCAATCATGGTAGC-TGGT-CTATCT
GU052035.1                     ACAGTG-GCGAGTTCCCTAGCACTGGCAATCATGGTAGC-TGGT-CTATCT
GU052042.1                     ACAGTG-GCGAGTTCCCTAGCACTGGCAATCATGGTAGC-TGGT-CTATCT
GU052050.1                     ACAGTG-GCGAGTTCCCTAGCACTGGCAATCATGGTAGC-TGGT-CTATCT
GU052057.1                     ACAGTG-GCGAGTTCCCTAGCACTGGCAATCATGGTAGC-TGGT-CTATCT
GU052065.1                     ACAGTG-GCGAGTTCCCTAGCACTGGCAATCATGGTAGC-TGGT-CTATCT
GU052073.1                     ACAGTG-GCGAGTTCCCTAGCACTGGCAATCATGGTAGC-TGGT-CTATCT
GU052089.1                     ACAGTG-GCGAGTTCCCTAGCACTGGCAATCATGGTAGC-TGGT-CTATCT
GU052097.1                     ACAGTG-GCGAGTTCCCTAGCACTGGCAATCATGGTAGC-TGGT-CTATCT
GU052105.1                     ACAGTG-GCGAGTTCCCTAGCACTGGCAATCATGGTAGC-TGGT-CTATCT
GU052113.1                     ACAGTG-GCGAGTTCCCTAGCACTGGCAATCATGGTAGC-TGGT-CTATCT
GU052127.1                     ACAGTG-GCGAGTTCCCTAGCACTGGCAATCATGGTAGC-TGGT-CTATCT
GU052135.1                     ACAGTG-GCGAGTTCCCTAGCACTGGCAATCATGGTAGC-TGGT-CTATCT
GU052142.1                     ACAGTG-GCGAGTTCCCTAGCACTGGCAATCATGGTAGC-TGGT-CTATCT
GU052465.1                     ACAGTG-GCGAGTTCCCTAGCACTGGCAATCATGGTAGC-TGGT-CTATCT
GU052494.1                     ACAGTG-GCGAGTTCCCTAGCACTGGCAATCATGGTAGC-TGGT-CTATCT
GU186672.1                     ACAGTG-GCGAGTTCCCTAGCACTGGCAATCATGGTAGC-TGGT-CTATCT
GU186692.1                     ACAGTG-GCGAGTTCCCTAGCACTGGCAATCATGGTAGC-TGGT-CTATCT
JF732739.1                     ACAGTG-GCGAGTTCCCTAGCACTGGCAATCATGGTAGC-TGGT-CTATCT
JF772148.1                     ACAGTG-GCGAGTTCCCTAGCACTGGCAATCATGGTAGC-TGGT-CTATCT
JF772156.1                     ACAGTG-GCGAGTTCCCTAGCACTGGCAATCATGGTAGC-TGGT-CTATCT
JF772164.1                     ACAGTG-GCGAGTTCCCTAGCACTGGCAATCATGGTAGC-TGGT-CTATCT
JX013485.1                     ACAGTG-GCGAGTTCCCTAGCACTGGCAATCATGGTAGC-TGGT-CTATCT
KC784945.1                     ACAGTG-GCGAGTTCCCTAGCACTGGCAATCATGGTAGC-TGGT-CTATCT
KX364460.1                     ACAGTG-GCGAGTTCCCTAGCACTGGCAATCATGGTAGC-TGGT-CTATCT
KX364468.1                     ACAGTG-GCGAGTTCCCTAGCACTGGCAATCATGGTAGC-TGGT-CTATCT
LG018241.1                     ACAGTG-GCGAGTTCCCTAGCACTGGCAATCATGGTAGC-TGGT-CTATCT
LG018243.1                     ACAGTG-GCGAGTTCCCTAGCACTGGCAATCATGGTAGC-TGGT-CTATCT
LX269712.1                     ACAGTG-GCGAGTTCCCTAGCACTGGCAATCATGGTAGC-TGGT-CTATCT
LZ248488.1                     ACAGTG-GTGAGTTCCCTAGCACTGGCAATCATGGTAGC-TGGT-CTATCT
LZ248490.1                     ACAGTG-GCGAGTTCCCTAGCACTGGCAATCATGGTAGC-TGGT-CTATCT
LZ248492.1                     ACAGTG-GCGAGTTCCCTAGCACTGGCAATCATGGTAGC-TGGT-CTATCT
FJ455820.1                     ACAGTGTGCGAGTTCCCTAGCACTGGCAATCATGGTAGCCTGGTTCTATCT
FJ602816.1                     ACAGTGTGCGAGTTCCCTAGCACTGGCAATCATGGTAGCCTGGTTCTATCT
FJ602817.1                     ACAGTGTGCGAGTTCCCTAGCACTGGCAATCATGGTAGCCTGGTTCTATCT
FJ602819.1                     ACAGTGTGCGAGTTCCCTAGCACTGGCAATCATGGTAGCCTGGTTCTATCT
FJ602820.1                     ACAGTGTGCGAGTTCGCTAGCACTGGCAATCATGGTAGCCTGGTTCTATCT
FJ602822.1                     ACAGTGTGCGAGTTCCCTAGCACTGGCAATCATGGTAGCCTGGTTCTATCT
FJ602823.1                     ACAGTGTGCGAGTTCCCTAGCACTGGCAATCATGGTAGCCTGGTTCTATCT
FJ602824.1                     ACAGTGTGCGAGTTCCCTAGCACTGGCAATCATGGTAGCCTGGTTCTATCT
FJ602825.1                     ACAGTGTGCGAGTTCCCTAGCACTGGCAATCATGGTAGCCTGGTTCTATCT
FJ602826.1                     ACAGTGTGCGAGTTCCCTAGCTCTGGCAATCATGGTAGCCTGGTTCTATCT
FJ602827.1                     ACAGTGTGCGAGTTCCCTAGCACTGGCAATCATGGTAGCCTGGTTCTATCT
FJ602828.1                     ACAGTGTGCGAGTTCCCTAGCACTGGCAATCATGGTAGCCTGGTTCTATCT
FJ602868.1                     ACAGTGTGCGAGTTGCCTAGCACTGGCAATCATGGTAGCCTGGTTCTATCT
                                ***** * *** *  ****  ************ *  * **** ******
```

Supplementary Figure S3. Phylogenetic trees of the IAV HA.

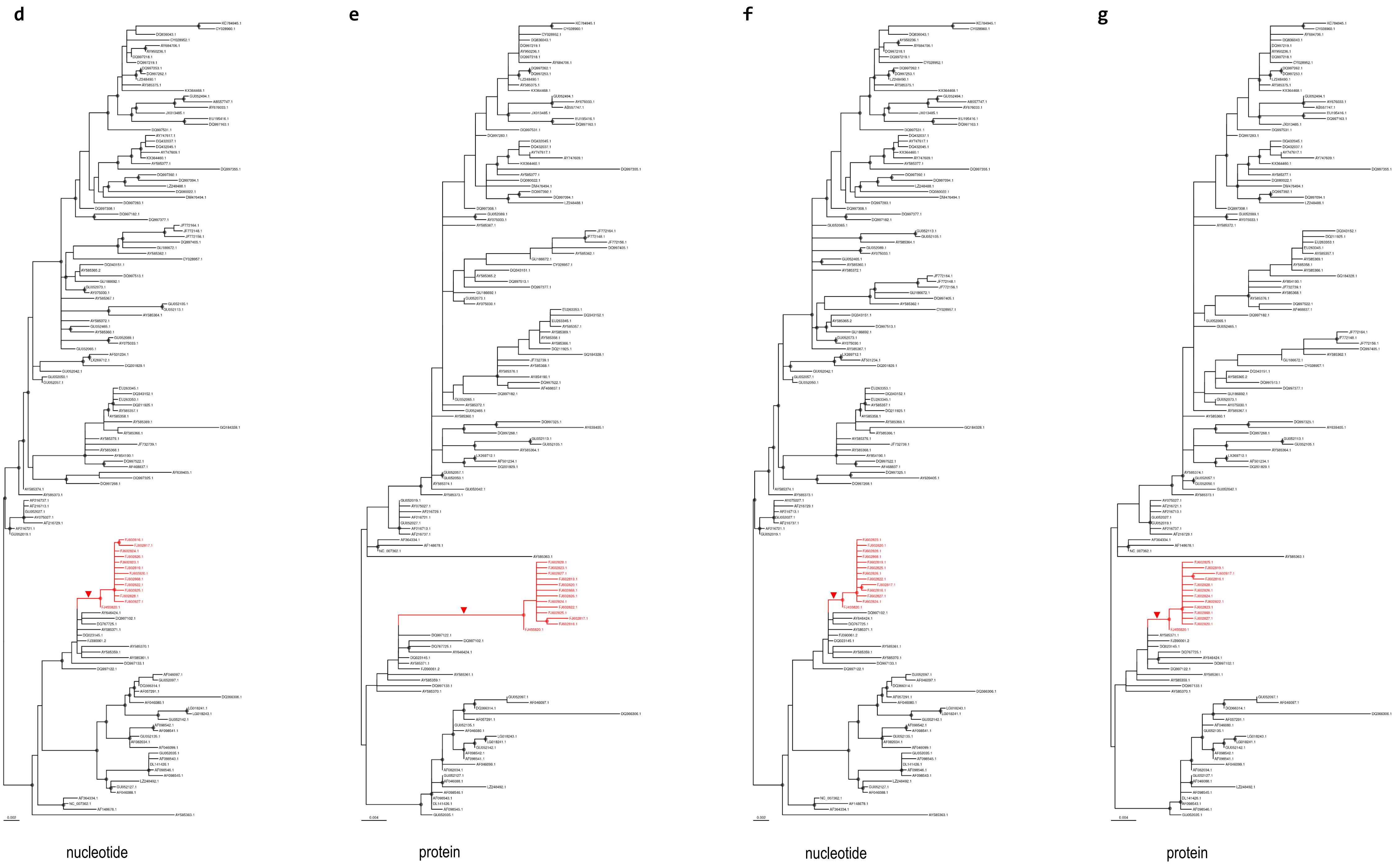

Supplementary Figure S4. Multiple alignment of the SEOV S segment CDS sequences.

```
NC_005236.1/NP_942556.1   197   CCGACAGGATTGCAGCAGGGAAGAACATC-GGGCA    230
AB618116.1                      CCGACAGGATTGCAGCAGGGAAGAATATC-GGGCA
AF187082.1                      CCGACAGGATTGCAGCAGGGAAGAACATC-GGGCA
AF329389.1                      CCGACAGGATTGCAGCAGGGAAGAACATC-GGGCA
AY627049.2                      CCGACAGGATTGCAGCAGGGAAGAACATC-GGGCA
FJ803207.1                      CCGACAGGATTGCAGCAGGGAAGAACATC-GGGCA
FJ803208.1                      CCGACAGGATTGCAGCAGGGAAGAACATC-GGGCA
FJ803211.1                      CCGACAGGATTGCAGCAGGGAAAAACATC-GGGCA
FJ803213.1                      CCGACAGGATTGCAGCAGGGAAGAACATC-GGGCA
FJ803214.1                      CCGACAGGATTGCAGCAGGGAAGAACATC-GGGCA
FJ803216.1                      CCGACAGGATTGCAGCAGGGAAGAACATC-GGGCA
FJ803217.1                      CCGACAGGATTGCAGCAGGGAAGAACATC-GGGCA
GQ279379.1                      CCGACAGGATTGCAGCAGGGAAGAACATC-GGGCA
GQ279381.1                      CCGACAGGATTGCAGCAGGGAAGAACATC-GGGCA
GQ279384.1                      CCGACAGGATTGCAGCAGGGAAGAACATC-GGGCA
GQ279385.1                      CCGACAGGATTGCAGCAGGGAAAAACATC-GGGCA
GQ279392.1                      CCGACAGGATTGCAGCAGGGAAGAACATC-GGGCA
GQ279393.1                      CCGACAGGATTGCAGCAGGGAAGAACATC-GGGCA
GU592941.1                      CCGACAGGATTGCAGCAGGGAAGAACATC-GGGCA
GU592942.1                      CCGACAGGATTGCAGCAGGGAAGAACATC-GGGCA
GU592945.1                      CCGACAGGATTGCAGCAGGGAAAAACATC-GGGCA
GU592950.1                      CCGACAGGATTGCAGCAGGGAAGAACATC-GGGCA
GU592951.1                      CCGACAGGATTGCAGCAGGGAAGAACATC-GGGCA
GU592953.1                      CCGACAGGATTGCAGCAGGGAAAAACATC-GGGCA
JF693884.2                      CCGACAGGATTGCAGCAGGGAAGAACATC-GGGCA
JQ665917.1                      CCGACAGGATTGCAGCAGGGAAGAACATC-GGGCA
JQ665919.1                      CCGACAGGATTGCAGCAGGGAAGAACATC-GGGCA
JQ665926.1                      CCGACAGGATTGCAGCAGGGAAGAACATC-GGGCA
KU204960.2                      CCGACAGGATTGCAGCAGGGAAGAACATC-GGGCA
KY639632.1                      CCGACAGGATTGCAGCAGGGAAAAACATC-GGGCA
KY639666.1                      CCGACAGGATTGCAGCAGGGAAAAACATC-GGGCA
KY639670.1                      CCGACAGGATTGCAGCAGGAAAAAACATC-GGGCA
KY639672.1                      CTGACAGGATTGCAGCAGGGAAAAACATC-GGGCA
KY639673.1                      CCGACAGGATTGCAGCAGGAAAGAACATC-GGGCA
KY639683.1                      CCGACAGGATTGCAGCAGGAAAAAACATC-GGGCA
KY639689.1                      CCGACAGGATTGCAGCAGGAAAAAACATC-GGGCA
KY639701.1                      CCGACAGGATTGCAGCAGGAAAAAACATC-GGGCA
KY639706.1                      CCGACAGGATTGCAGCAGGAAAAAACATC-GGGCA
KY639710.1                      CCGACAGGATTGCAGCAGGGAAAAACATC-GGGCA
KY639711.1                      CCGACAGGATTGCAGCAGGGAAAAACATC-GGGCA
MF149947.1                      CCGACAGGATTGCAGCAGGGAAGAACATC-GGGCA
MF149948.1                      CCGACAGGATTGCAGCAGGGAAGAACATC-GGGCA
MF149949.1                      CCGACAGGATTGCAGCAGGGAAGAACATC-GGGCA
MF149950.1                      CCGACAGGATTGCAGCAGGGAAGAACATC-GGGCA
MF149954.1                      CCGACAGGATTGCAGCAGGAAAGAACATC-GGGCA
MF149956.1                      CCGACAGGATTGCAGCAGGGAAGAACATC-GGGCA
HC494660.1                      CCGACAGA-TTGCAGCAGGGAAGAACATCCGGGCA
M34881.1                        CCGACAGA-TTGCAGCAGGGAAGAACATCCGGGCA
                                *  *****   ********** ** ** *** *****
```

Supplementary Figure S5. Phylogenetic trees of the SEOV S segment.

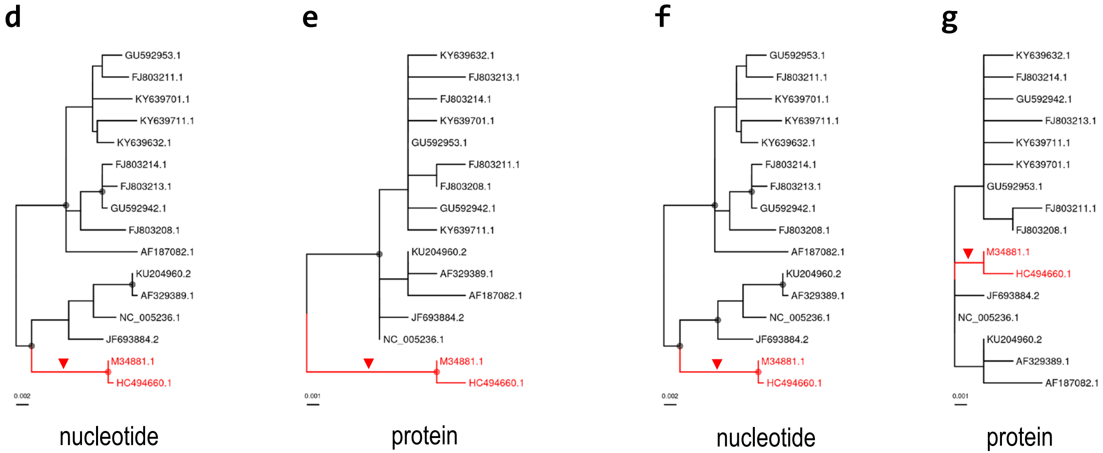

Supplement: Supplementary file 3 — Additional file 3: Supplementary Figures S1–S5. [file 12859_2021_4182_MOESM3_ESM.pdf]
