# Supplementary material for: Rapid protein sequence evolution via compensatory frameshift is widespread in RNA virus genomes
Source: BMC Bioinformatics. 2021 May 17;22:251. doi: 10.1186/s12859-021-04182-9 (PMC8127213; doi:10.1186/s12859-021-04182-9)

Rapid protein sequence evolution via compensatory frameshift is widespread in RNA virus genomes

Dongbin Park, Yoonsoo Hahn

Department of Life Science, Chung-Ang University, Seoul 06794, Republic of Korea

Additional file 4. Supplementary Data S1

2. NC_006213.1/YP_009555240.1

**CDS cluster ID**	**2**
**CDS cluster name**	NC_006213.1/YP_009555240.1
**Total sequences**	121
**Reference forms**	24
**Compensatory frameshift forms**	97
**Virus**	Human coronavirus OC43 (HCoV-OC43)
**Protein**	hemagglutinin-esterase

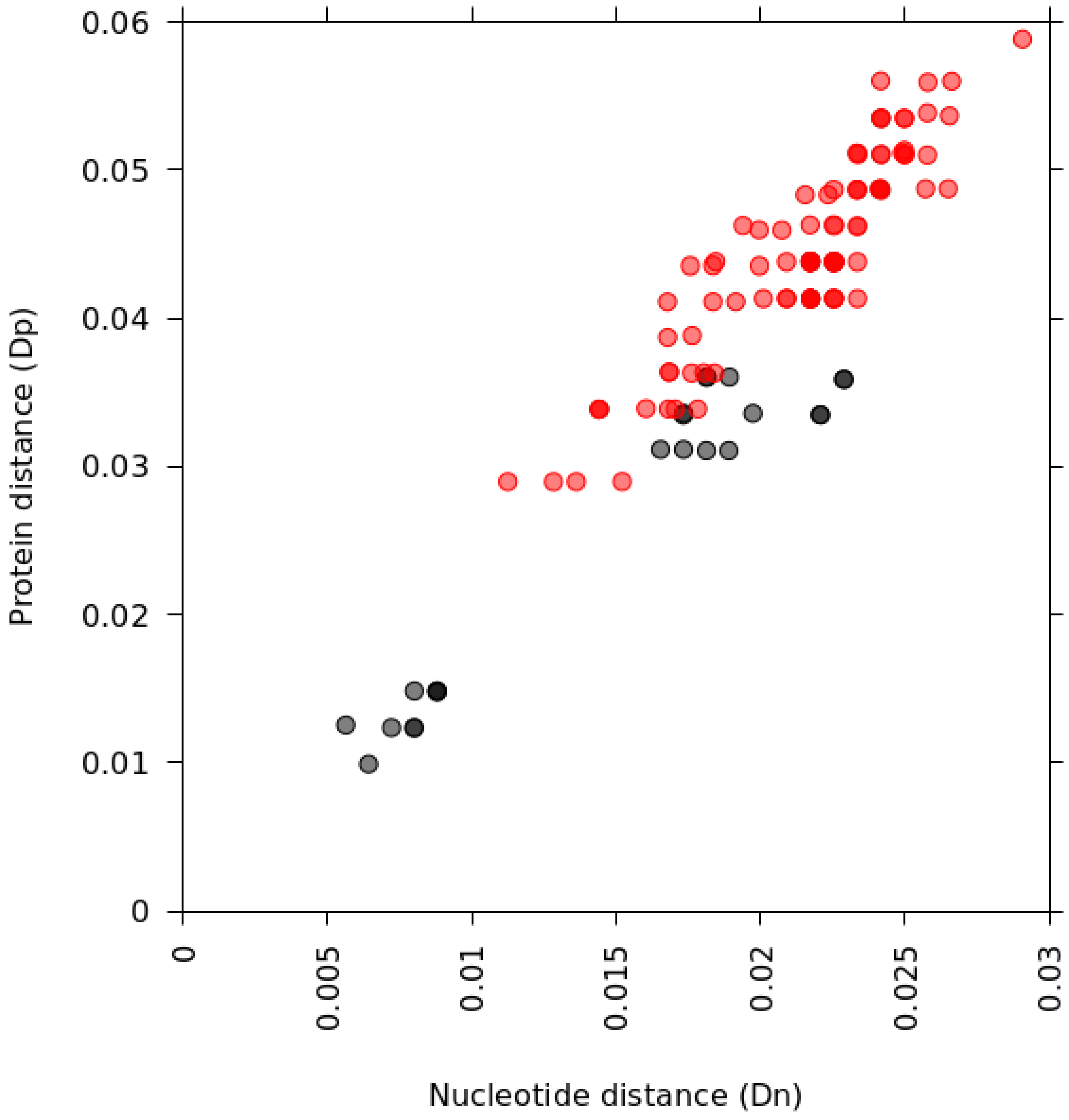

6. NC_003443.1/NP_598406.1

CDS cluster ID	6
CDS cluster name	NC_003443.1/NP_598406.1
Total sequences	33
Reference forms	3
Compensatory frameshift forms	30
Virus	Human rubulavirus 2
Protein	Large protein

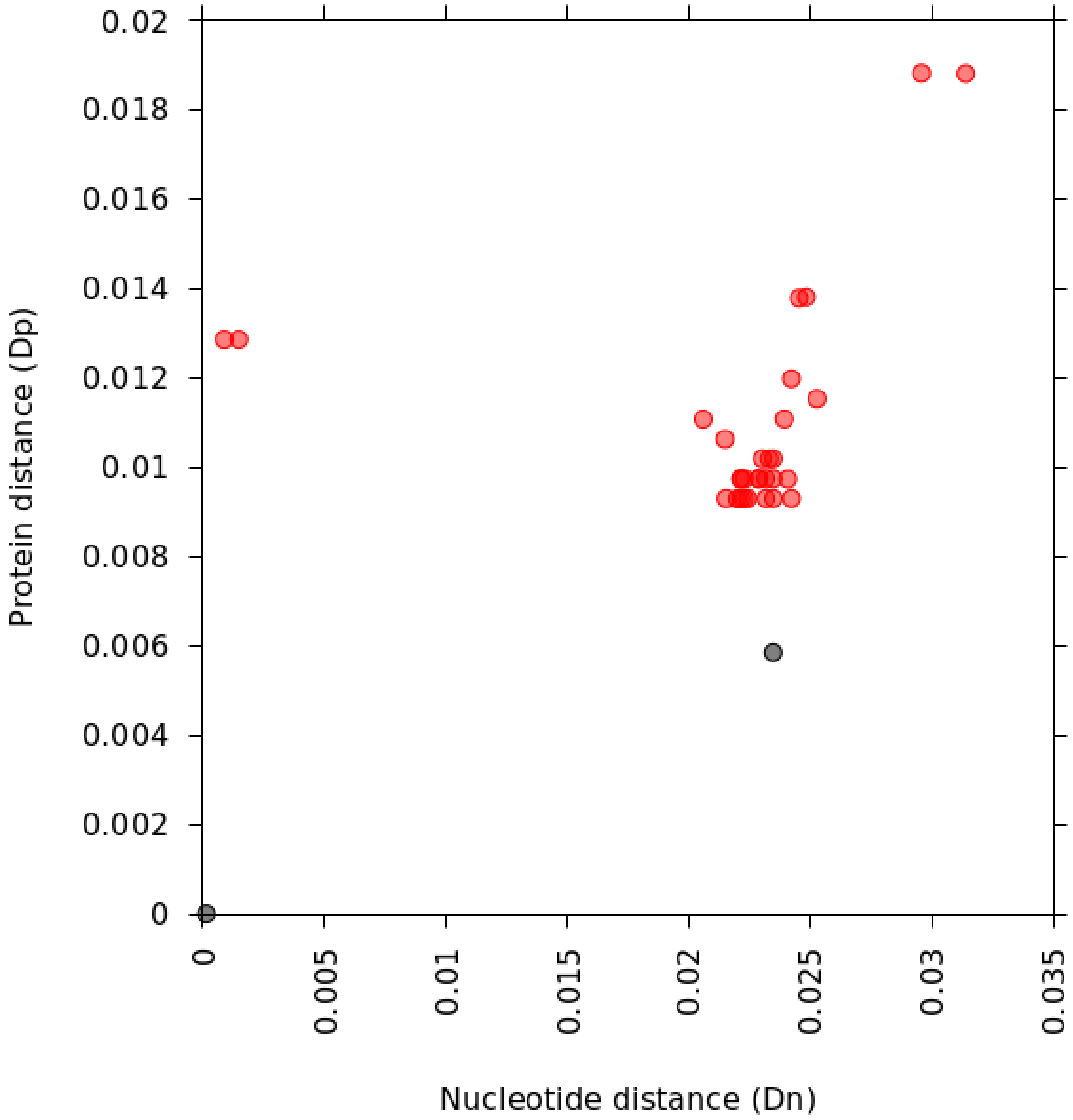

8. NC_003215.1/NP_463457.1

**CDS cluster ID**	**8**
**CDS cluster name**	NC_003215.1/NP_463457.1
**Total sequences**	22
**Reference forms**	6
**Compensatory frameshift forms**	16
**Virus**	Semliki Forest virus
**Protein**	Nonstructural polyprotein

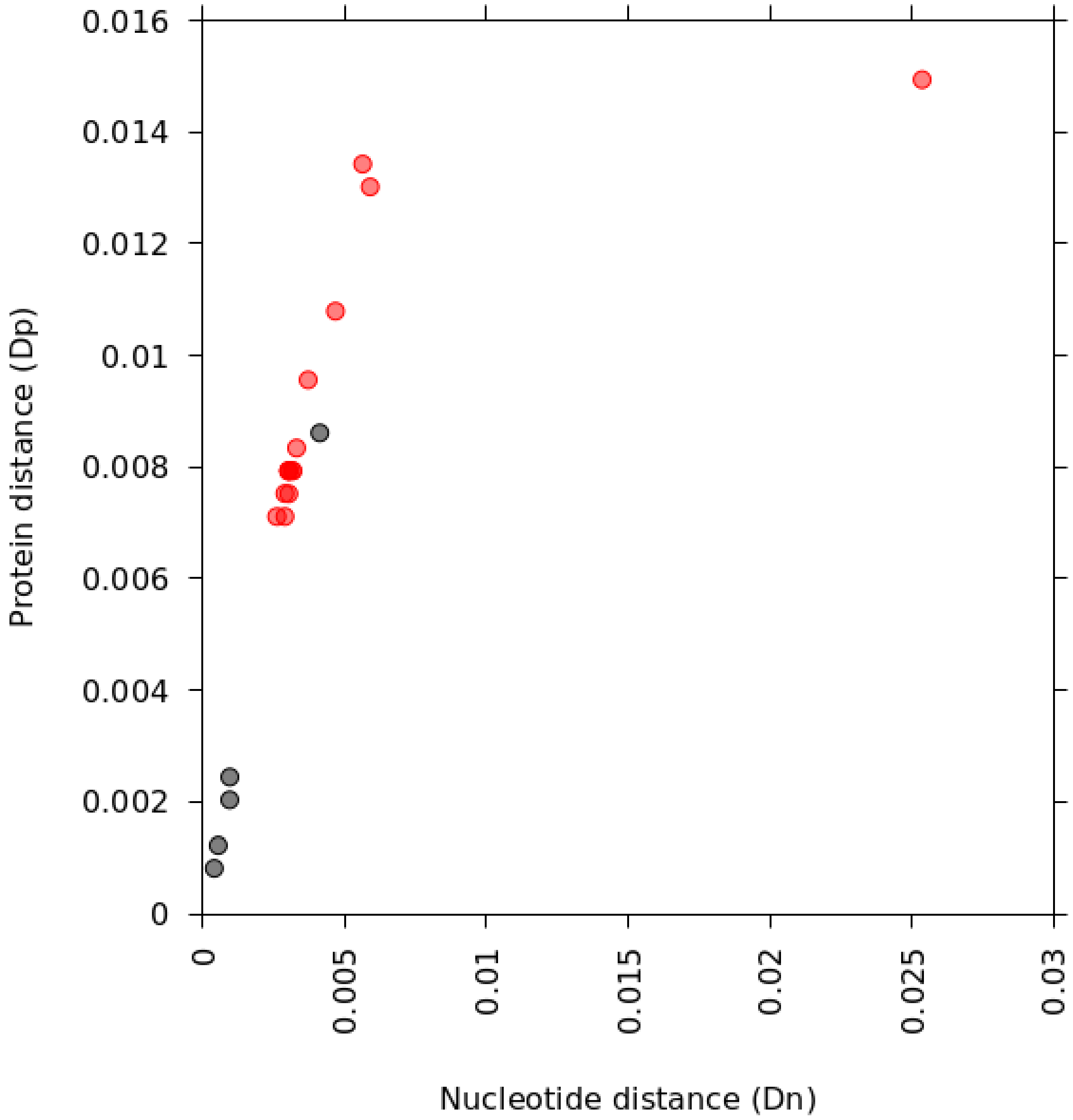

9. NC_007362.1/YP_308669.1

**CDS cluster ID**	9
**CDS cluster name**	NC_007362.1/YP_308669.1
**Total sequences**	145
**Reference forms**	129
**Compensatory frameshift forms**	14
**Virus**	Influenza A virus (A/goose/Guangdong/1/1996(H5N1))
**Protein**	hemagglutinin

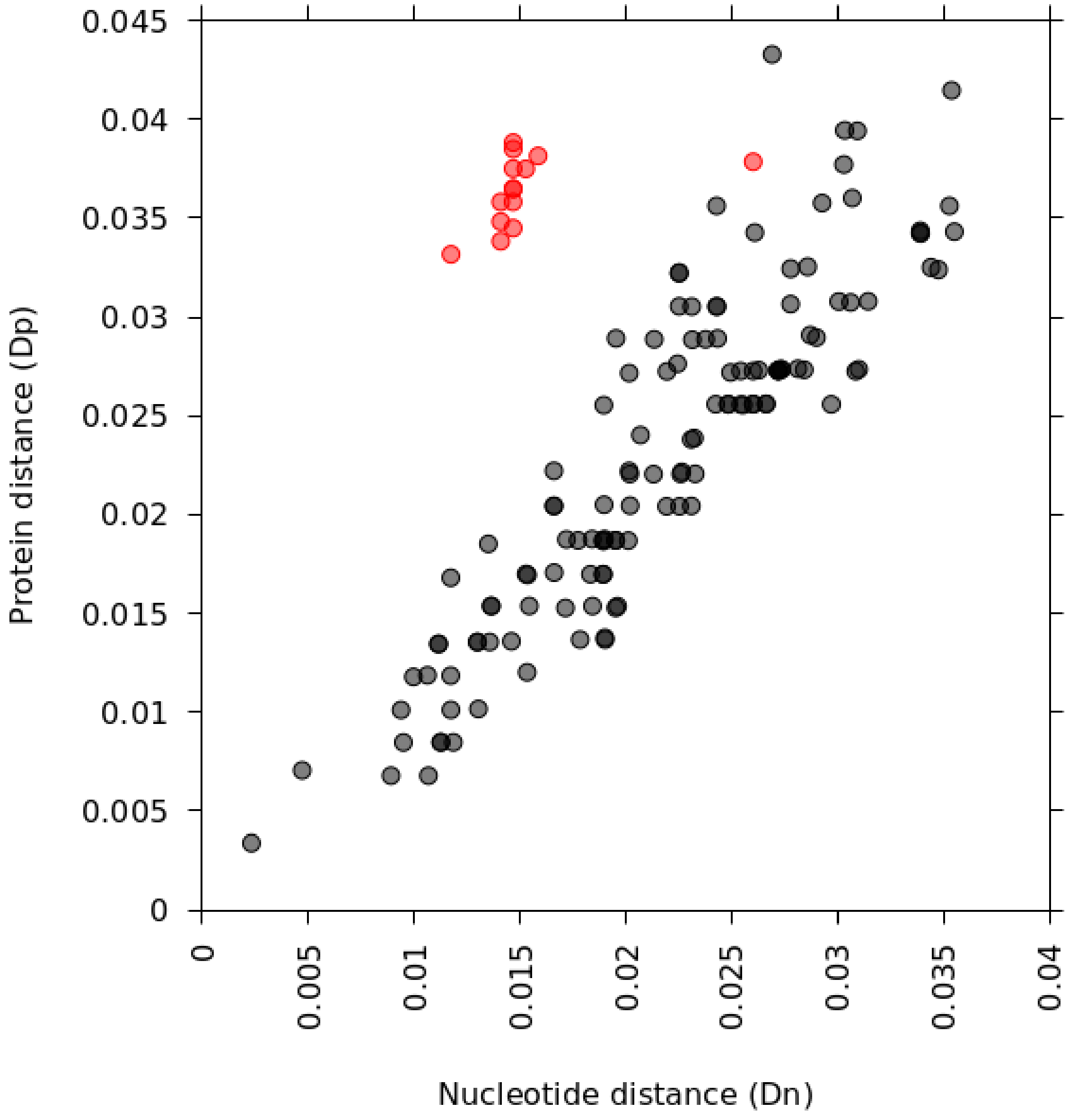

10. NC_001616.1/NP_056759.1

**CDS cluster ID**	**10**
**CDS cluster name**	NC_001616.1/NP_056759.1
**Total sequences**	13
**Reference forms**	2
**Compensatory frameshift forms**	11
**Virus**	Potato virus Y
**Protein**	polyprotein

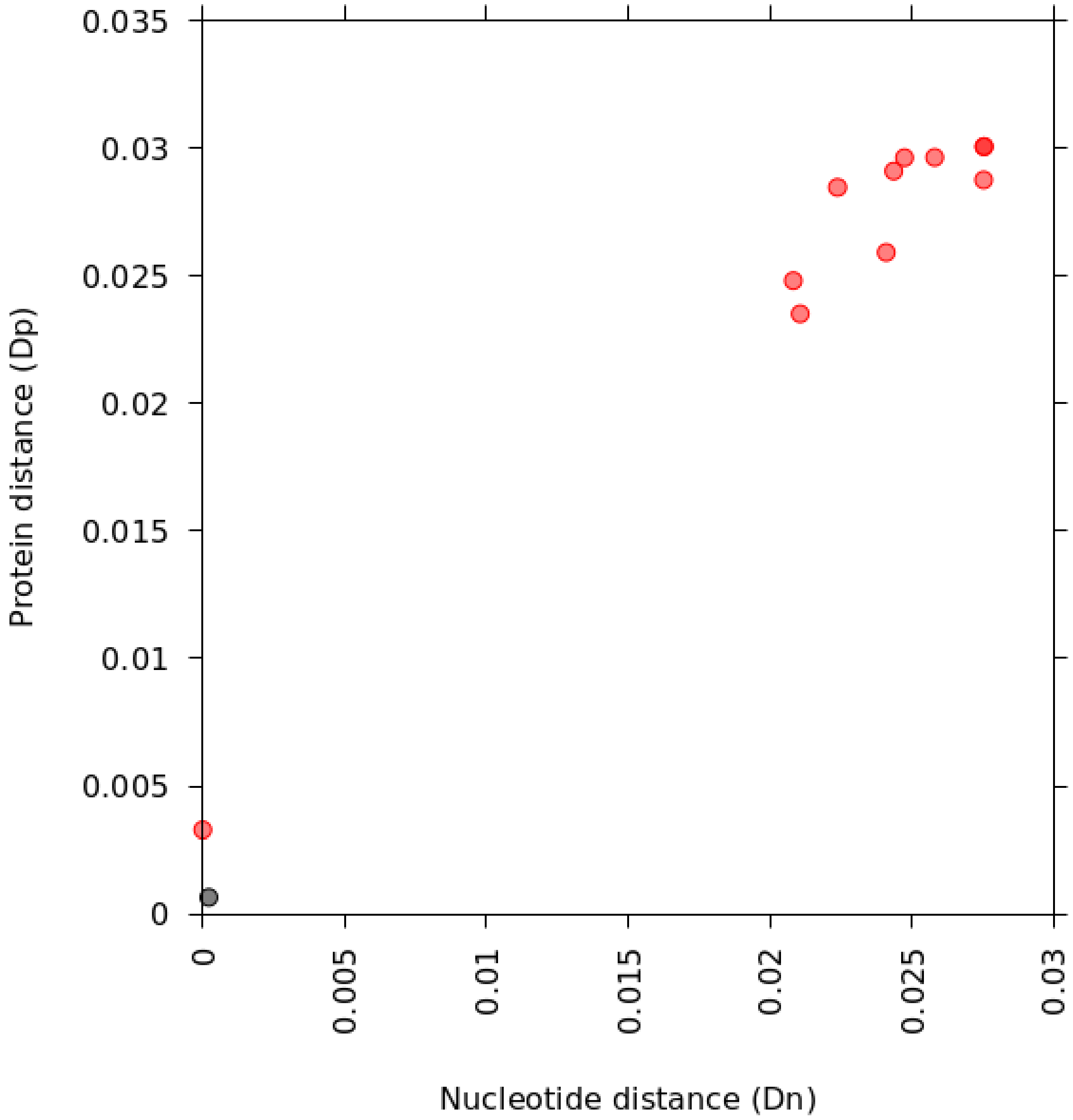

14. NC_003624.1/NP_619708.1

**CDS cluster ID**	**14**
**CDS cluster name**	NC_003624.1/NP_619708.1
**Total sequences**	12
**Reference forms**	2
**Compensatory frameshift forms**	10
**Virus**	Impatiens necrotic spot virus
**Protein**	non-structural protein

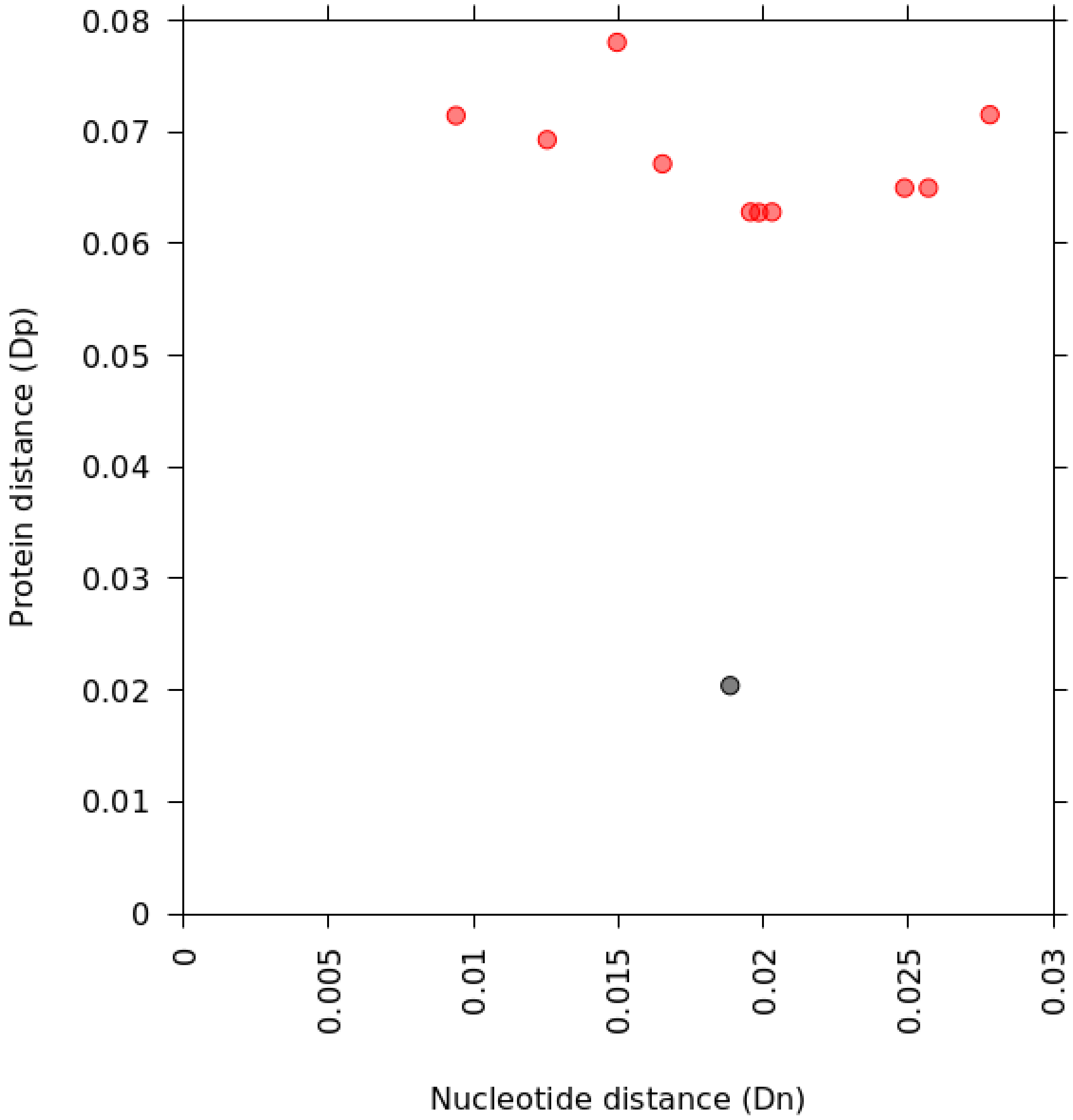

15. NC_001661.1/NP_042860.2

**CDS cluster ID**	**15**
**CDS cluster name**	NC_001661.1/NP_042860.2
**Total sequences**	12
**Reference forms**	2
**Compensatory frameshift forms**	9
**Virus**	Citrus tristeza virus
**Protein**	401-kDa viral polyprotein

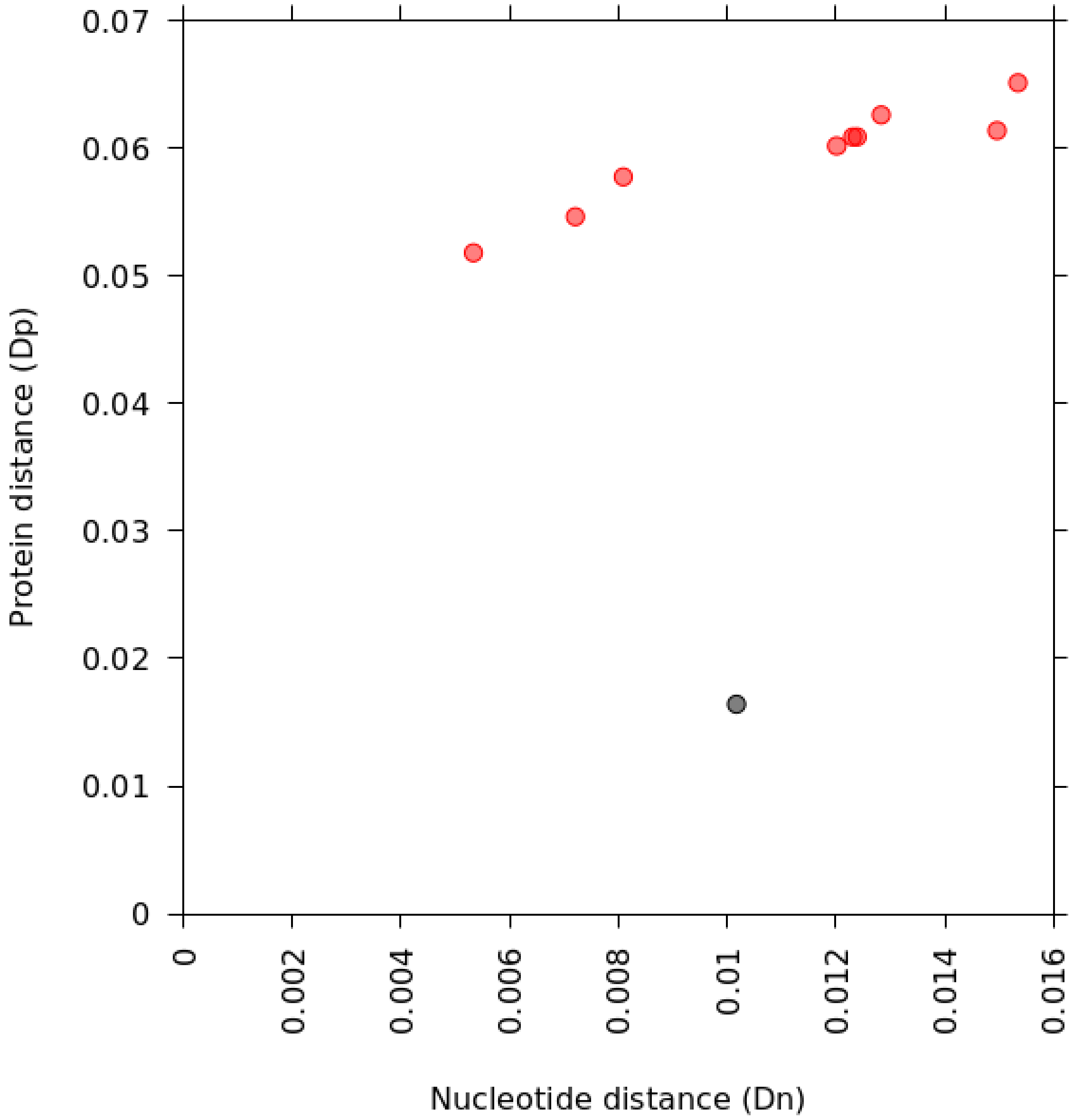

18. NC_004039.1/NP_659729.1

**CDS cluster ID**	**18**
**CDS cluster name**	NC_004039.1/NP_659729.1
**Total sequences**	11
**Reference forms**	4
**Compensatory frameshift forms**	7
**Virus**	Potato virus A
**Protein**	polyprotein

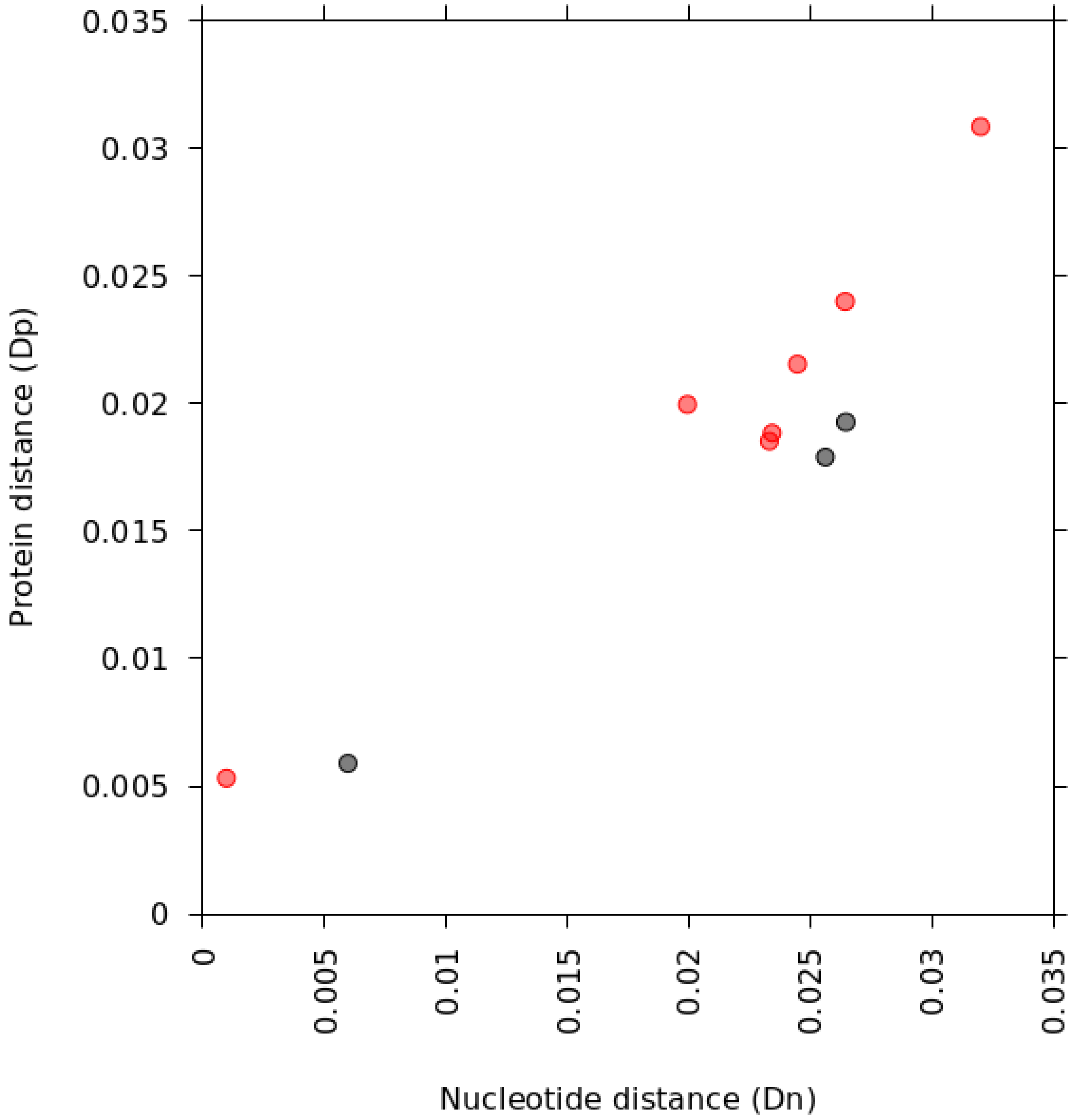

25. NC_004297.1/NP_694872.1

**CDS cluster ID**	**25**
**CDS cluster name**	NC_004297.1/NP_694872.1
**Total sequences**	6
**Reference forms**	2
**Compensatory frameshift forms**	4
**Virus**	Lassa mammarenavirus
**Protein**	L protein

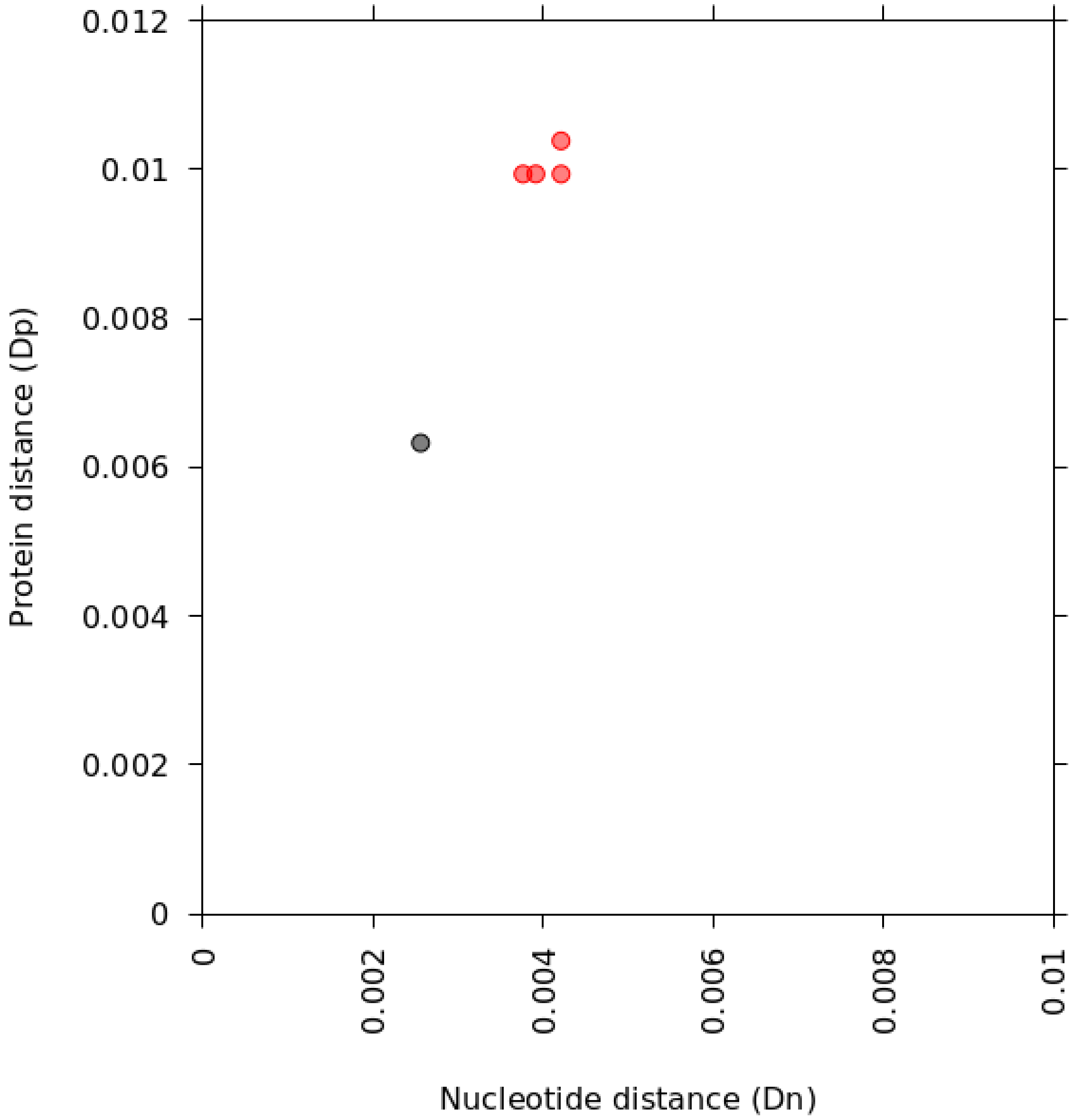

28. NC_001607.1/NP_042021.1

**CDS cluster ID**	**28**
**CDS cluster name**	NC_001607.1/NP_042021.1
**Total sequences**	50
**Reference forms**	46
**Compensatory frameshift forms**	3
**Virus**	Borna disease virus 1 (BoDV-1)
**Protein**	phosphoprotein

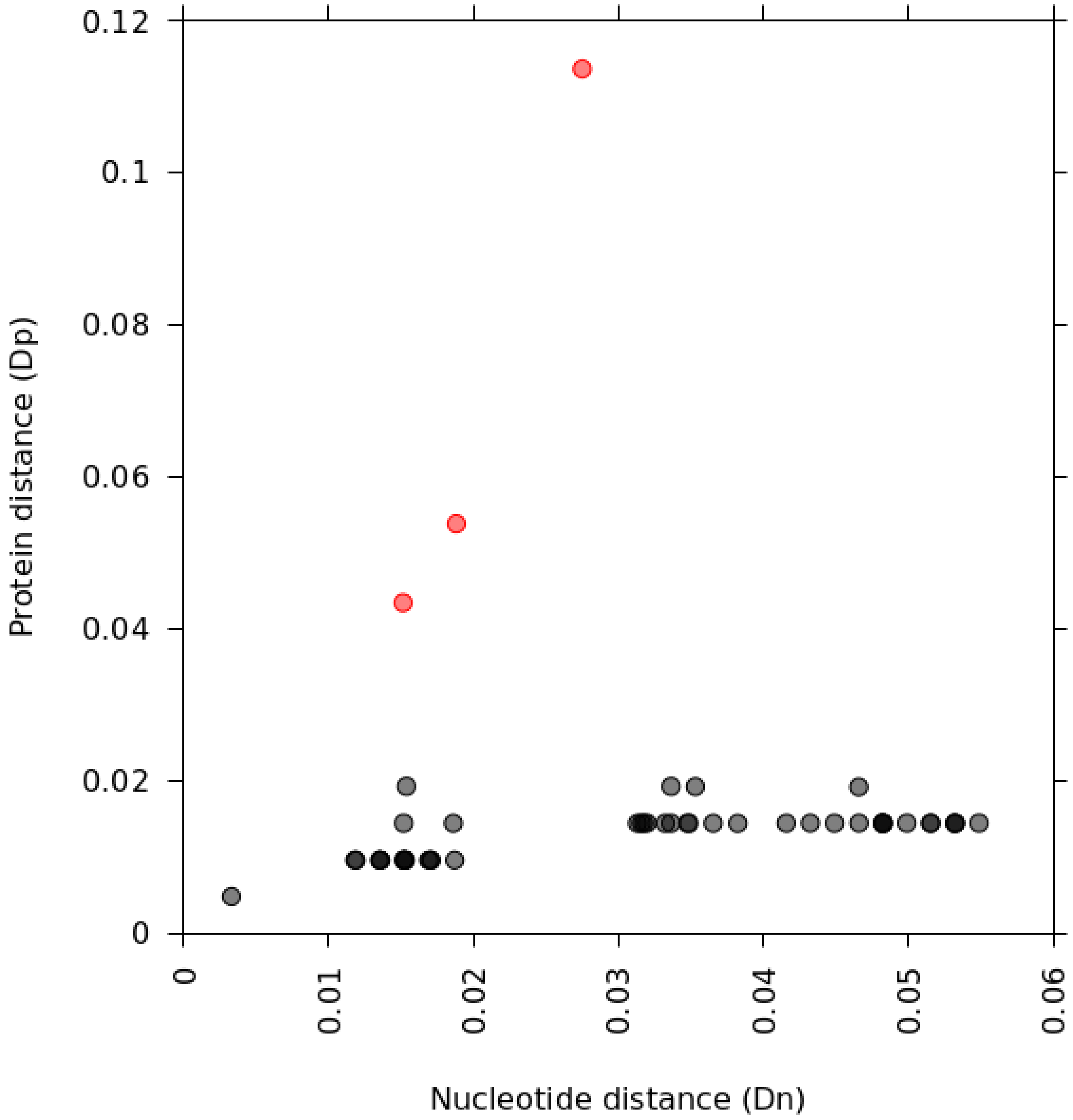

29. NC_002024.2/YP_053235.1

**CDS cluster ID**	**29**
**CDS cluster name**	NC_002024.2/YP_053235.1
**Total sequences**	5
**Reference forms**	2
**Compensatory frameshift forms**	3
**Virus**	Alfalfa mosaic virus
**Protein**	89.7 kd protein

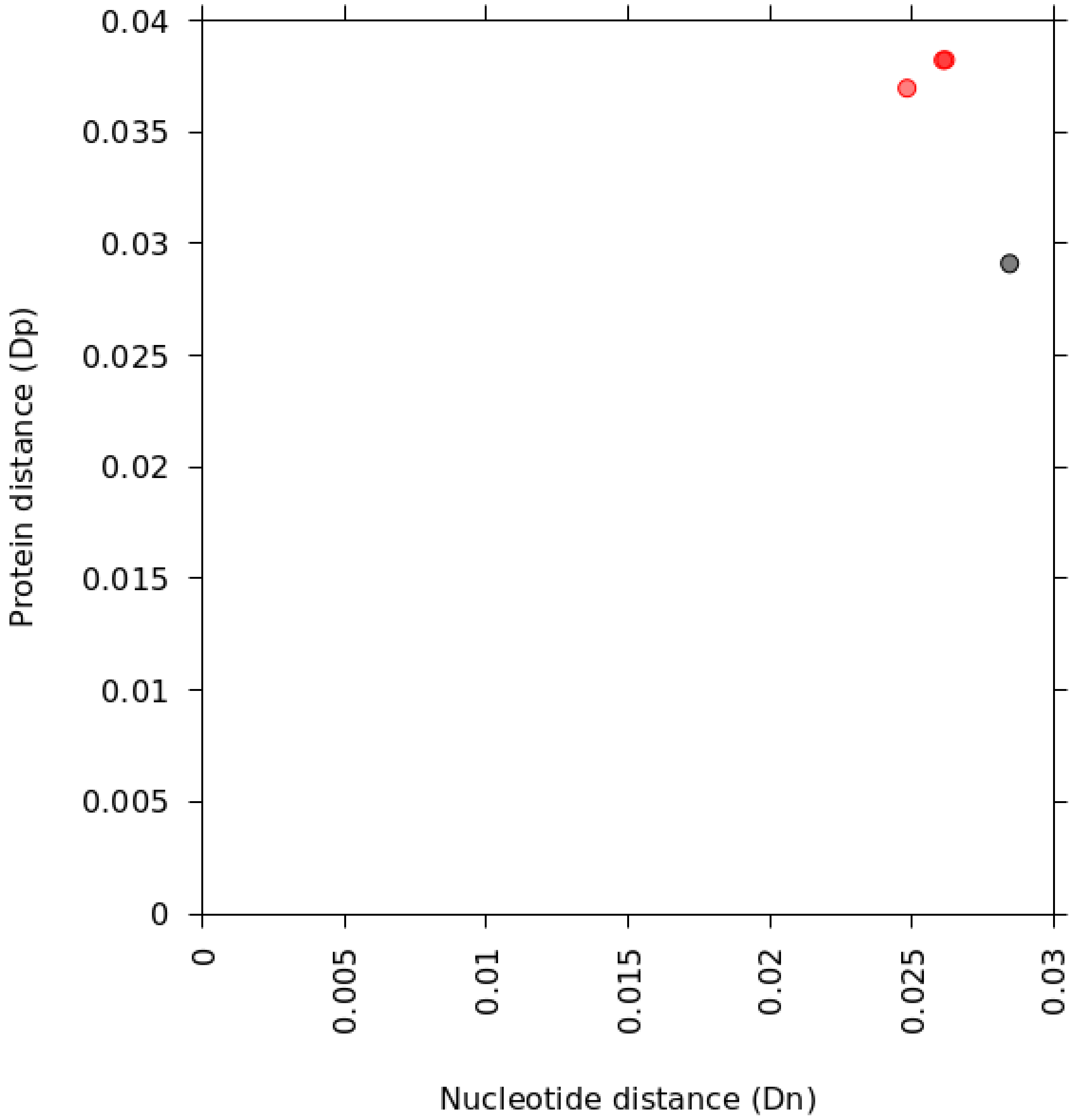

33. NC_005036.1/NP_872277.1

**CDS cluster ID**	33
**CDS cluster name**	NC_005036.1/NP_872277.1
**Total sequences**	242
**Reference forms**	239
**Compensatory frameshift forms**	3
**Virus**	Goose paramyxovirus SF02
**Protein**	hemagglutinin-neuraminidase

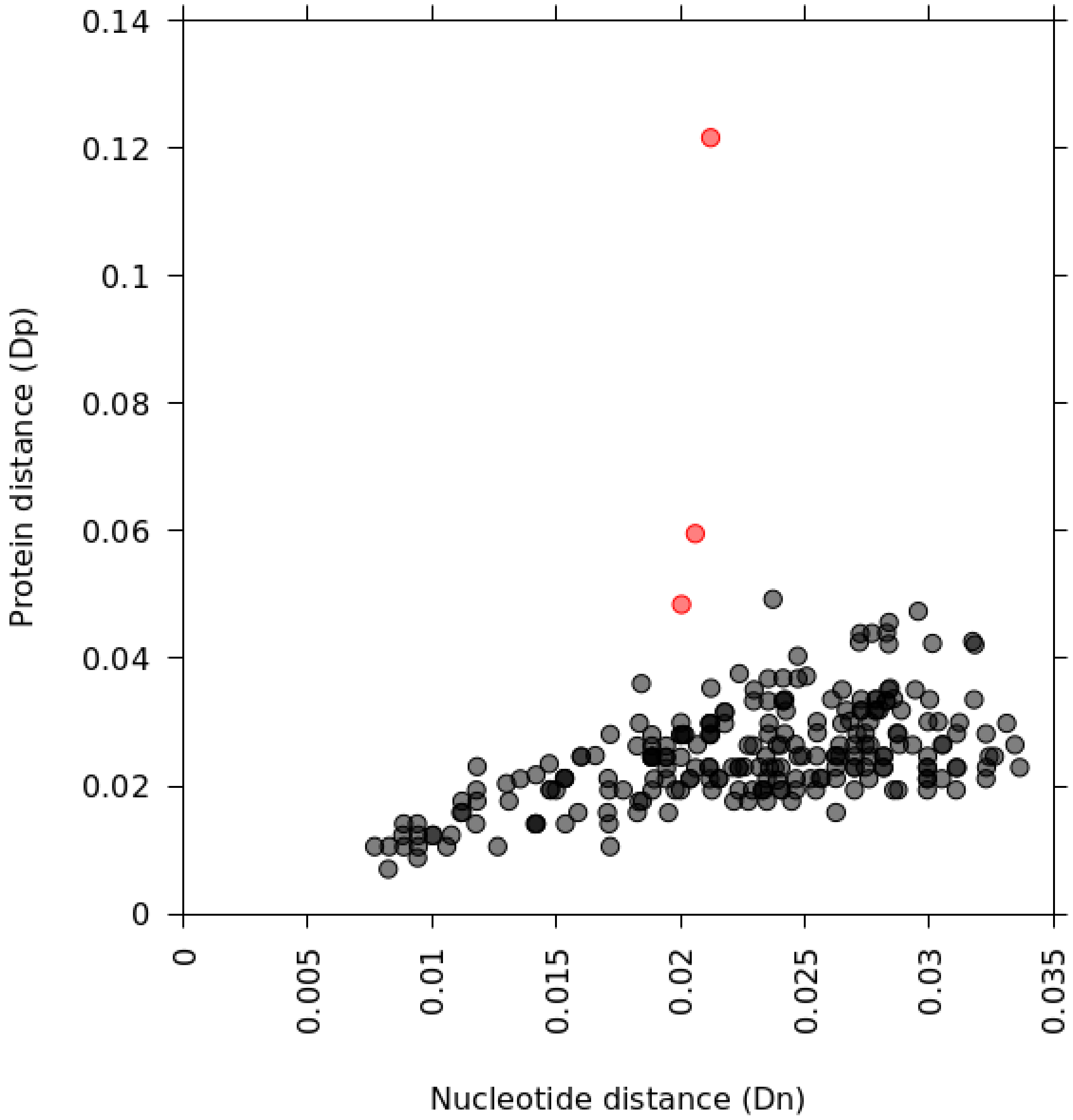

34. NC_005138.1/NP_932792.1

CDS cluster ID	34
CDS cluster name	NC_005138.1/NP_932792.1
Total sequences	43
Reference forms	40
Compensatory frameshift forms	3
Virus	Lily symptomless virus
Protein	coat protein

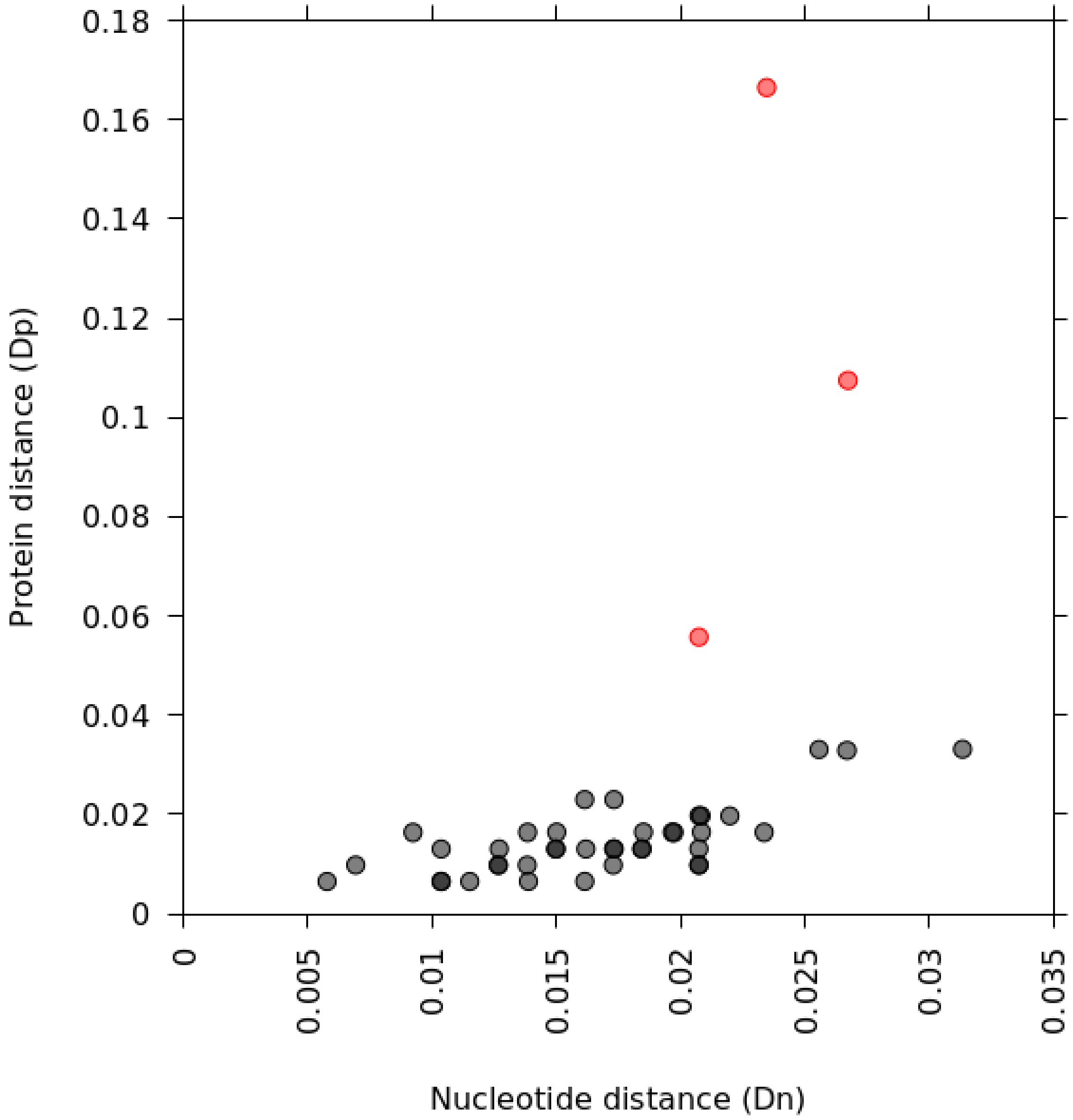

36. NC_009640.1/YP_001331032.1

**CDS cluster ID**	**36**
**CDS cluster name**	NC_009640.1/YP_001331032.1
**Total sequences**	5
**Reference forms**	2
**Compensatory frameshift forms**	3
**Virus**	Porcine rubulavirus (PorPV)
**Protein**	matrix protein

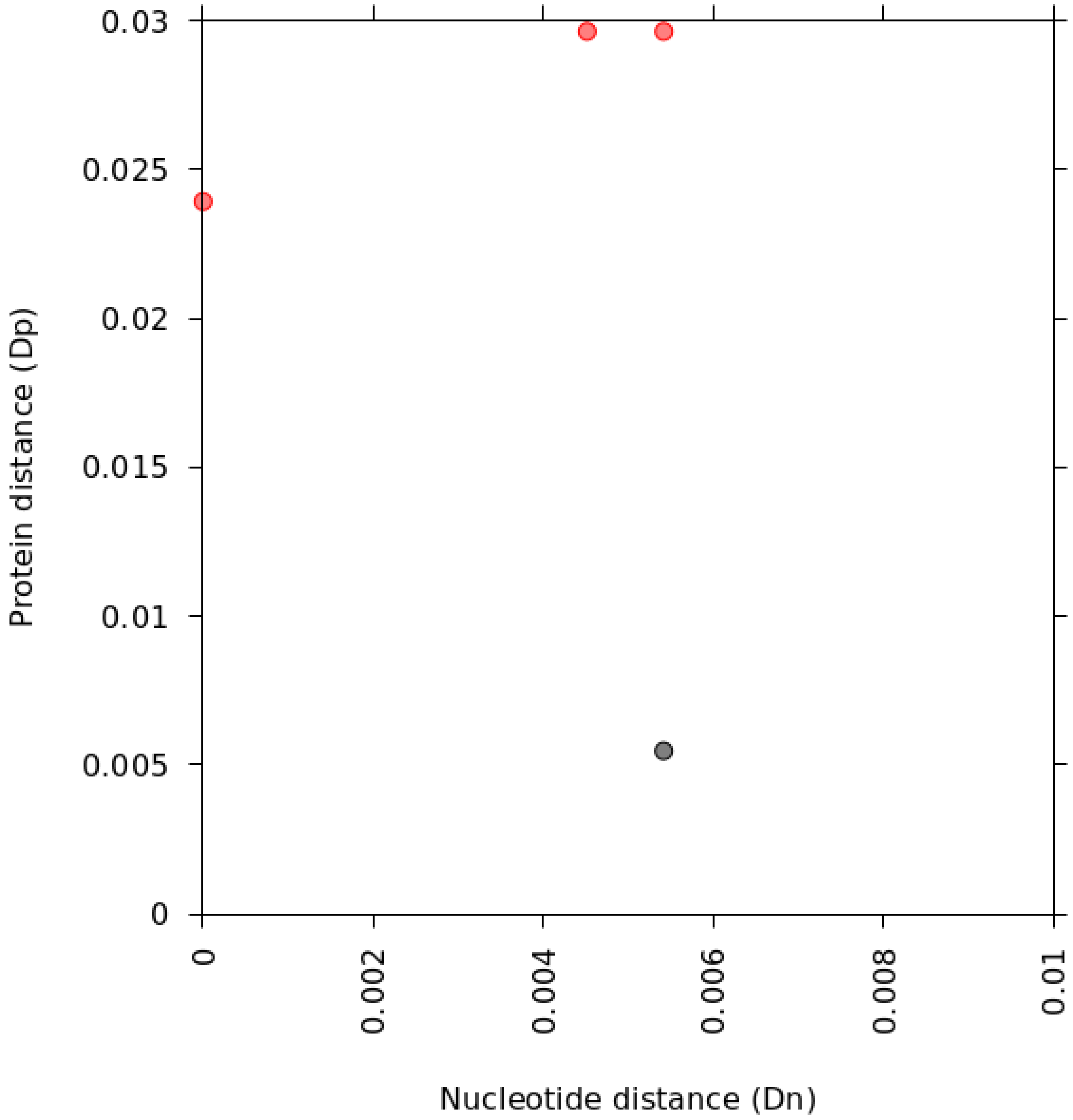

37. NC_026435.1/YP_009118628.1

**CDS cluster ID**	**37**
**CDS cluster name**	NC_026435.1/YP_009118628.1
**Total sequences**	5498
**Reference forms**	5493
**Compensatory frameshift forms**	3
**Virus**	Influenza A virus (A/California/07/2009(H1N1))
**Protein**	polymerase PB1

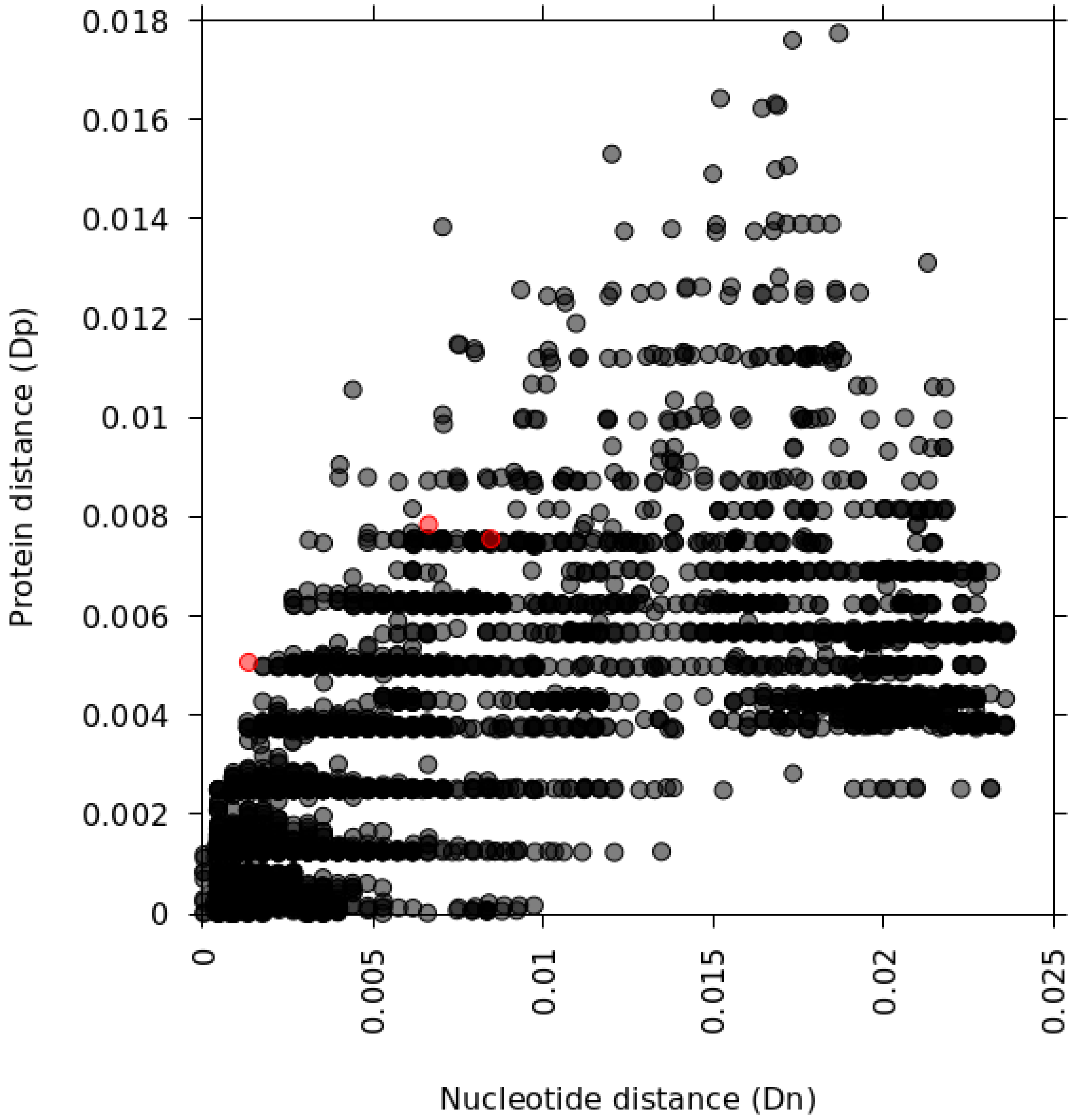

38. NC_039223.1/YP_009513198.1

**CDS cluster ID**	**38**
**CDS cluster name**	NC_039223.1/YP_009513198.1
**Total sequences**	303
**Reference forms**	300
**Compensatory frameshift forms**	3
**Virus**	Avian avulavirus 1
**Protein**	hemagglutinin-neuraminidase

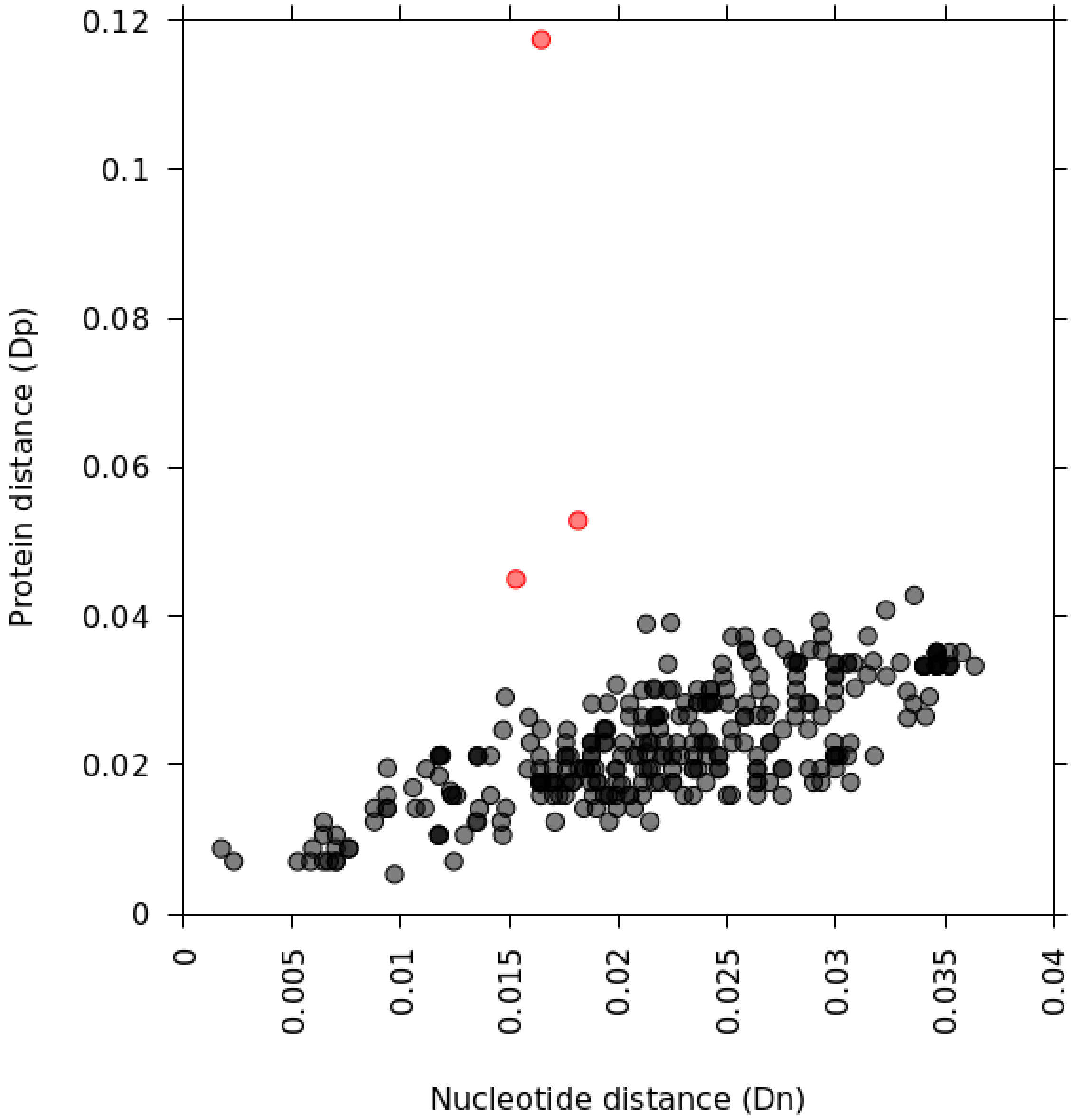

40. NC_001661.1/NP_042867.1

**CDS cluster ID**	**40**
**CDS cluster name**	NC_001661.1/NP_042867.1
**Total sequences**	55
**Reference forms**	53
**Compensatory frameshift forms**	2
**Virus**	Citrus tristeza virus
**Protein**	25-kDa coat protein

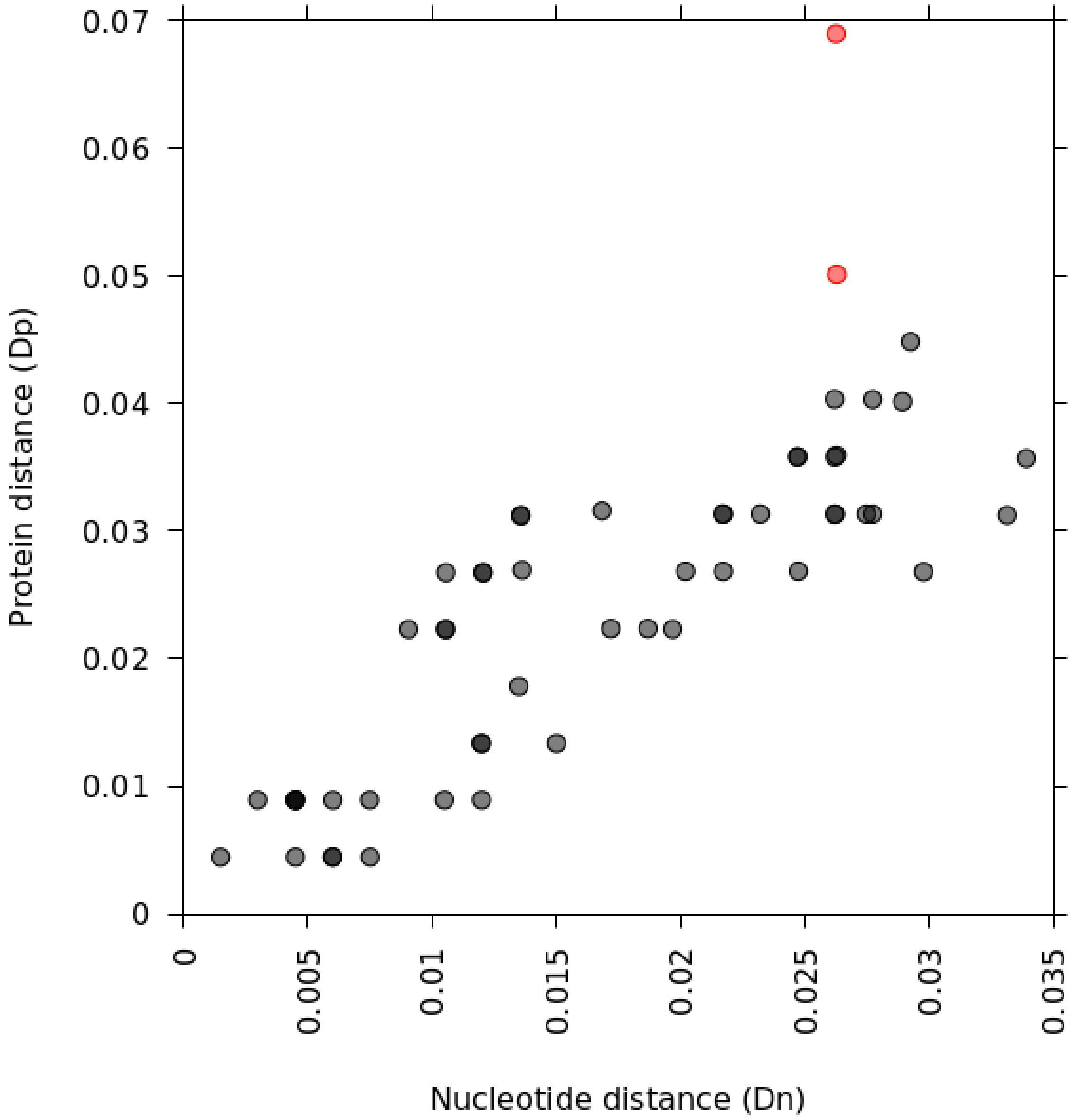

41. NC_002350.1/NP_059449.1

**CDS cluster ID**	**41**
**CDS cluster name**	NC_002350.1/NP_059449.1
**Total sequences**	26
**Reference forms**	24
**Compensatory frameshift forms**	2
**Virus**	Wheat yellow mosaic virus
**Protein**	polyprotein

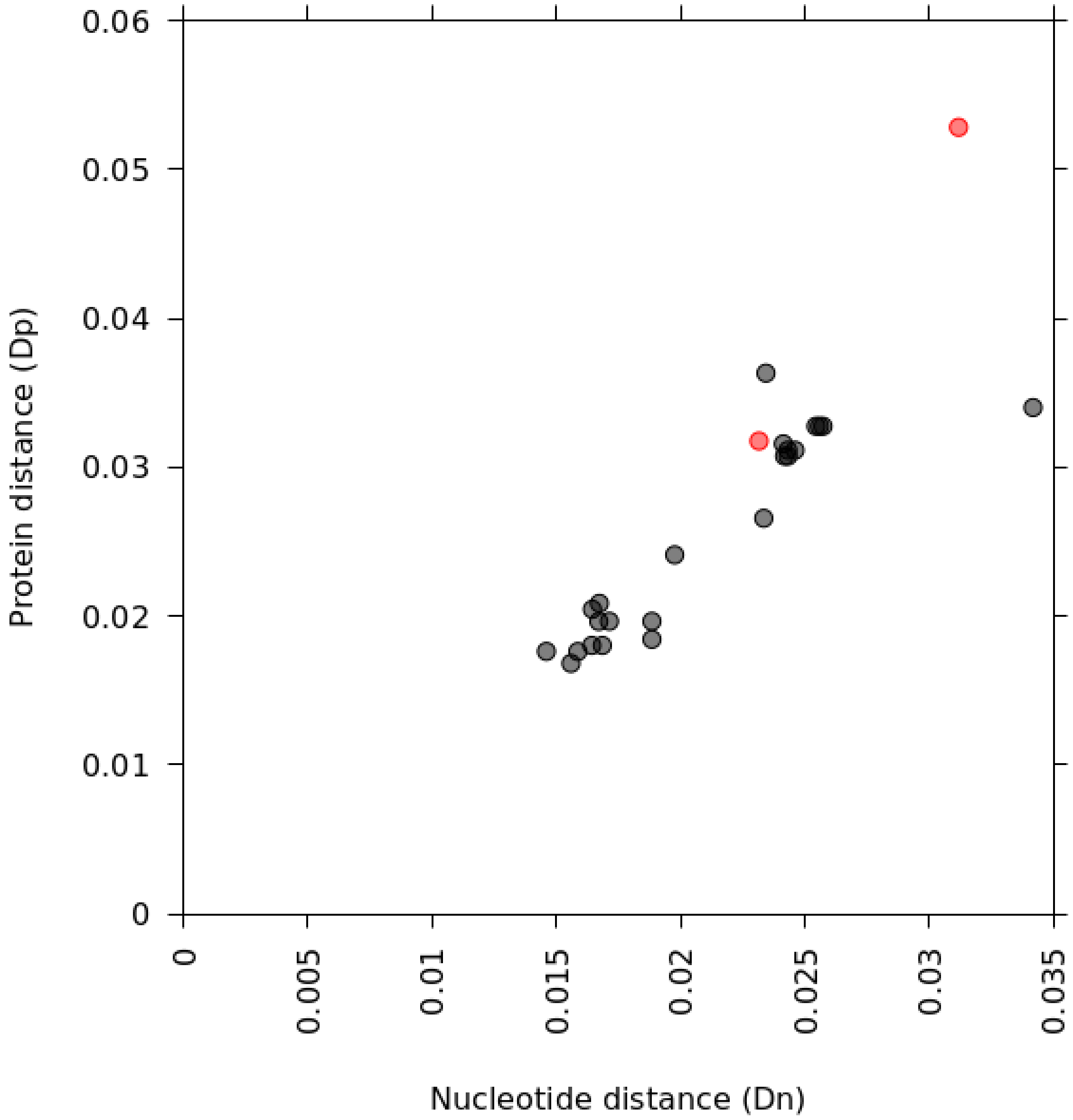

42. NC_002549.1/NP_066249.1

**CDS cluster ID**	**42**
**CDS cluster name**	NC_002549.1/NP_066249.1
**Total sequences**	159
**Reference forms**	137
**Compensatory frameshift forms**	2
**Virus**	Zaire ebolavirus
**Protein**	minor nucleoprotein

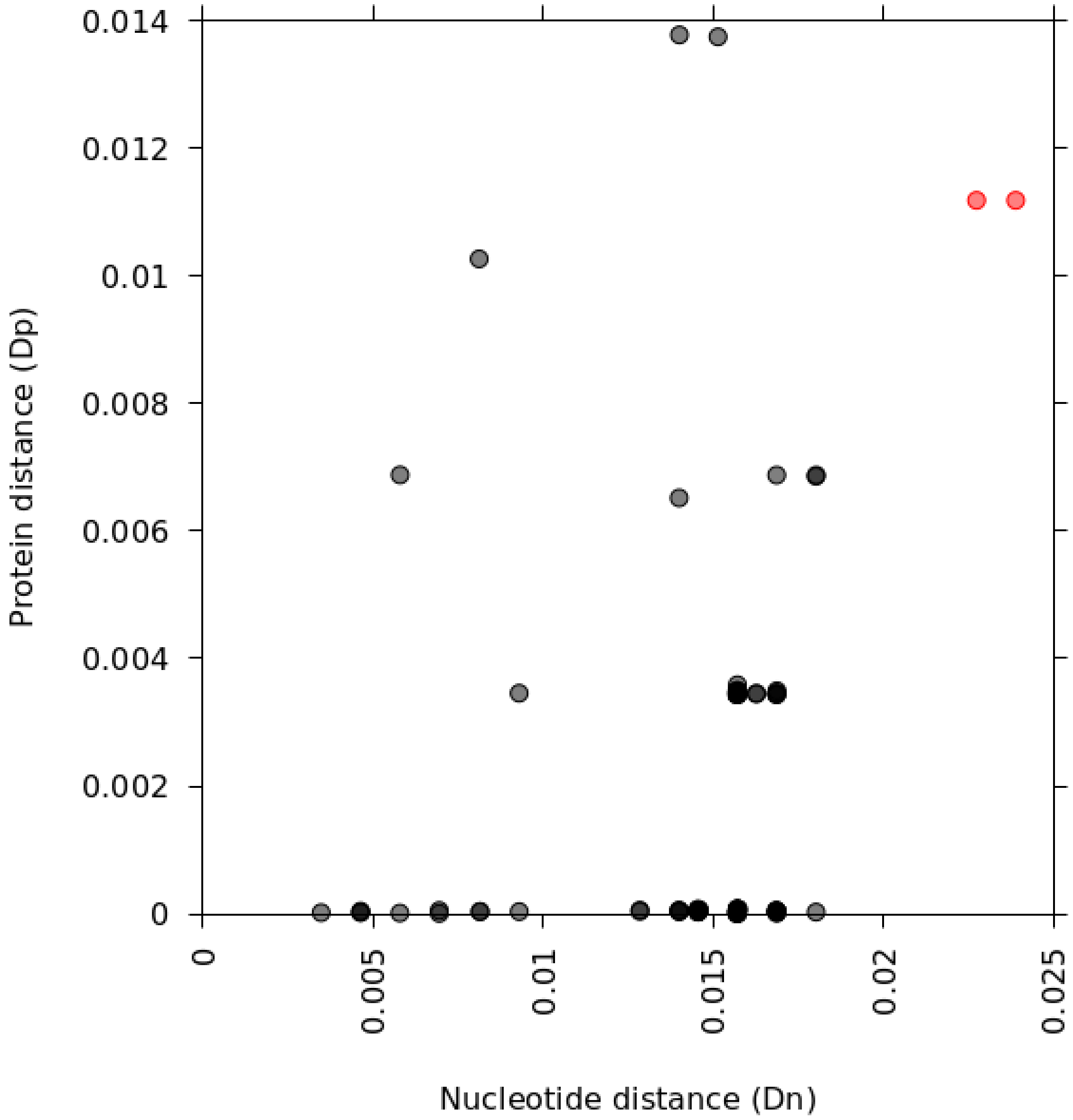

43. NC_003045.1/NP_150075.1

**CDS cluster ID**	**43**
**CDS cluster name**	NC_003045.1/NP_150075.1
**Total sequences**	83
**Reference forms**	81
**Compensatory frameshift forms**	2
**Virus**	Bovine coronavirus
**Protein**	32 kDa non-structural protein

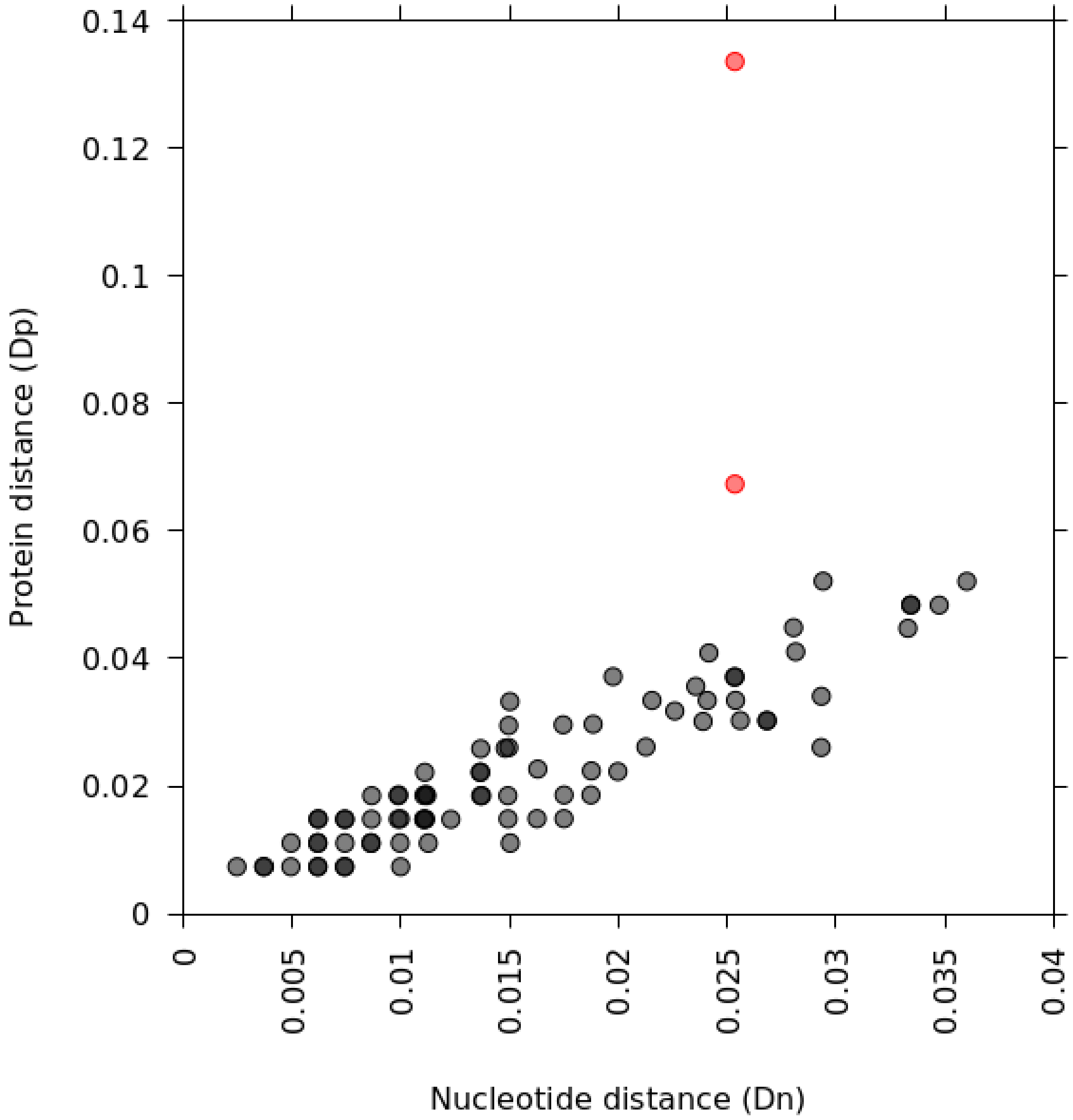

46. NC_003770.1/NP_620540.1

**CDS cluster ID**	**46**
**CDS cluster name**	NC_003770.1/NP_620540.1
**Total sequences**	4
**Reference forms**	2
**Compensatory frameshift forms**	2
**Virus**	Rice ragged stunt virus
**Protein**	Pns7

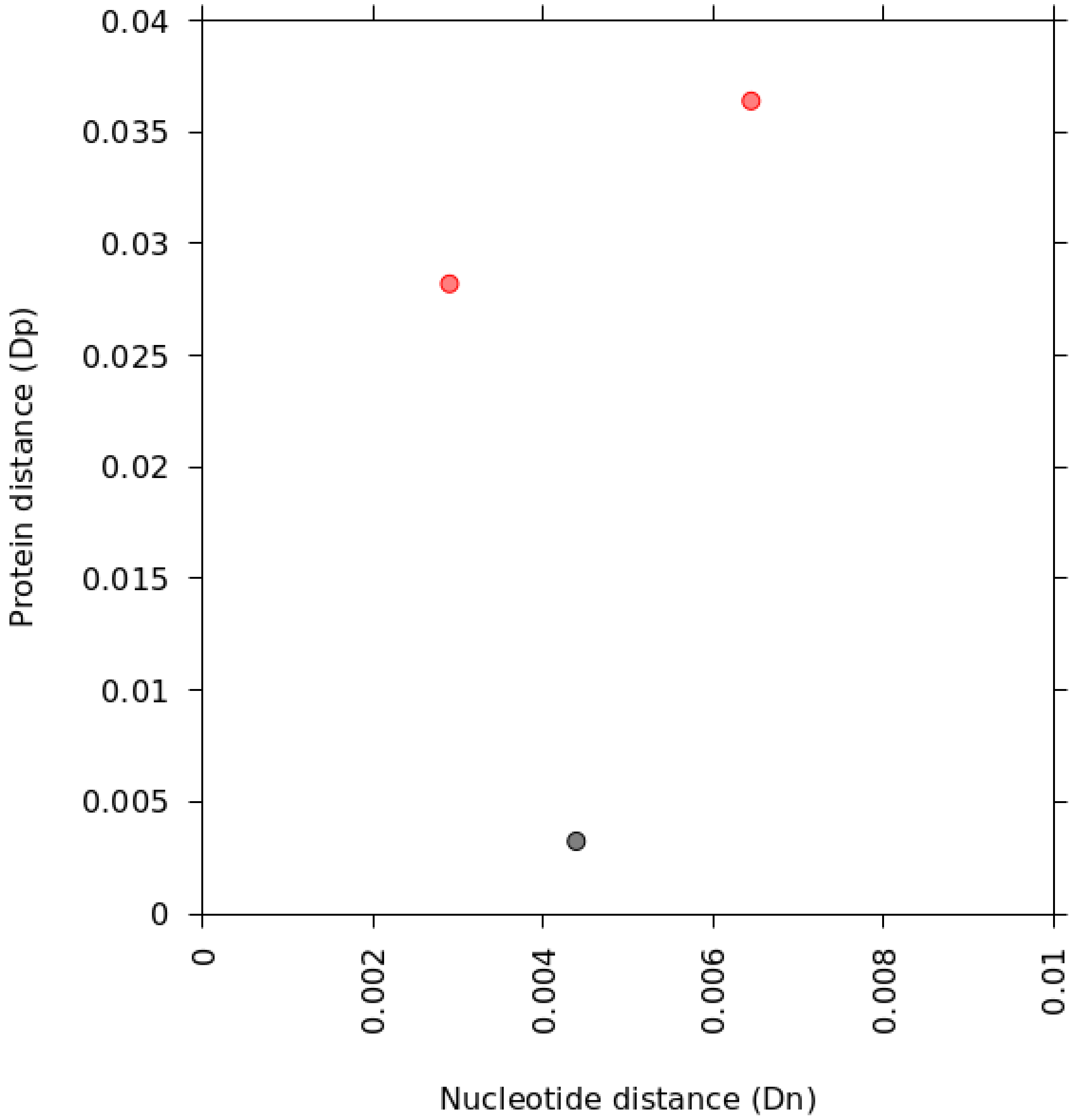

51. NC_005138.1/NP_932788.1

CDS cluster ID	51
CDS cluster name	NC_005138.1/NP_932788.1
Total sequences	10
Reference forms	8
Compensatory frameshift forms	2
Virus	Lily symptomless virus
Protein	RNA-dependent RNA polymerase

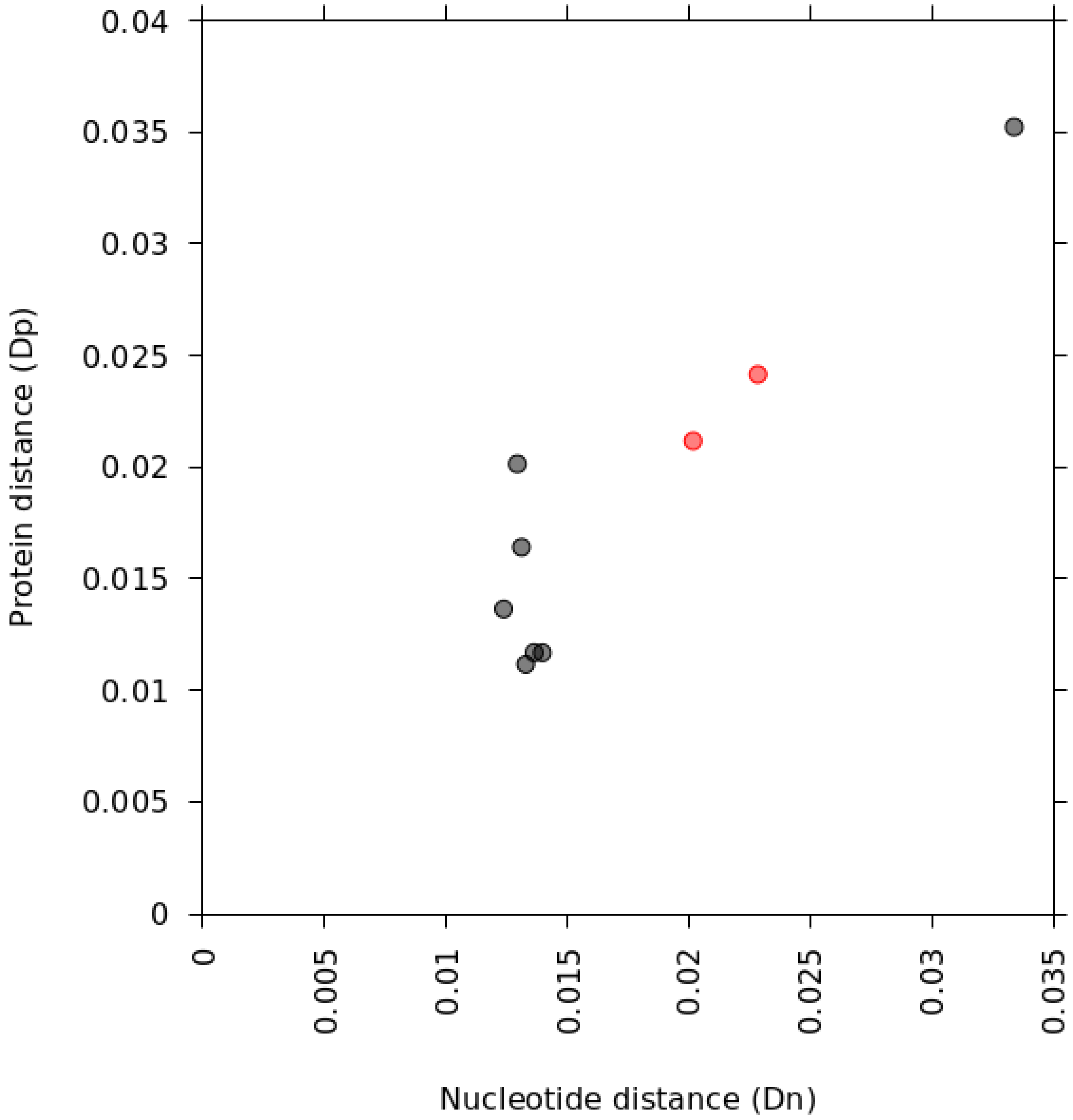

52. NC_005236.1/NP_942556.1

**CDS cluster ID**	**52**
**CDS cluster name**	NC_005236.1/NP_942556.1
**Total sequences**	48
**Reference forms**	46
**Compensatory frameshift forms**	2
**Virus**	Seoul orthohantavirus
**Protein**	nucleocapsid protein

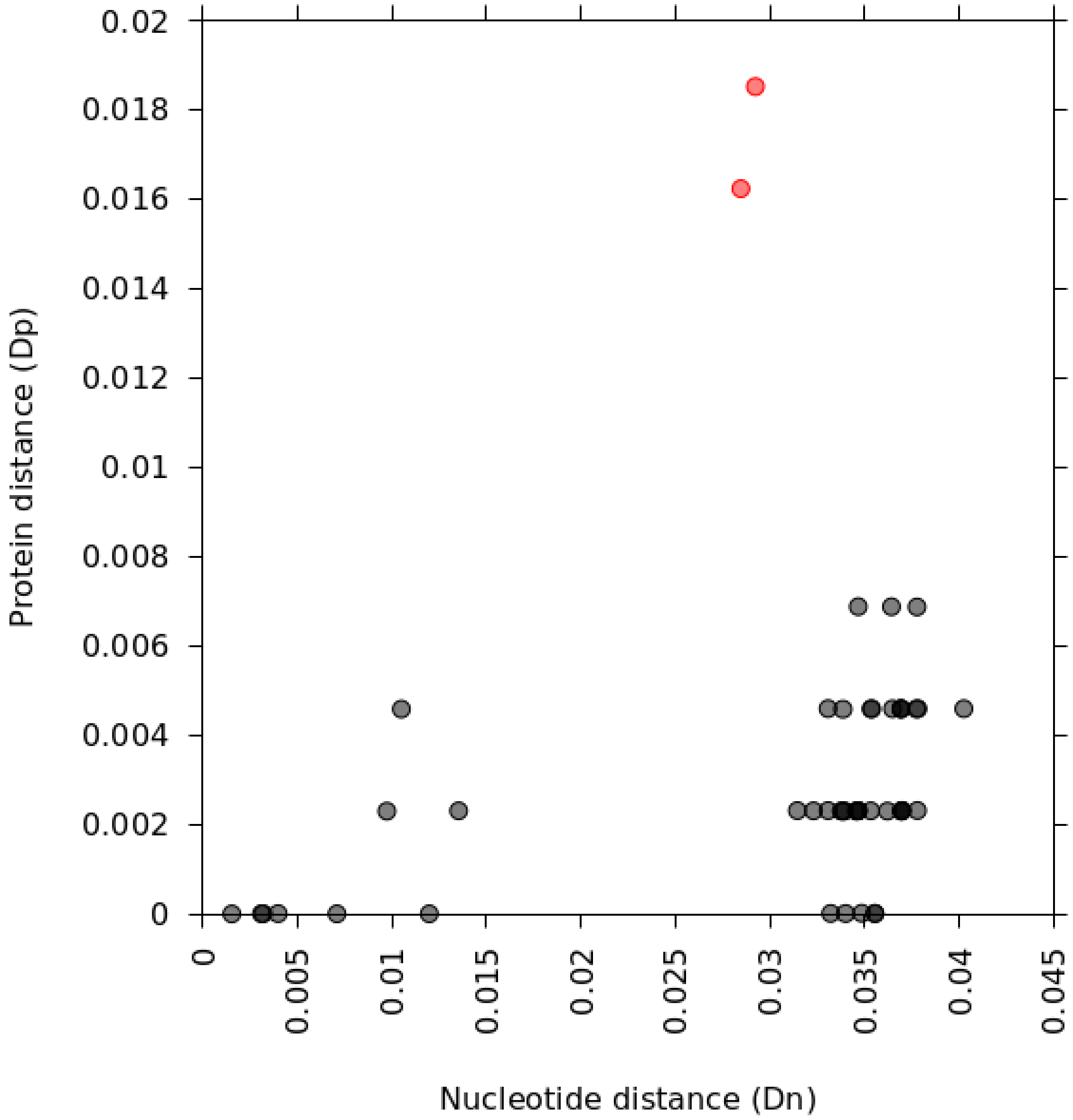

53. NC_005831.2/YP_003766.2

**CDS cluster ID**	**53**
**CDS cluster name**	NC_005831.2/YP_003766.2
**Total sequences**	56
**Reference forms**	53
**Compensatory frameshift forms**	2
**Virus**	Human coronavirus NL63 (HCoV-NL63)
**Protein**	replicase polyprotein 1ab

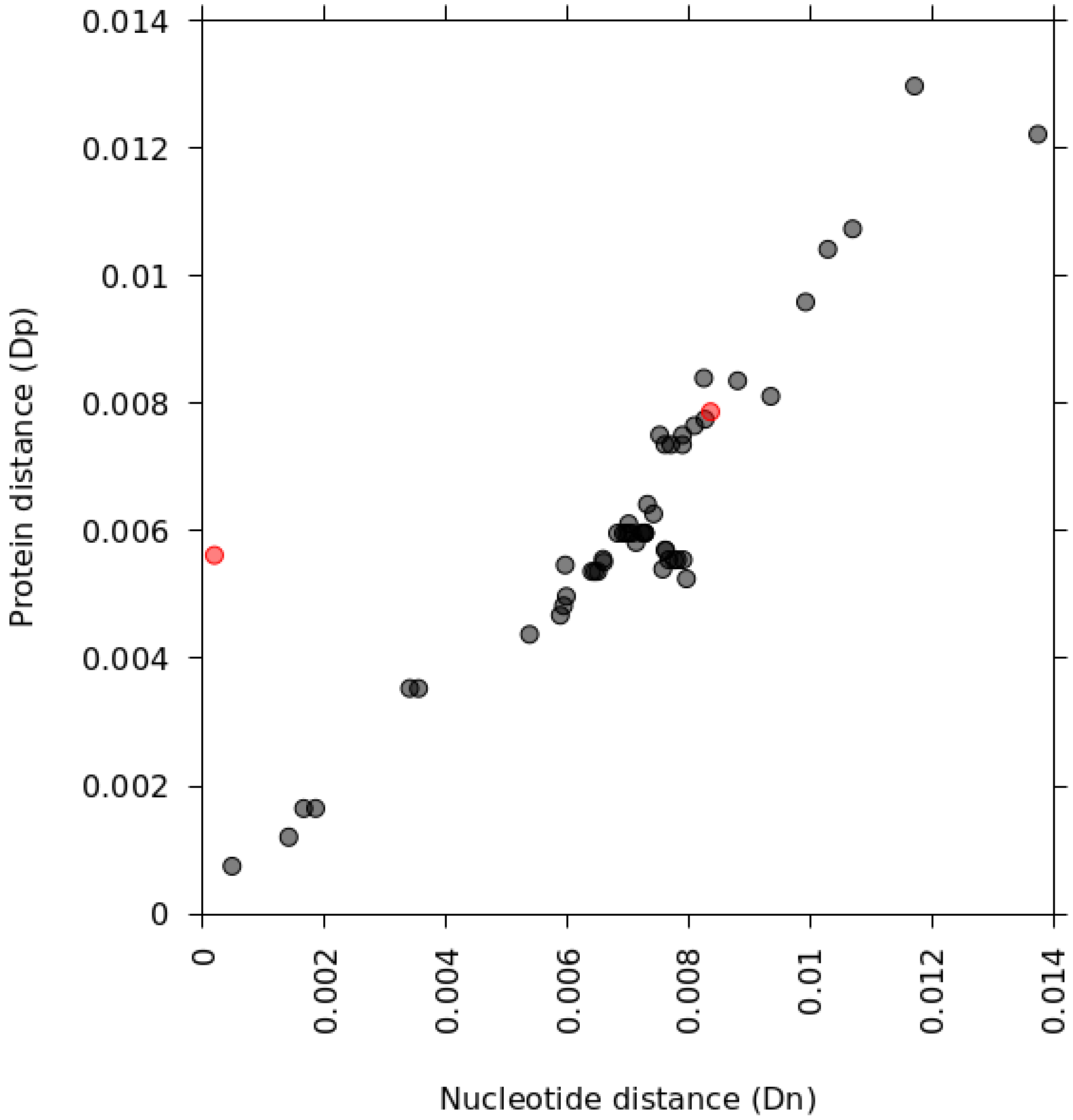

54. NC_006009.1/YP_052954.1

**CDS cluster ID**	**54**
**CDS cluster name**	NC_006009.1/YP_052954.1
**Total sequences**	14
**Reference forms**	12
**Compensatory frameshift forms**	2
**Virus**	African horse sickness virus
**Protein**	NS3 protein

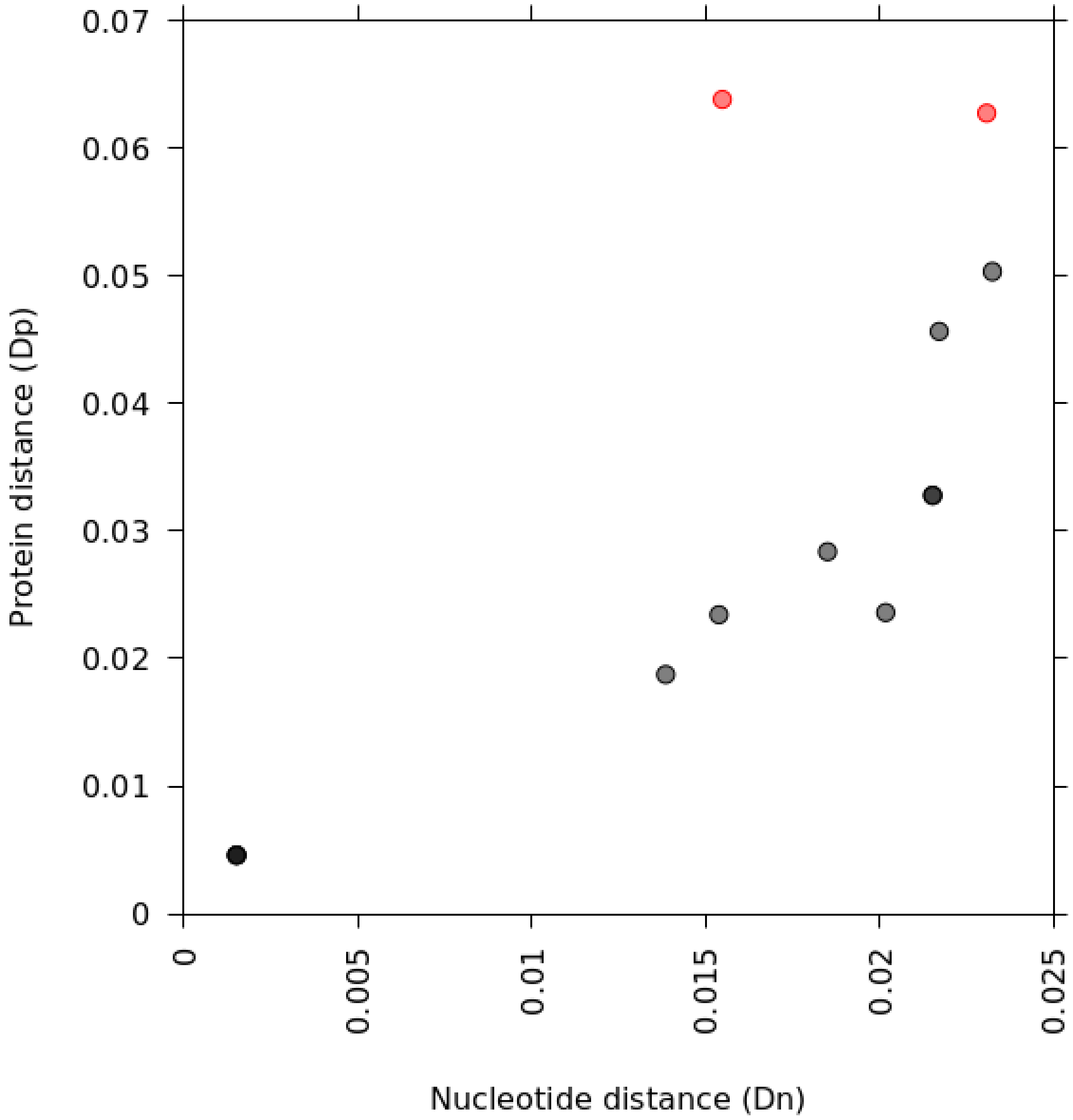

55. NC_006383.2/YP_133826.1

**CDS cluster ID**	**55**
**CDS cluster name**	NC_006383.2/YP_133826.1
**Total sequences**	43
**Reference forms**	41
**Compensatory frameshift forms**	2
**Virus**	Peste des petits ruminants virus
**Protein**	fusion protein

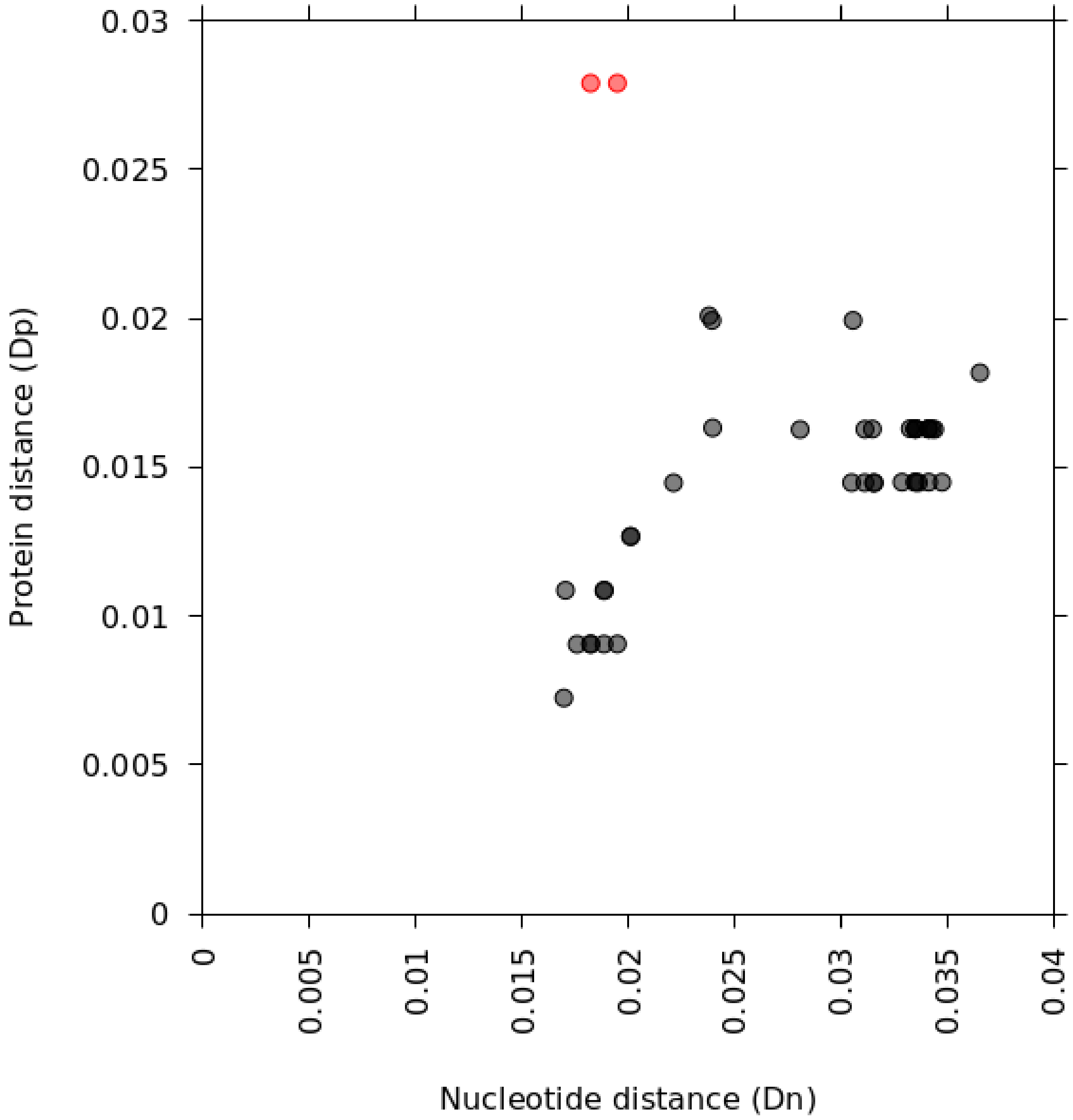

56. NC_008306.1/YP_717933.1

**CDS cluster ID**	**56**
**CDS cluster name**	NC_008306.1/YP_717933.1
**Total sequences**	4
**Reference forms**	2
**Compensatory frameshift forms**	2
**Virus**	Melon yellow spot virus
**Protein**	RNA dependent RNA polymerase

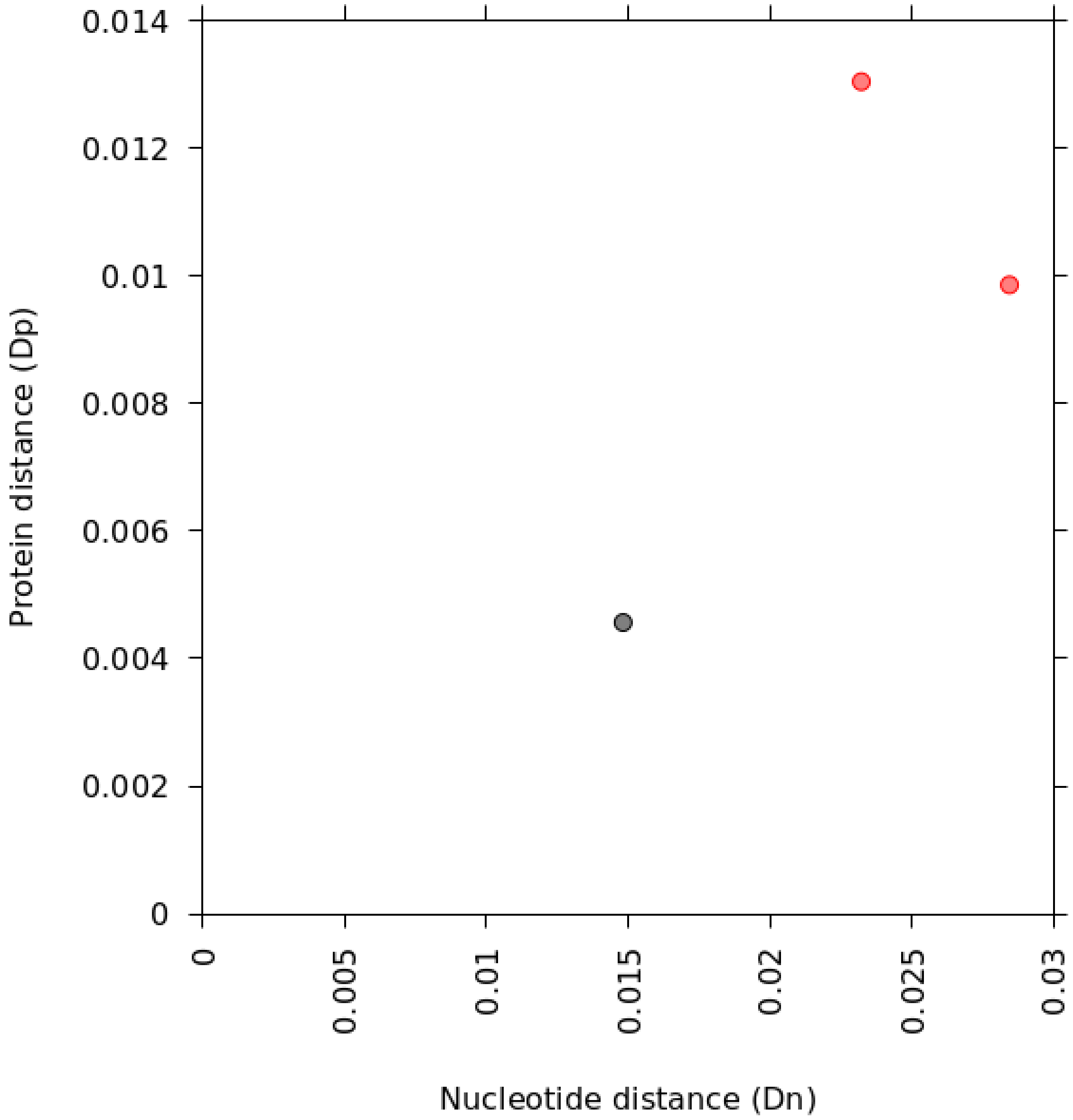

59. NC_009825.1/YP_001469632.1

**CDS cluster ID**	**59**
**CDS cluster name**	NC_009825.1/YP_001469632.1
**Total sequences**	4
**Reference forms**	2
**Compensatory frameshift forms**	2
**Virus**	Hepatitis C virus genotype 4
**Protein**	polyprotein

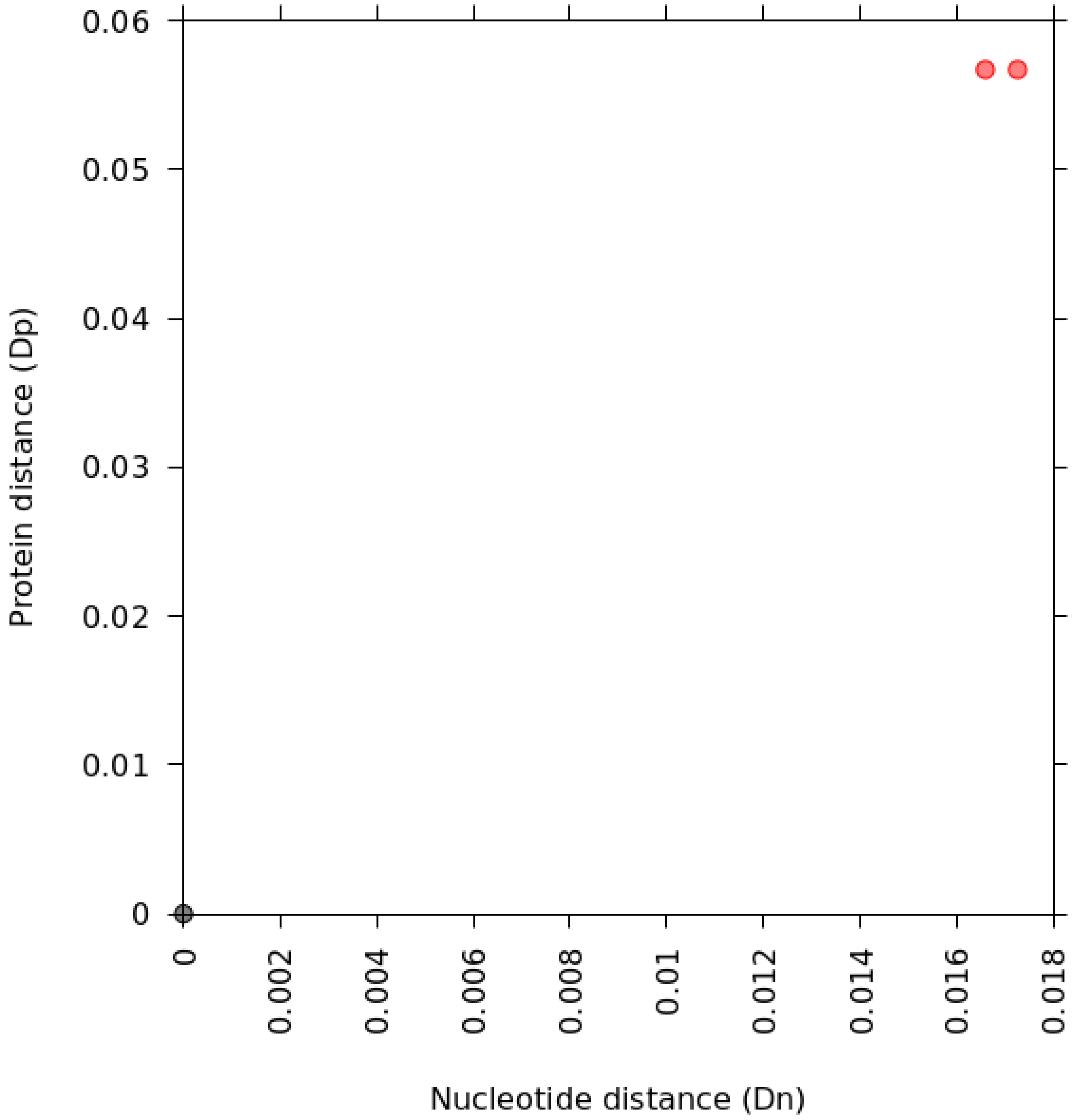

61. NC_011500.2/YP_002302219.1

**CDS cluster ID**	**61**
**CDS cluster name**	NC_011500.2/YP_002302219.1
**Total sequences**	10
**Reference forms**	8
Compensatory frameshift forms	2
**Virus**	Rotavirus A
**Protein**	NSP1

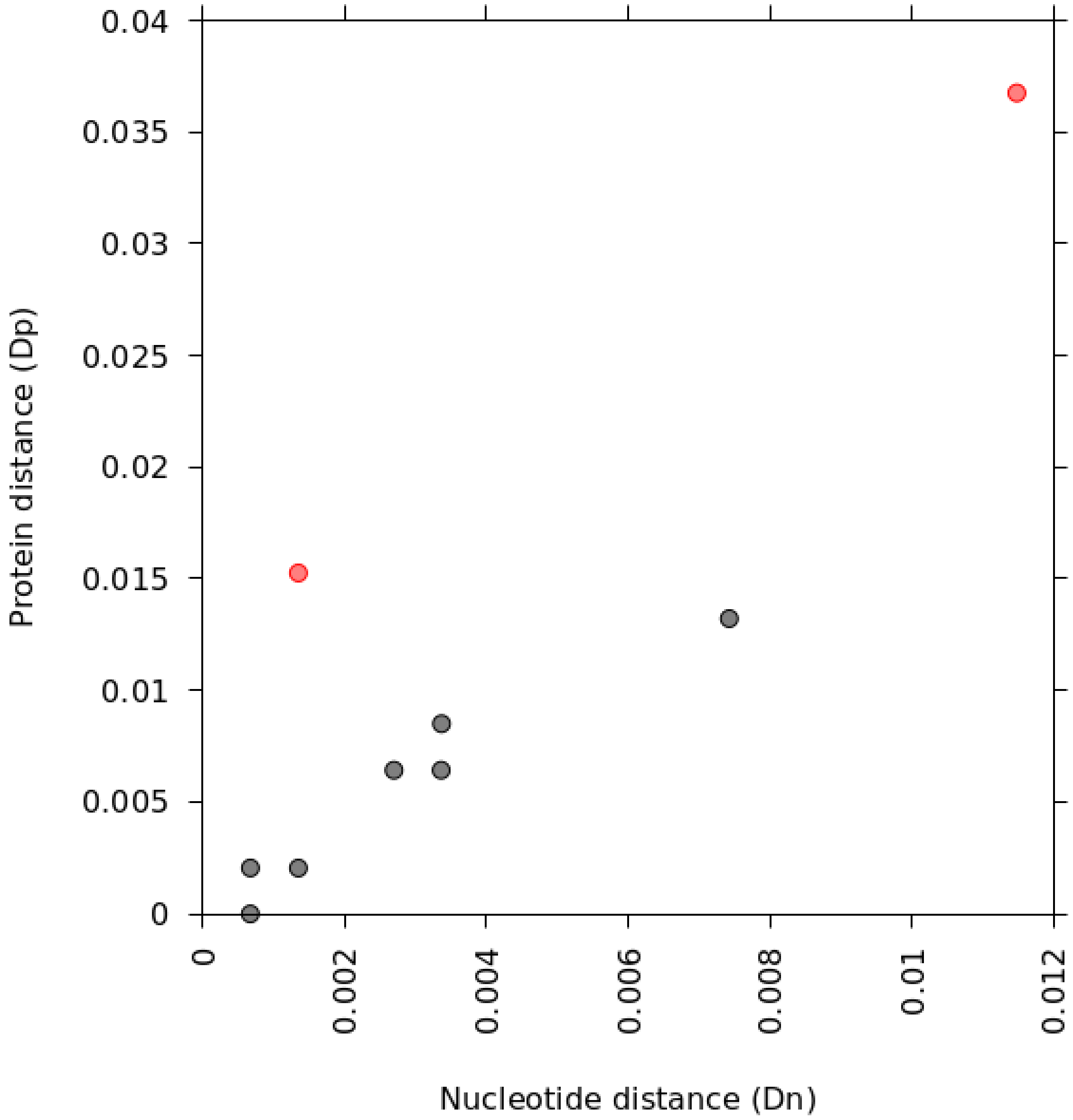

62. NC_011506.2/YP_002302226.1

**CDS cluster ID**	**62**
**CDS cluster name**	NC_011506.2/YP_002302226.1
**Total sequences**	8
**Reference forms**	6
**Compensatory frameshift forms**	2
**Virus**	Rotavirus A
**Protein**	VP2

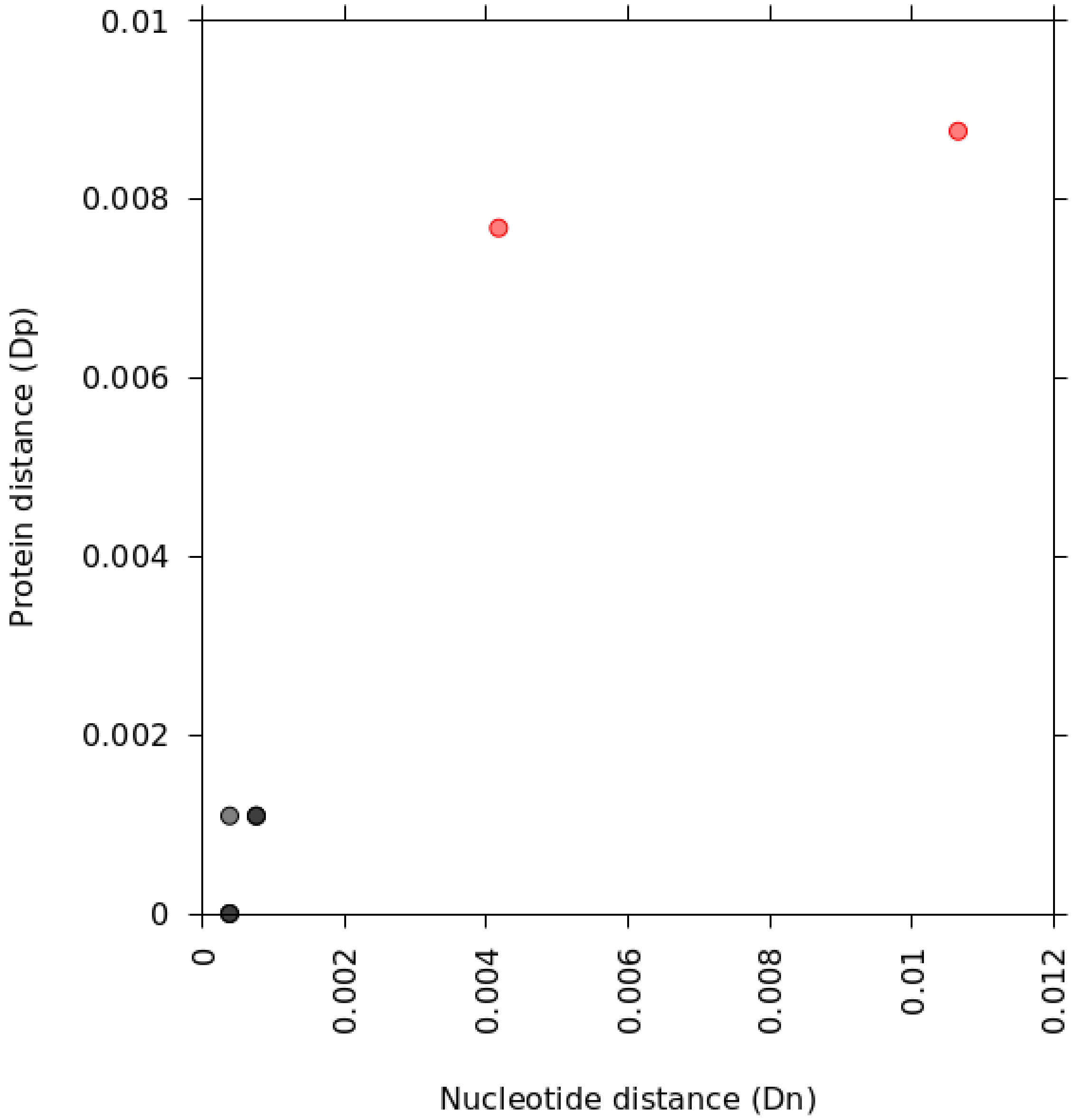

65. NC_017970.1/YP_006382460.1

**CDS cluster ID**	**65**
**CDS cluster name**	NC_017970.1/YP_006382460.1
**Total sequences**	14
**Reference forms**	12
**Compensatory frameshift forms**	2
**Virus**	Sweet potato virus 2
**Protein**	polyprotein

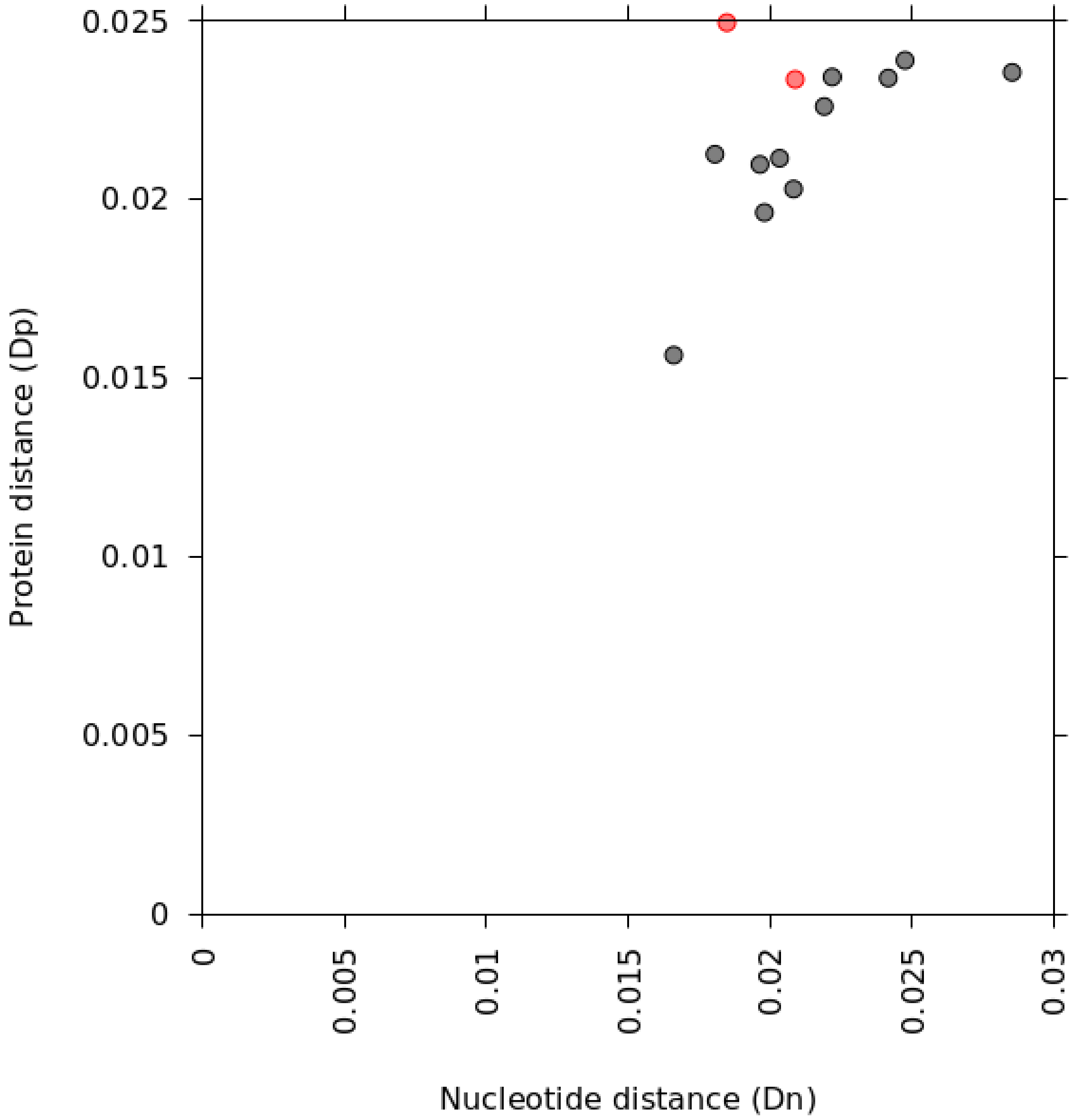

66. NC_026424.1/YP_009118473.1

**CDS cluster ID**	**66**
**CDS cluster name**	NC_026424.1/YP_009118473.1
**Total sequences**	1115
**Reference forms**	1113
**Compensatory frameshift forms**	2
**Virus**	Influenza A virus (A/Shanghai/02/2013(H7N9))
**Protein**	polymerase PA

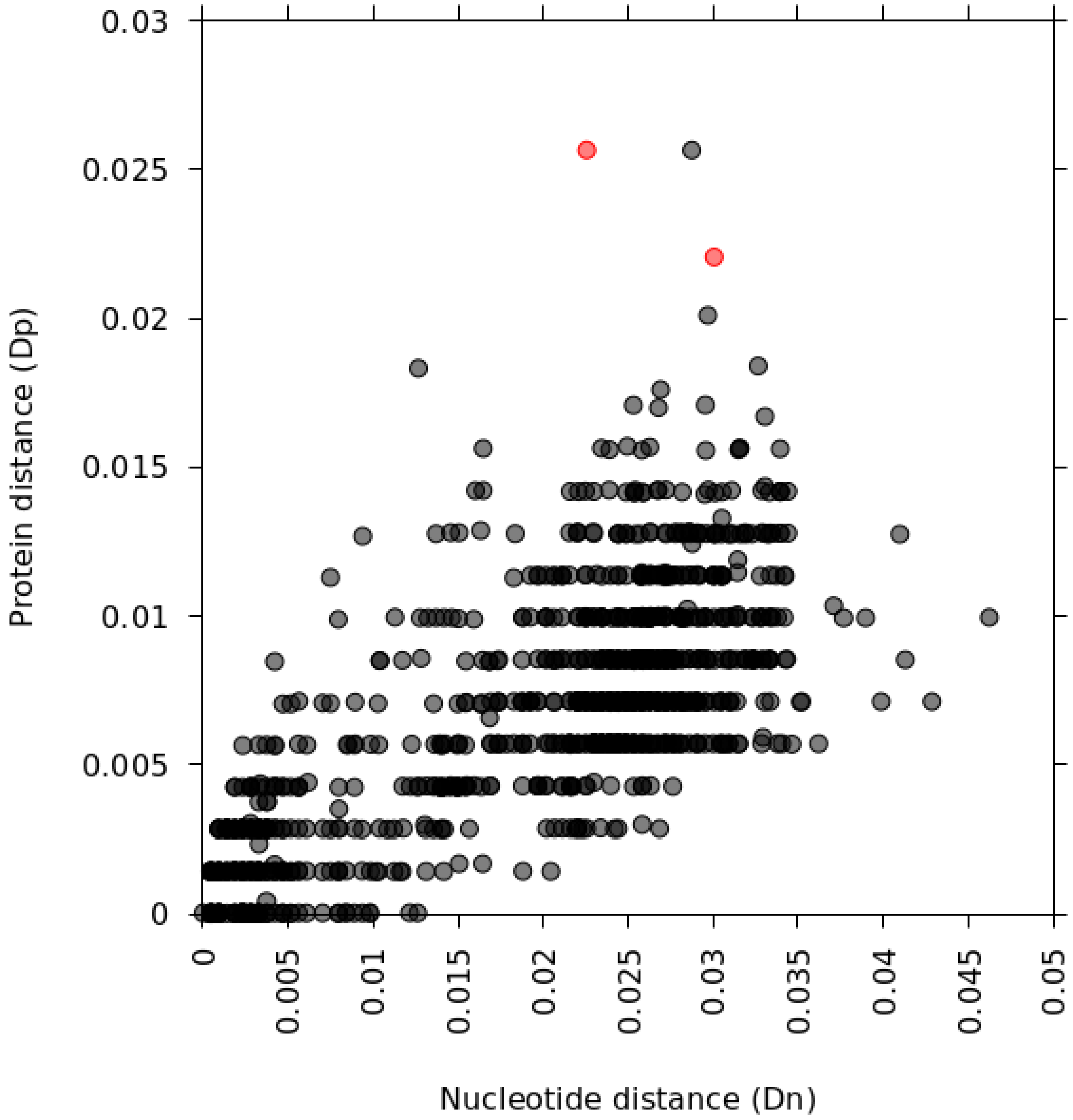

67. NC_026438.1/YP_009118631.1

**CDS cluster ID**	**67**
**CDS cluster name**	NC_026438.1/YP_009118631.1
**Total sequences**	5563
**Reference forms**	5561
**Compensatory frameshift forms**	2
**Virus**	Influenza A virus (A/California/07/2009(H1N1))
**Protein**	polymerase PB2

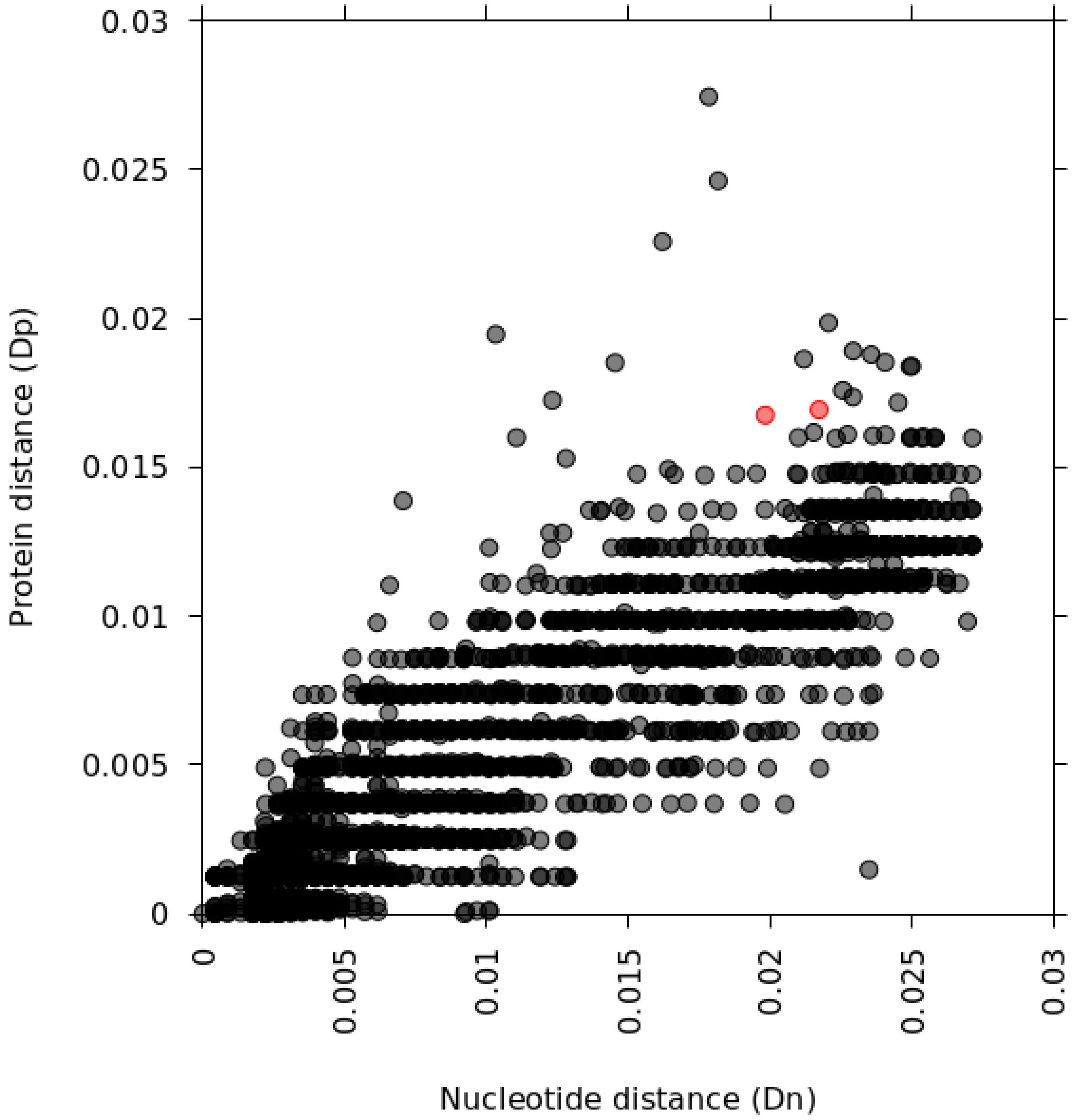

68. NC_038291.1/YP_009505553.1

**CDS cluster ID**	**68**
**CDS cluster name**	NC_038291.1/YP_009505553.1
**Total sequences**	1395
**Reference forms**	1354
**Compensatory frameshift forms**	2
**Virus**	Porcine reproductive and respiratory syndrome virus 2
**Protein**	GP5 envelope protein

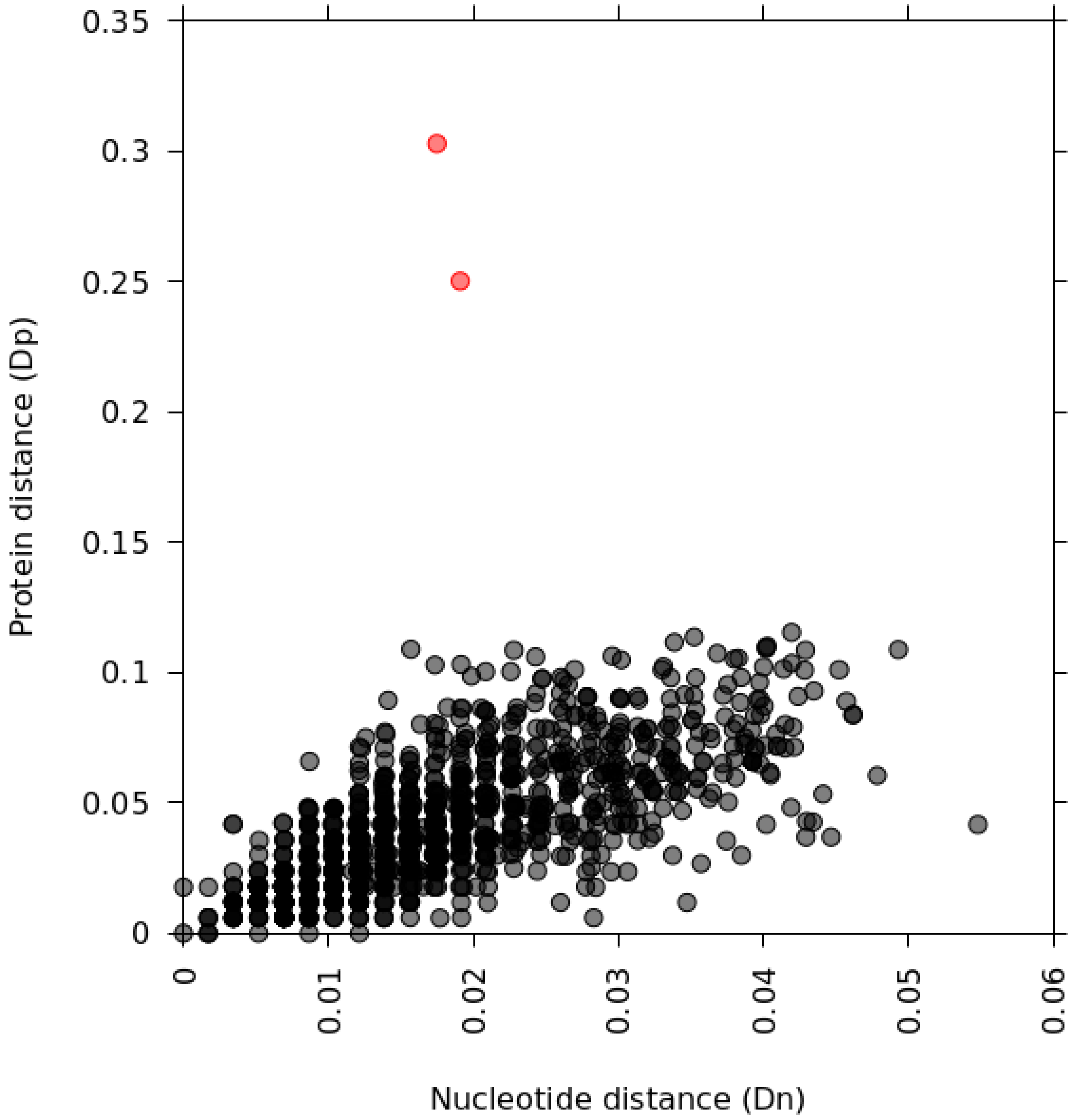

70. NC_001434.1/NP_056779.1

**CDS cluster ID**	**70**
**CDS cluster name**	NC_001434.1/NP_056779.1
**Total sequences**	7
**Reference forms**	5
**Compensatory frameshift forms**	1
**Virus**	Hepatitis E virus
**Protein**	polyprotein

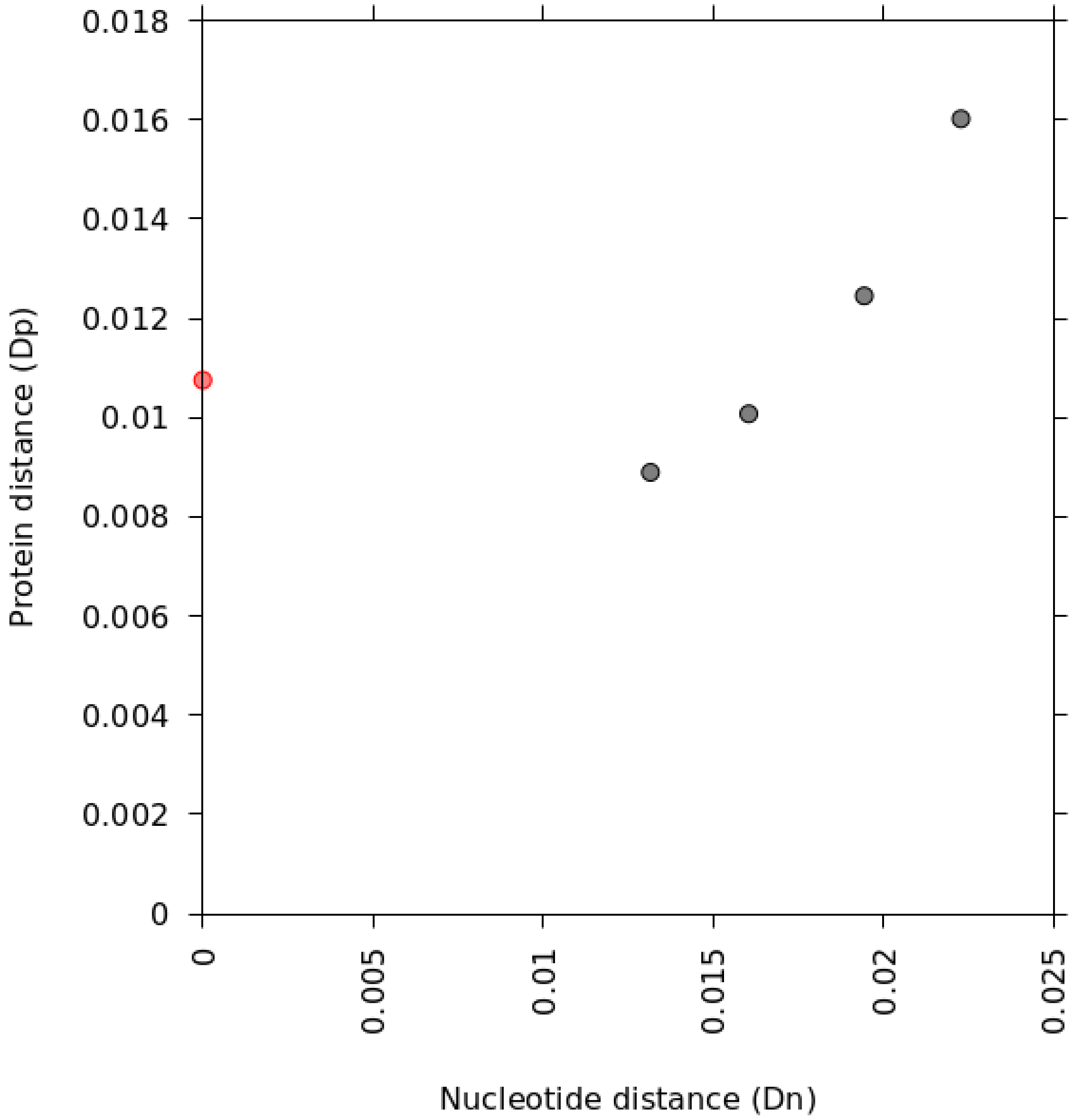

71. NC_001437.1/NP_059434.1

CDS cluster ID	**71**
CDS cluster name	NC_001437.1/NP_059434.1
Total sequences	166
Reference forms	163
Compensatory frameshift forms	1
Virus	Japanese encephalitis virus
Protein	polyprotein

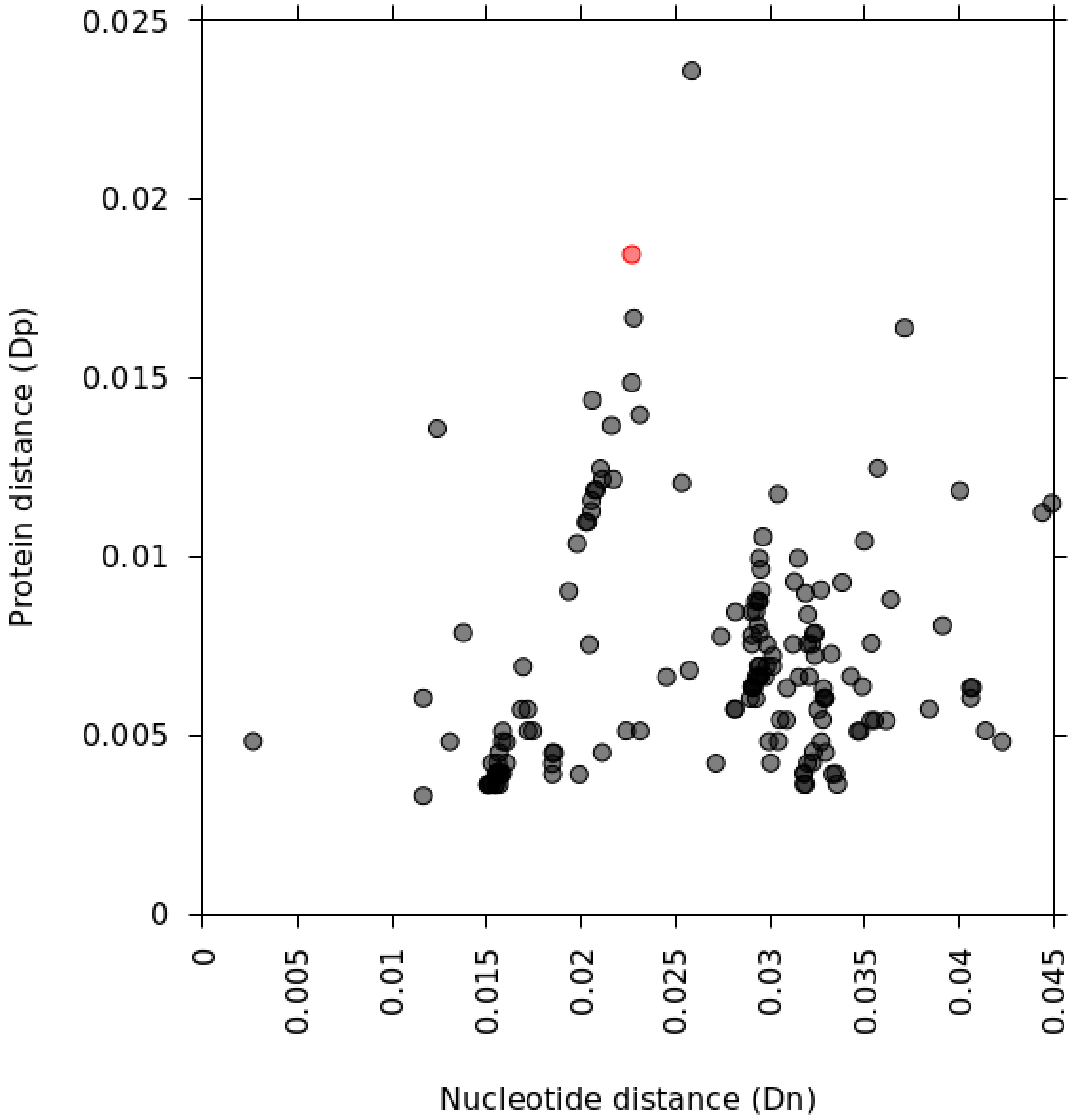

72. NC_001479.1/NP_056777.1

**CDS cluster ID**	**72**
**CDS cluster name**	NC_001479.1/NP_056777.1
**Total sequences**	27
**Reference forms**	26
**Compensatory frameshift forms**	1
**Virus**	Encephalomyocarditis virus
**Protein**	hypothetical protein

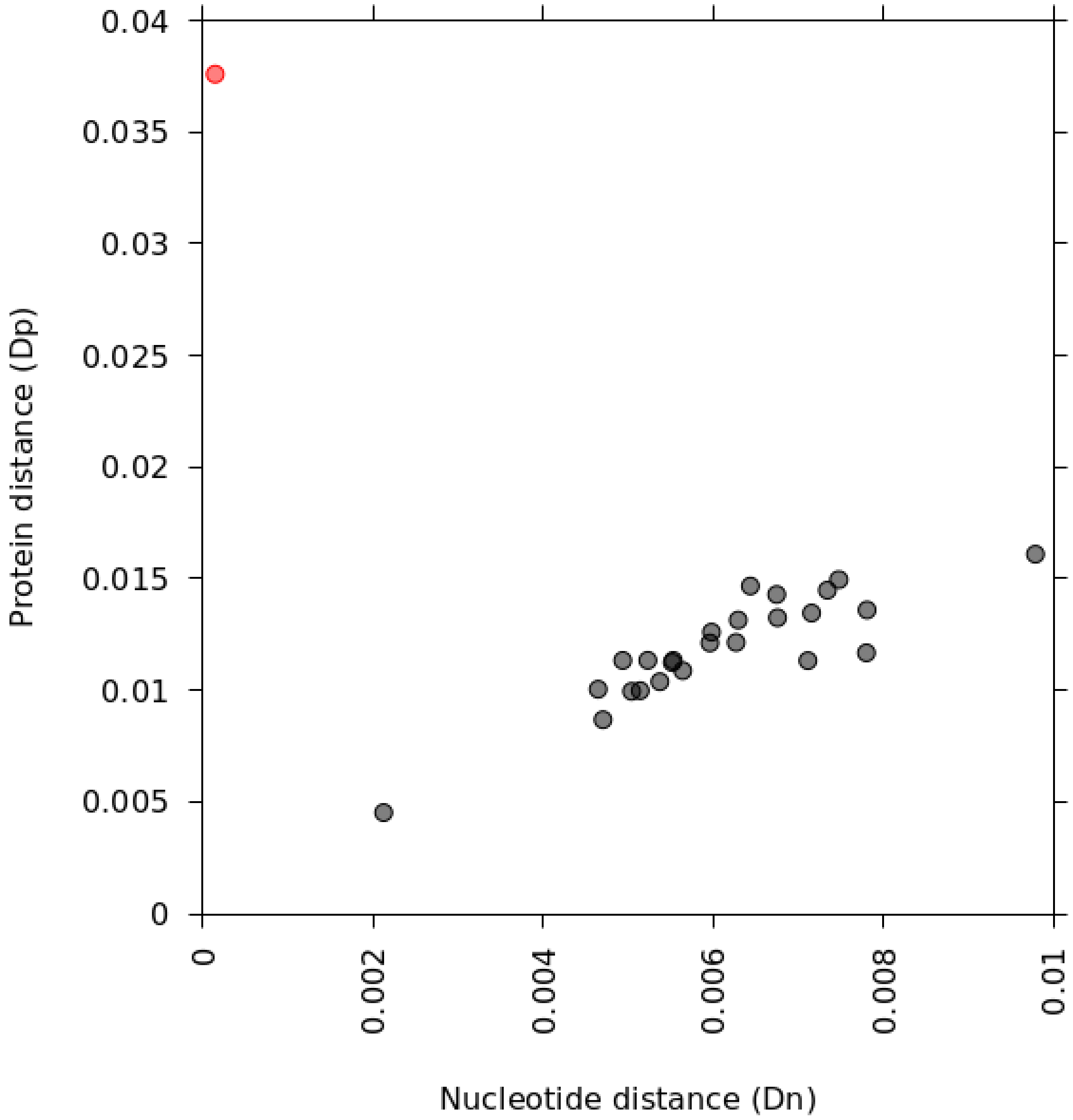

75. NC_001498.1/NP_056919.1

CDS cluster ID	75
CDS cluster name	NC_001498.1/NP_056919.1
Total sequences	238
Reference forms	235
Compensatory frameshift forms	1
Virus	Measles morbillivirus
Protein	phosphoprotein

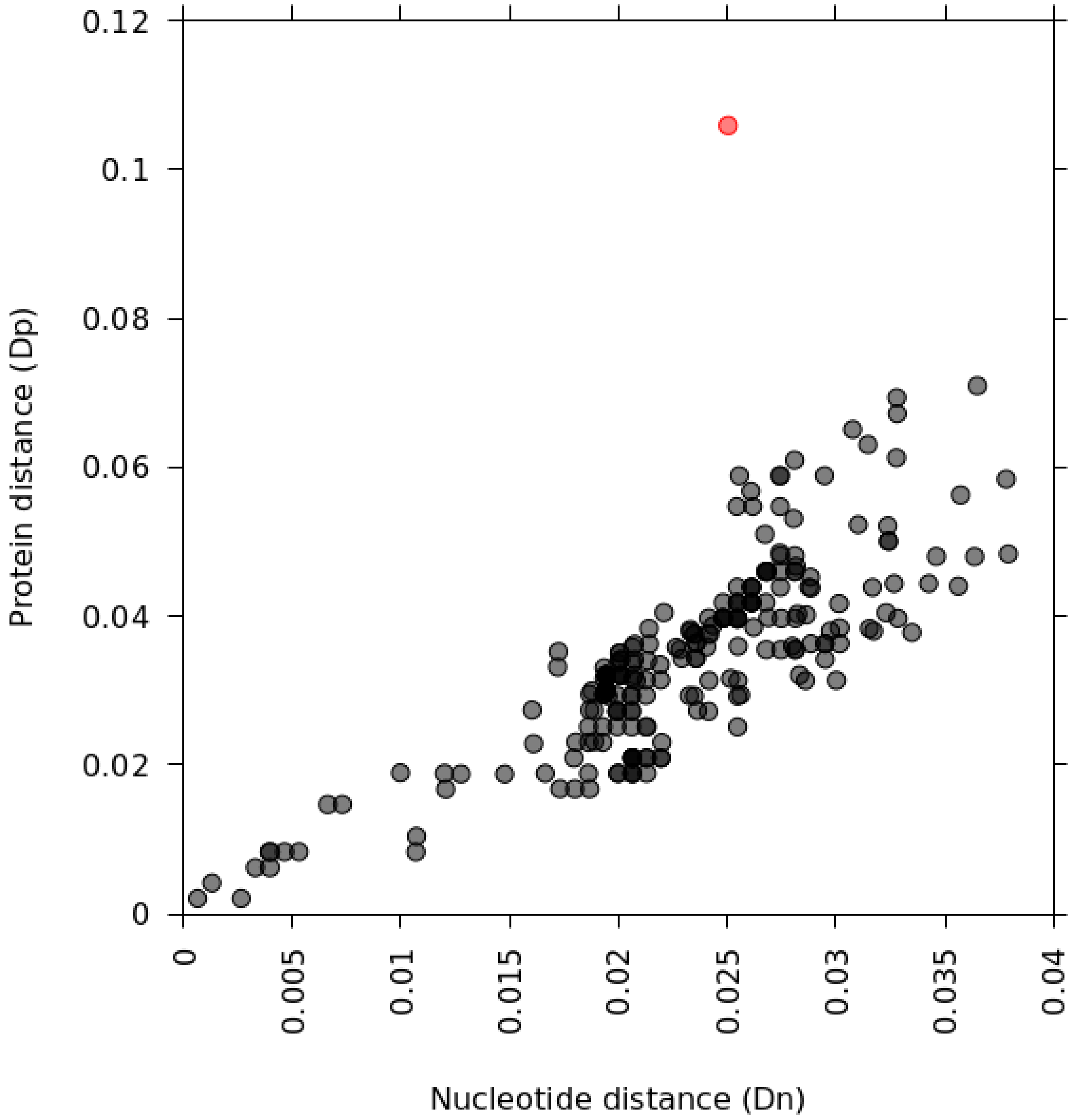

76. NC_001545.2/NP_062883.2

**CDS cluster ID**	**76**
**CDS cluster name**	NC_001545.2/NP_062883.2
**Total sequences**	30
**Reference forms**	29
**Compensatory frameshift forms**	1
**Virus**	Rubella virus
**Protein**	non-structural polyprotein

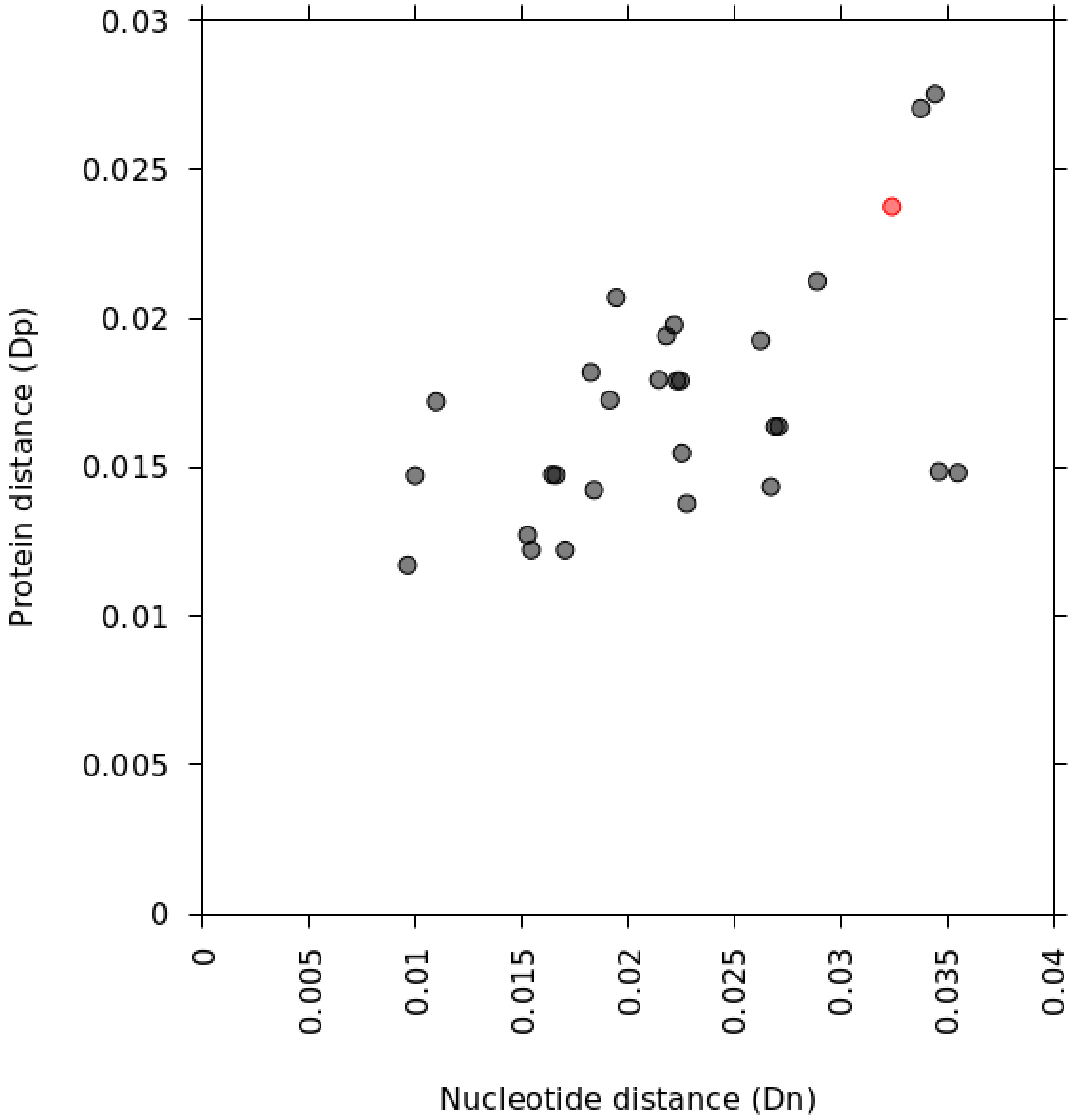

77. NC_001608.3/YP_001531159.1

**CDS cluster ID**	**77**
**CDS cluster name**	NC_001608.3/YP_001531159.1
**Total sequences**	5
**Reference forms**	4
**Compensatory frameshift forms**	1
**Virus**	Marburg marburgvirus
**Protein**	RNA-dependent RNA polymerase

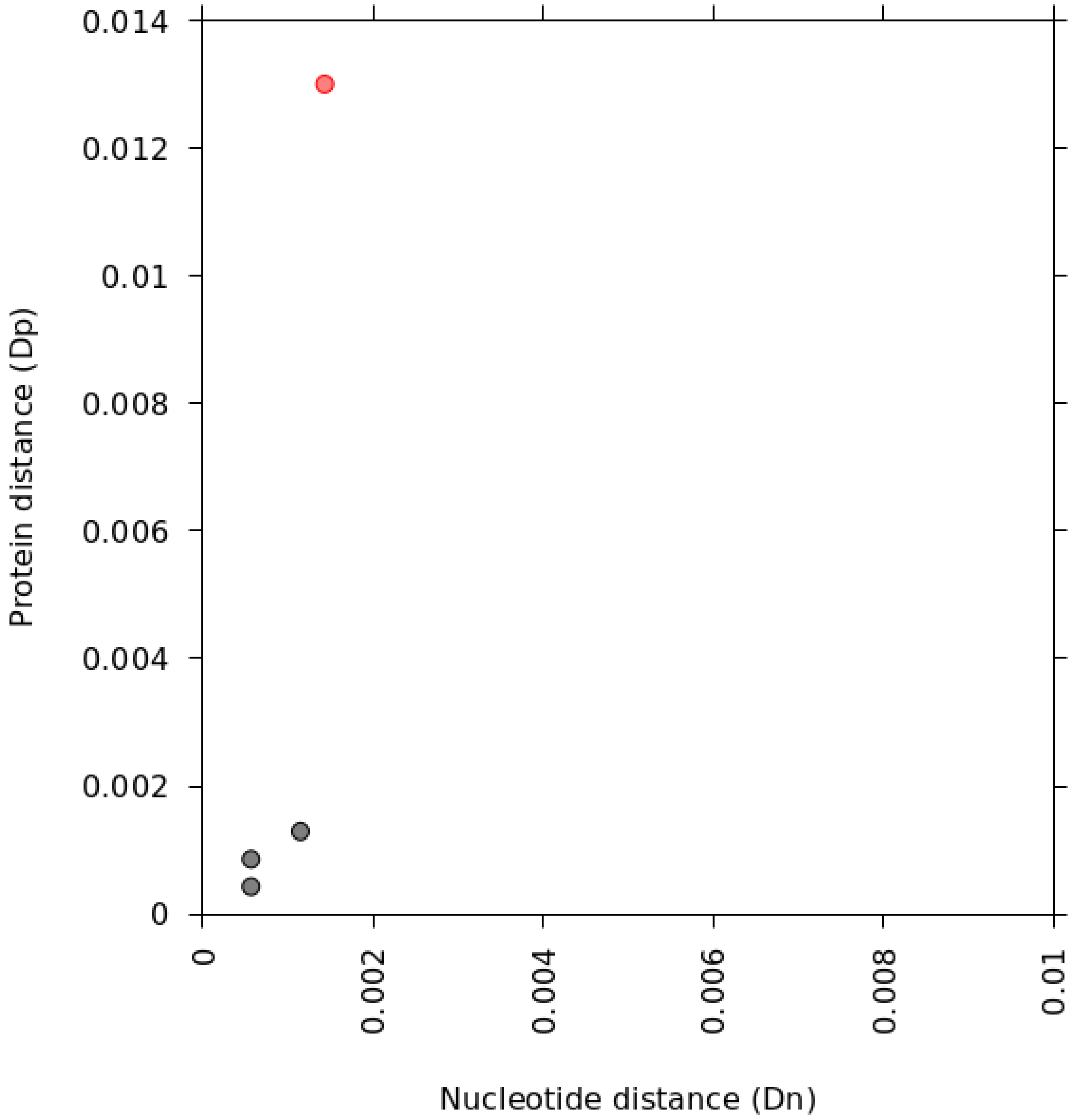

80. NC_001803.1/NP_044593.1

**CDS cluster ID**	**80**
**CDS cluster name**	NC_001803.1/NP_044593.1
**Total sequences**	495
**Reference forms**	494
**Compensatory frameshift forms**	1
**Virus**	Respiratory syncytial virus
**Protein**	Matrix protein (M)

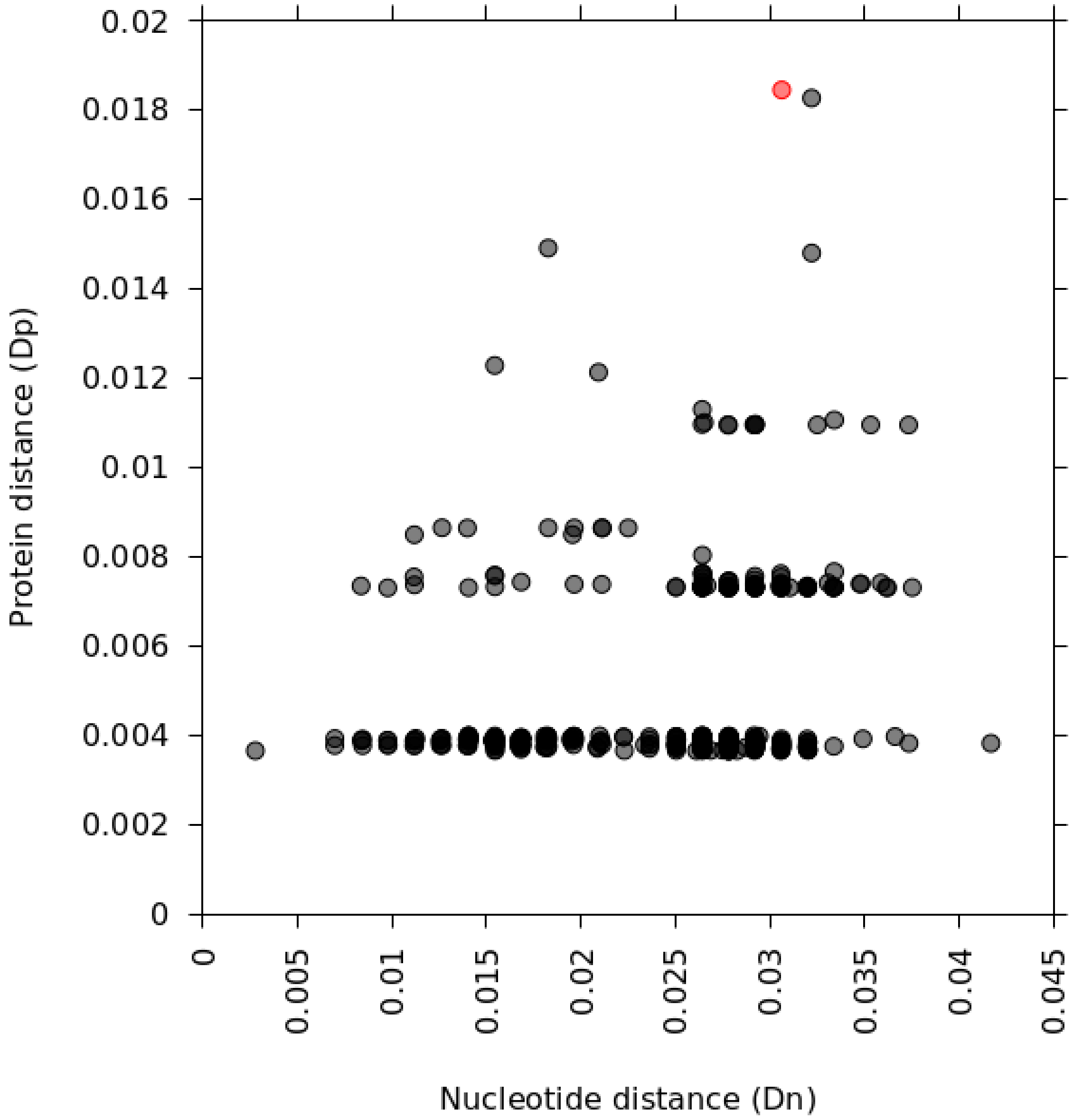

81. NC_001841.1/NP_045216.1

CDS cluster ID	81
CDS cluster name	NC_001841.1/NP_045216.1
Total sequences	28
Reference forms	25
Compensatory frameshift forms	1
Virus	Sweet potato feathery mottle virus (SPFMV)
Protein	polyprotein

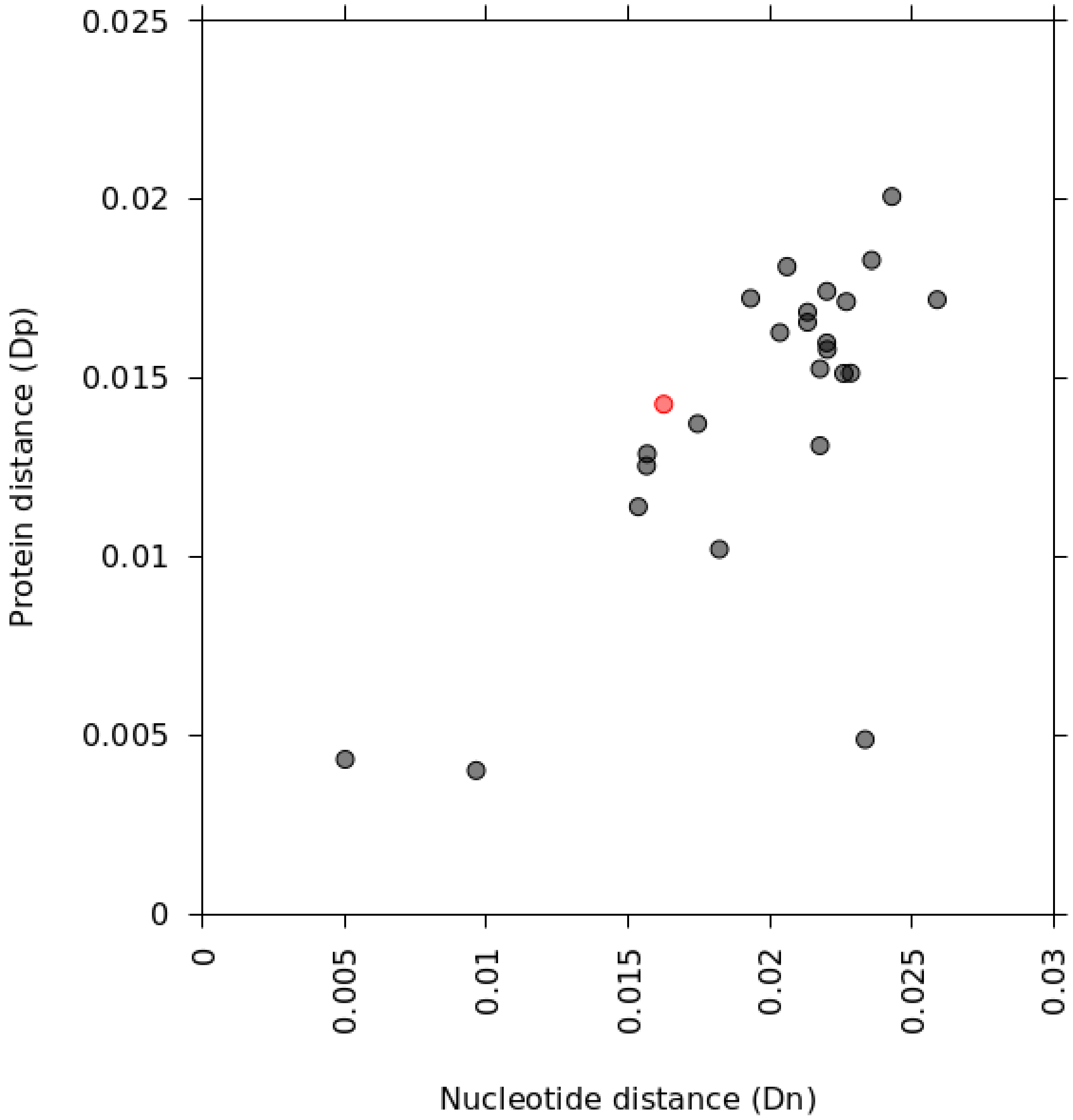

82. NC_001906.3/NP_047107.2

**CDS cluster ID**	**82**
**CDS cluster name**	NC_001906.3/NP_047107.2
**Total sequences**	13
**Reference forms**	12
**Compensatory frameshift forms**	1
**Virus**	Hendra henipavirus
**Protein**	phosphoprotein P

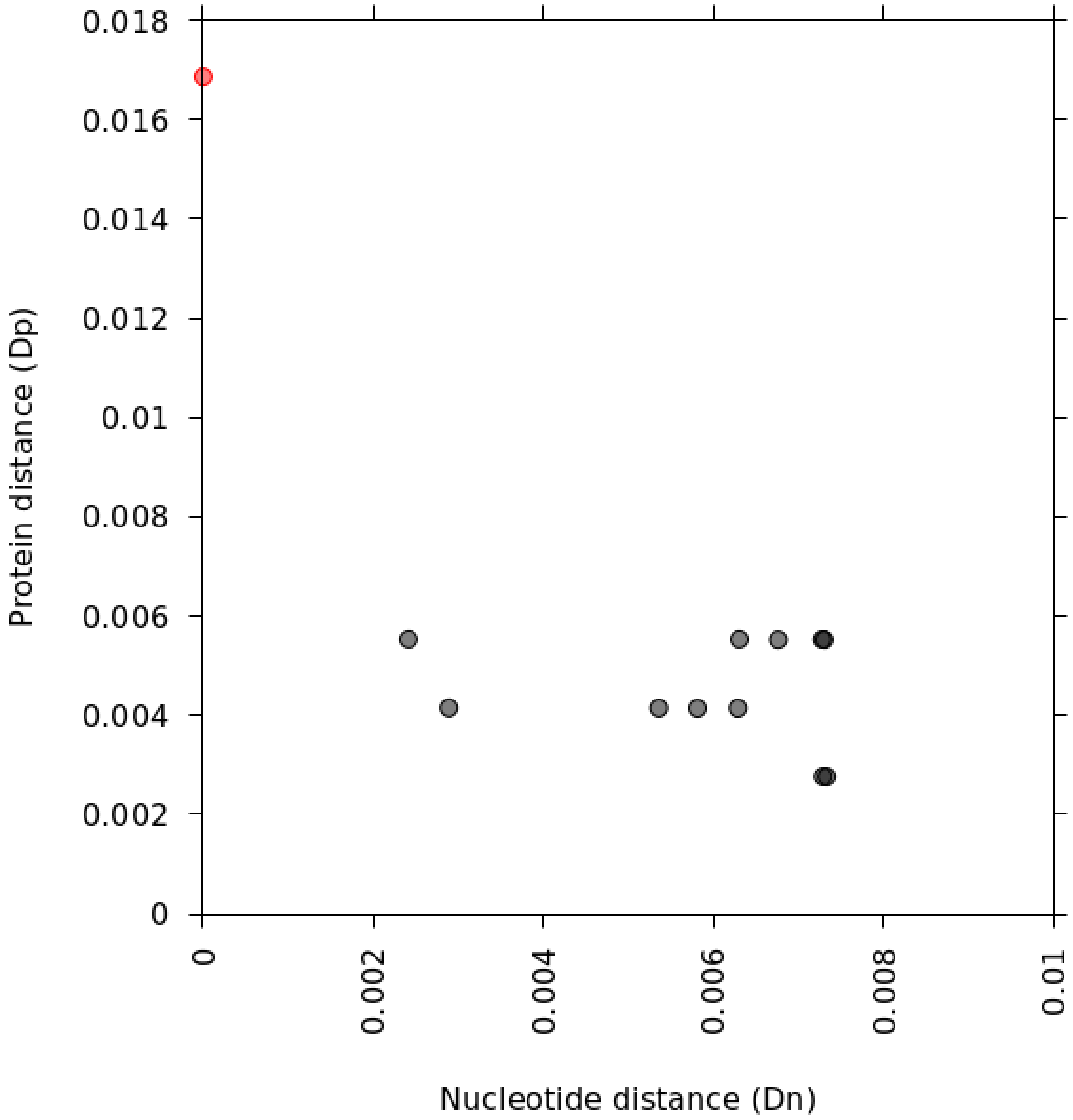

83. NC_001906.3/NP_047110.2

**CDS cluster ID**	**83**
**CDS cluster name**	NC_001906.3/NP_047110.2
**Total sequences**	13
**Reference forms**	12
**Compensatory frameshift forms**	1
**Virus**	Hendra henipavirus
**Protein**	matrix

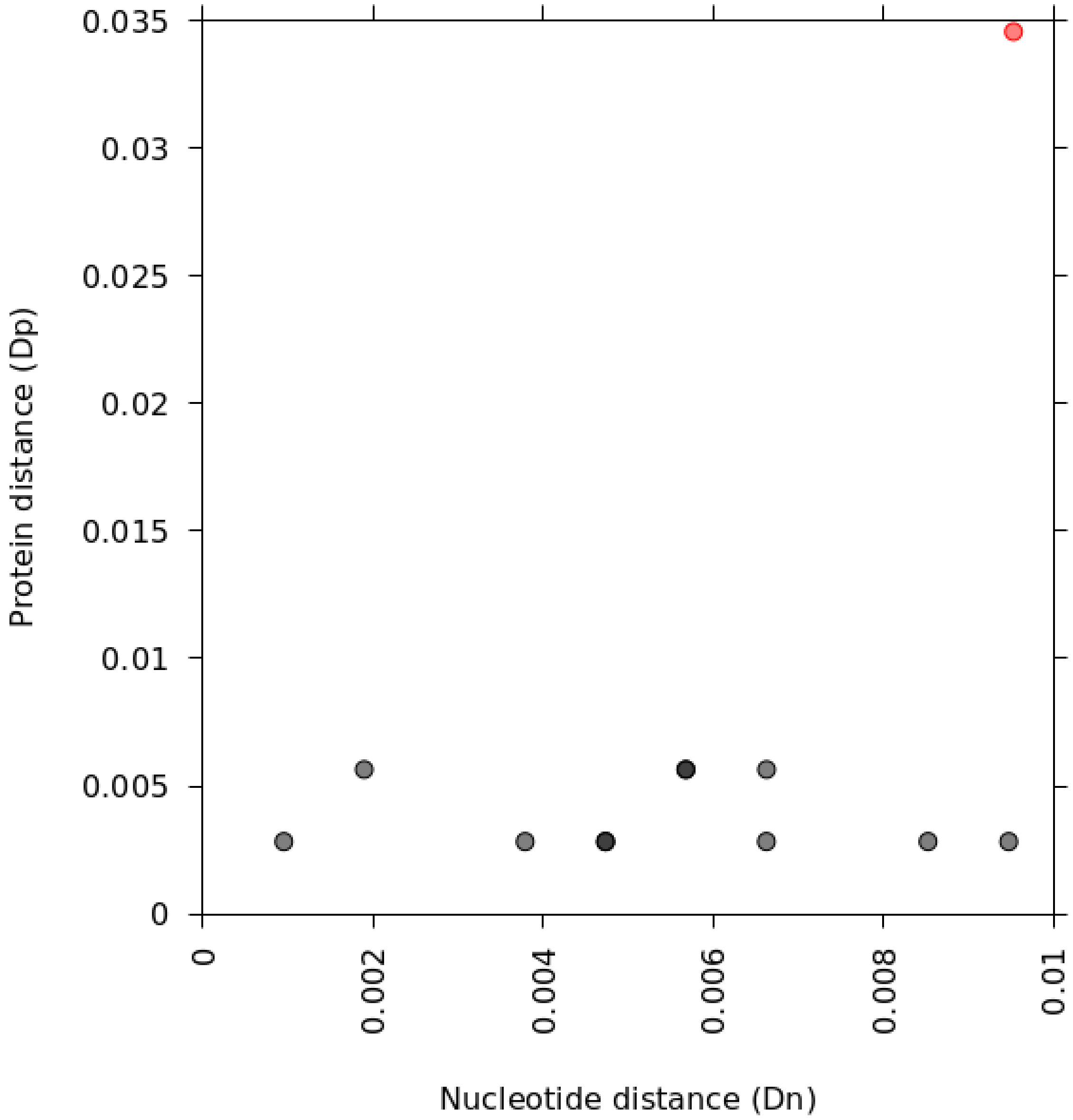

84. NC_001906.3/NP_047111.2

**CDS cluster ID**	**84**
**CDS cluster name**	NC_001906.3/NP_047111.2
**Total sequences**	13
**Reference forms**	12
**Compensatory frameshift forms**	1
**Virus**	Hendra henipavirus
**Protein**	fusion

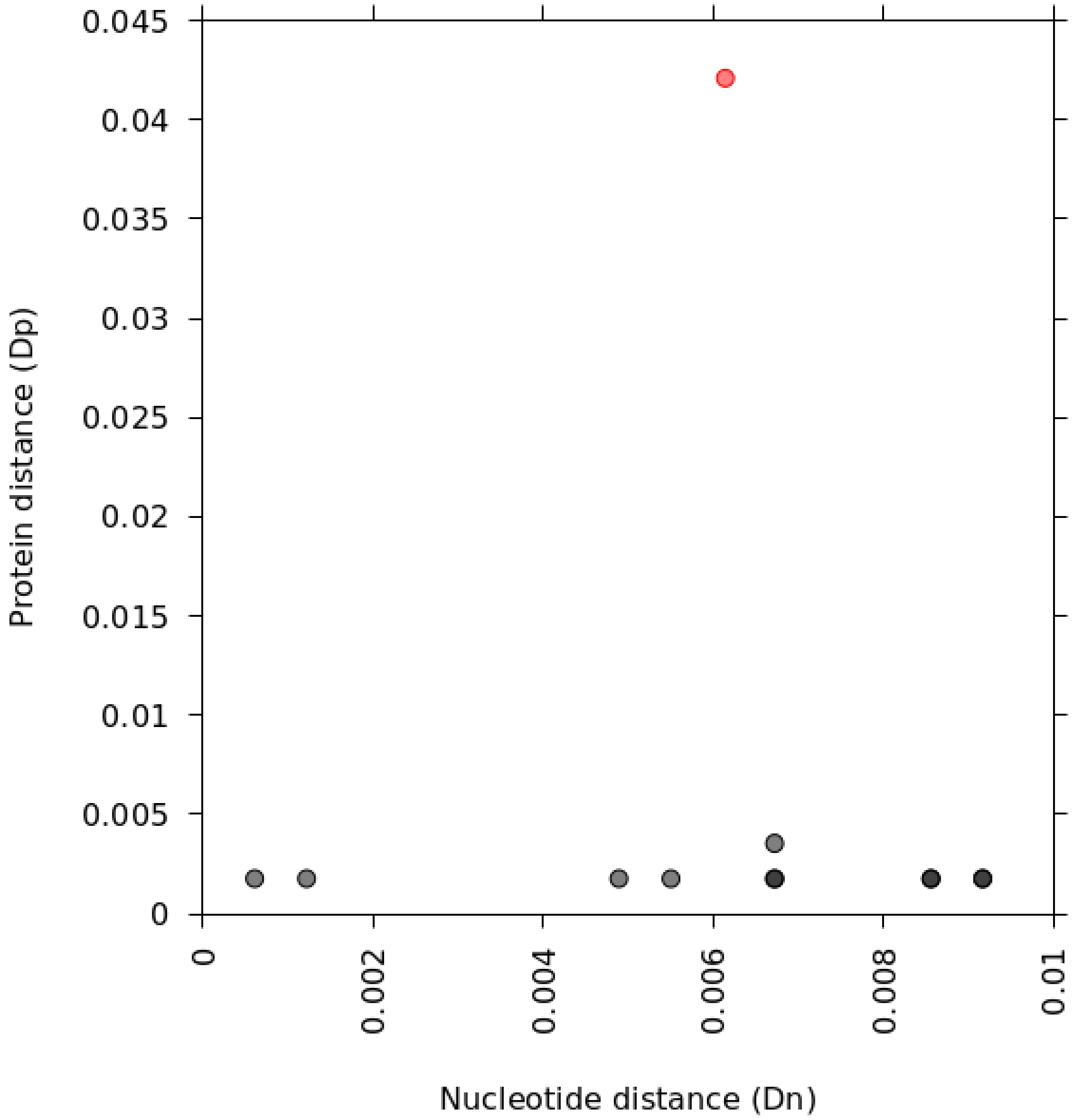

85. NC_001906.3/NP_047113.3

**CDS cluster ID**	**85**
**CDS cluster name**	NC_001906.3/NP_047113.3
**Total sequences**	12
**Reference forms**	11
**Compensatory frameshift forms**	1
**Virus**	Hendra henipavirus
**Protein**	polymerase

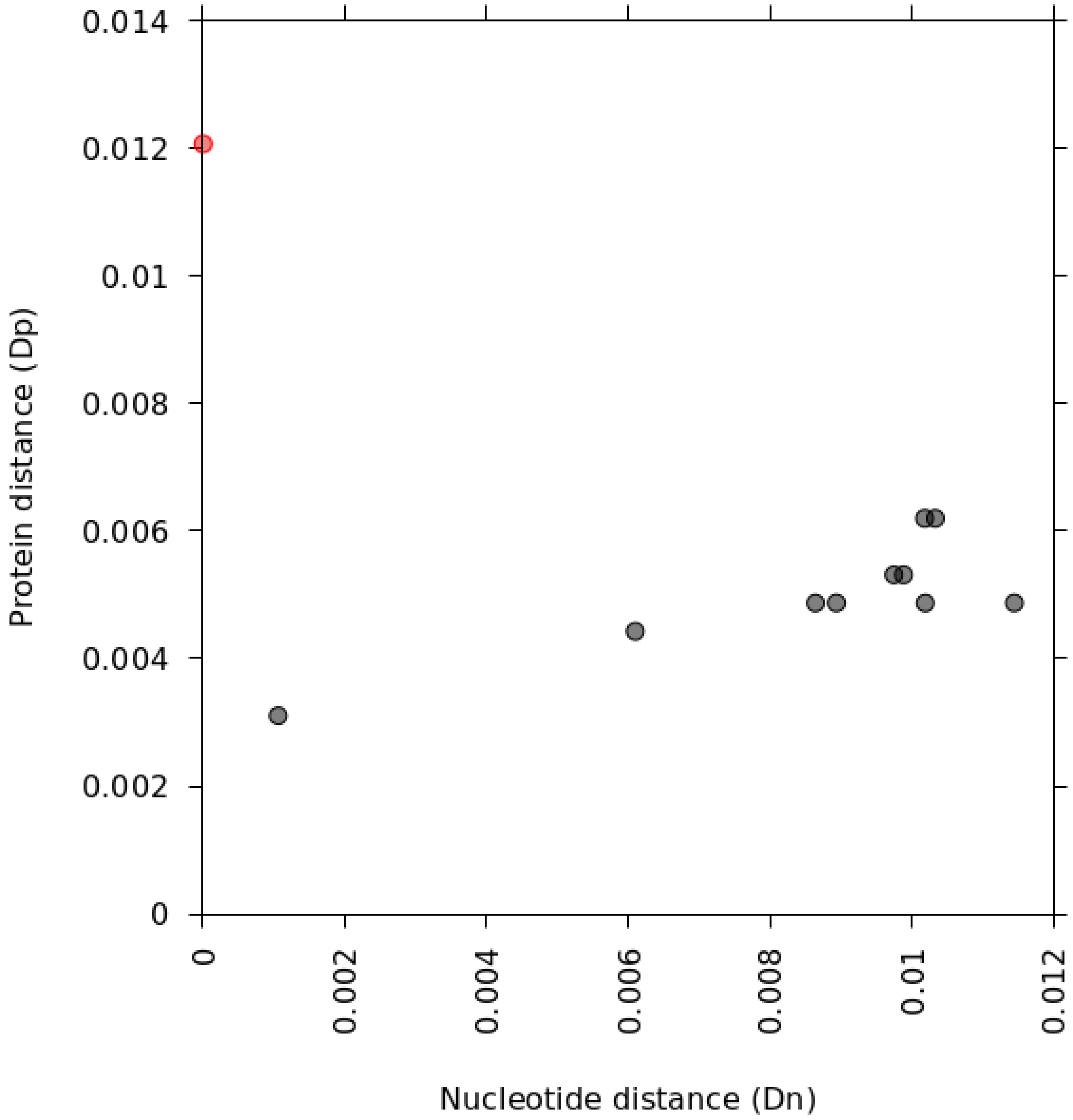

87. NC_001921.1/NP_047202.1

**CDS cluster ID**	**87**
**CDS cluster name**	NC_001921.1/NP_047202.1
**Total sequences**	12
**Reference forms**	11
**Compensatory frameshift forms**	1
**Virus**	Canine morbillivirus
**Protein**	phosphoprotein P

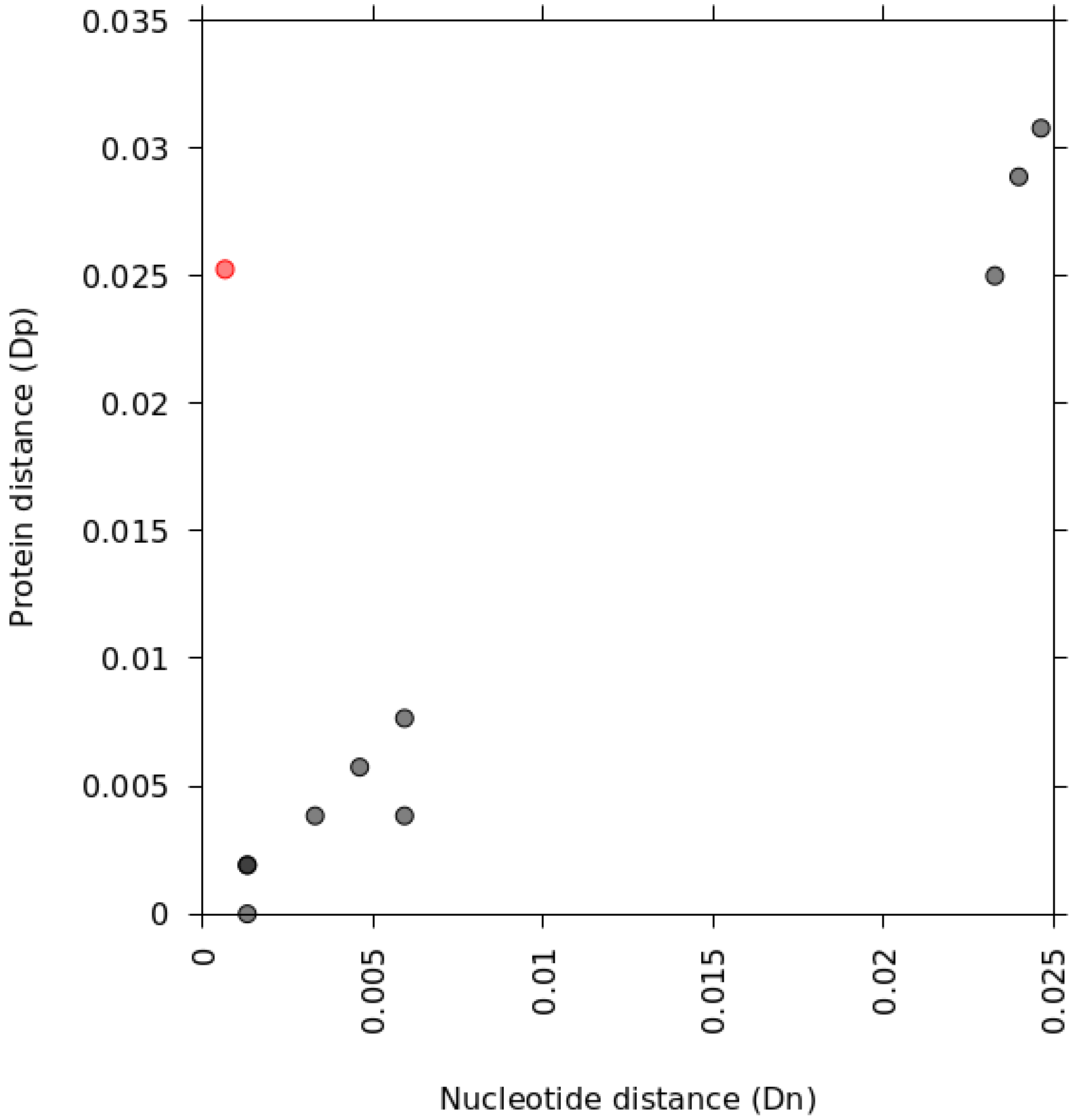

88. NC_001961.1/NP_047409.1

**CDS cluster ID**	**88**
**CDS cluster name**	NC_001961.1/NP_047409.1
**Total sequences**	69
**Reference forms**	68
**Compensatory frameshift forms**	1
**Virus**	Porcine reproductive and respiratory syndrome virus
**Protein**	GP3 envelope protein

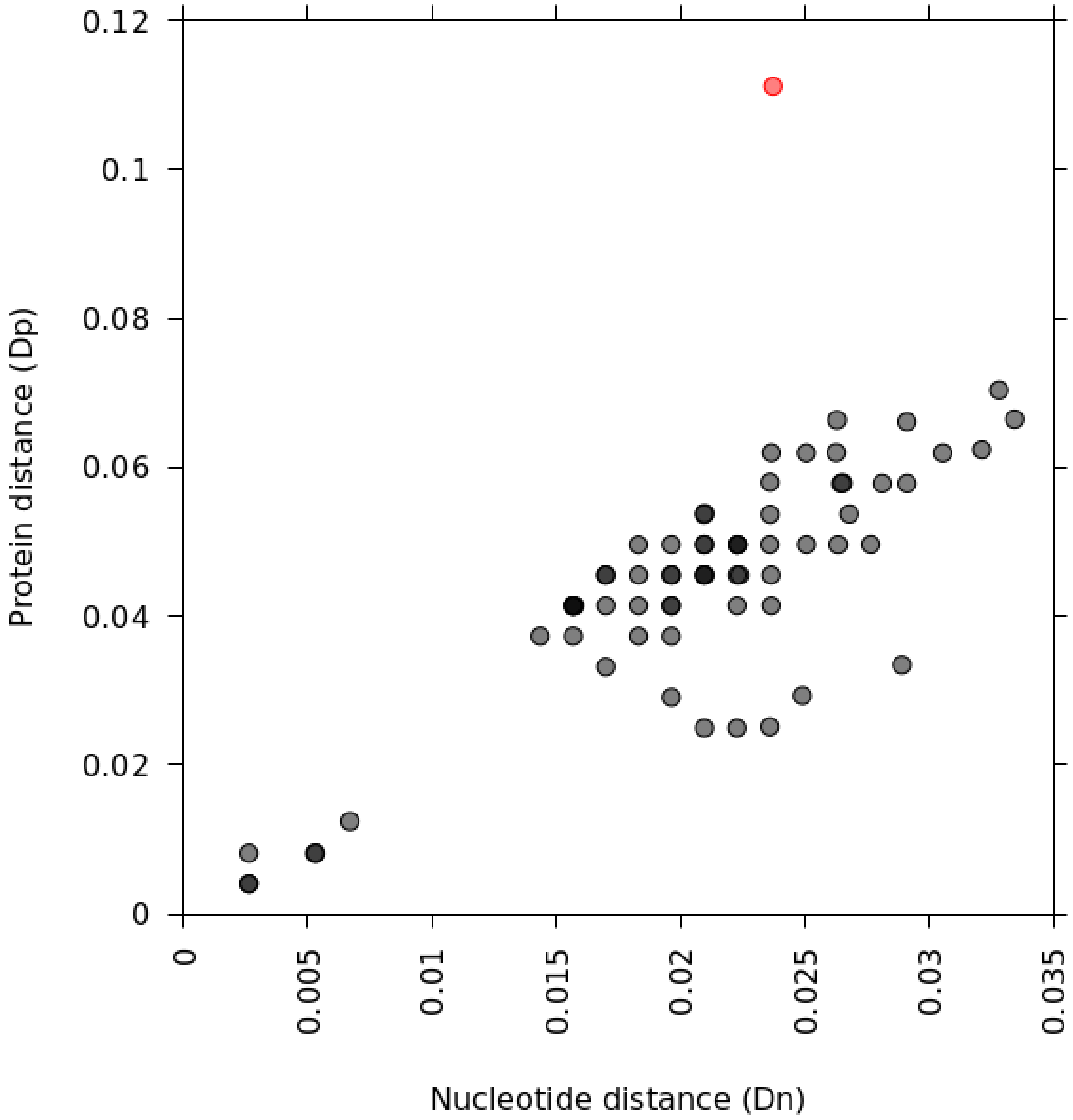

89. NC_001961.1/NP_066135.1

**CDS cluster ID**	**89**
**CDS cluster name**	NC_001961.1/NP_066135.1
**Total sequences**	65
**Reference forms**	64
**Compensatory frameshift forms**	1
**Virus**	Porcine reproductive and respiratory syndrome virus
**Protein**	ORF 1ab polyprotein

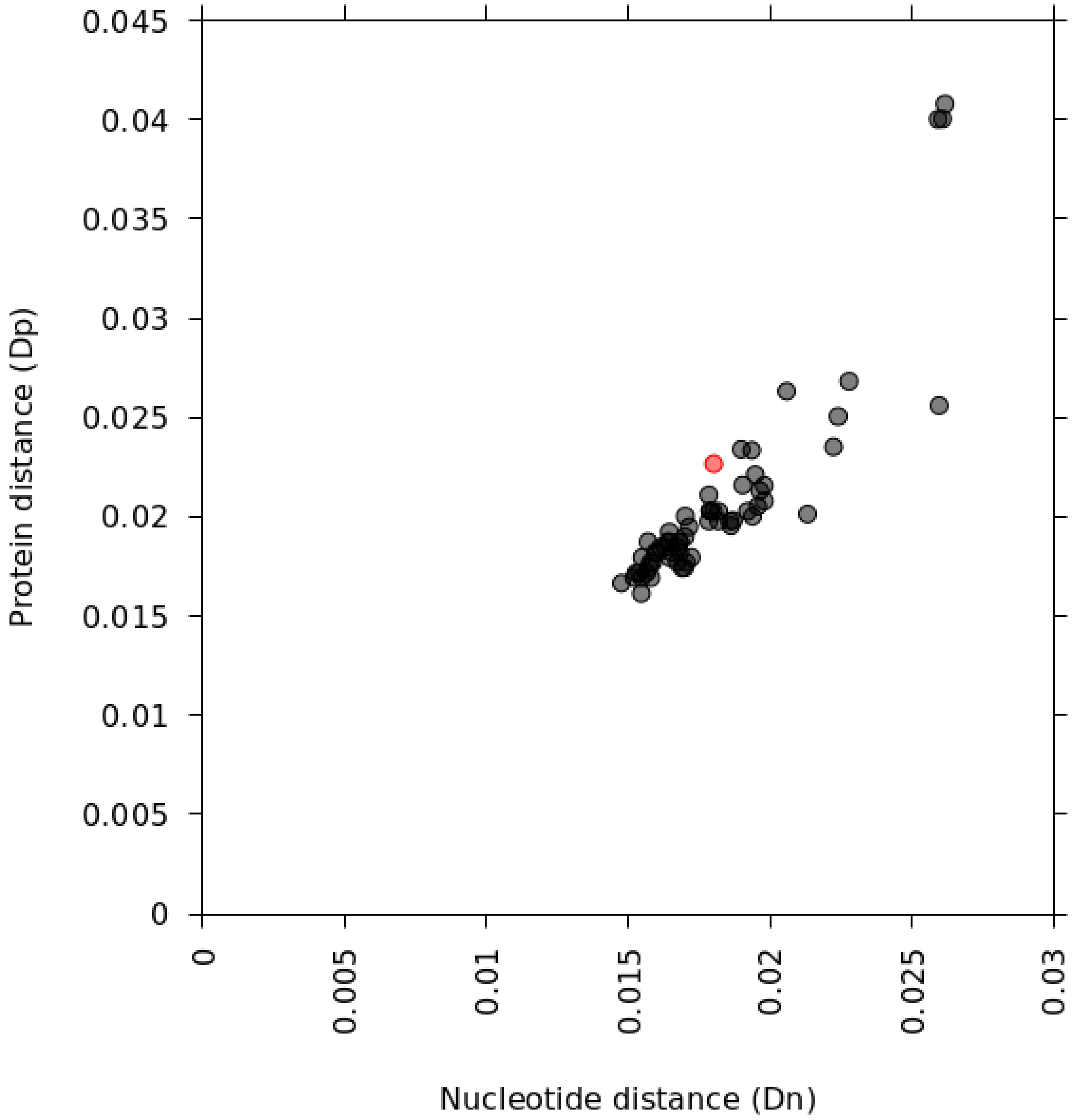

91. NC_002058.3/NP_041277.1

**CDS cluster ID**	**91**
**CDS cluster name**	NC_002058.3/NP_041277.1
**Total sequences**	110
**Reference forms**	109
**Compensatory frameshift forms**	1
**Virus**	Enterovirus C
**Protein**	genome polyprotein

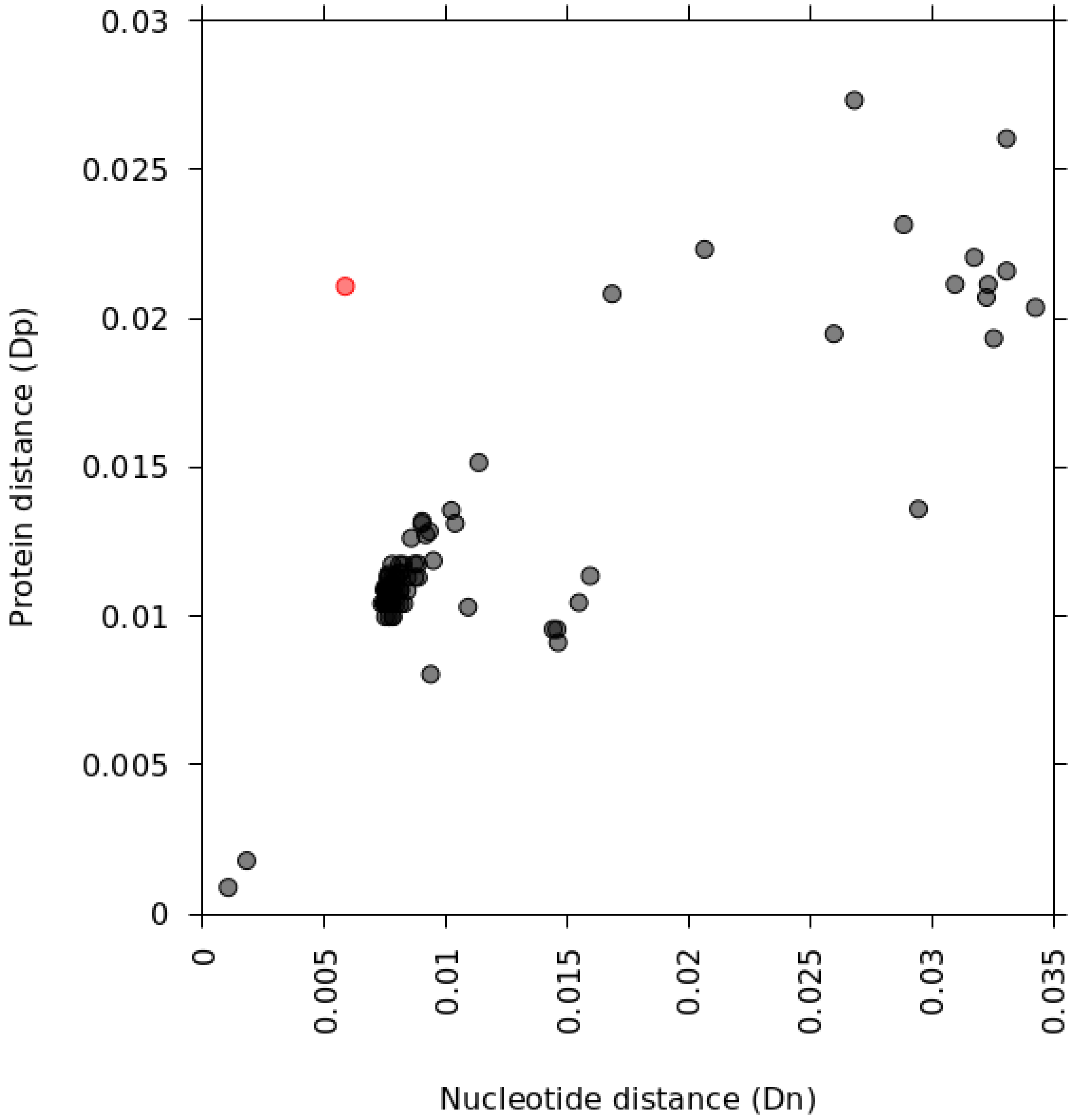

92. NC_002306.3/YP_004070193.2

**CDS cluster ID**	**92**
**CDS cluster name**	NC_002306.3/YP_004070193.2
**Total sequences**	8
**Reference forms**	7
**Compensatory frameshift forms**	1
**Virus**	Feline infectious peritonitis virus
**Protein**	polyprotein ab

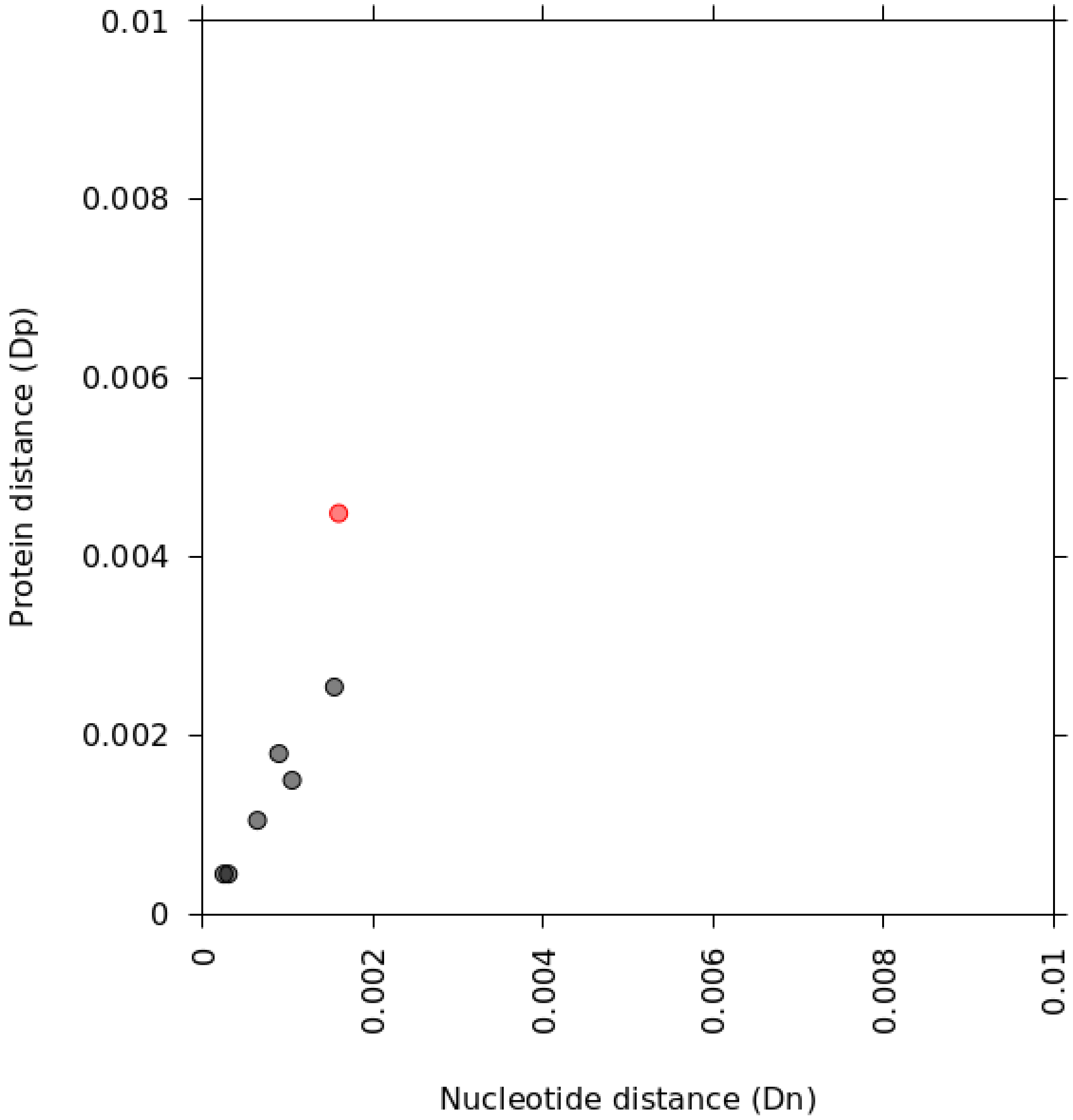

94. NC_002634.1/NP_072165.1

**CDS cluster ID**	**94**
**CDS cluster name**	NC_002634.1/NP_072165.1
**Total sequences**	33
**Reference forms**	32
**Compensatory frameshift forms**	1
**Virus**	Soybean mosaic virus
**Protein**	polyprotein precursor

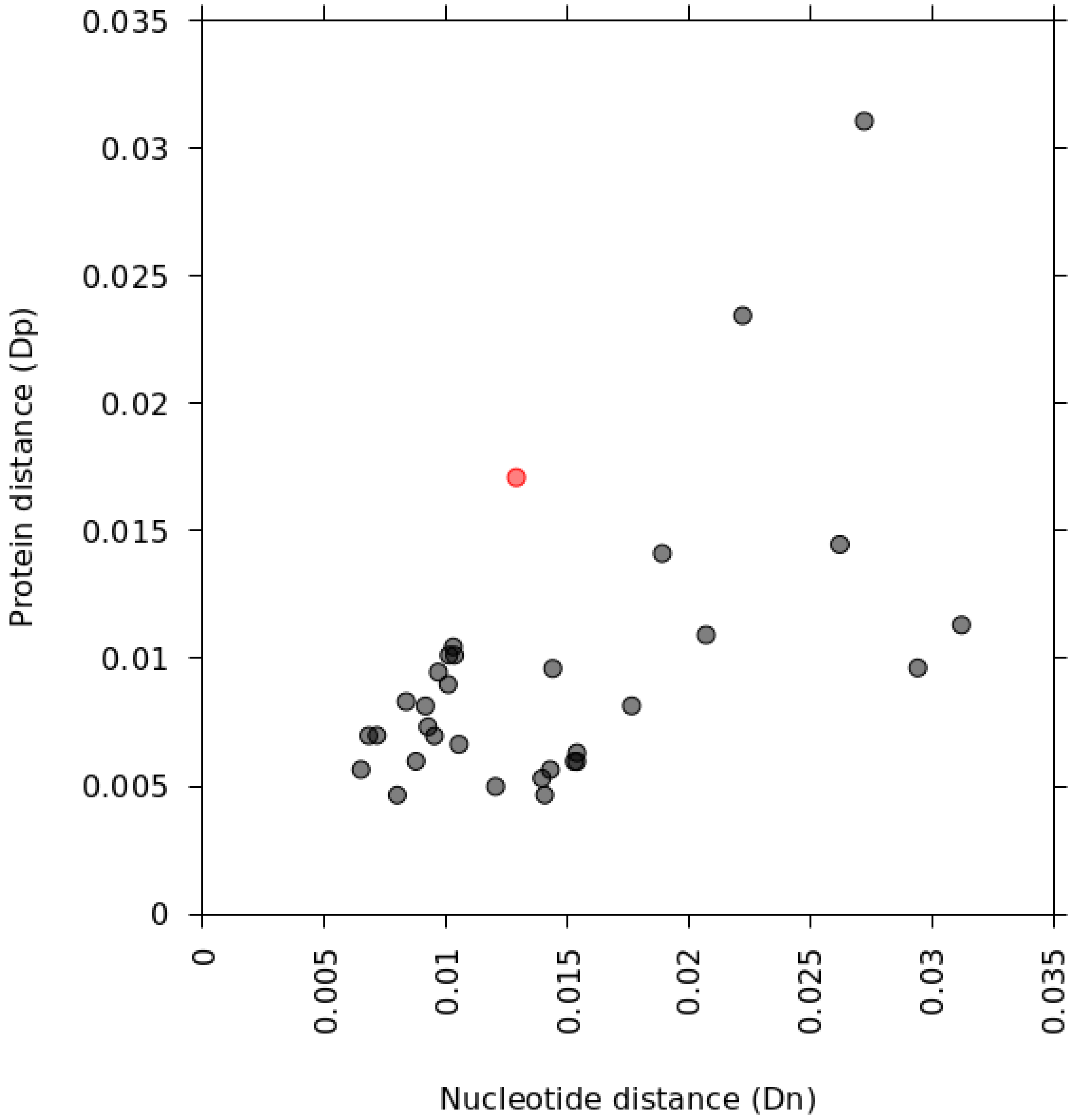

99. NC_003045.1/NP_150077.1

**CDS cluster ID**	**99**
**CDS cluster name**	NC_003045.1/NP_150077.1
**Total sequences**	219
**Reference forms**	218
Compensatory frameshift forms	1
**Virus**	Bovine coronavirus
**Protein**	spike structural protein

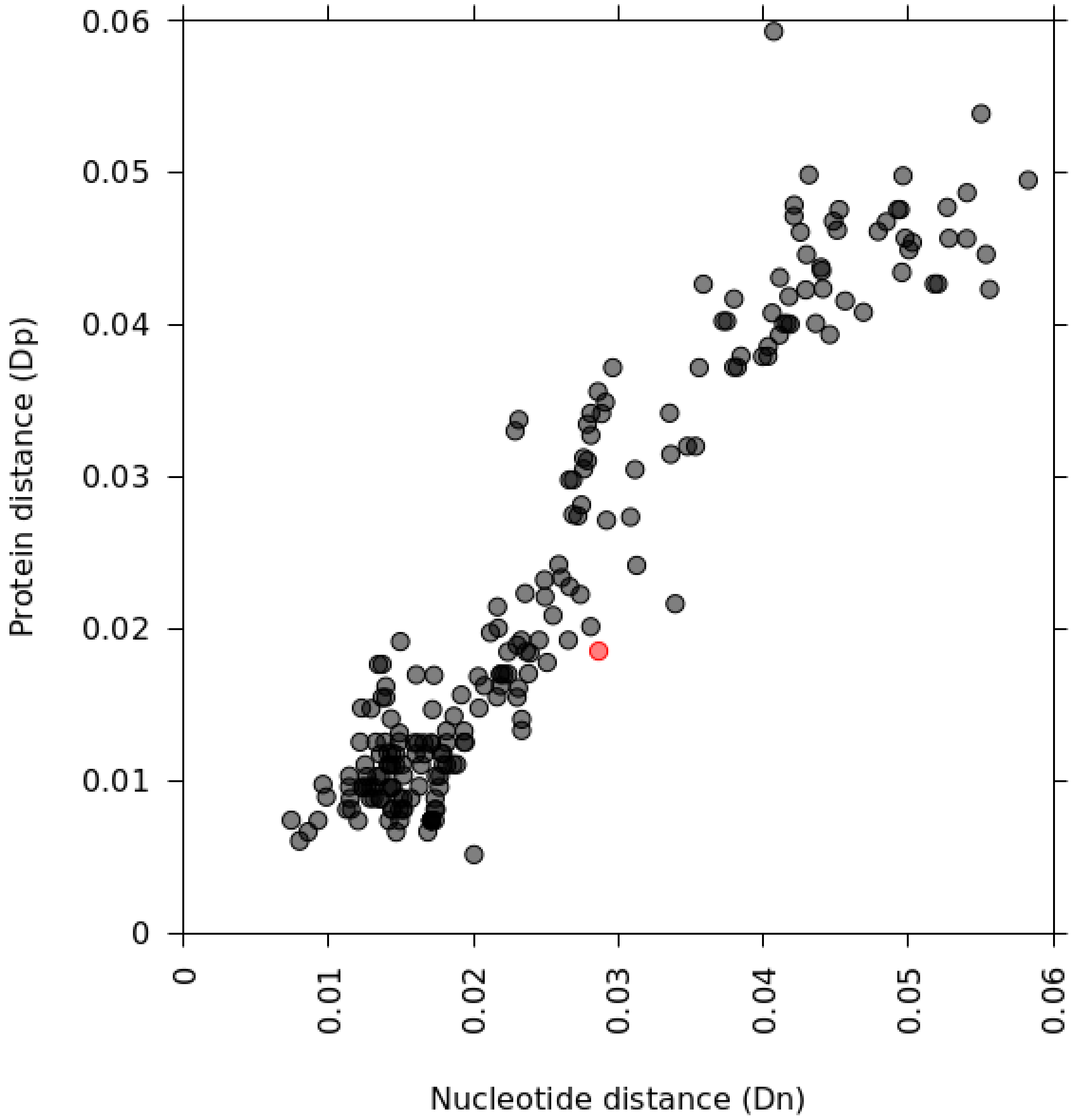

100. NC_003436.1/NP_598313.1

**CDS cluster ID**	**100**
**CDS cluster name**	NC_003436.1/NP_598313.1
**Total sequences**	415
**Reference forms**	414
**Compensatory frameshift forms**	1
**Virus**	Porcine epidemic diarrhea virus (PEDV)
**Protein**	membrane protein

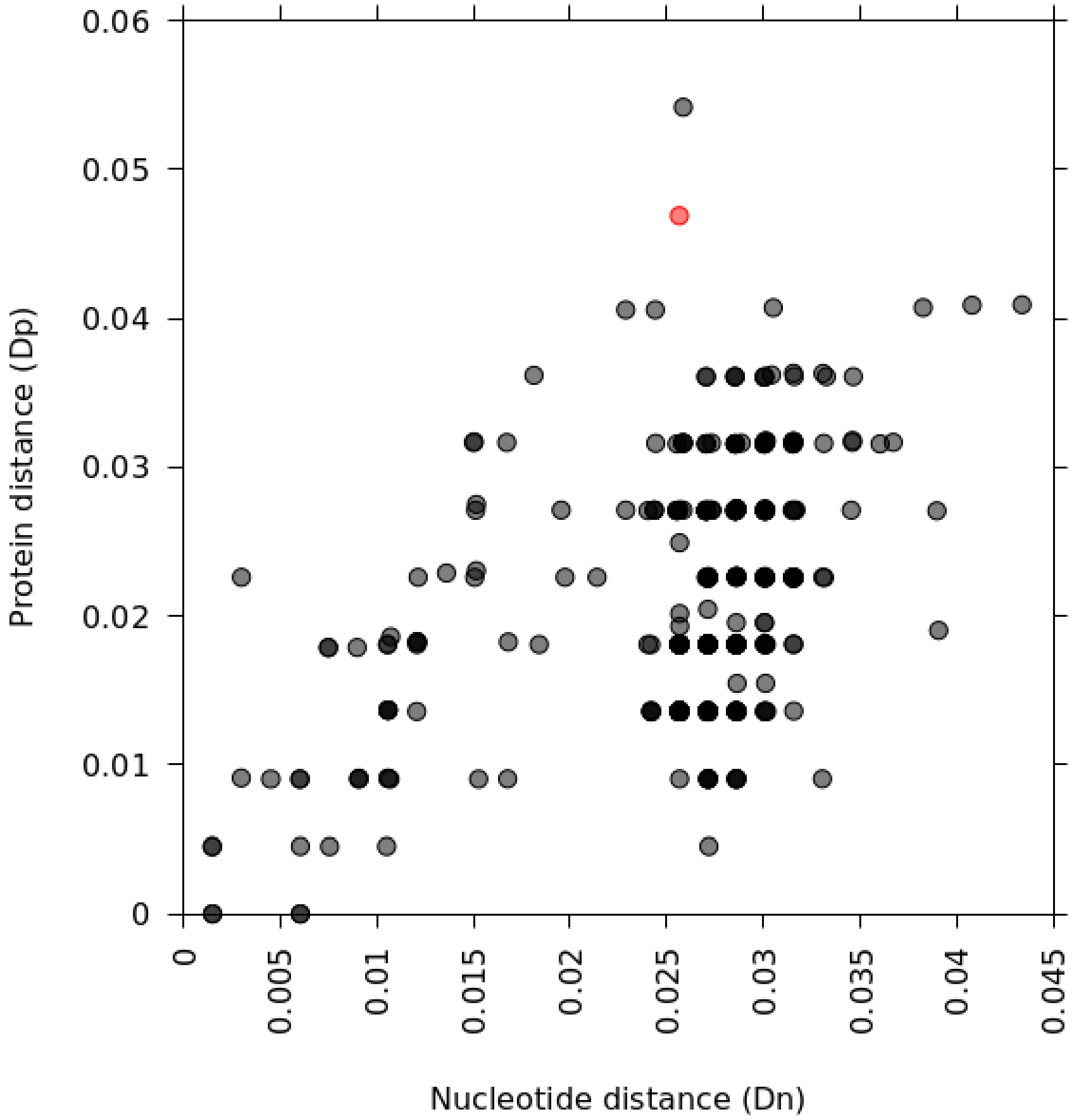

101. NC_003443.1/NP_598403.1

**CDS cluster ID**	**101**
**CDS cluster name**	NC_003443.1/NP_598403.1
**Total sequences**	5
**Reference forms**	4
**Compensatory frameshift forms**	1
**Virus**	Human rubulavirus 2
**Protein**	matrix protein

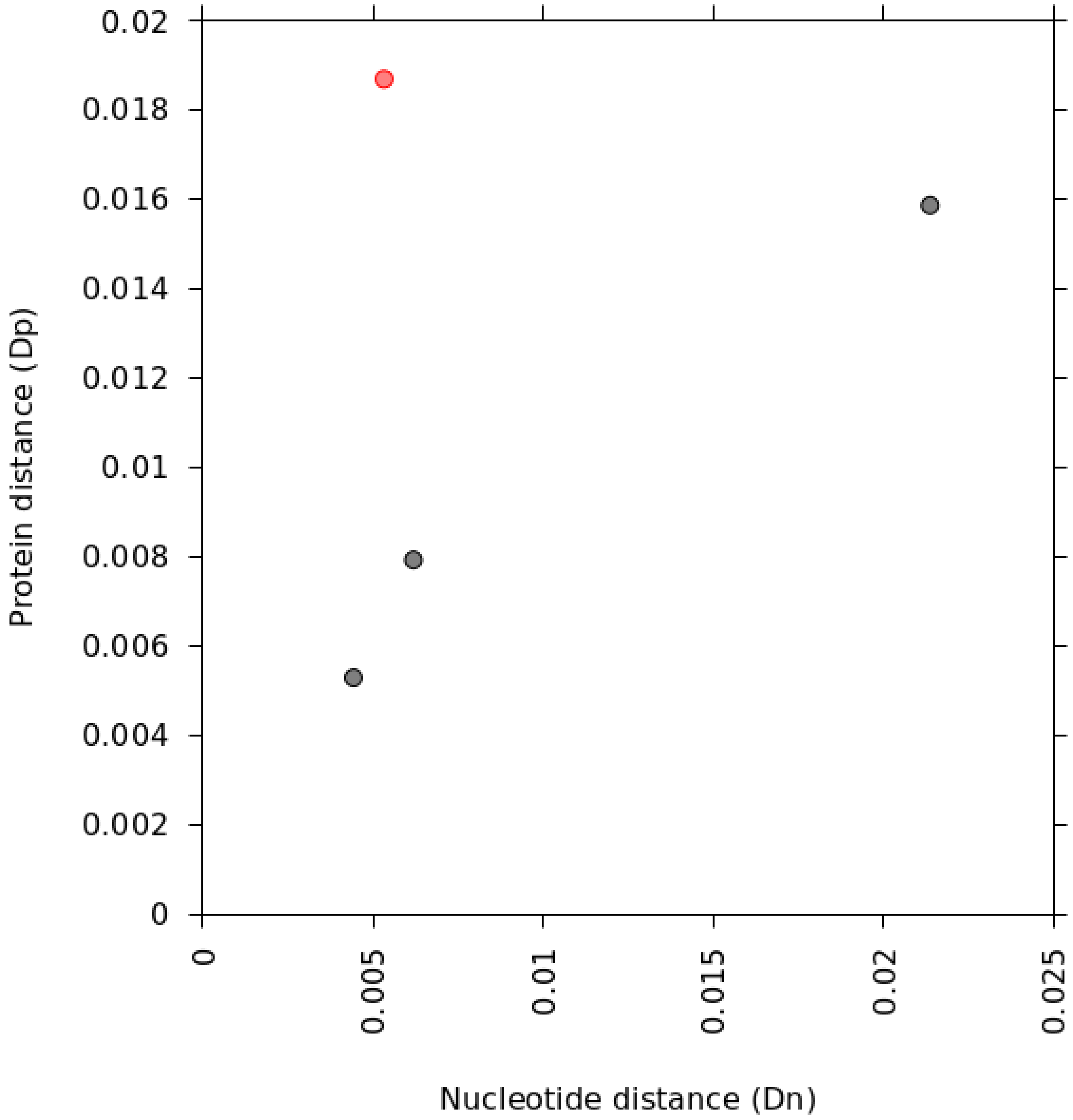

102. NC_003452.1/NP_602313.1

**CDS cluster ID**	**102**
**CDS cluster name**	NC_003452.1/NP_602313.1
**Total sequences**	3
**Reference forms**	2
**Compensatory frameshift forms**	1
**Virus**	American plum line pattern virus
**Protein**	putative polymerase p2

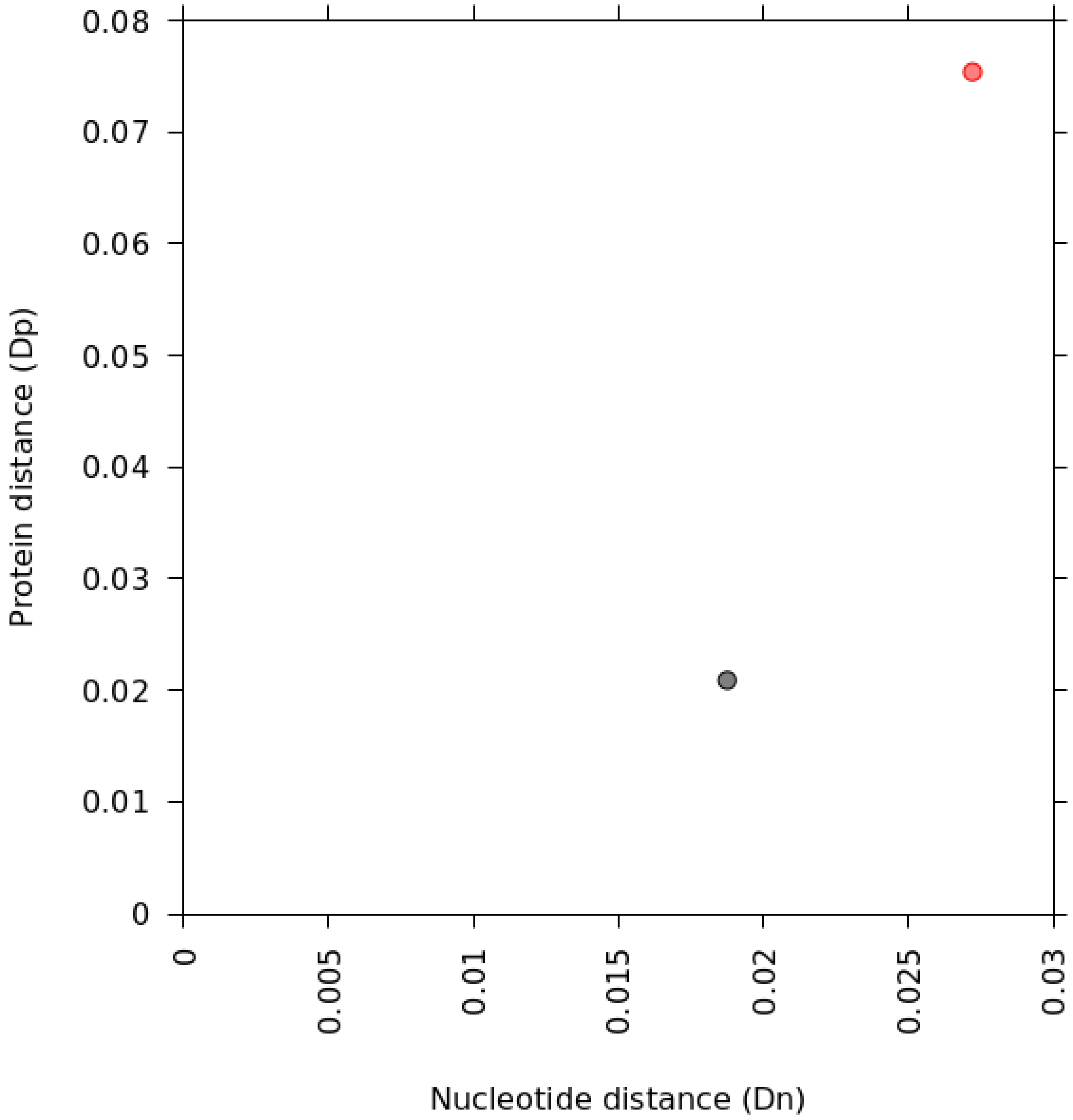

105. NC_003520.1/NP_612628.1

**CDS cluster ID**	**105**
**CDS cluster name**	NC_003520.1/NP_612628.1
**Total sequences**	5
**Reference forms**	4
**Compensatory frameshift forms**	1
**Virus**	Beet soil-borne virus (BSBV)
**Protein**	replicase (contains methyltransferase, helicase and RNA-dependent RNA polymerase domains)

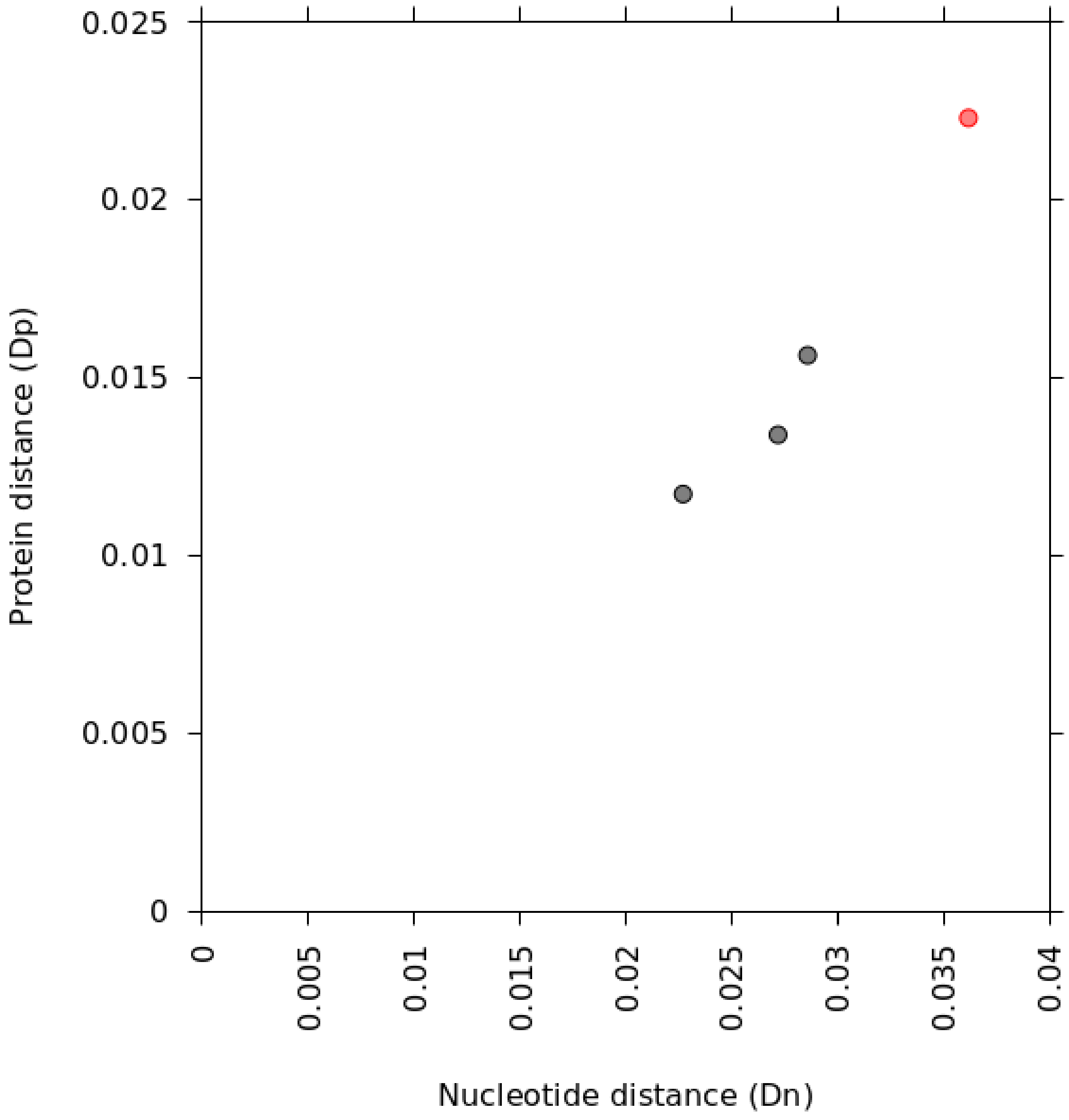

108. NC_003624.1/NP_619709.1

**CDS cluster ID**	**108**
**CDS cluster name**	NC_003624.1/NP_619709.1
**Total sequences**	30
**Reference forms**	29
Compensatory frameshift forms	1
**Virus**	Impatiens necrotic spot virus
**Protein**	nucleocapsid protein

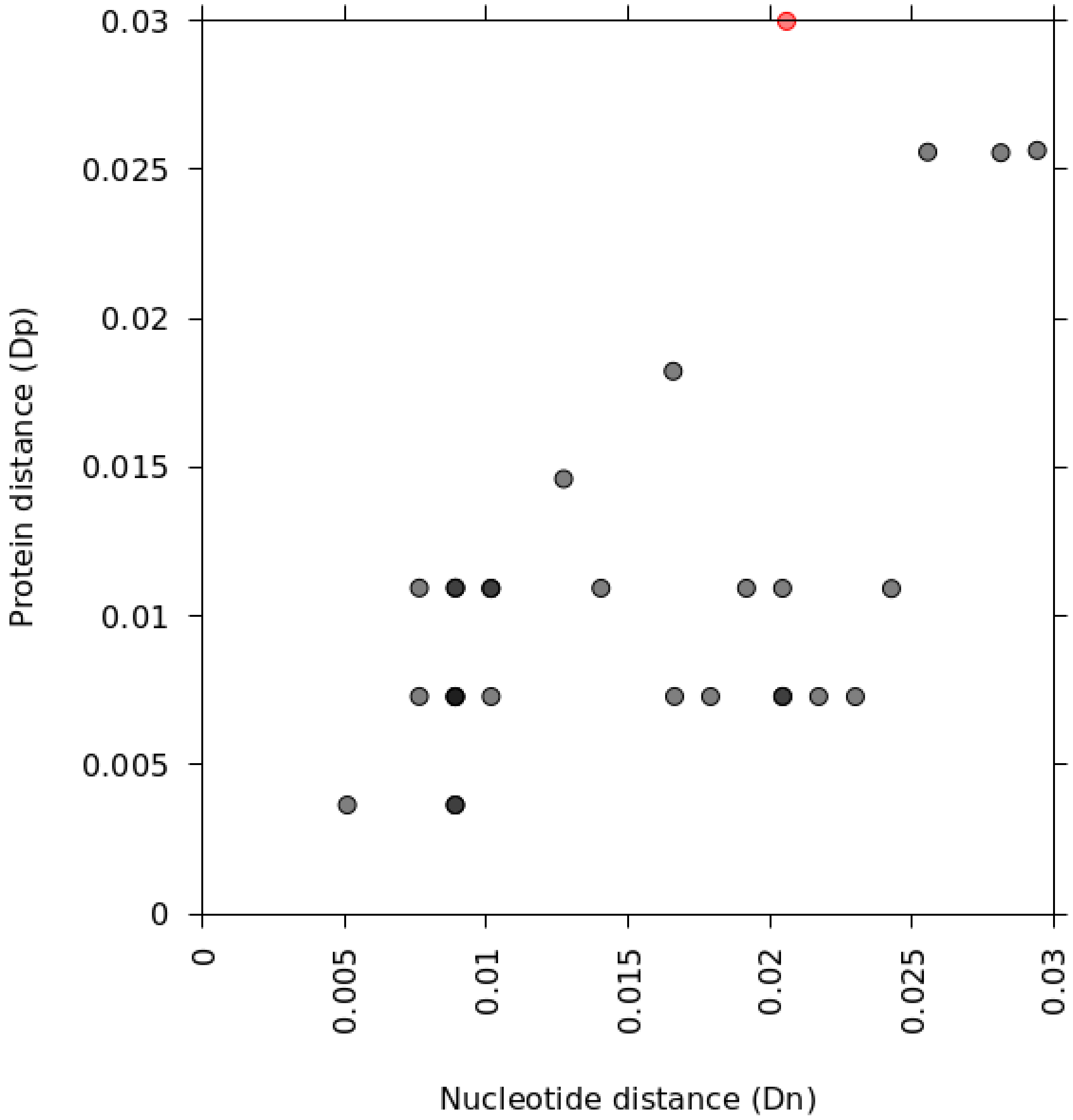

110. NC_003731.1/NP_620456.1

**CDS cluster ID**	**110**
**CDS cluster name**	NC_003731.1/NP_620456.1
**Total sequences**	25
**Reference forms**	24
**Compensatory frameshift forms**	1
**Virus**	Rice black streaked dwarf virus
**Protein**	hypothetical protein

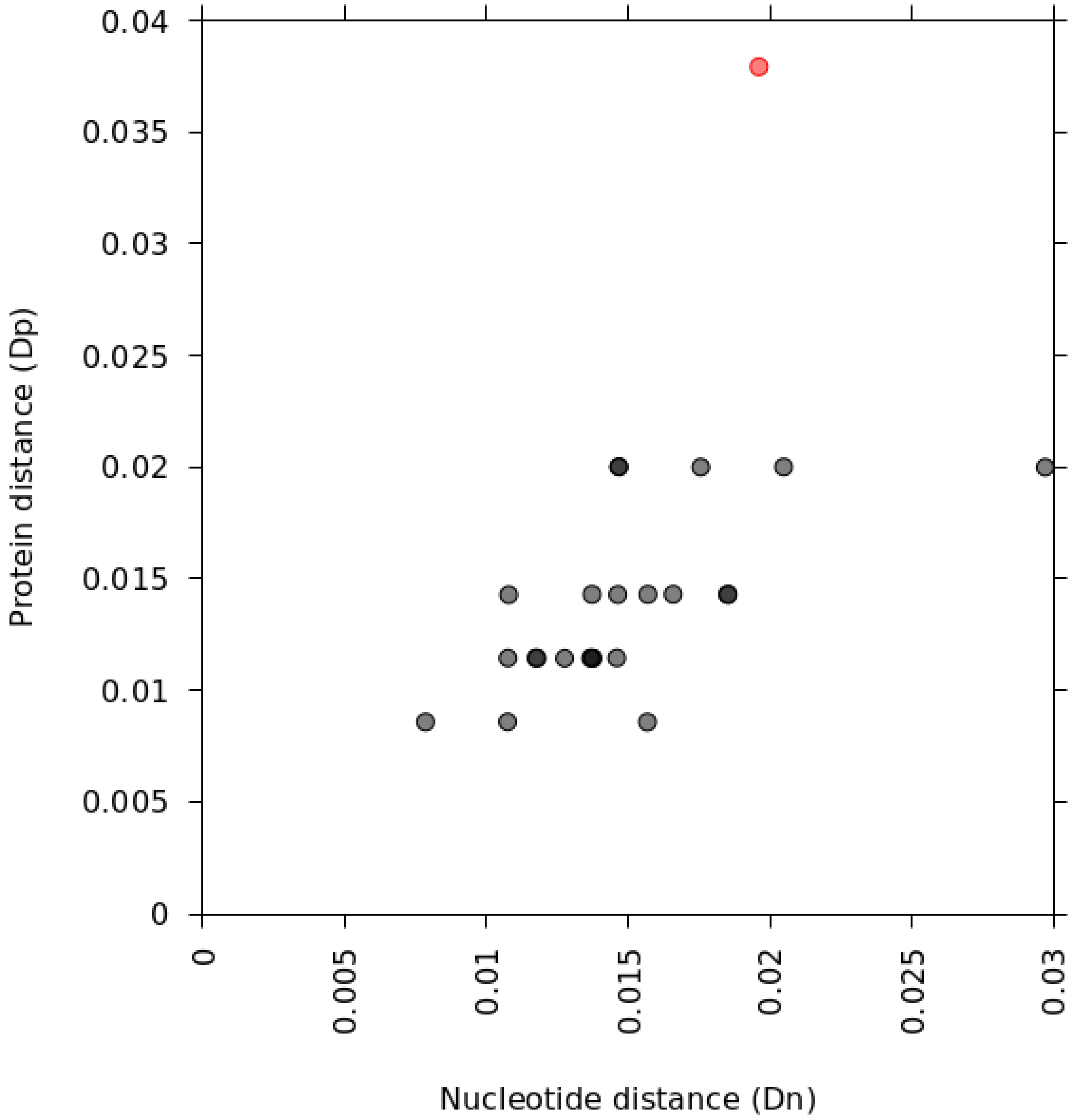

113. NC_003755.1/NP_620522.1

**CDS cluster ID**	**113**
**CDS cluster name**	NC_003755.1/NP_620522.1
**Total sequences**	25
**Reference forms**	24
**Compensatory frameshift forms**	1
**Virus**	Rice stripe tenuivirus
**Protein**	RNA Polymerase

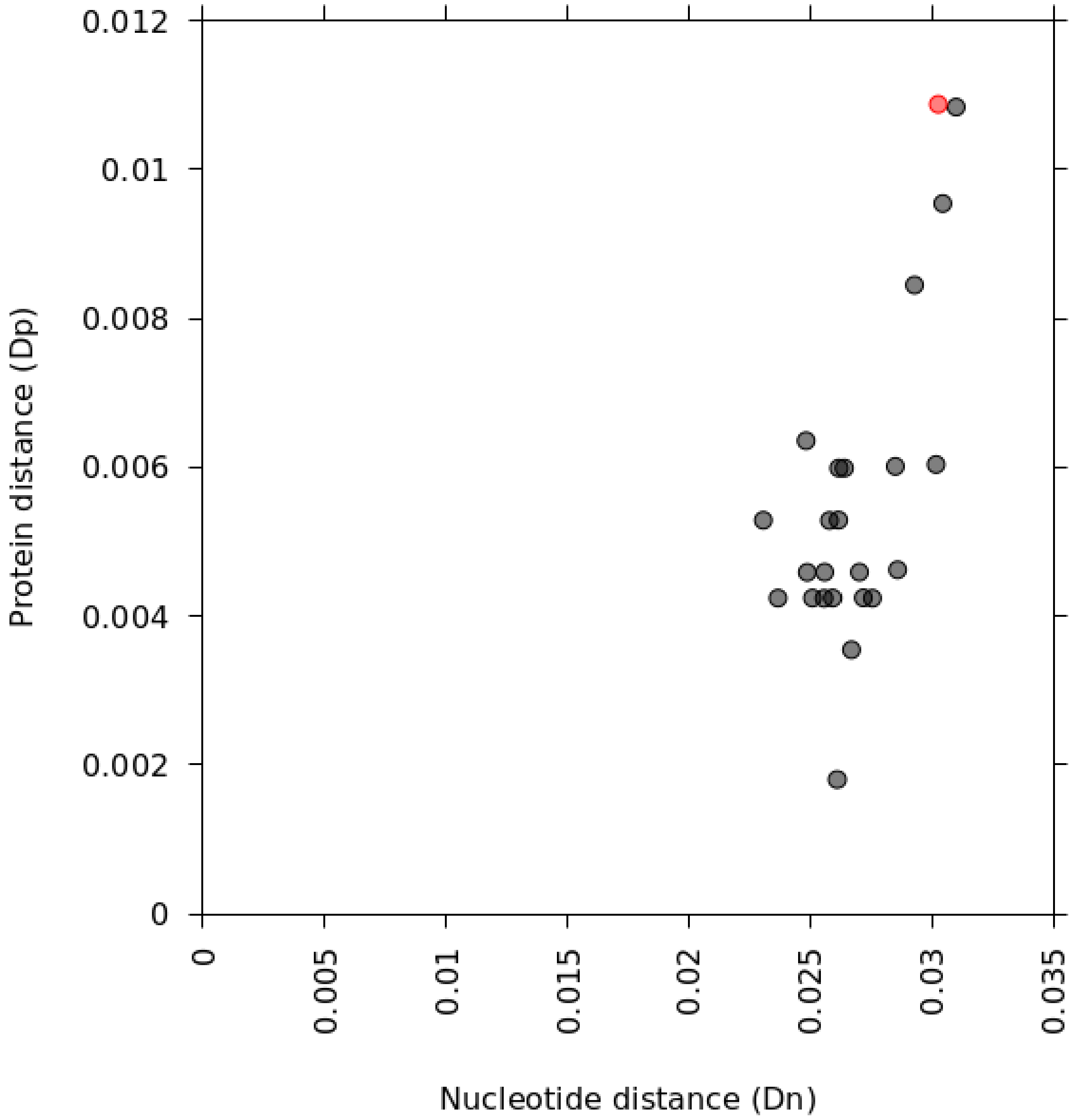

115. NC_003771.1/NP_620541.1

**CDS cluster ID**	**115**
**CDS cluster name**	NC_003771.1/NP_620541.1
**Total sequences**	4
**Reference forms**	3
**Compensatory frameshift forms**	1
**Virus**	Rice ragged stunt virus
**Protein**	RNA-dependent RNA polymerase

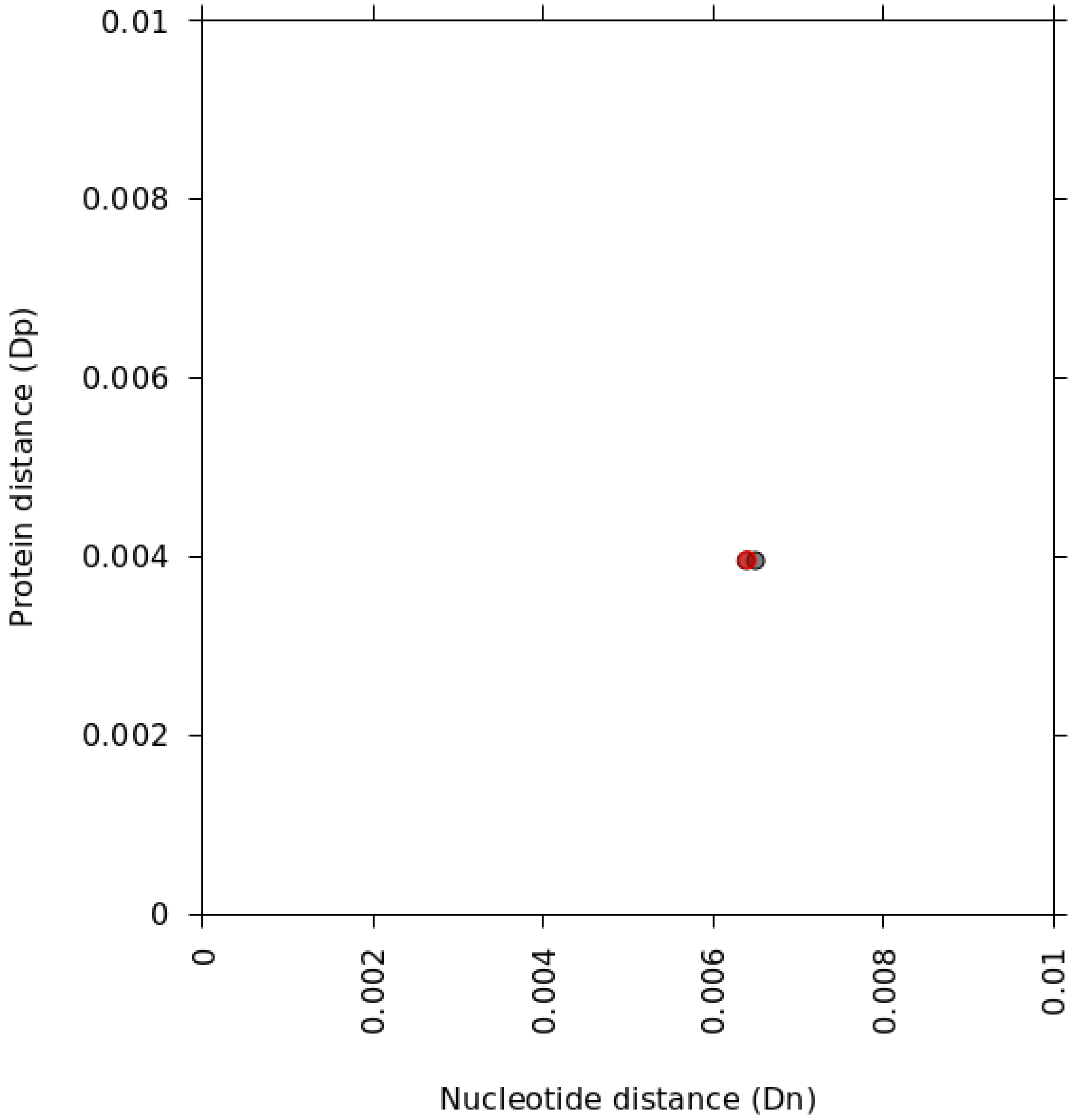

119. NC_003836.1/NP_620758.1

**CDS cluster ID**	**119**
**CDS cluster name**	NC_003836.1/NP_620758.1
**Total sequences**	16
**Reference forms**	14
**Compensatory frameshift forms**	1
**Virus**	Tomato aspermy virus
**Protein**	3a protein

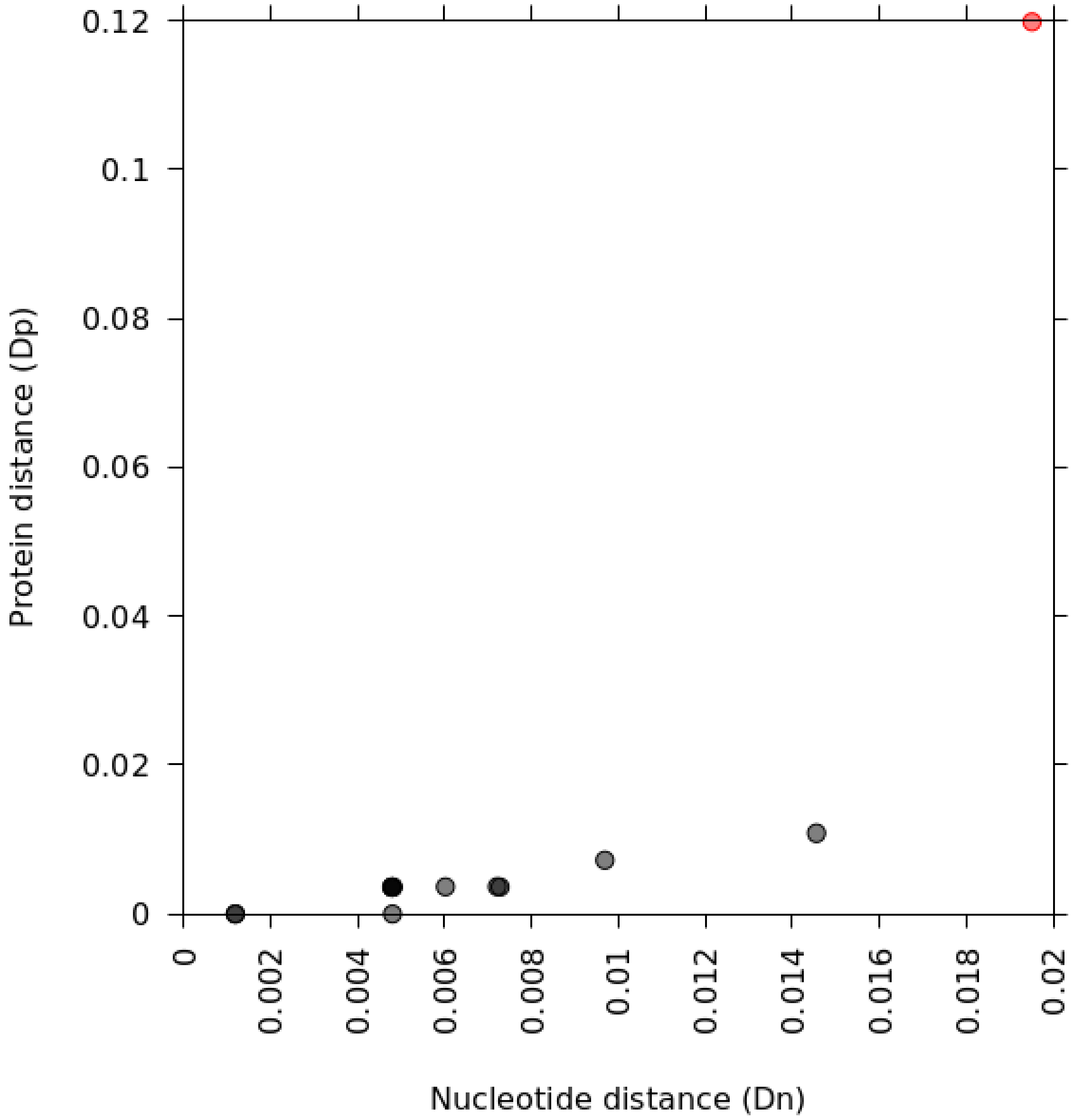

120. NC_003843.1/NP_620770.1

**CDS cluster ID**	**120**
**CDS cluster name**	NC_003843.1/NP_620770.1
**Total sequences**	7
**Reference forms**	6
**Compensatory frameshift forms**	1
**Virus**	Watermelon silver mottle tospovirus (WSMoV)
**Protein**	NSs

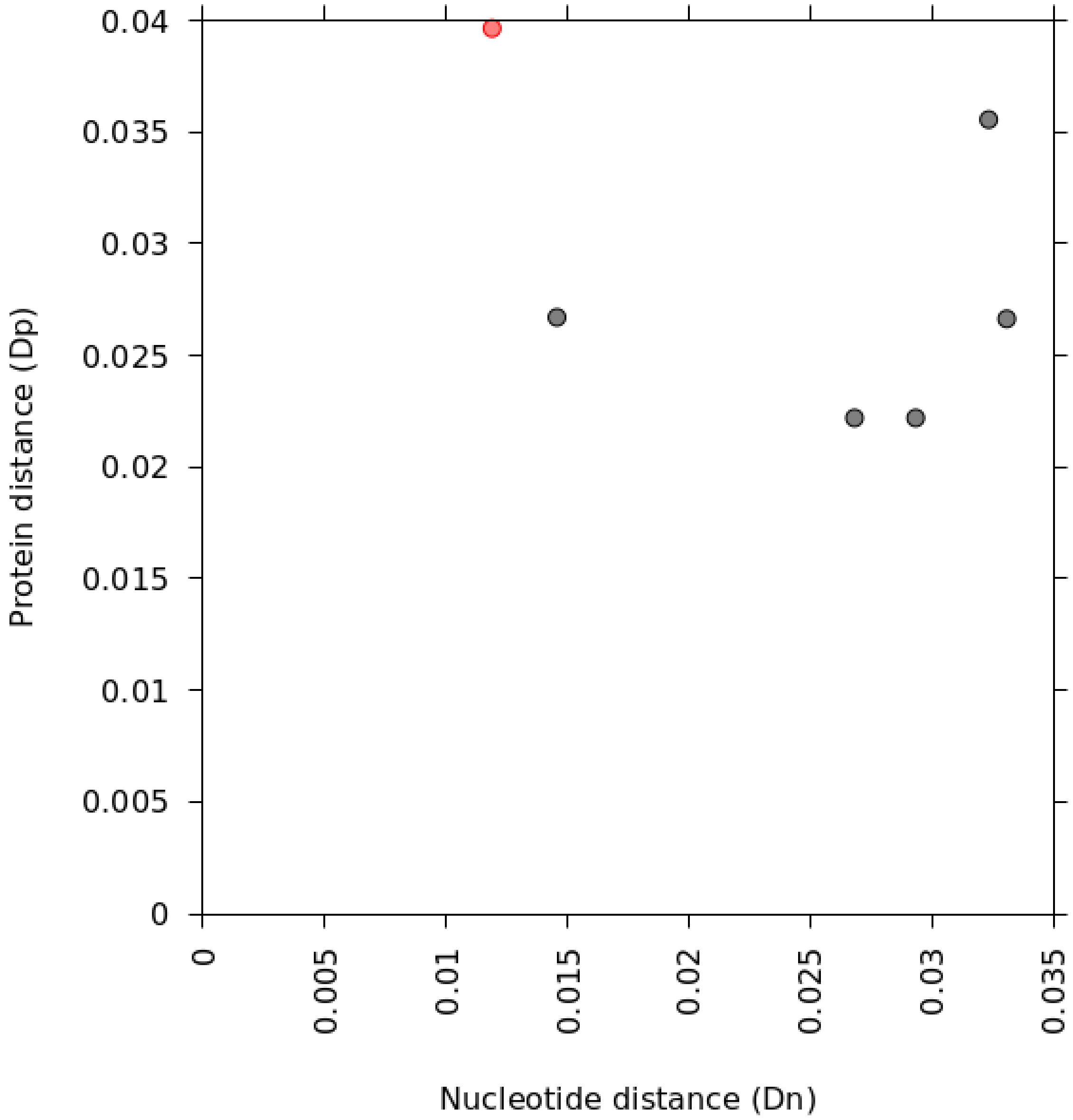

126. NC_005775.1/NP_982303.1

**CDS cluster ID**	**126**
**CDS cluster name**	NC_005775.1/NP_982303.1
**Total sequences**	42
**Reference forms**	41
**Compensatory frameshift forms**	1
**Virus**	Oropouche virus
**Protein**	polyprotein

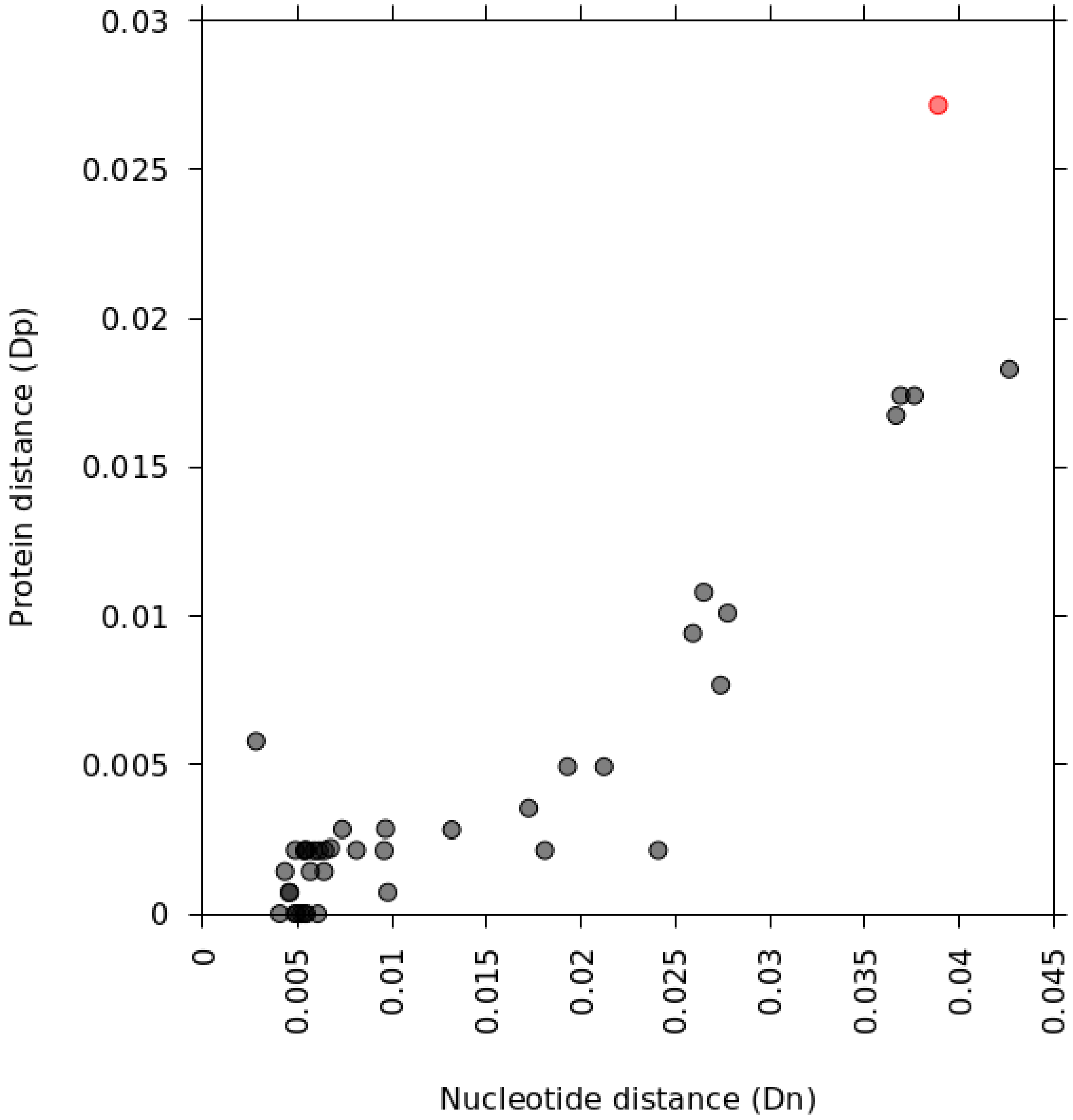

128. NC_006008.2/YP_052953.2

**CDS cluster ID**	**128**
**CDS cluster name**	NC_006008.2/YP_052953.2
**Total sequences**	27
**Reference forms**	26
**Compensatory frameshift forms**	1
**Virus**	Bluetongue virus
**Protein**	VP6 protein

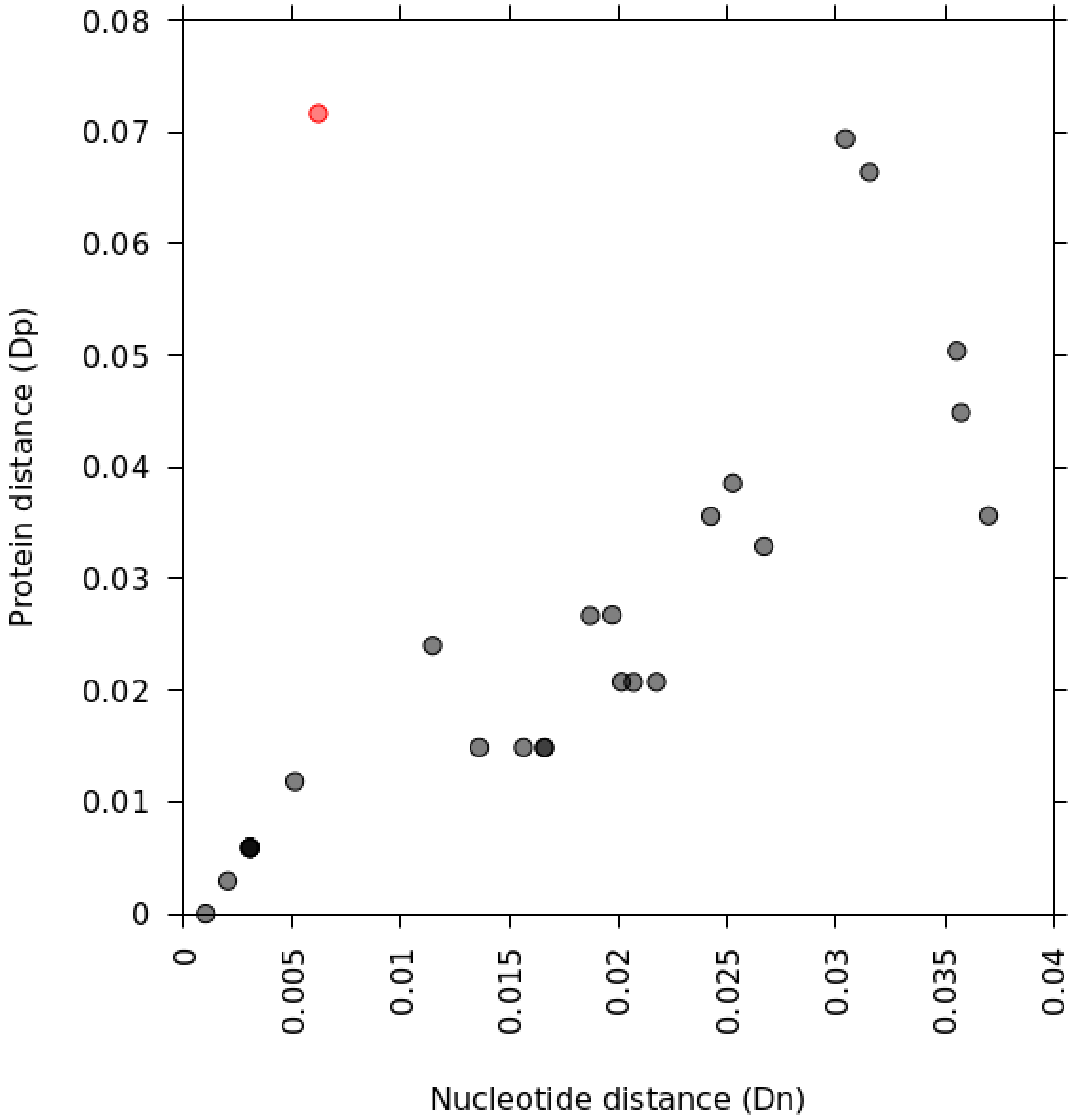

129. NC_006383.2/YP_133822.1

**CDS cluster ID**	**129**
**CDS cluster name**	NC_006383.2/YP_133822.1
**Total sequences**	41
**Reference forms**	40
**Compensatory frameshift forms**	1
**Virus**	Peste des petits ruminants virus
**Protein**	phosphoprotein

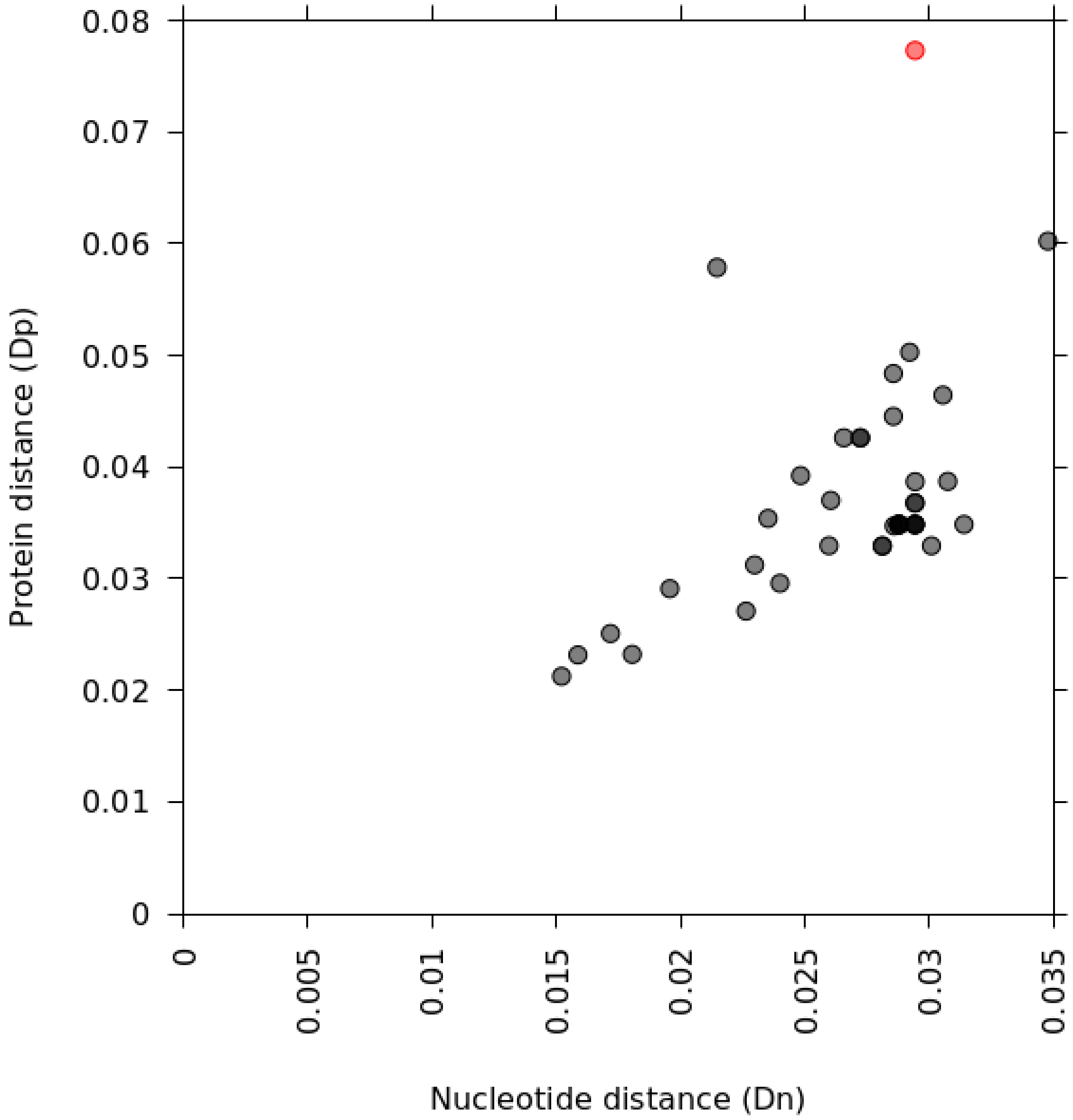

130. NC_006943.1/YP_224085.1

**CDS cluster ID**	**130**
**CDS cluster name**	NC_006943.1/YP_224085.1
**Total sequences**	3
**Reference forms**	2
**Compensatory frameshift forms**	1
**Virus**	Hydrangea ringspot virus
**Protein**	triple gene block protein

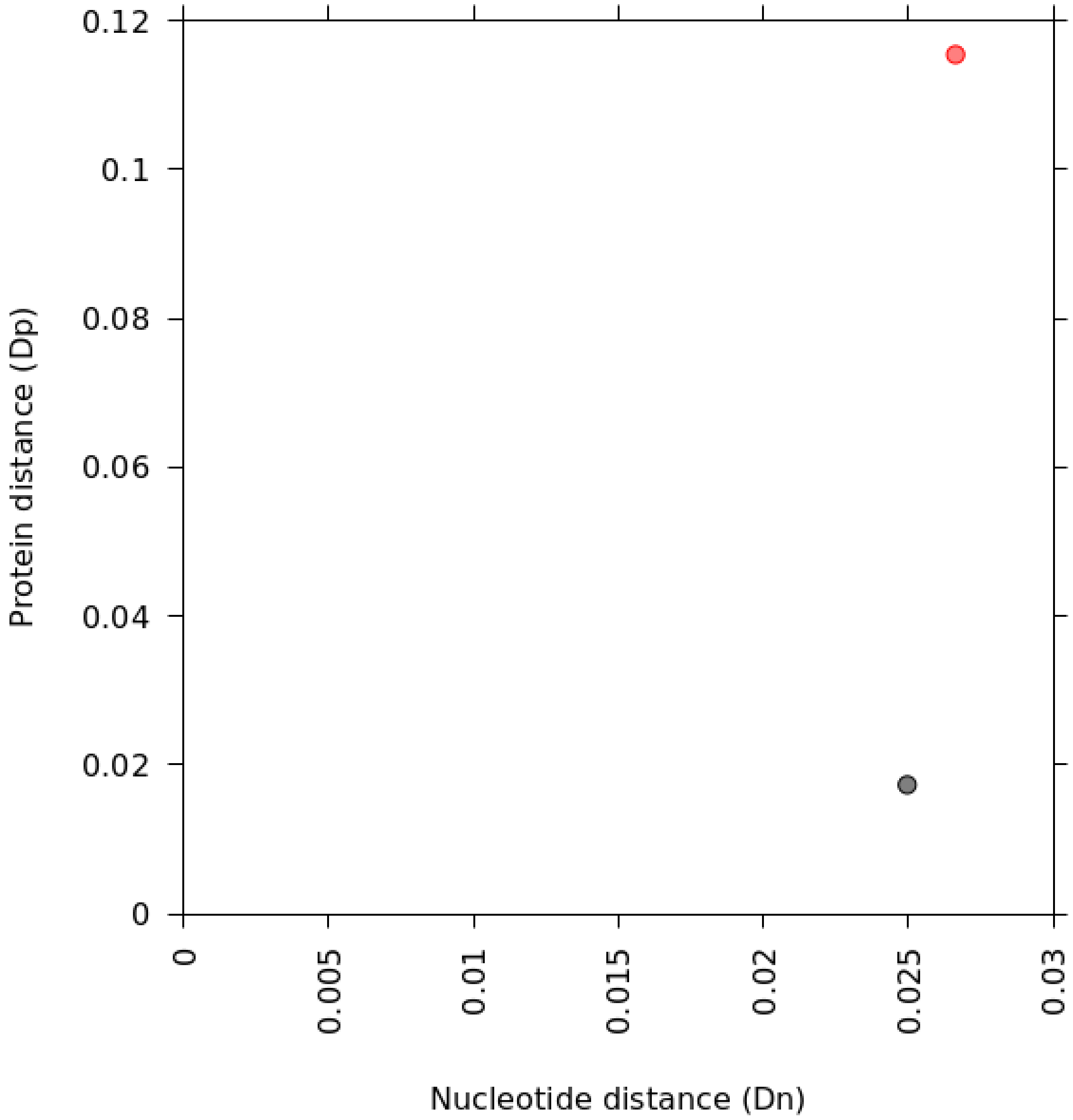

131. NC_007341.1/YP_293699.1

**CDS cluster ID**	**131**
**CDS cluster name**	NC_007341.1/YP_293699.1
**Total sequences**	49
**Reference forms**	48
**Compensatory frameshift forms**	1
**Virus**	Tomato chlorosis virus
**Protein**	heat shock protein 70

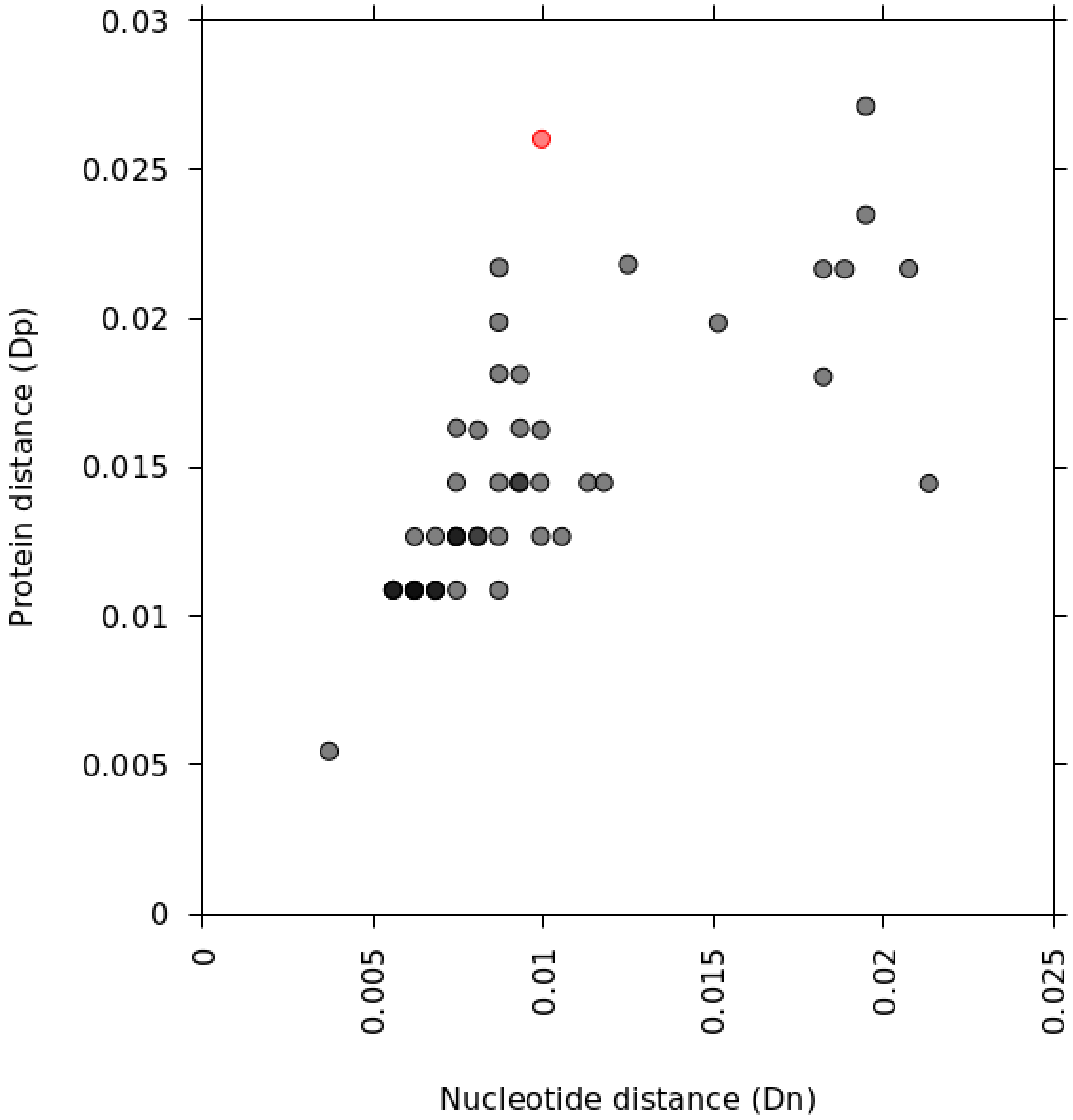

134. NC_008040.1/YP_611155.1

**CDS cluster ID**	**134**
**CDS cluster name**	NC_008040.1/YP_611155.1
**Total sequences**	35
**Reference forms**	34
**Compensatory frameshift forms**	1
**Virus**	Redspotted grouper nervous necrosis virus
**Protein**	protein A

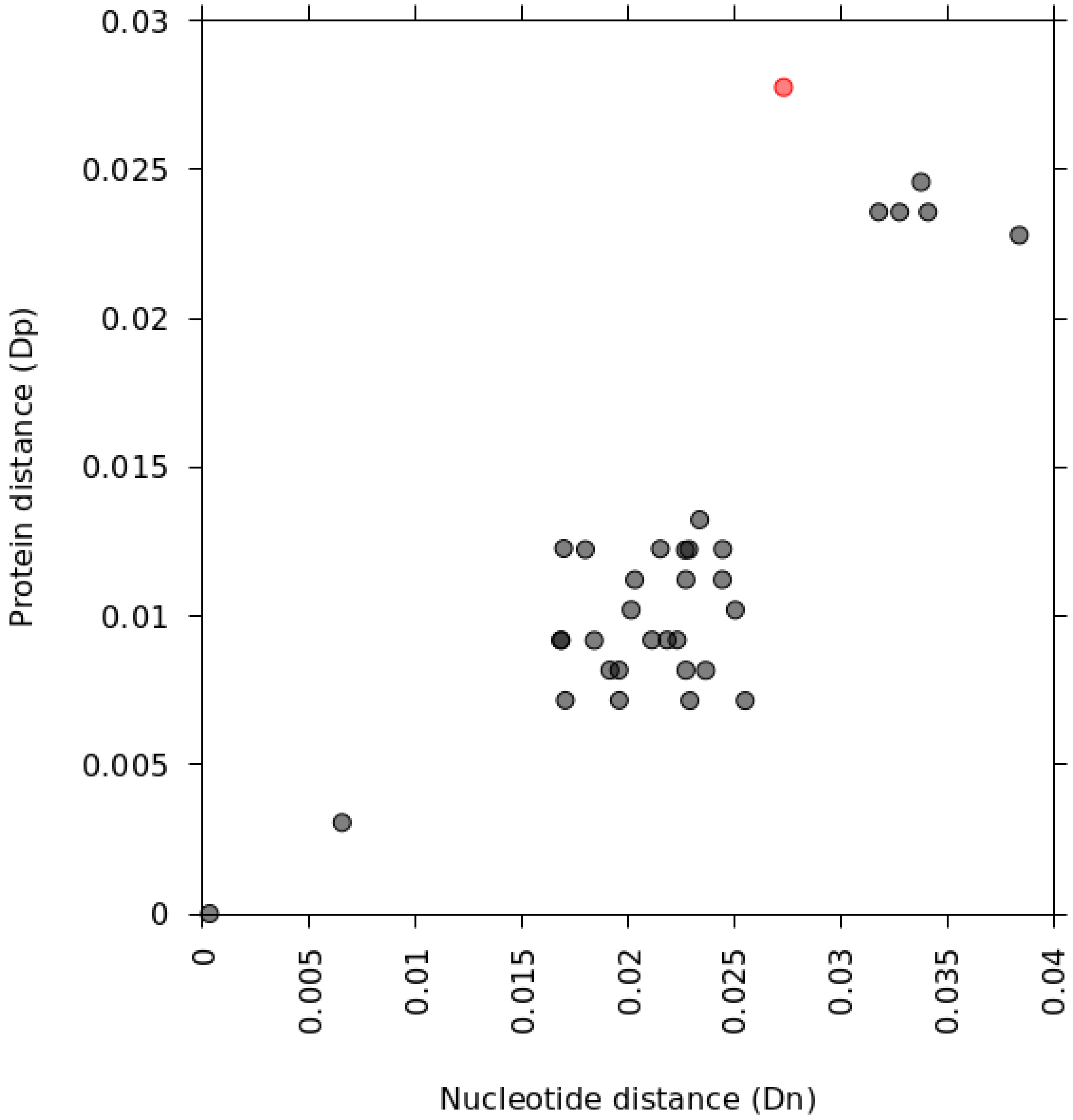

136. NC_009019.1/YP_001039952.1

**CDS cluster ID**	**136**
**CDS cluster name**	NC_009019.1/YP_001039952.1
**Total sequences**	3
**Reference forms**	2
**Compensatory frameshift forms**	1
**Virus**	Tylonycteris bat coronavirus HKU4
**Protein**	orf1ab polyprotein

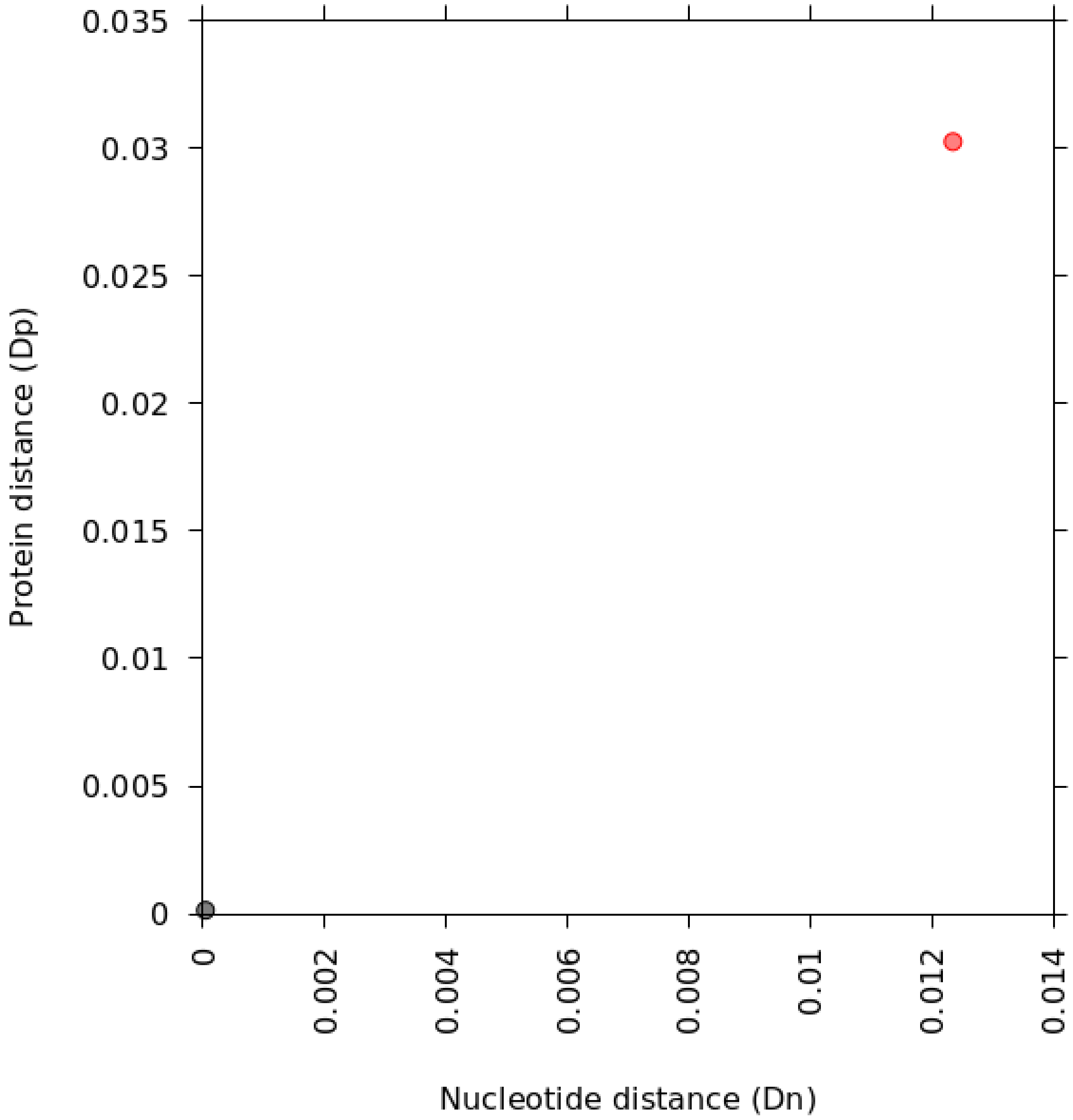

143. NC_012936.1/YP_003029844.1

**CDS cluster ID**	**143**
**CDS cluster name**	NC_012936.1/YP_003029844.1
**Total sequences**	3
**Reference forms**	2
**Compensatory frameshift forms**	1
**Virus**	Rat coronavirus Parker
**Protein**	orf1ab polyprotein

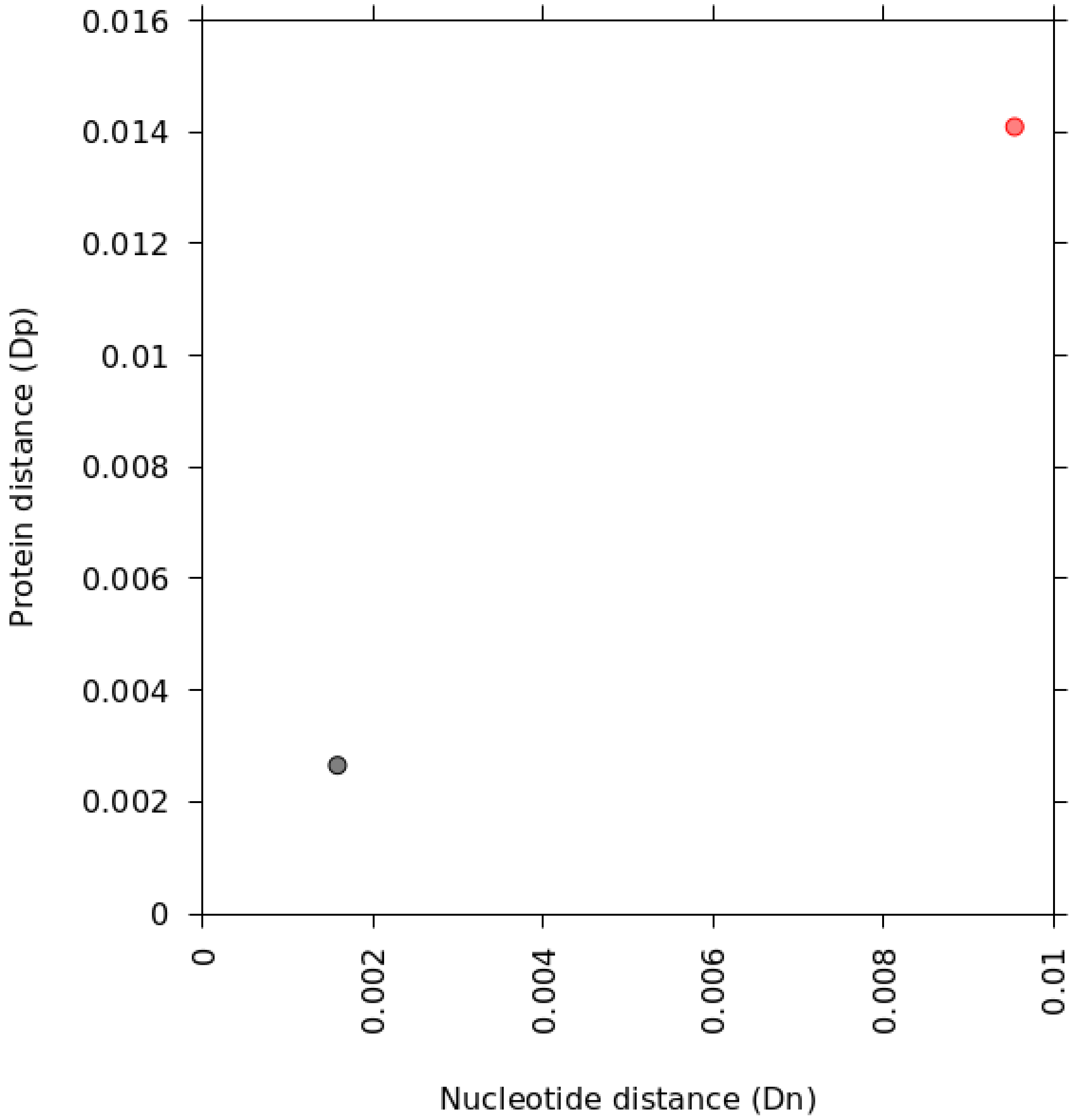

144. NC_012936.1/YP_003029851.1

**CDS cluster ID**	**144**
**CDS cluster name**	NC_012936.1/YP_003029851.1
**Total sequences**	4
**Reference forms**	3
**Compensatory frameshift forms**	1
**Virus**	Rat coronavirus Parker
**Protein**	membrane protein

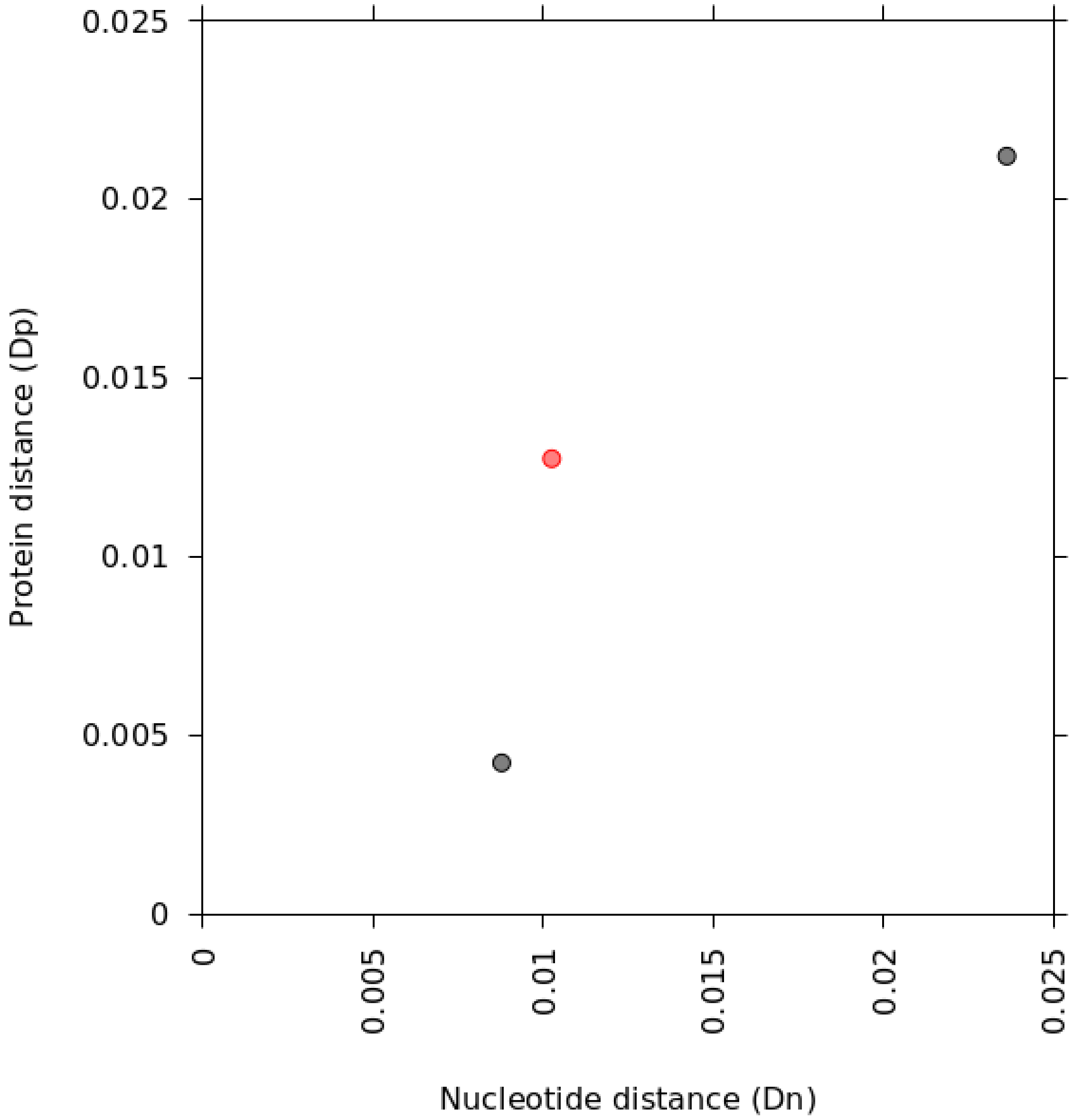

146. NC_013398.1/YP_003240110.1

**CDS cluster ID**	**146**
**CDS cluster name**	NC_013398.1/YP_003240110.1
**Total sequences**	20
**Reference forms**	19
**Compensatory frameshift forms**	1
**Virus**	Epizootic hemorrhagic disease virus (serotype 1 / strain New Jersey)
**Protein**	VP3 protein

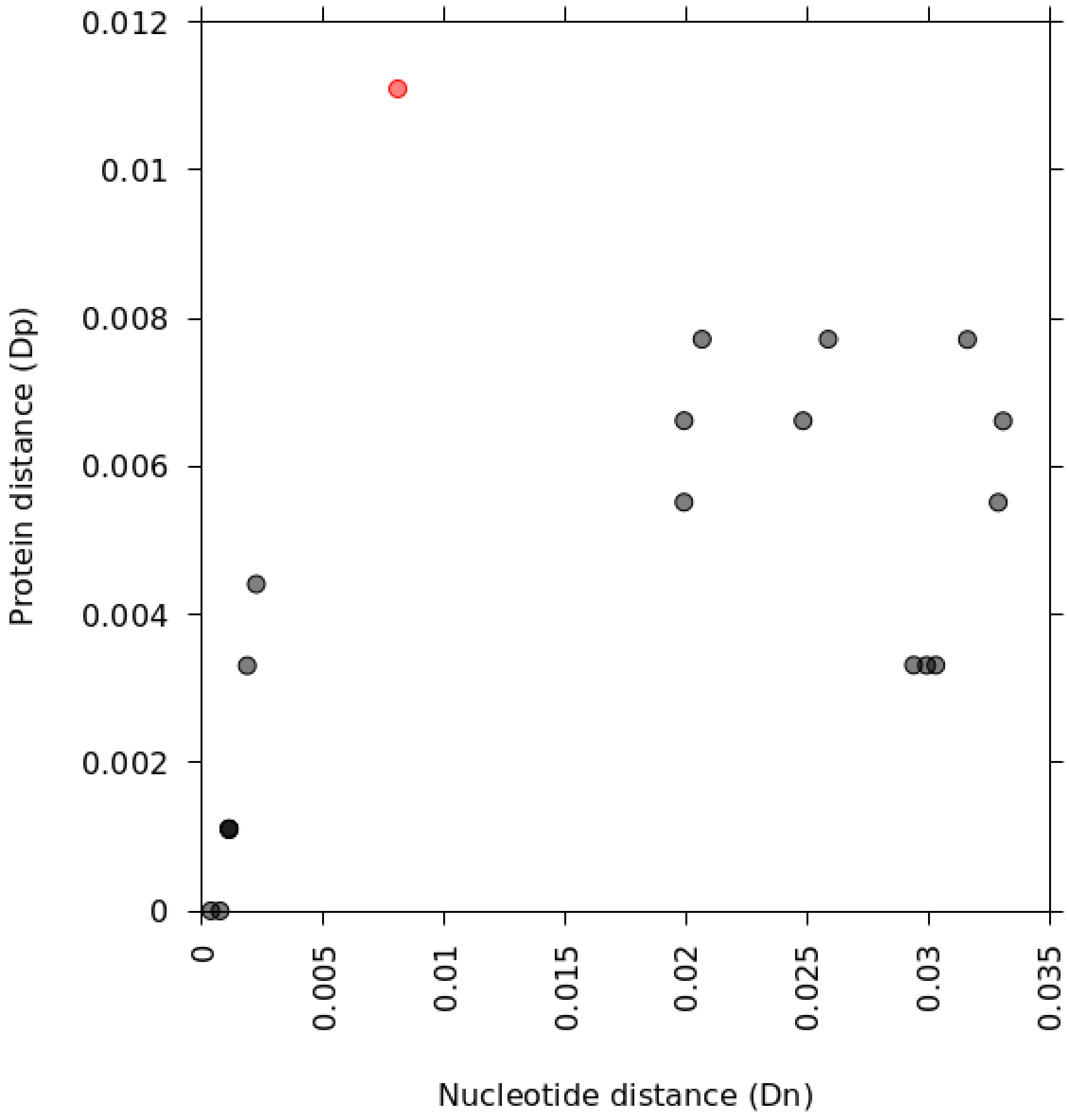

147. NC_013400.1/YP_003240112.1

CDS cluster ID	147
CDS cluster name	NC_013400.1/YP_003240112.1
Total sequences	47
Reference forms	46
Compensatory frameshift forms	1
Virus	Epizootic hemorrhagic disease virus (serotype 1 / strain New Jersey)
Protein	NS1 protein

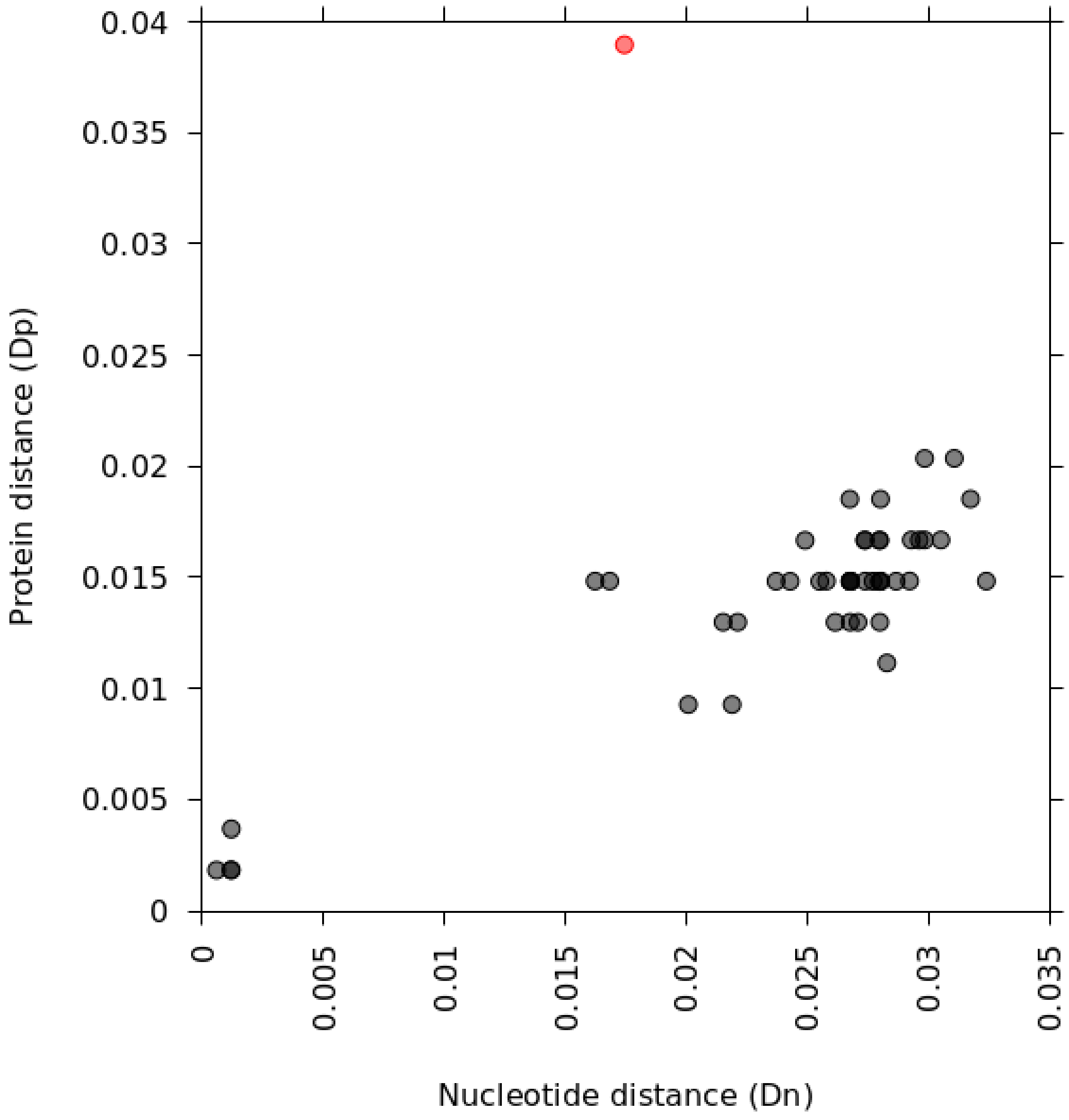

148. NC_014716.1/YP_004021938.1

**CDS cluster ID**	**148**
**CDS cluster name**	NC_014716.1/YP_004021938.1
**Total sequences**	8
**Reference forms**	7
**Compensatory frameshift forms**	1
**Virus**	Southern rice black-streaked dwarf virus
**Protein**	P3

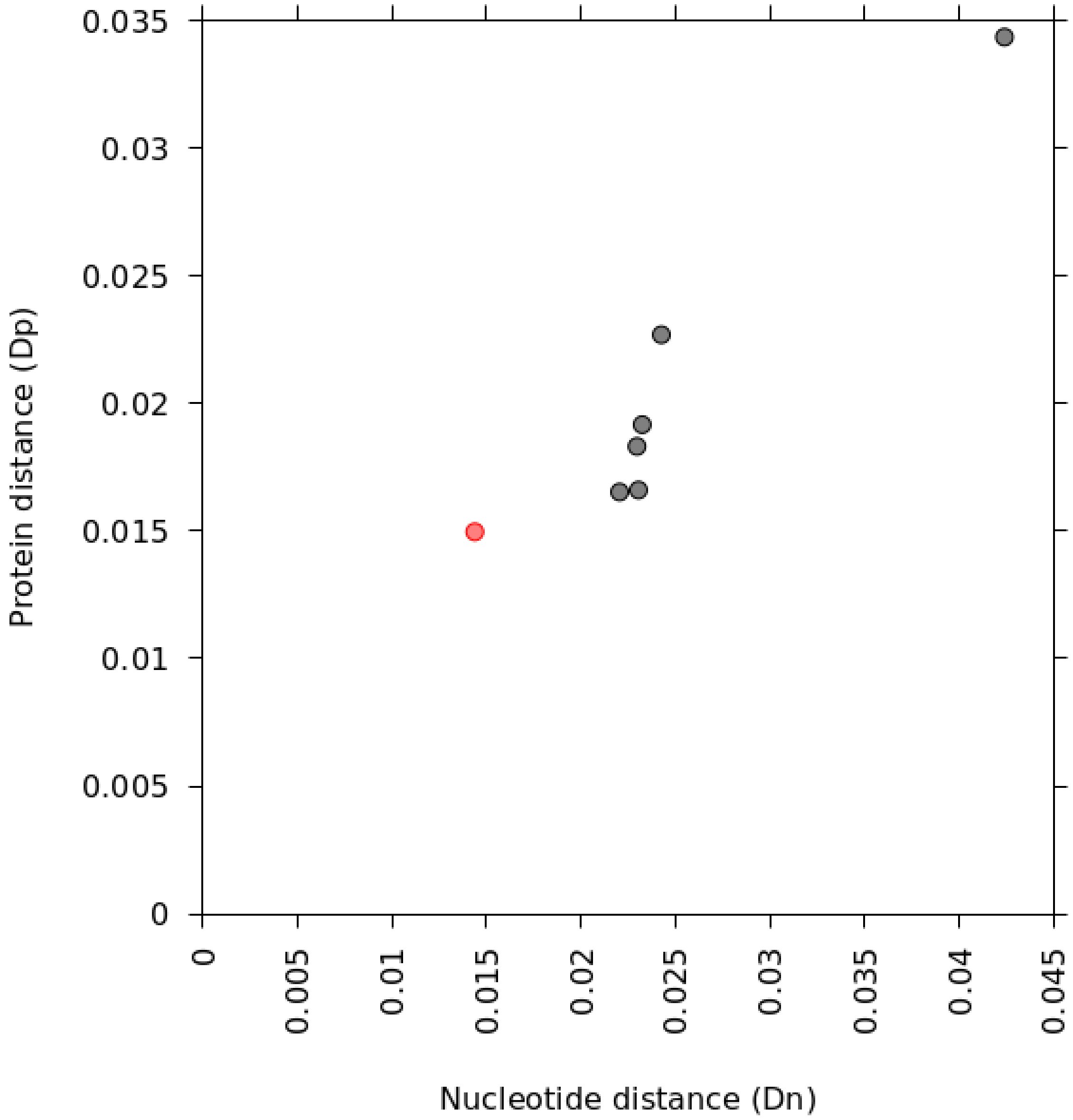

149. NC_015300.1/YP_004327591.1

CDS cluster ID	149
CDS cluster name	NC_015300.1/YP_004327591.1
Total sequences	85
Reference forms	84
Compensatory frameshift forms	1
Virus	Rose rosette emaravirus
Protein	putative nucleocapsid p3

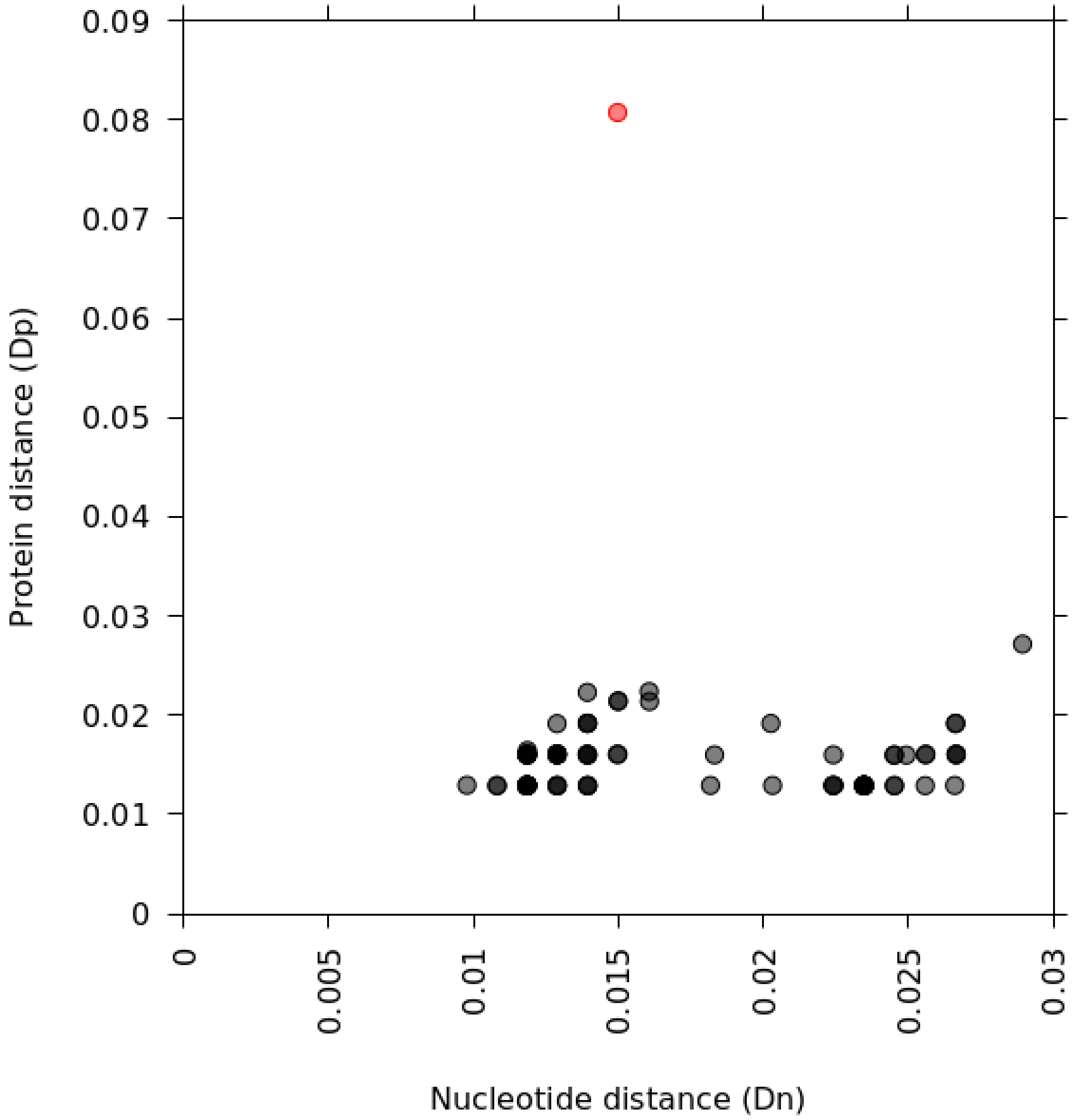

150. NC_015874.1/YP_004767305.1

**CDS cluster ID**	**150**
**CDS cluster name**	NC_015874.1/YP_004767305.1
**Total sequences**	25
**Reference forms**	24
**Compensatory frameshift forms**	1
**Virus**	Nam Dinh virus
**Protein**	pp1ab polyprotein

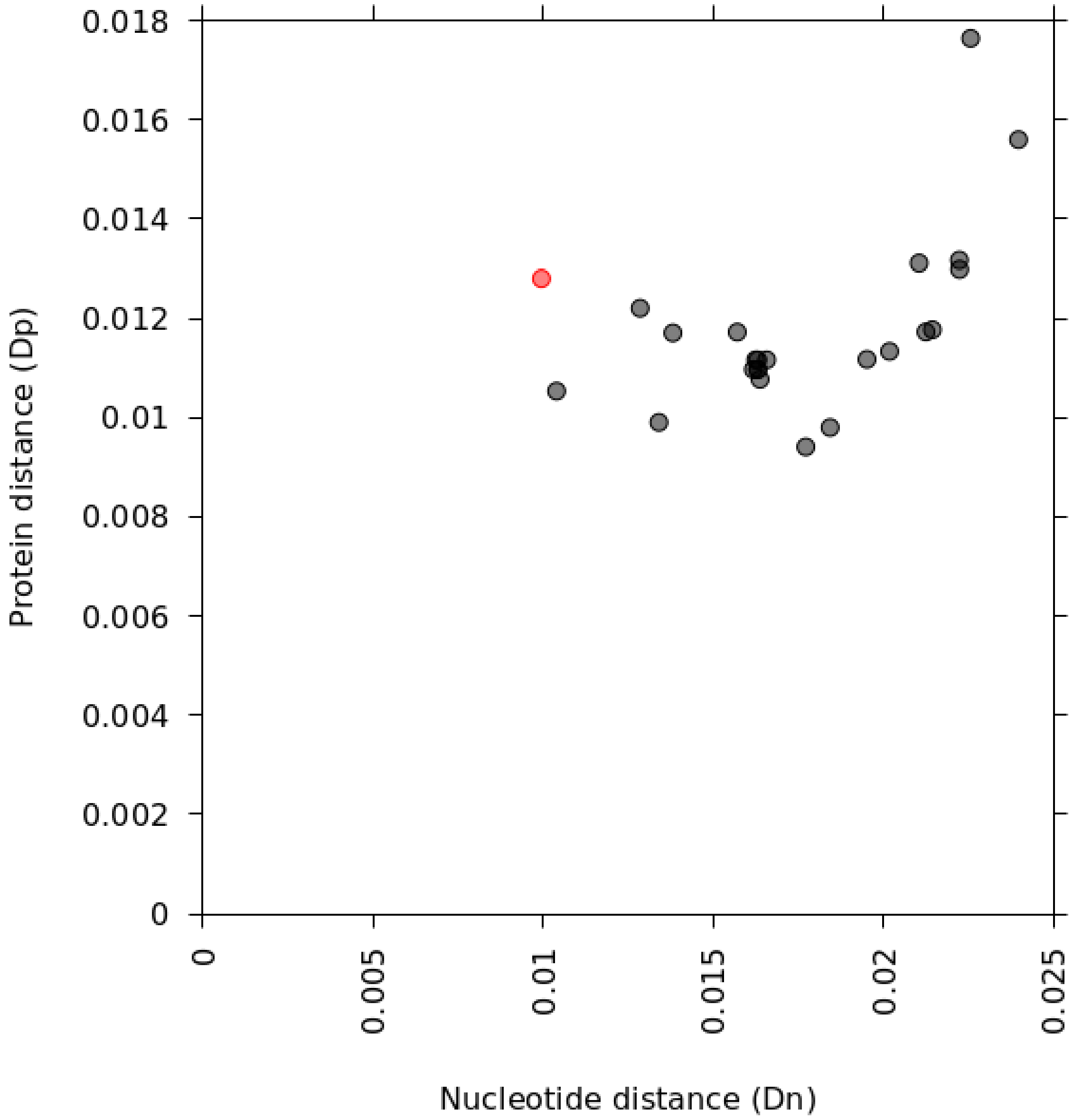

151. NC_016038.2/YP_004869995.3

**CDS cluster ID**	**151**
**CDS cluster name**	NC_016038.2/YP_004869995.3
**Total sequences**	13
**Reference forms**	12
**Compensatory frameshift forms**	1
**Virus**	Brassica yellows virus
**Protein**	read-through protein

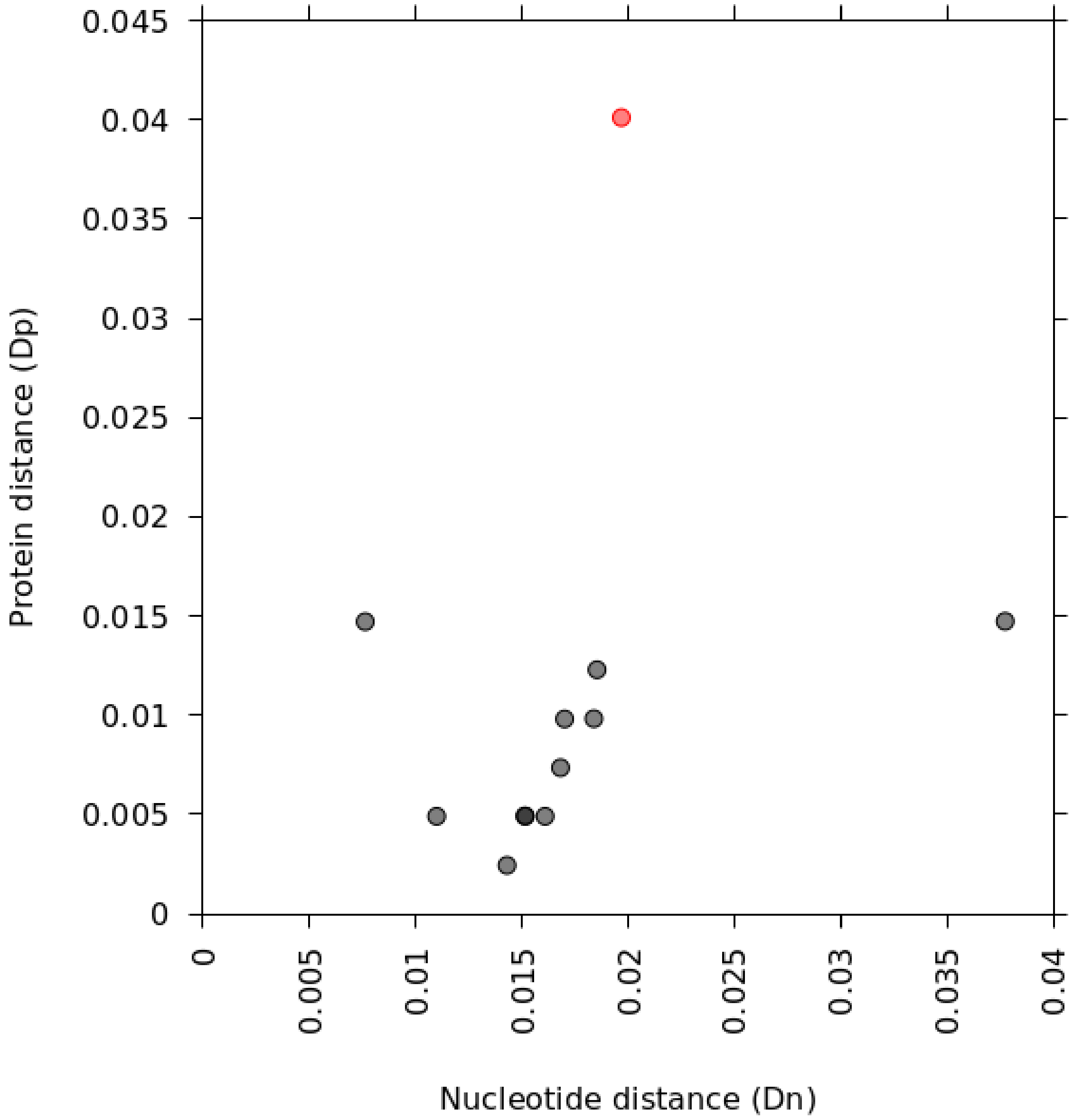

152. NC_016959.1/YP_005351233.1

**CDS cluster ID**	**152**
**CDS cluster name**	NC_016959.1/YP_005351233.1
**Total sequences**	8
**Reference forms**	7
**Compensatory frameshift forms**	1
**Virus**	Ndumu virus
**Protein**	structural polyprotein

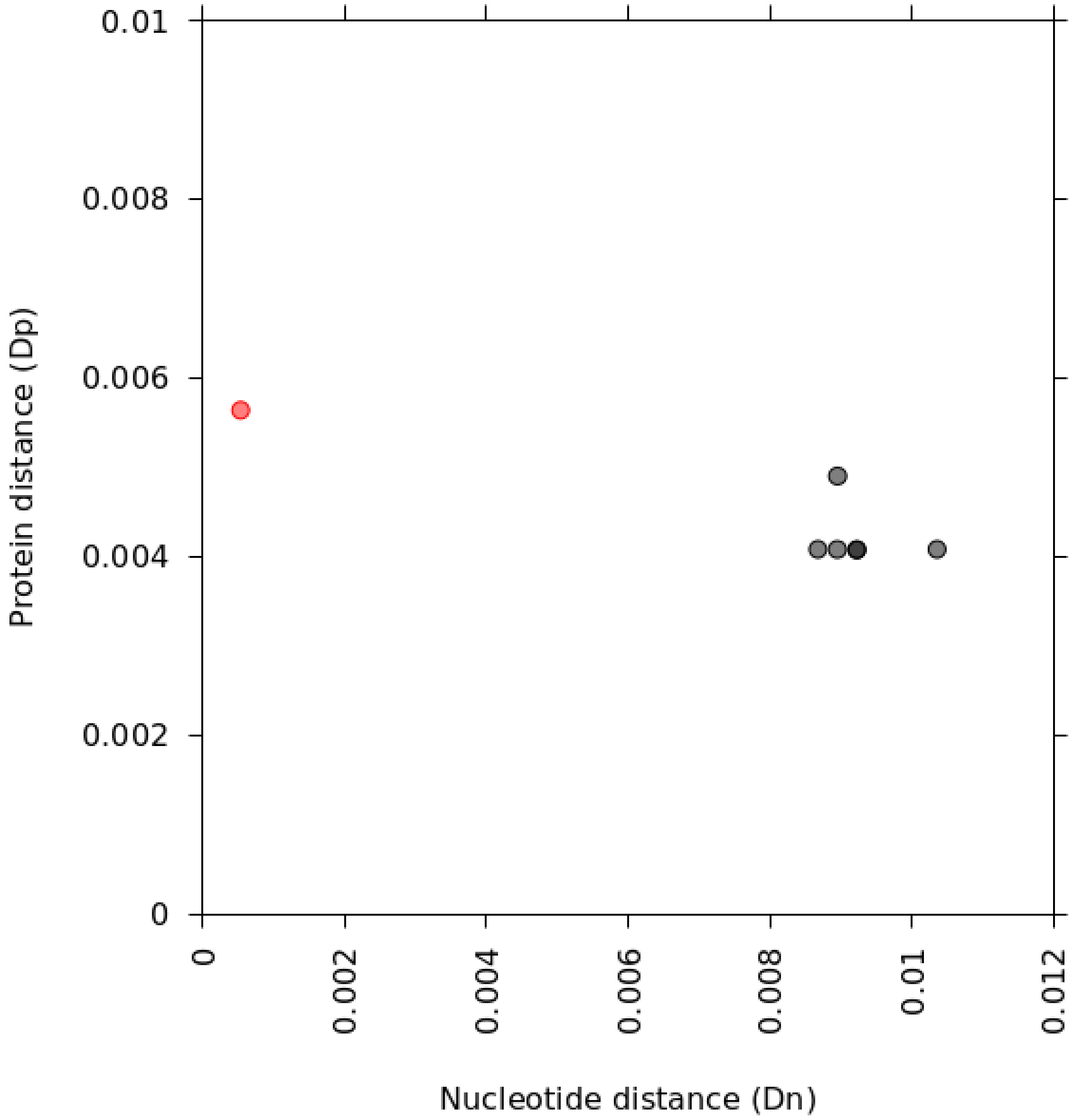

154. NC_019843.3/YP_009047202.1

**CDS cluster ID**	**154**
**CDS cluster name**	NC_019843.3/YP_009047202.1
**Total sequences**	413
**Reference forms**	410
**Compensatory frameshift forms**	1
**Virus**	Middle East respiratory syndrome-related coronavirus (MERS-CoV)
**Protein**	1AB polyprotein

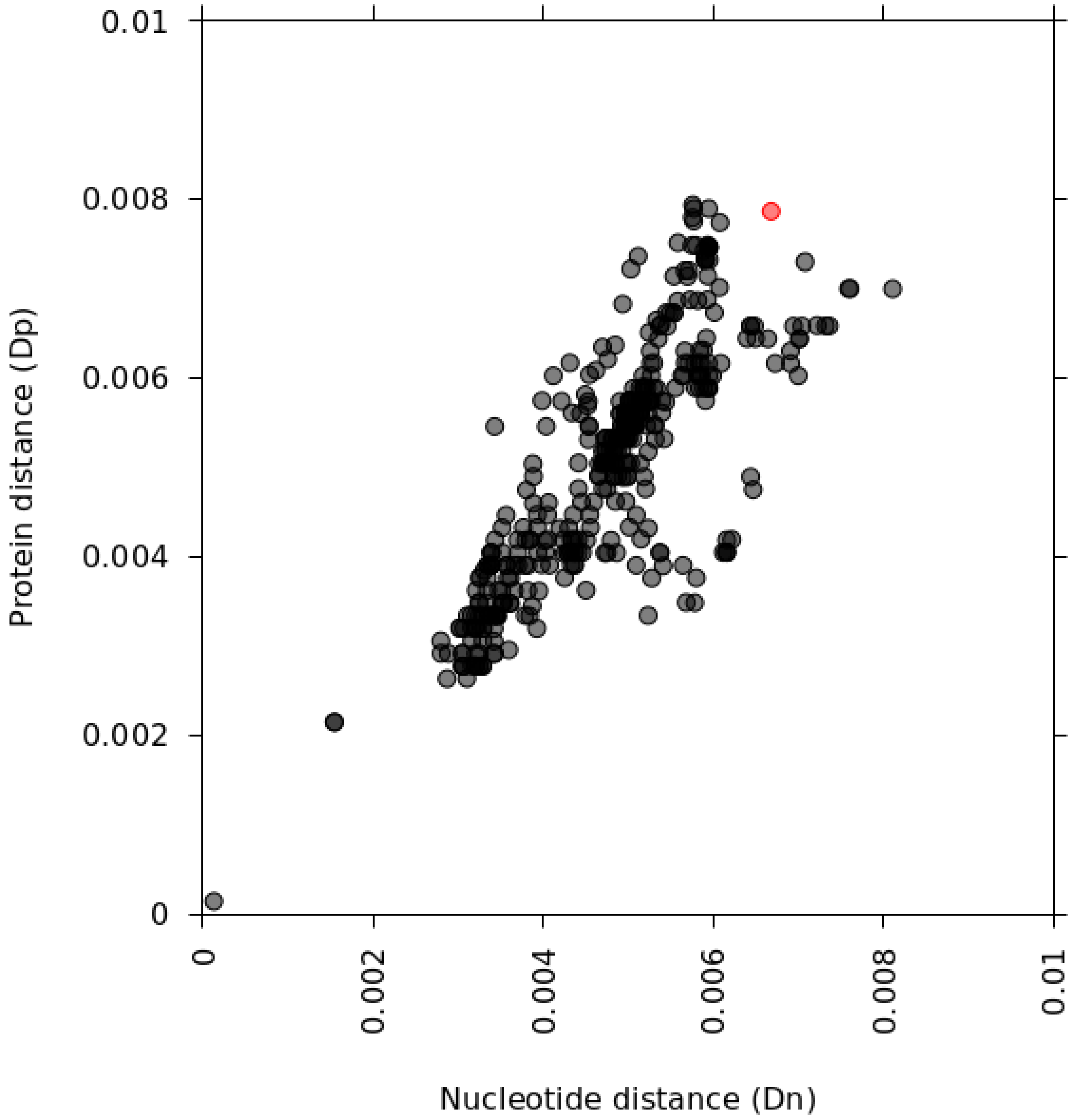

155. NC_021928.1/YP_008378663.1

**CDS cluster ID**	**155**
**CDS cluster name**	NC_021928.1/YP_008378663.1
**Total sequences**	3
**Reference forms**	2
**Compensatory frameshift forms**	1
**Virus**	Human parainfluenza virus 4a
**Protein**	fusion protein

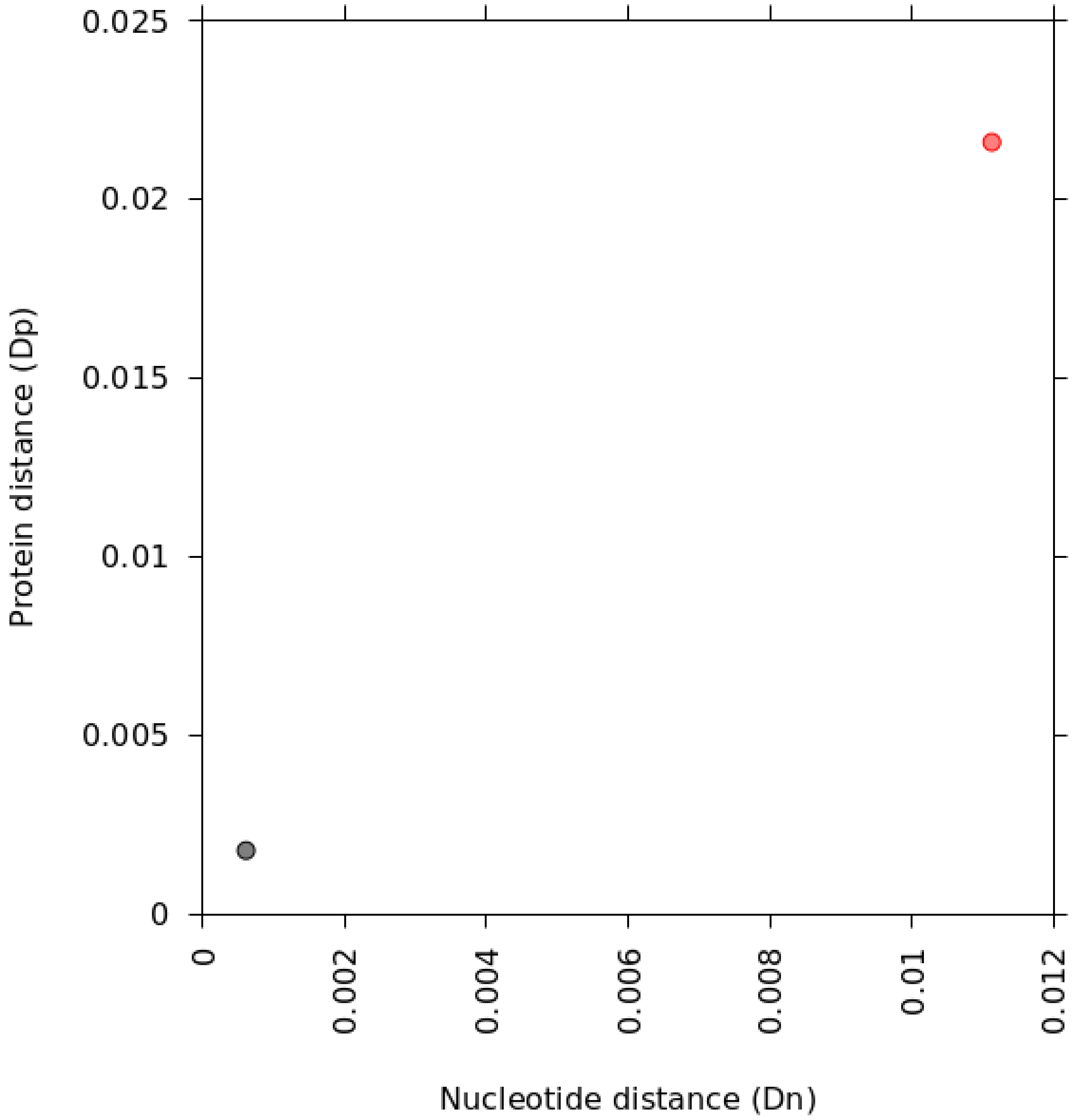

161. NC_026423.1/YP_009118471.1

**CDS cluster ID**	**161**
**CDS cluster name**	NC_026423.1/YP_009118471.1
**Total sequences**	1241
**Reference forms**	1240
**Compensatory frameshift forms**	1
**Virus**	Influenza A virus (A/Shanghai/02/2013(H7N9))
**Protein**	polymerase PB1

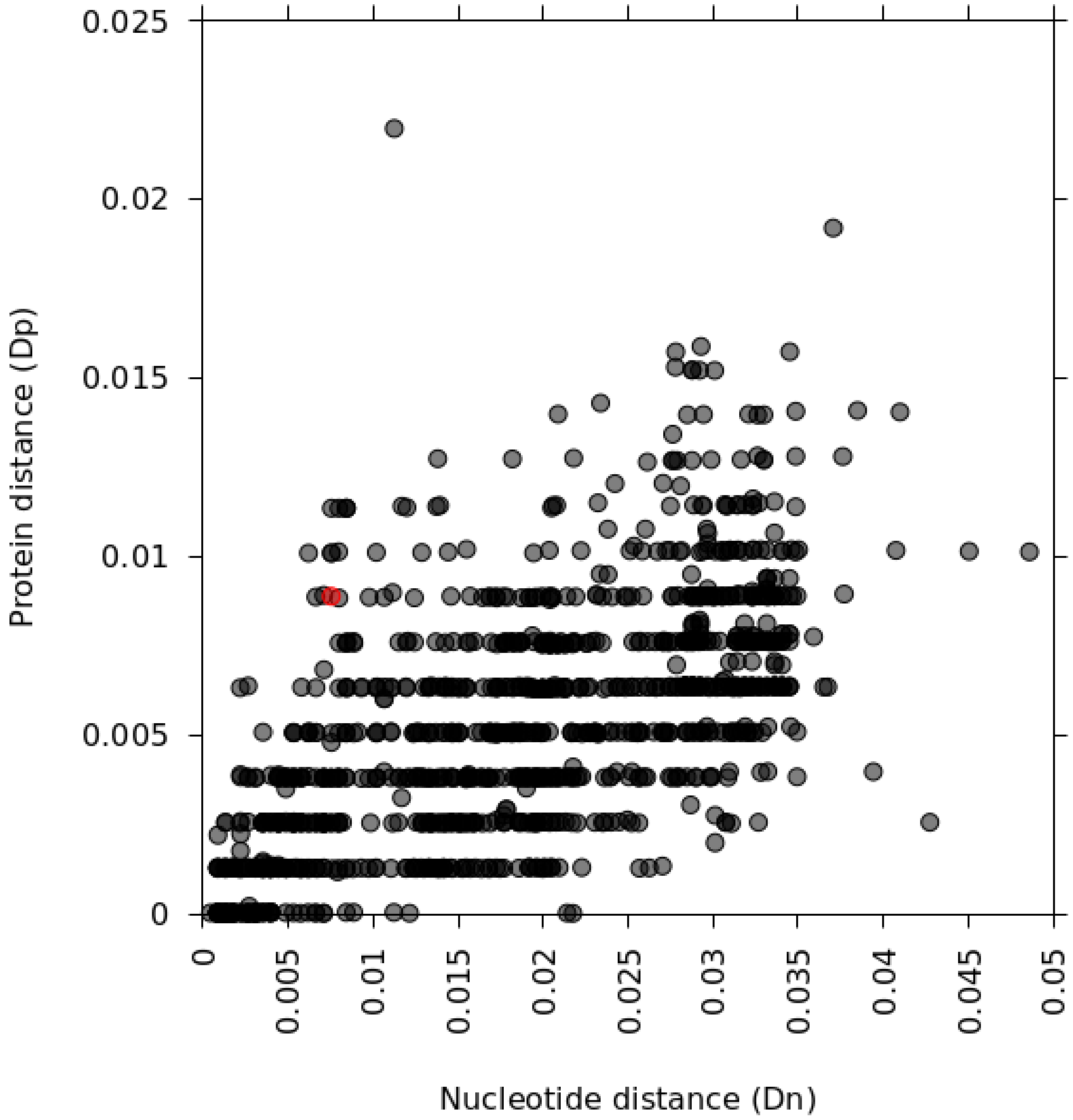

162. NC_026429.1/YP_009118481.1

**CDS cluster ID**	**162**
**CDS cluster name**	NC_026429.1/YP_009118481.1
**Total sequences**	493
**Reference forms**	492
**Compensatory frameshift forms**	1
**Virus**	Influenza A virus (A/Shanghai/02/2013(H7N9))
**Protein**	neuraminidase

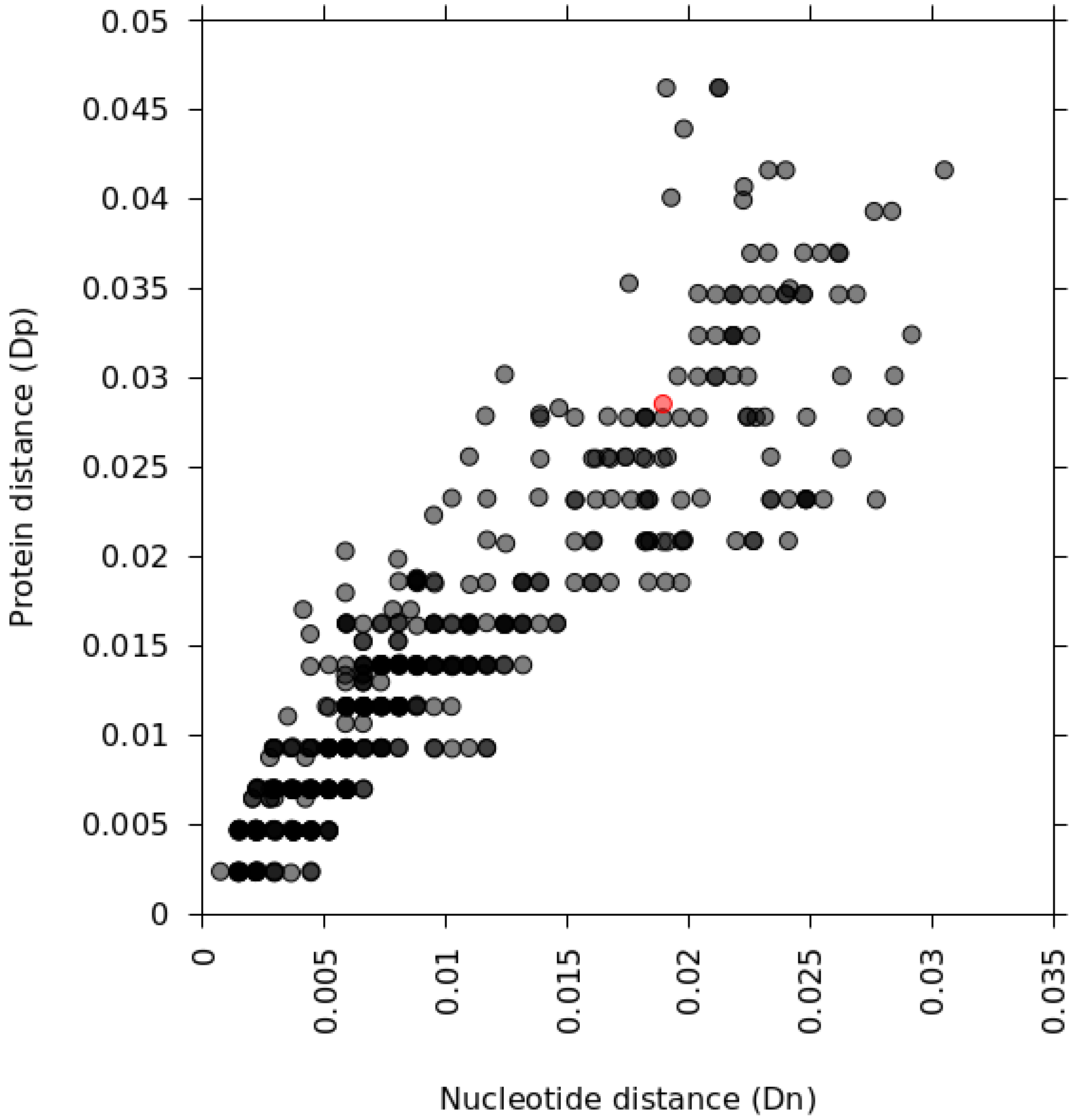

163. NC_026433.1/YP_009118626.1

**CDS cluster ID**	**163**
**CDS cluster name**	NC_026433.1/YP_009118626.1
**Total sequences**	5606
**Reference forms**	5604
**Compensatory frameshift forms**	1
**Virus**	Influenza A virus (A/California/07/2009(H1N1))
**Protein**	hemagglutinin

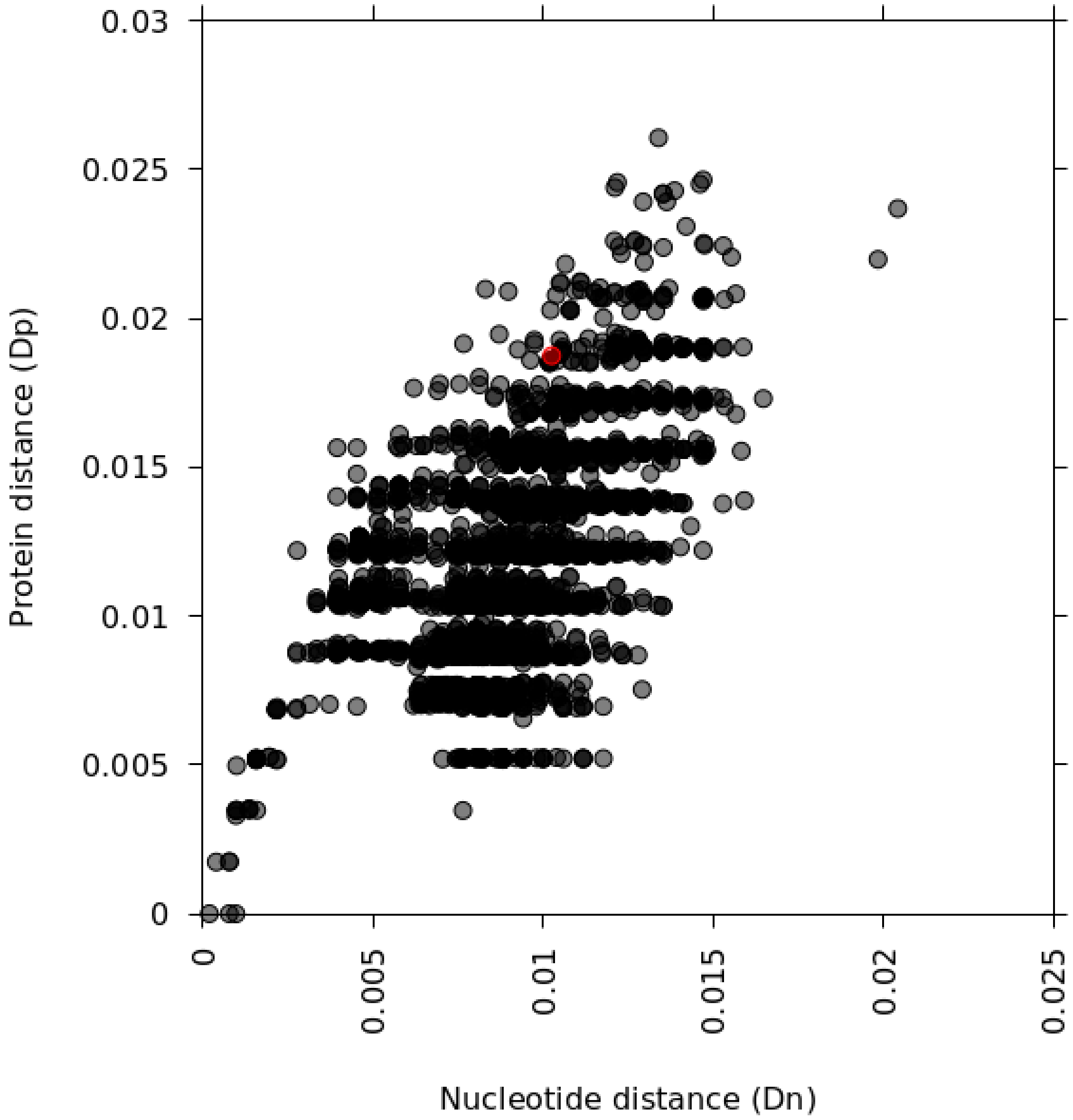

164. NC_026434.1/YP_009118627.1

**CDS cluster ID**	**164**
**CDS cluster name**	NC_026434.1/YP_009118627.1
**Total sequences**	4444
**Reference forms**	4442
**Compensatory frameshift forms**	1
**Virus**	Influenza A virus (A/California/07/2009(H1N1))
**Protein**	neuraminidase

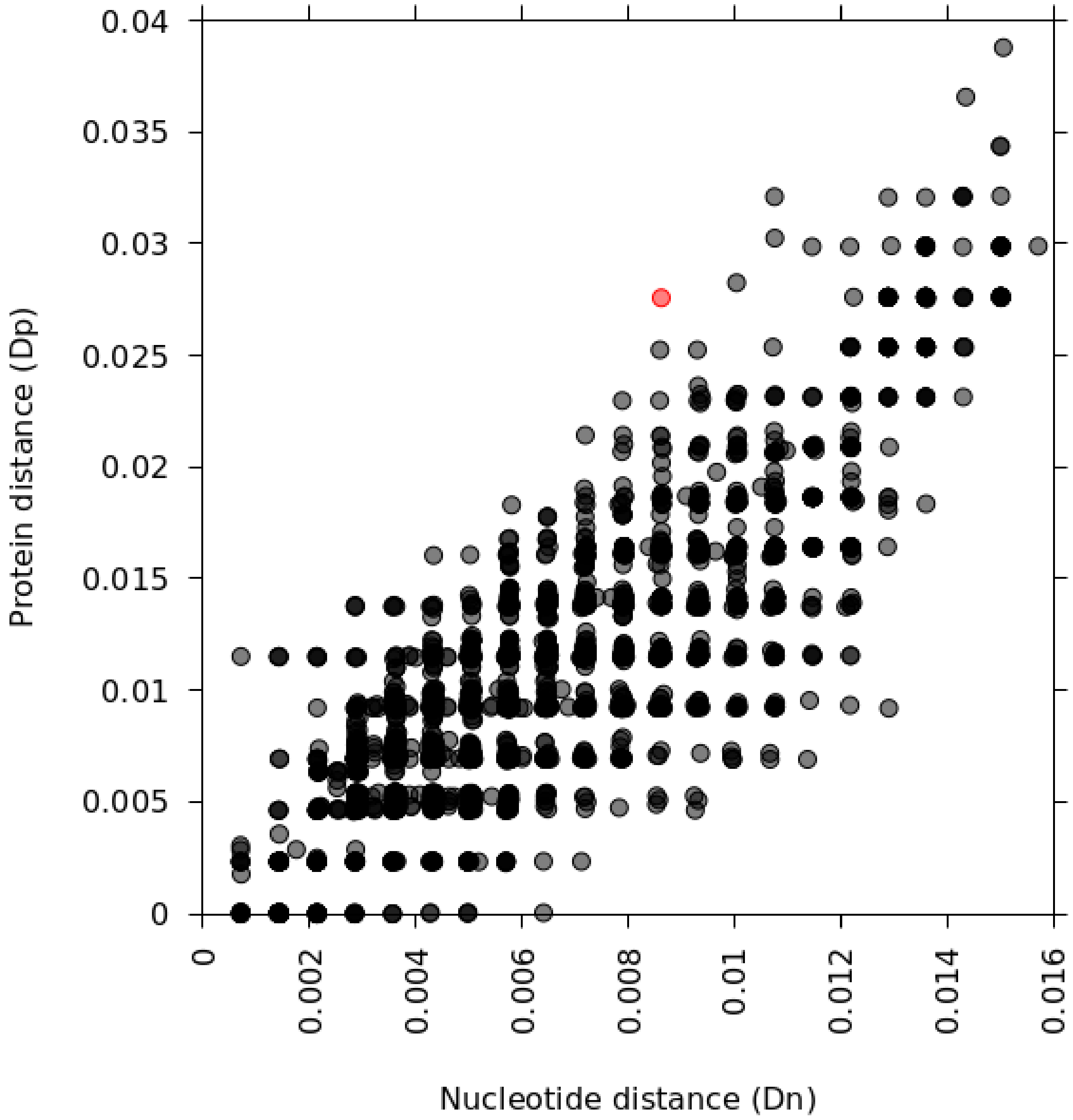

165. NC_027719.1/YP_009162643.1

**CDS cluster ID**	**165**
**CDS cluster name**	NC_027719.1/YP_009162643.1
**Total sequences**	5
**Reference forms**	4
**Compensatory frameshift forms**	1
**Virus**	Chrysanthemum stem necrosis virus (CSNV)
**Protein**	nonstructural protein

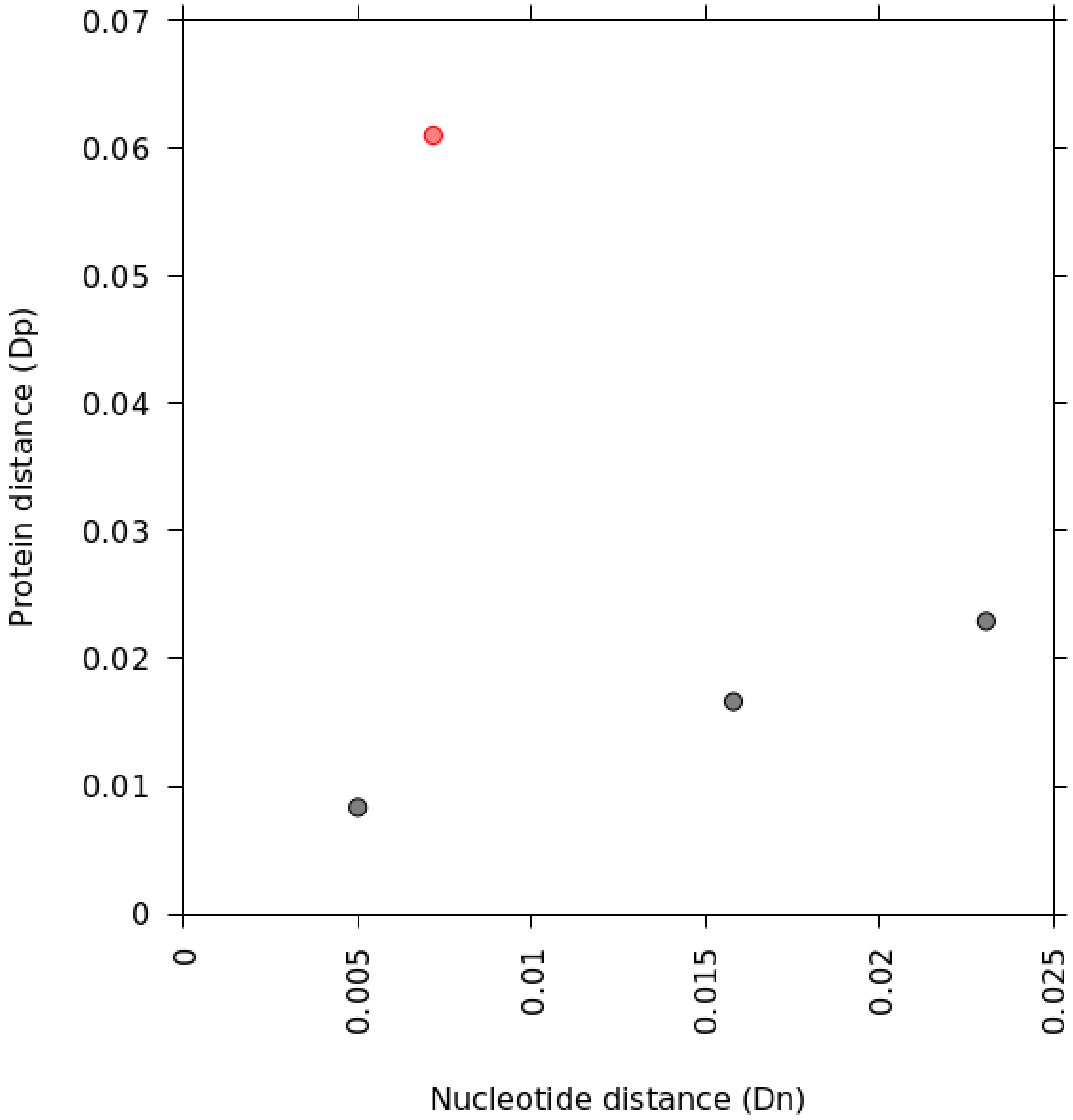

171. NC_035483.1/YP_009408637.1

**CDS cluster ID**	**171**
**CDS cluster name**	NC_035483.1/YP_009408637.1
**Total sequences**	4
**Reference forms**	3
**Compensatory frameshift forms**	1
**Virus**	Tomato chlorotic spot virus
**Protein**	RNA-dependent RNA polymerase

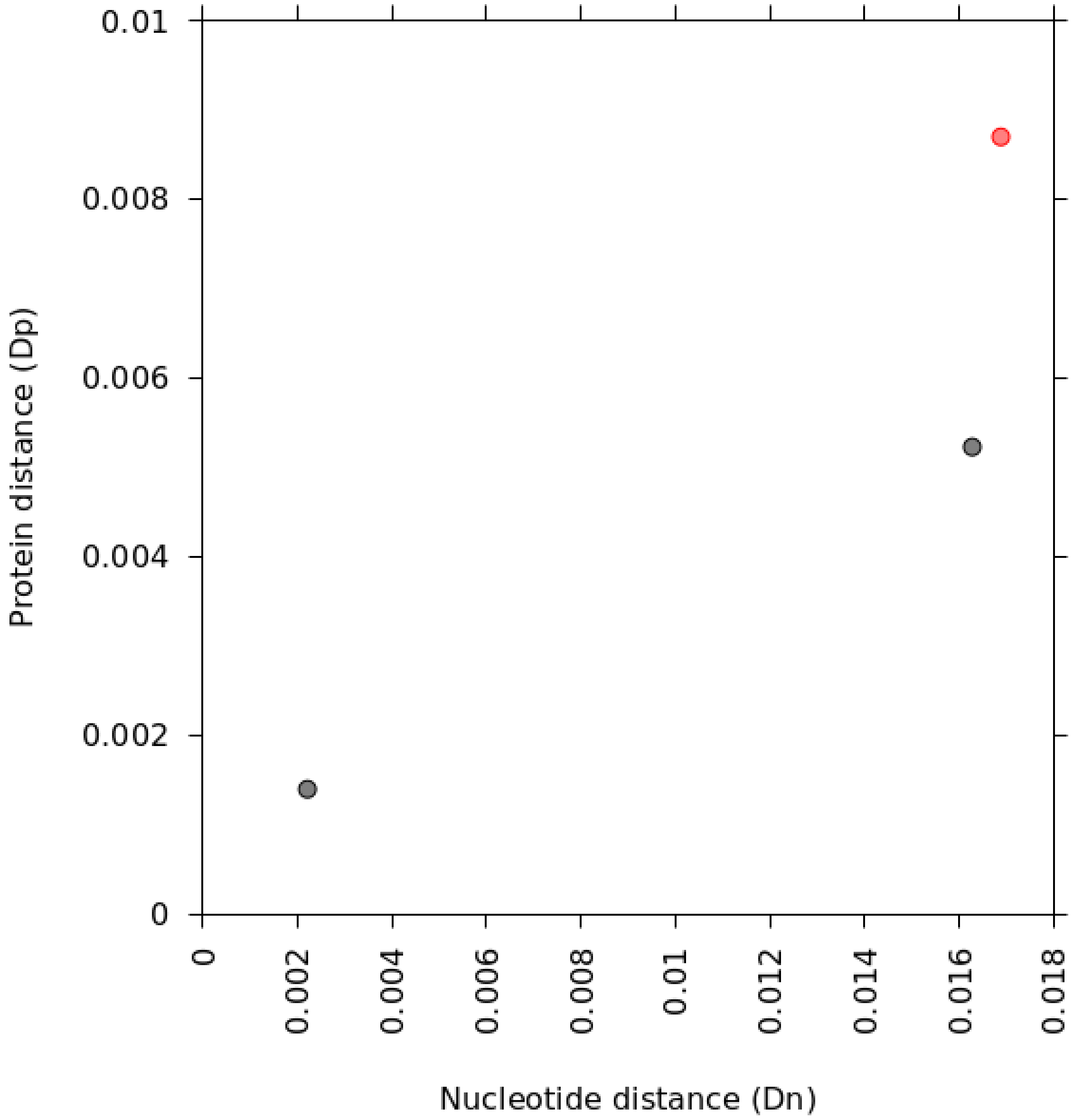

172. NC_035889.1/YP_009428568.1

**CDS cluster ID**	**172**
**CDS cluster name**	NC_035889.1/YP_009428568.1
**Total sequences**	415
**Reference forms**	414
**Compensatory frameshift forms**	1
**Virus**	Zika virus
**Protein**	polyprotein

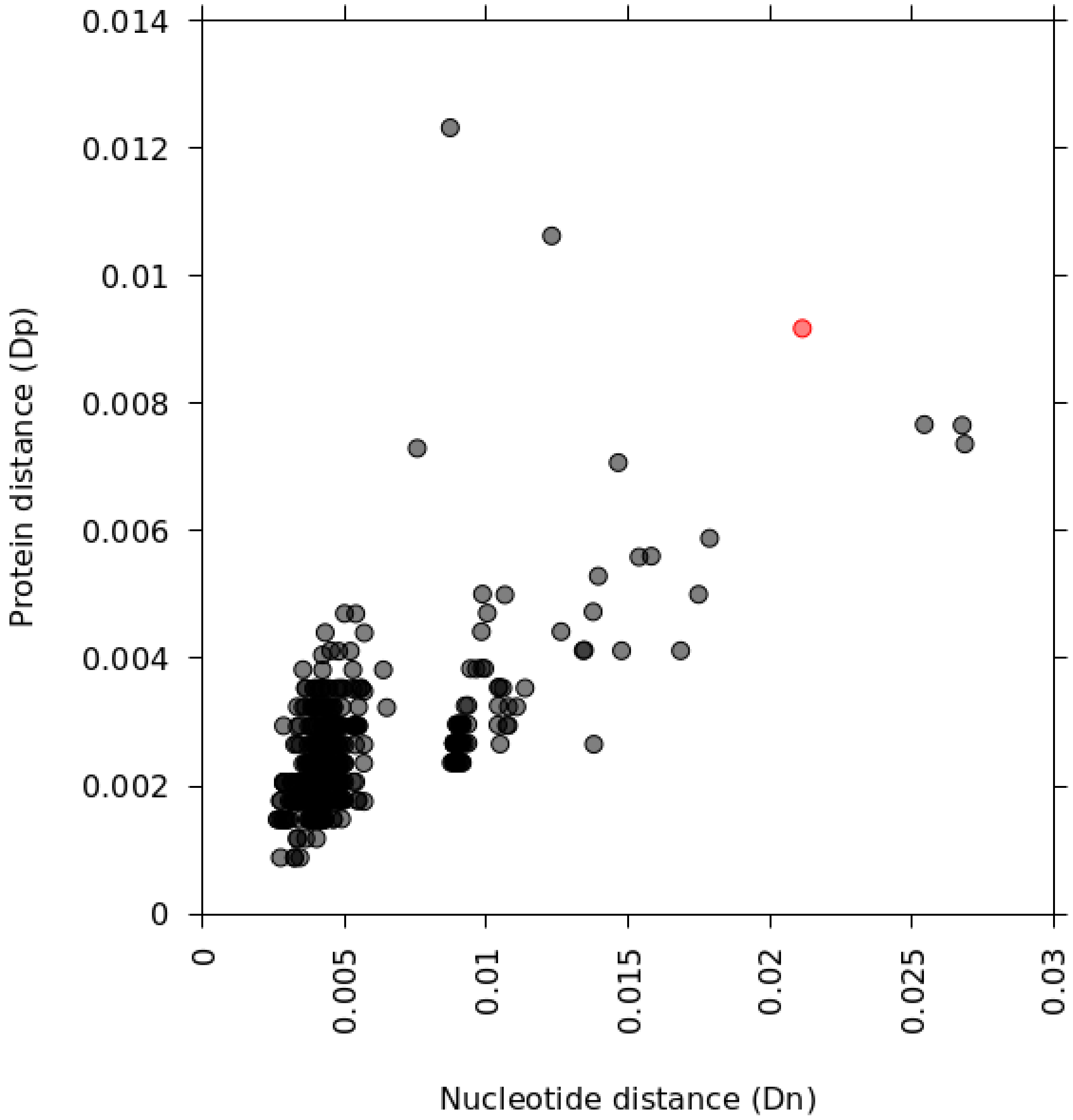

173. NC_036475.1/YP_009445961.1

**CDS cluster ID**	**173**
**CDS cluster name**	NC_036475.1/YP_009445961.1
**Total sequences**	19
**Reference forms**	18
**Compensatory frameshift forms**	1
**Virus**	Piscine orthoreovirus
**Protein**	outer fiber protein

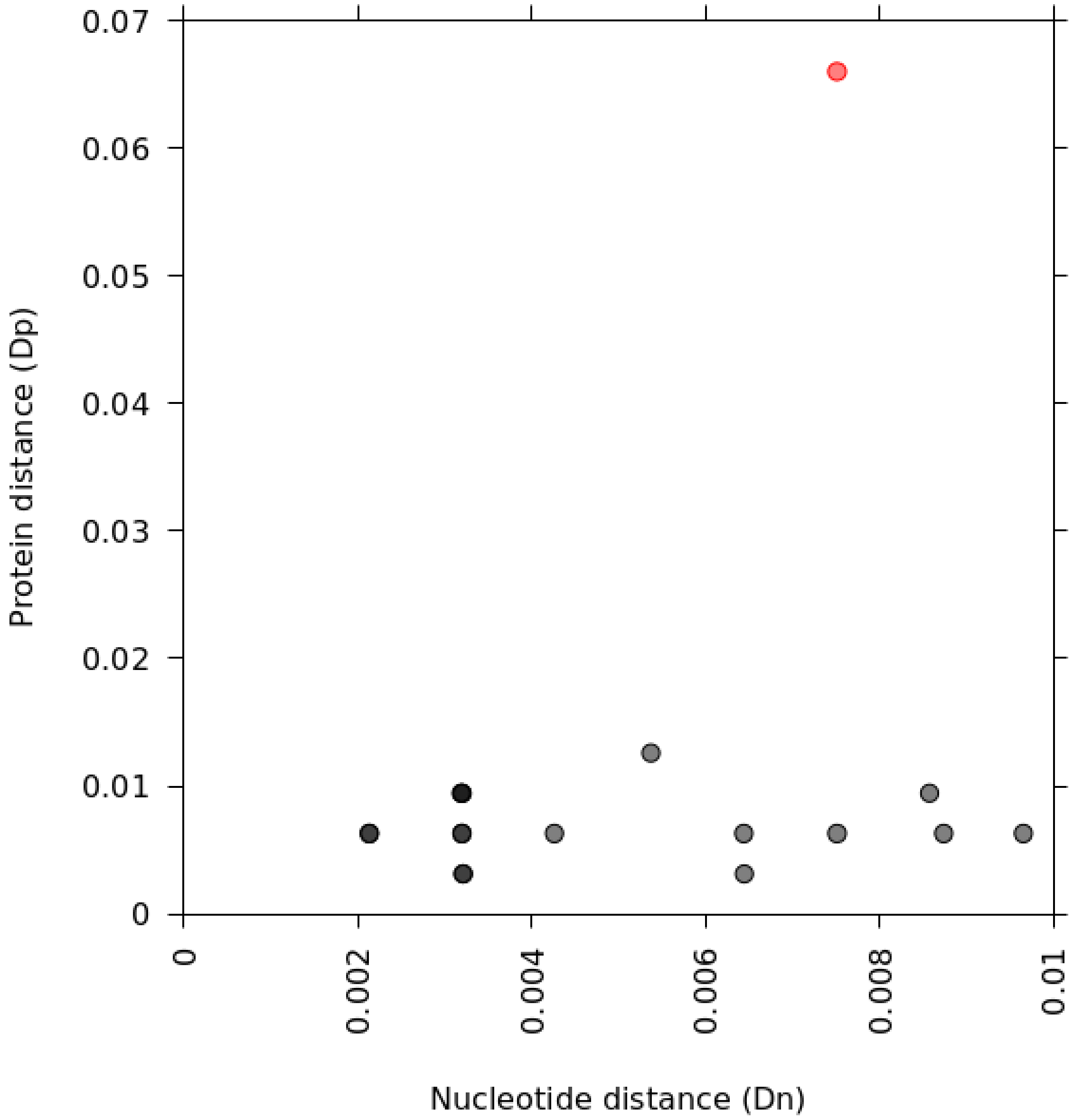

174. NC_037577.1/YP_009480345.1

CDS cluster ID	**174**
CDS cluster name	NC_037577.1/YP_009480345.1
Total sequences	16
Reference forms	15
Compensatory frameshift forms	1
Virus	Maize rough dwarf virus
Protein	major outer structural protein

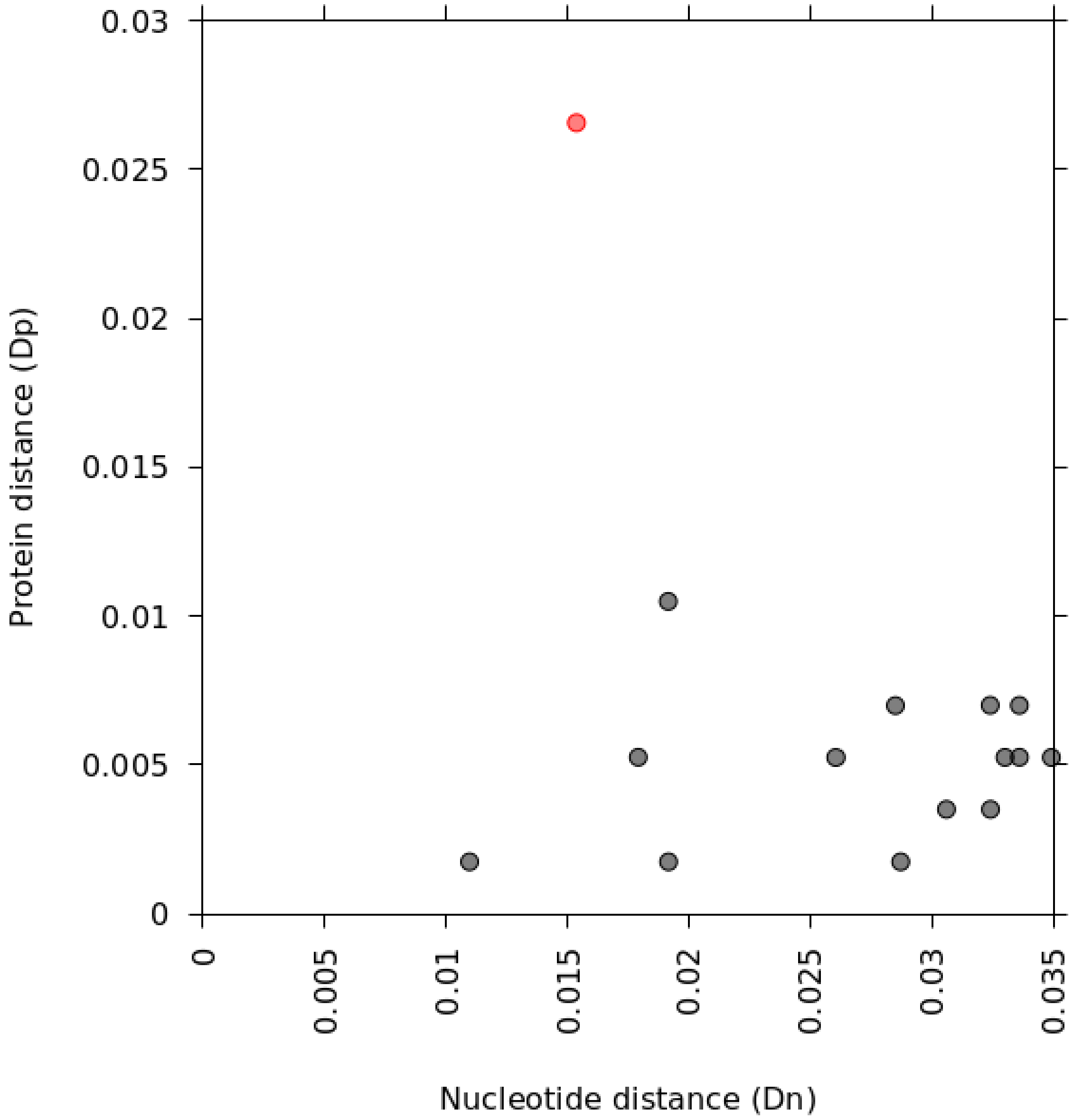

175. NC_038281.1/YP_009505498.1

CDS cluster ID	175
CDS cluster name	NC_038281.1/YP_009505498.1
Total sequences	4
Reference forms	3
Compensatory frameshift forms	1
Virus	Drosophila melanogaster sigmavirus HAP23
Protein	nucleocapsid protein

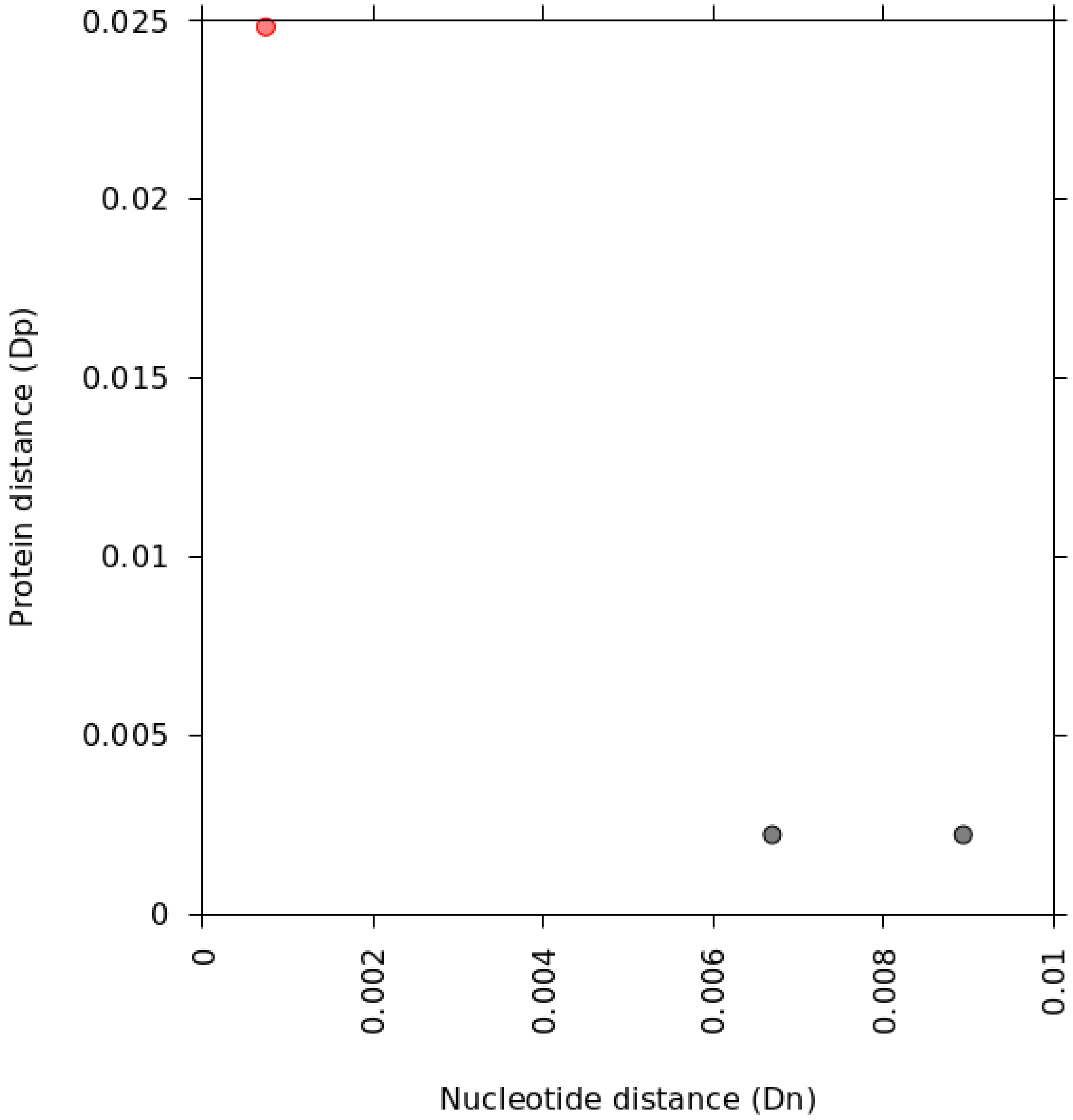

176. NC_038291.1/YP_009505547.1

**CDS cluster ID**	**176**
**CDS cluster name**	NC_038291.1/YP_009505547.1
**Total sequences**	74
**Reference forms**	73
**Compensatory frameshift forms**	1
**Virus**	Porcine reproductive and respiratory syndrome virus 2
**Protein**	1a replicase

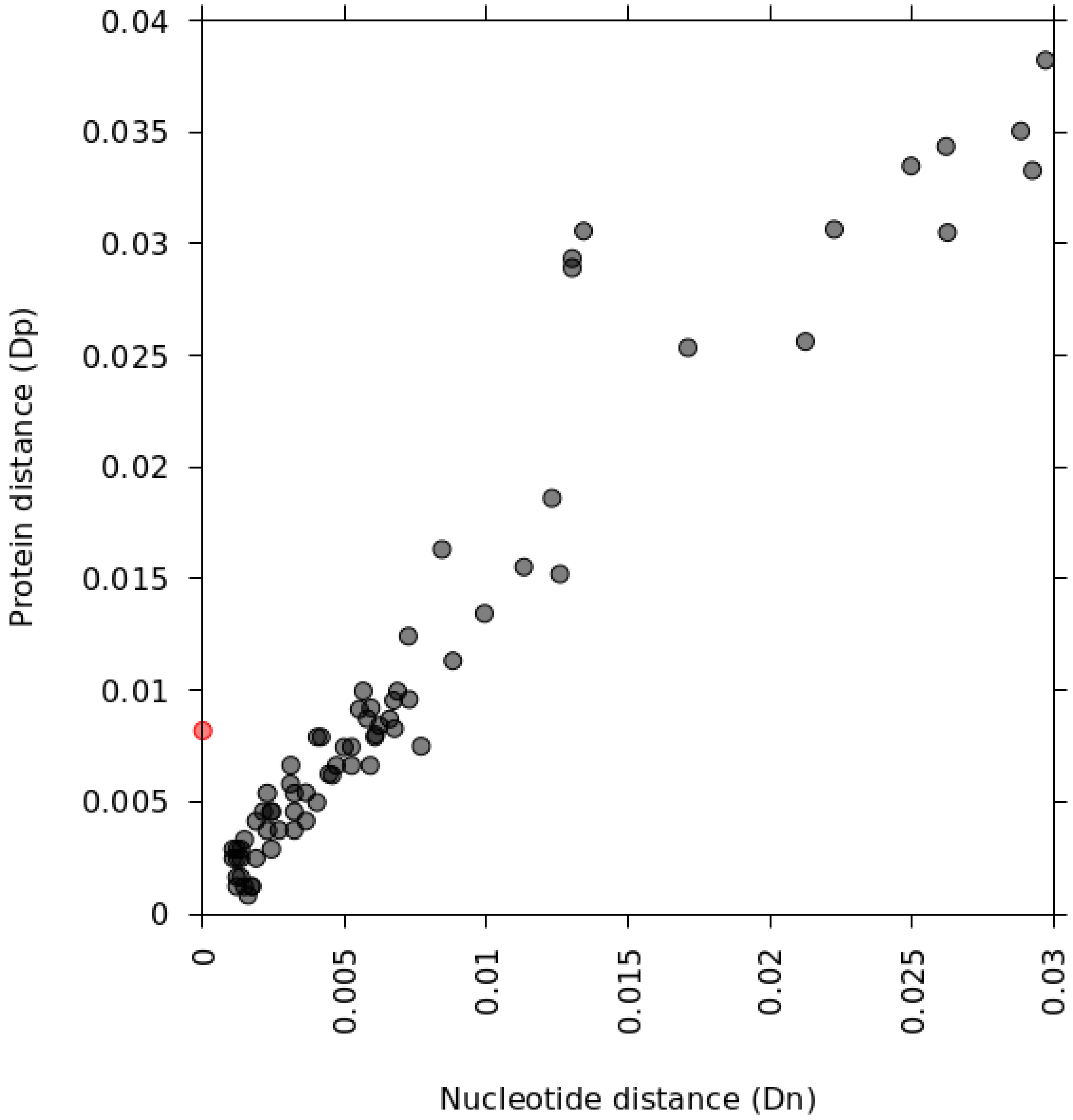

177. NC_038291.1/YP_009505551.1

CDS cluster ID	177
CDS cluster name	NC_038291.1/YP_009505551.1
Total sequences	82
Reference forms	81
Compensatory frameshift forms	1
Virus	Porcine reproductive and respiratory syndrome virus 2
Protein	GP3 envelope protein

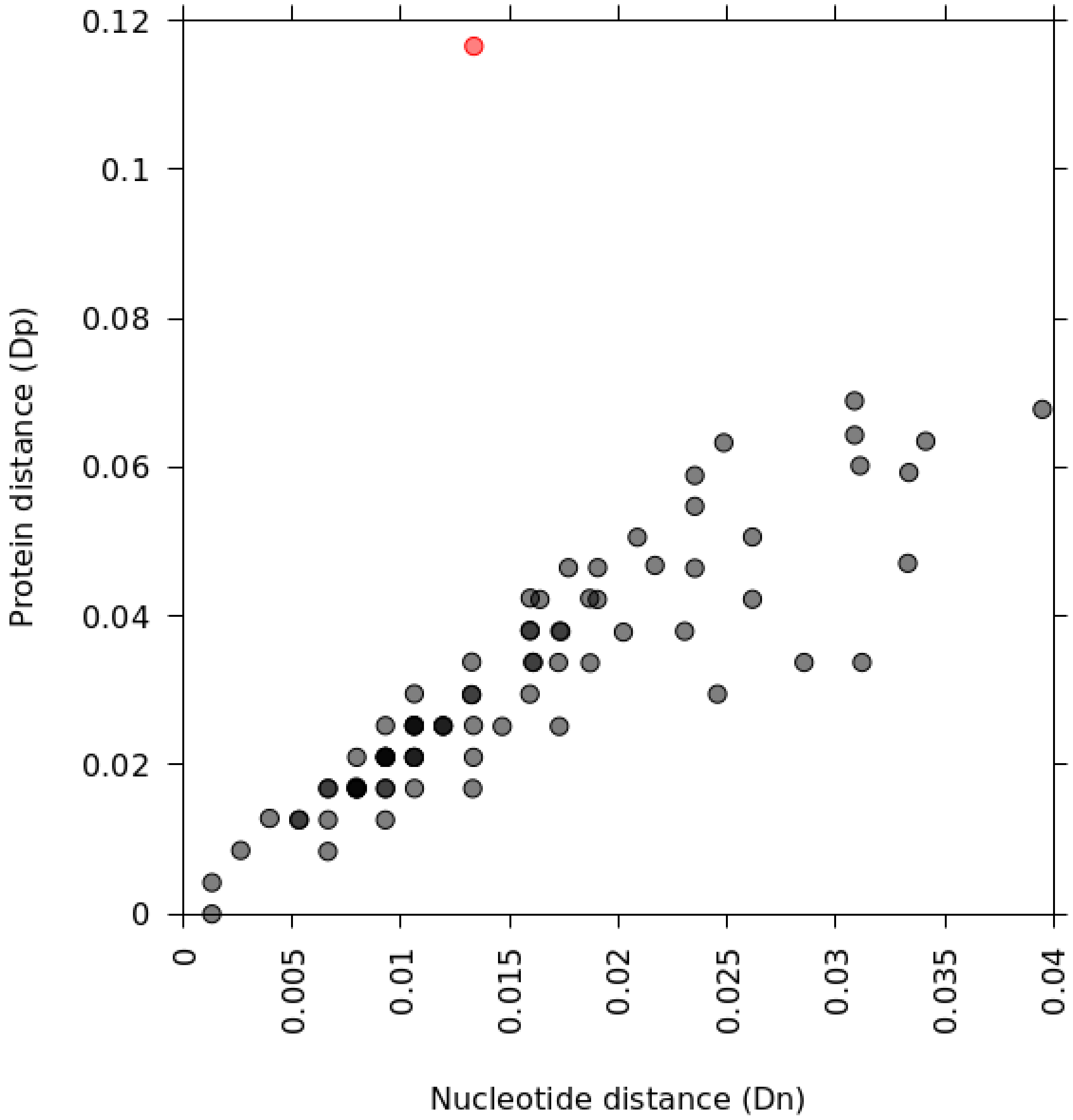

178. NC_038294.1/YP_007188577.3

**CDS cluster ID**	**178**
**CDS cluster name**	NC_038294.1/YP_007188577.3
**Total sequences**	413
**Reference forms**	410
**Compensatory frameshift forms**	1
**Virus**	Betacoronavirus England 1
**Protein**	ORF1b protein

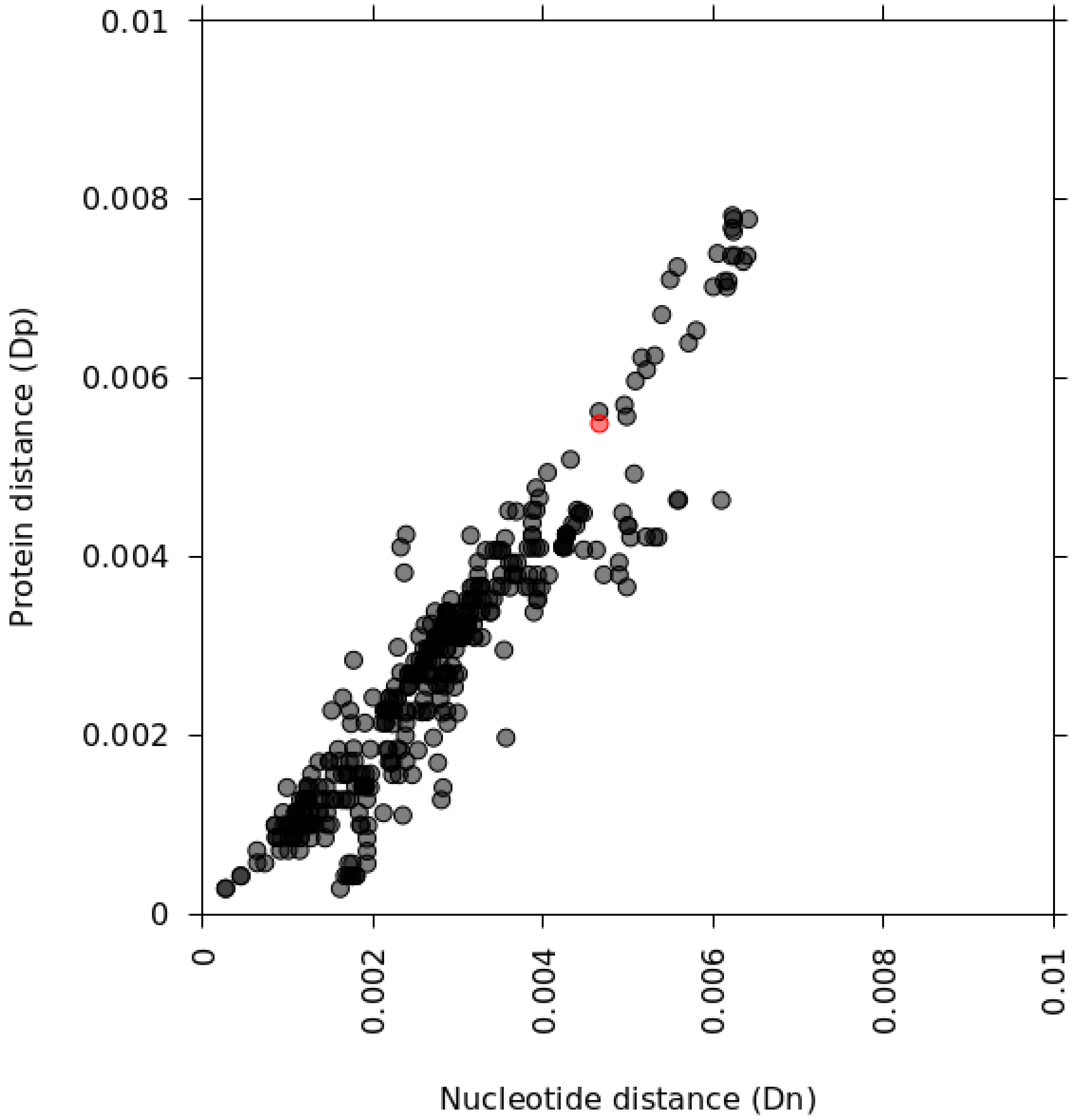

179. NC_038307.1/YP_009505604.1

**CDS cluster ID**	**179**
**CDS cluster name**	NC_038307.1/YP_009505604.1
**Total sequences**	16
**Reference forms**	15
**Compensatory frameshift forms**	1
**Virus**	Coxsackievirus B3
**Protein**	polyprotein

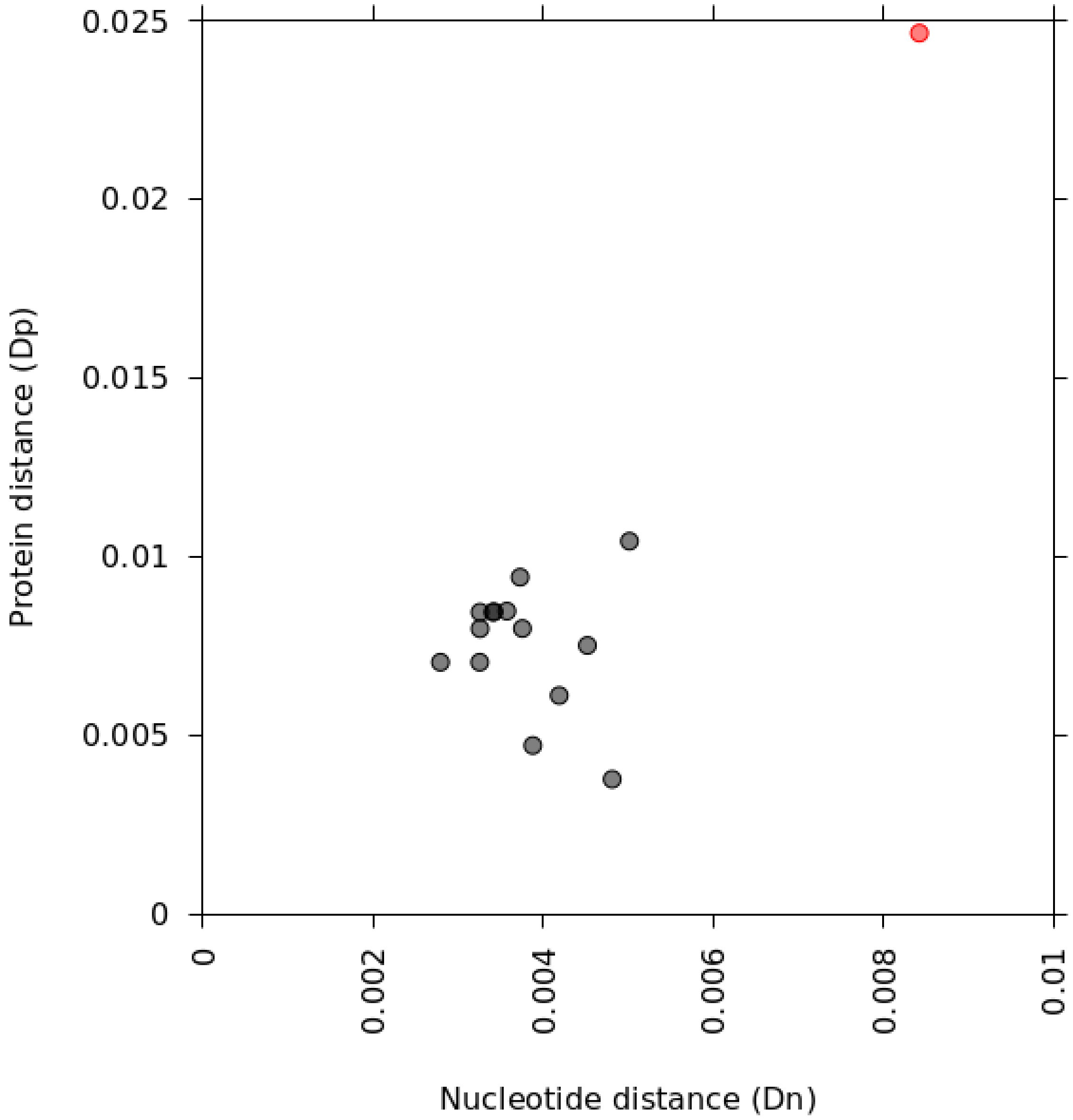

184. NC_038672.1/YP_009507799.1

**CDS cluster ID**	**184**
**CDS cluster name**	NC_038672.1/YP_009507799.1
**Total sequences**	3
**Reference forms**	2
**Compensatory frameshift forms**	1
**Virus**	Mucambo virus
**Protein**	structural polyprotein

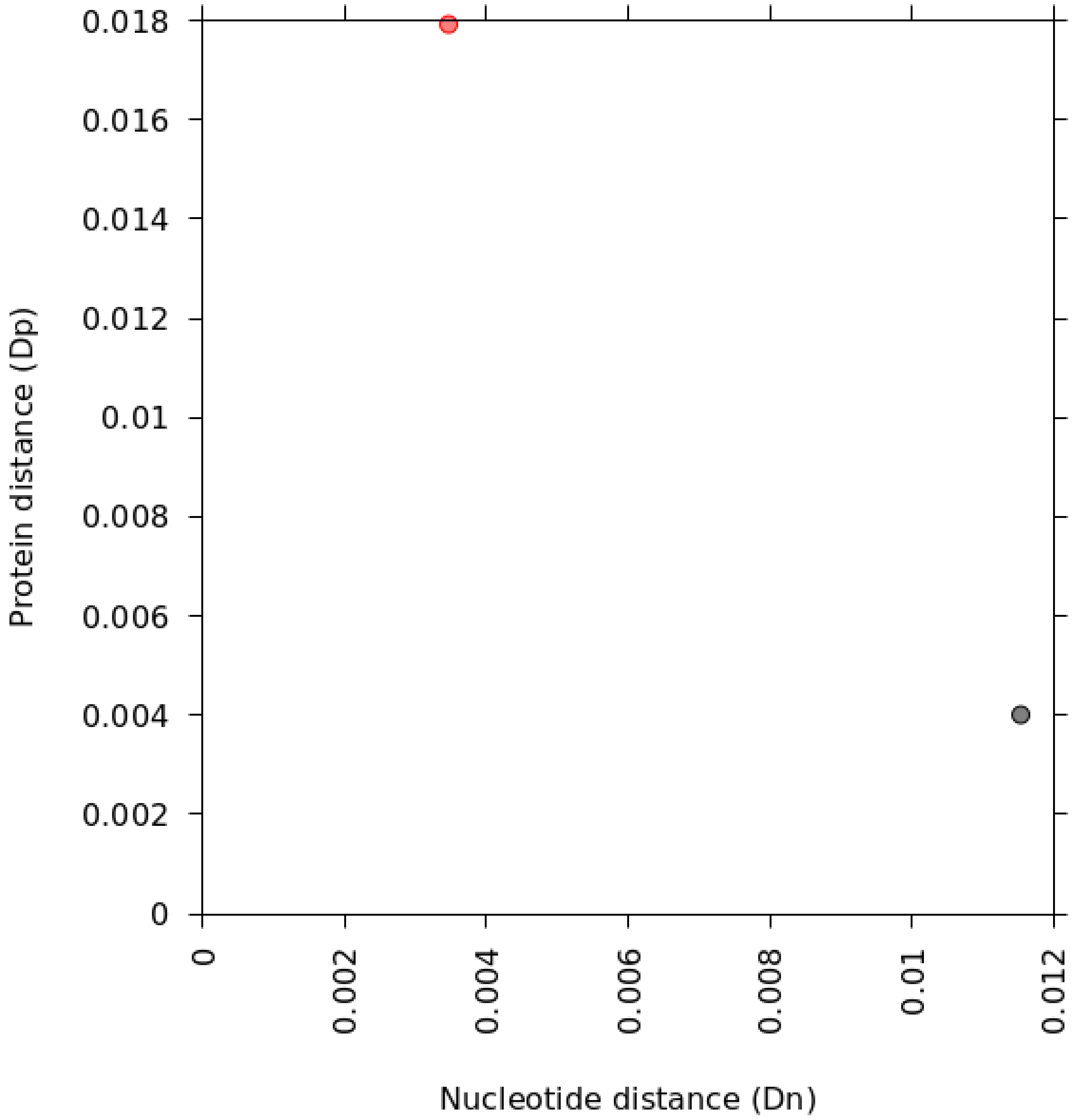

185. NC_038695.1/YP_009507827.1

**CDS cluster ID**	**185**
**CDS cluster name**	NC_038695.1/YP_009507827.1
**Total sequences**	3
**Reference forms**	2
**Compensatory frameshift forms**	1
**Virus**	Rockport virus
**Protein**	RNA-dependent RNA polymerase

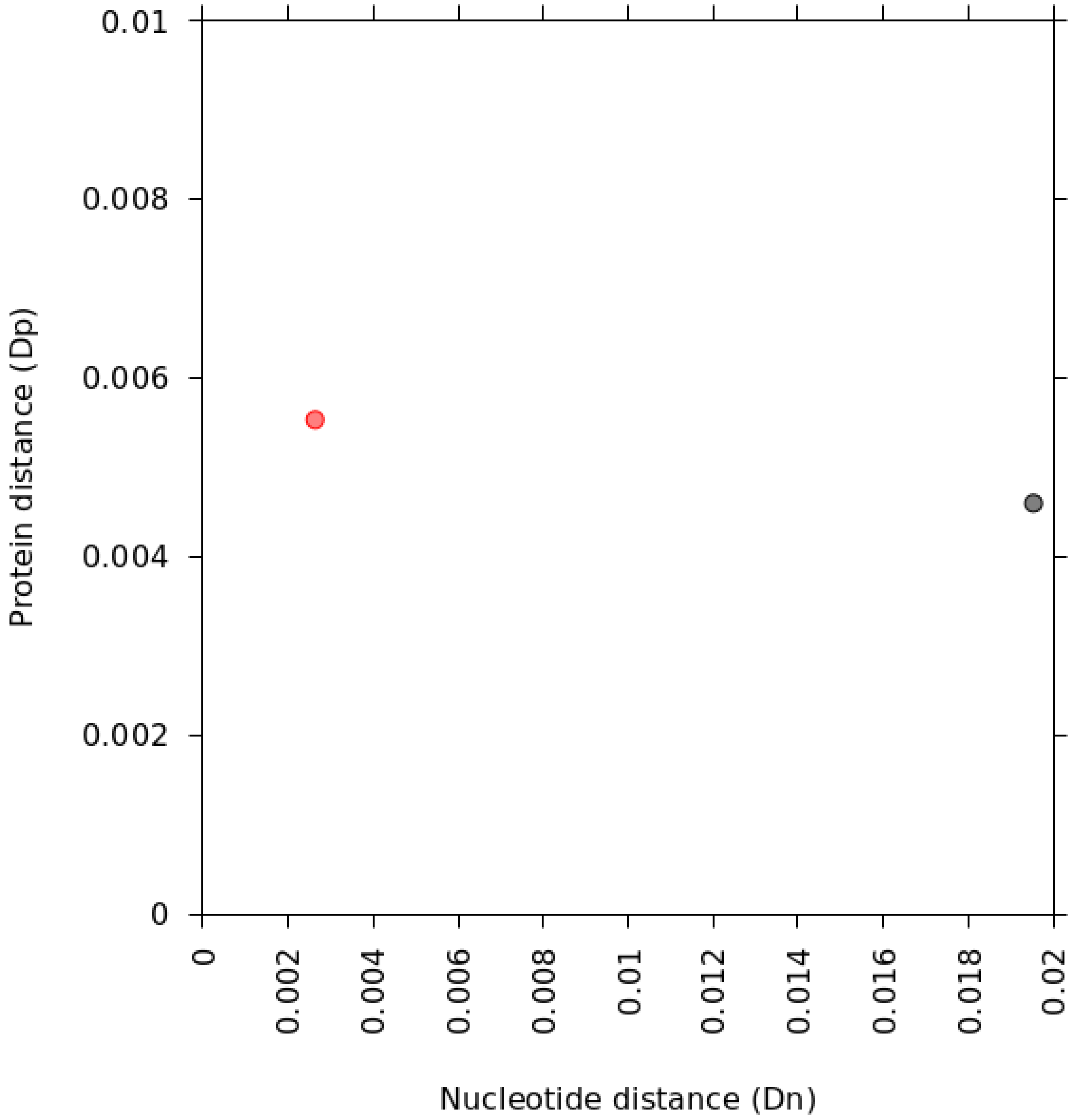

186. NC_038715.1/YP_009507856.1

**CDS cluster ID**	**186**
**CDS cluster name**	NC_038715.1/YP_009507856.1
**Total sequences**	4
**Reference forms**	3
**Compensatory frameshift forms**	1
**Virus**	Alajuela virus
**Protein**	polyprotein

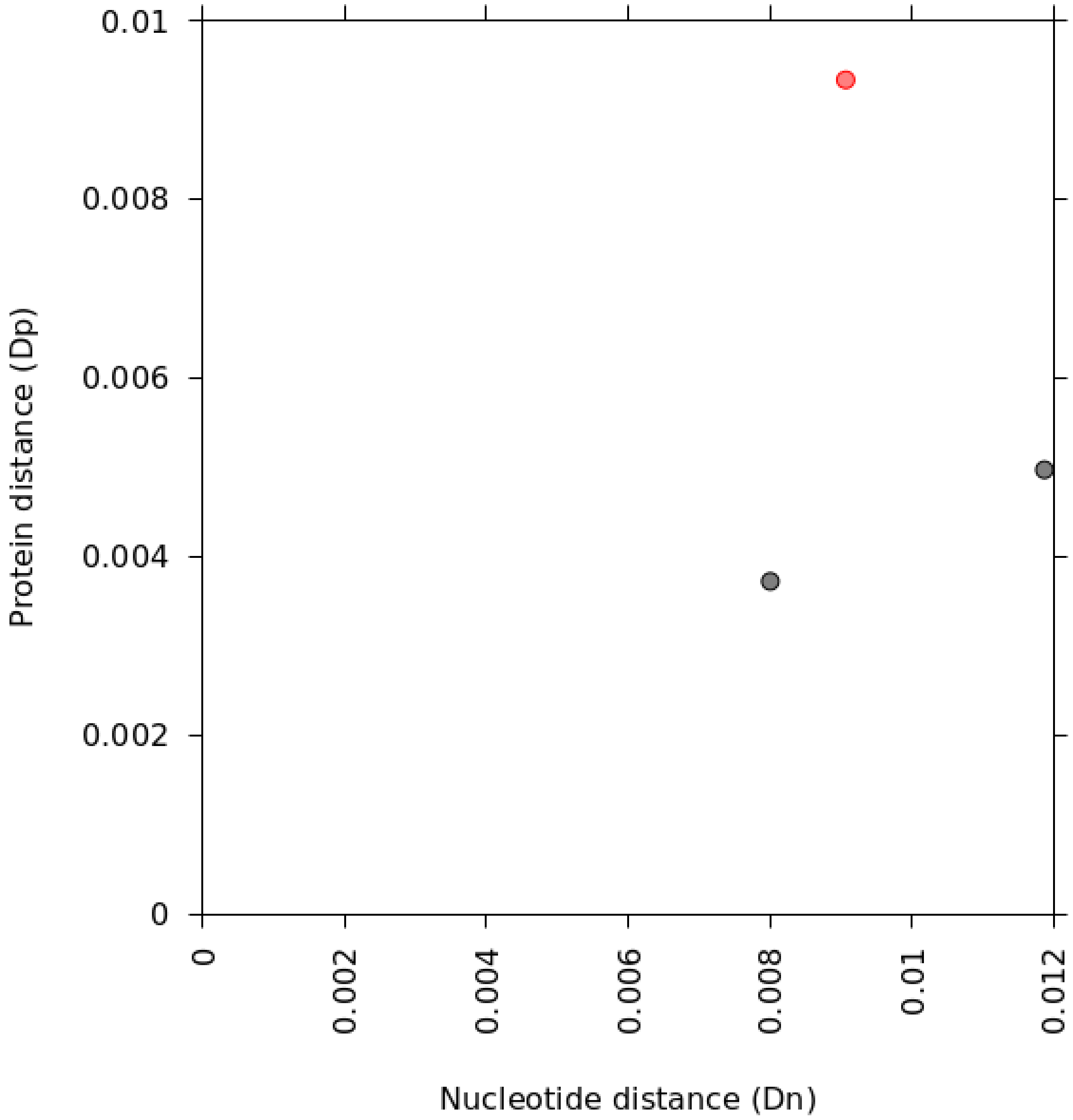

188. NC_039208.1/YP_009513020.1

**CDS cluster ID**	**188**
**CDS cluster name**	NC_039208.1/YP_009513020.1
**Total sequences**	107
**Reference forms**	106
**Compensatory frameshift forms**	1
**Virus**	Porcine coronavirus HKU15
**Protein**	replicase polyprotein

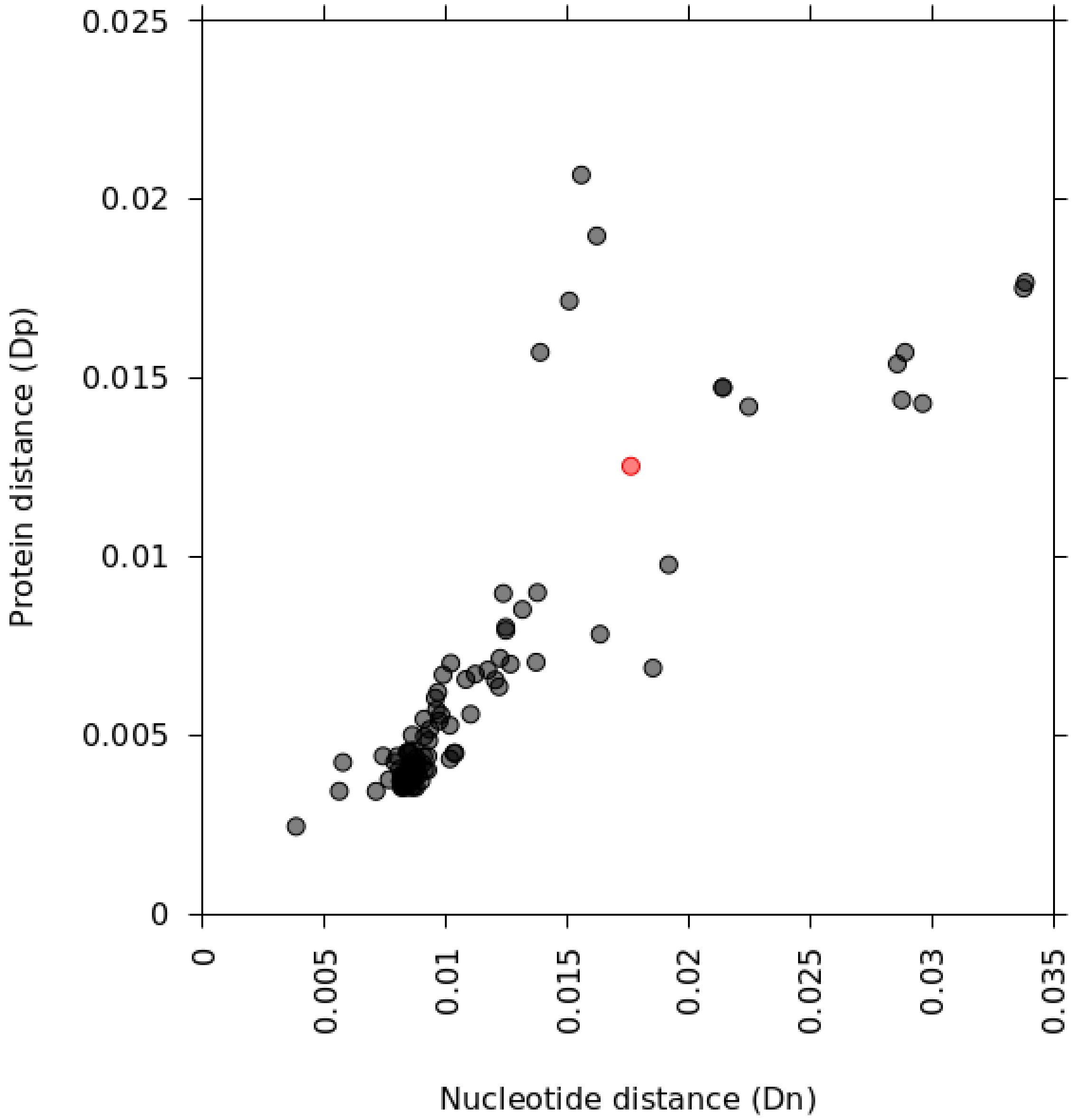

189. NC_039226.1/YP_009513207.1

**CDS cluster ID**	**189**
**CDS cluster name**	NC_039226.1/YP_009513207.1
**Total sequences**	3
**Reference forms**	2
**Compensatory frameshift forms**	1
**Virus**	Beet soil-borne mosaic virus (BSBMV)
**Protein**	polyprotein

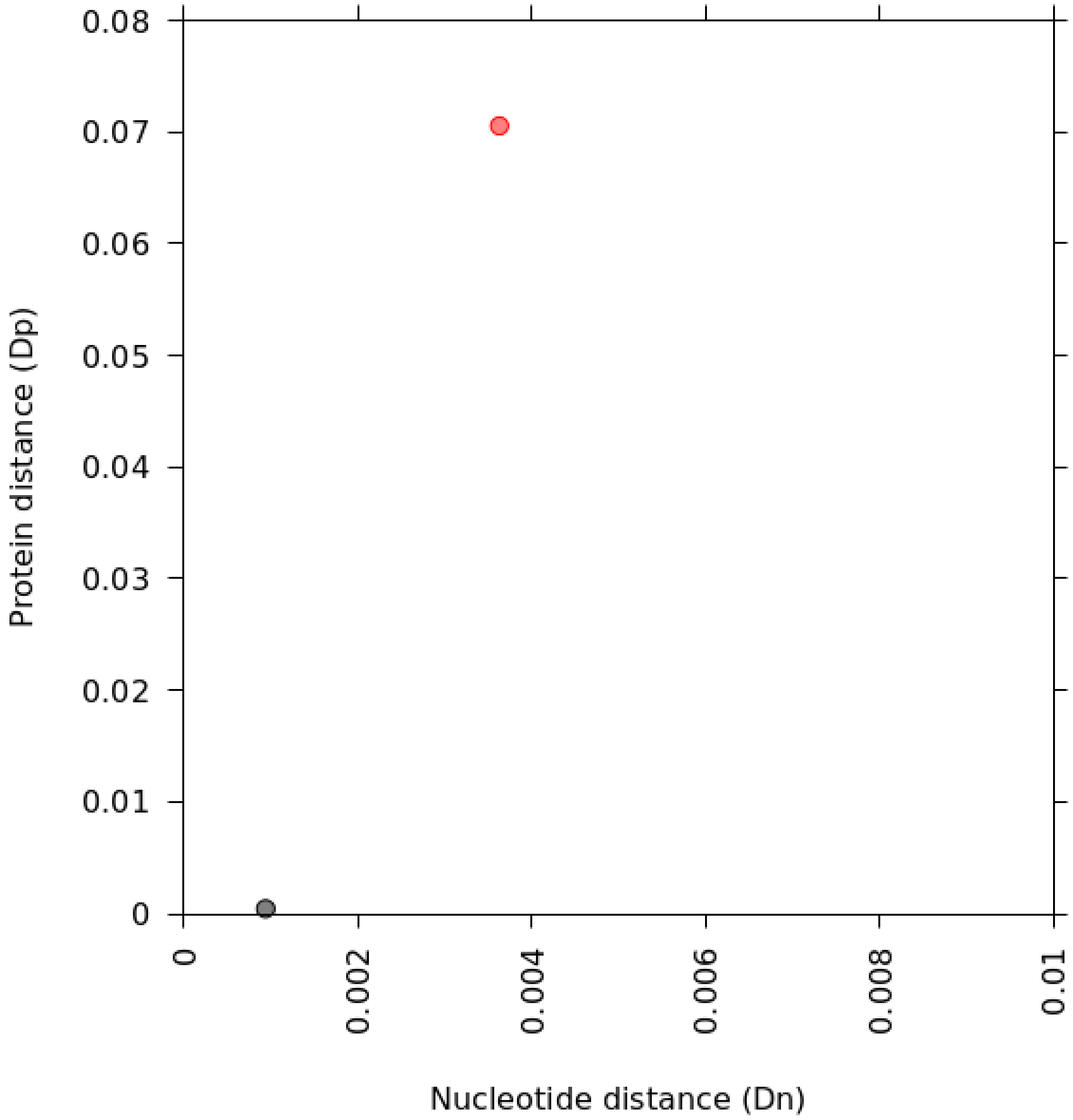

190. NC_039476.1/YP_009518838.1

**CDS cluster ID**	**190**
**CDS cluster name**	NC_039476.1/YP_009518838.1
**Total sequences**	178
**Reference forms**	177
**Compensatory frameshift forms**	1
**Virus**	Norovirus GII.2
**Protein**	nonstructural polyprotein

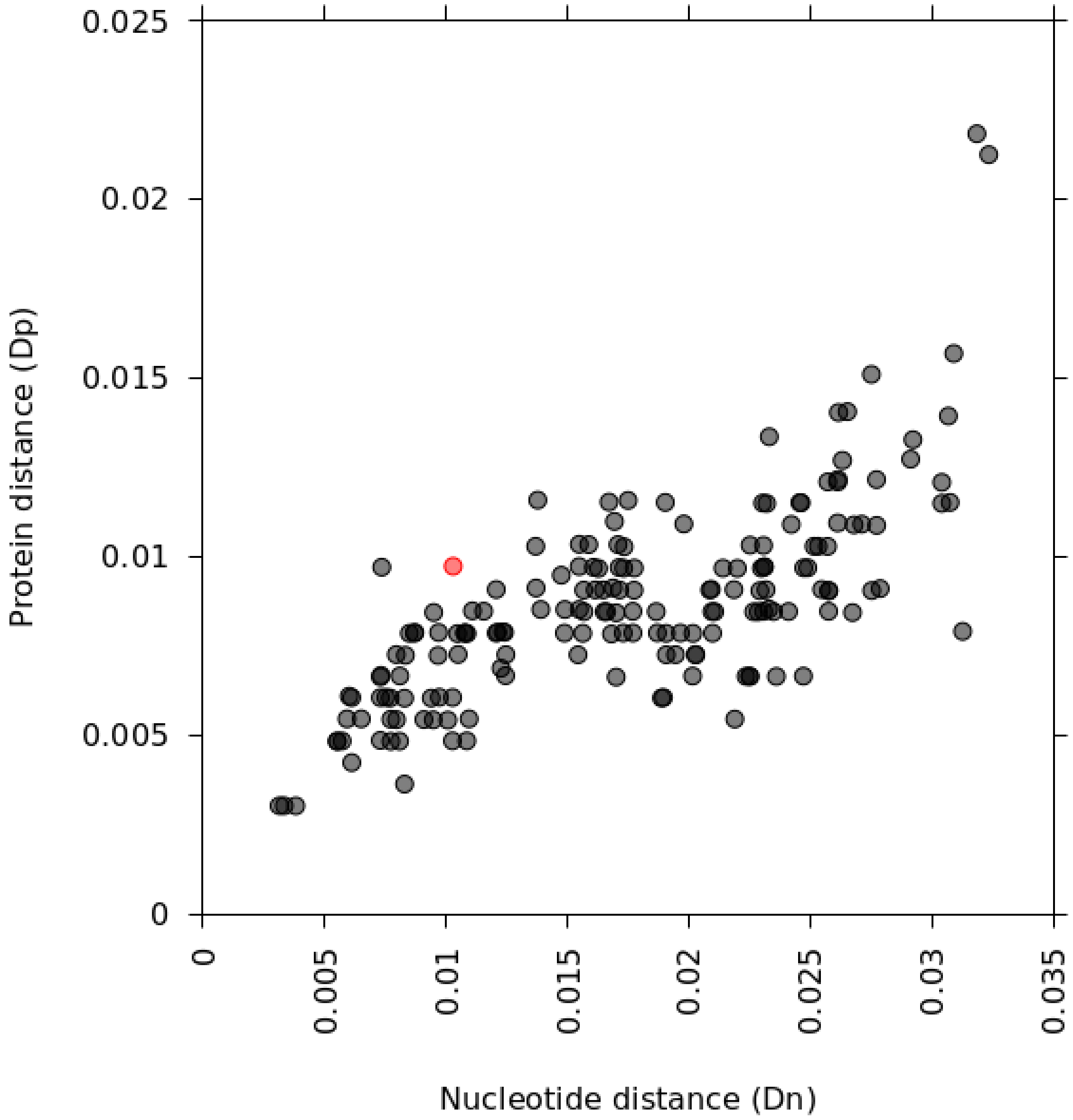

191. NC_039476.1/YP_009518839.1

**CDS cluster ID**	**191**
**CDS cluster name**	NC_039476.1/YP_009518839.1
**Total sequences**	322
**Reference forms**	321
**Compensatory frameshift forms**	1
**Virus**	Norovirus GII.2
**Protein**	VP1

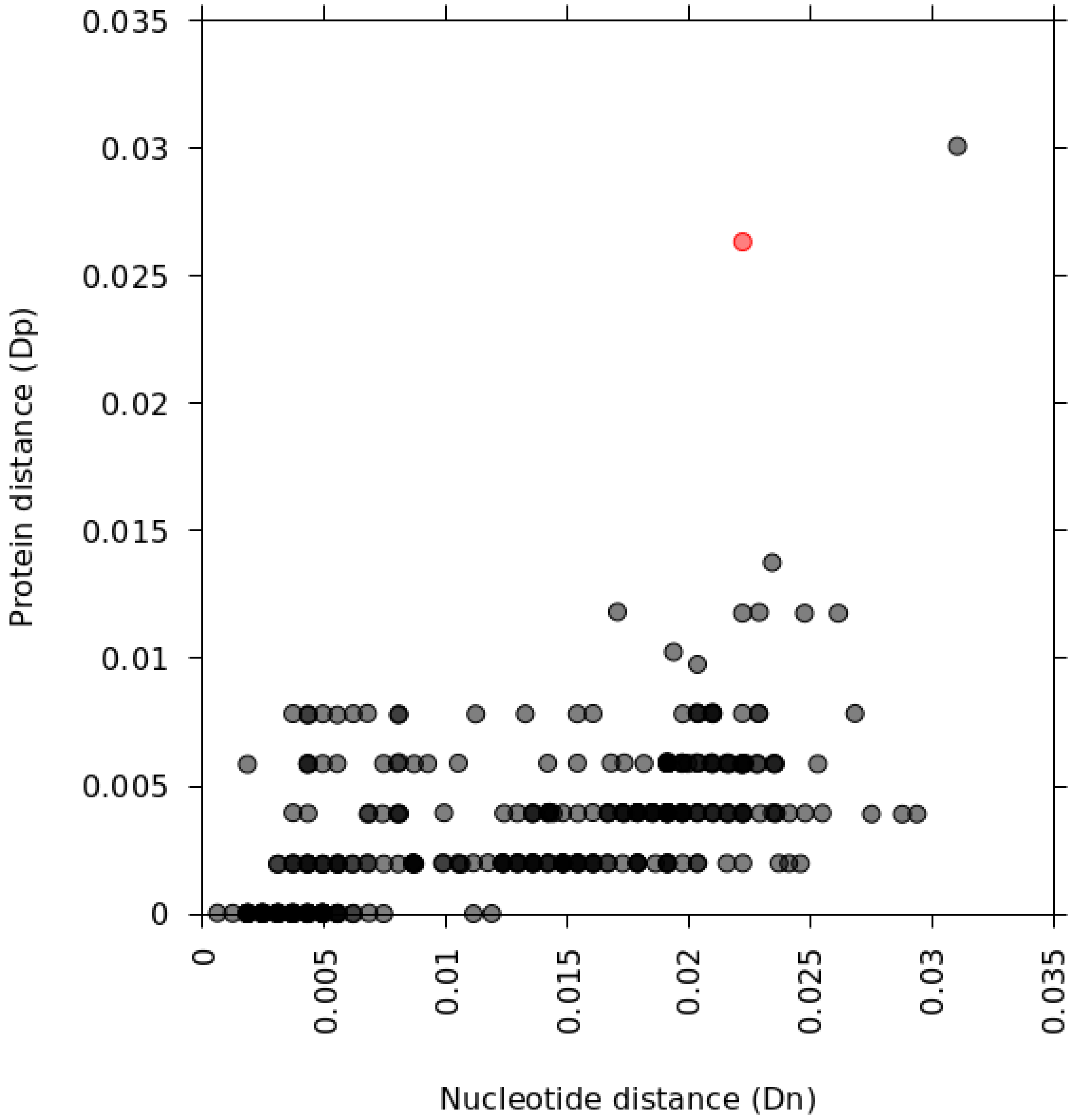

192. NC_039477.1/YP_009518841.1

**CDS cluster ID**	**192**
**CDS cluster name**	NC_039477.1/YP_009518841.1
**Total sequences**	178
**Reference forms**	177
**Compensatory frameshift forms**	1
**Virus**	Norovirus GII
**Protein**	nonstructural polyprotein

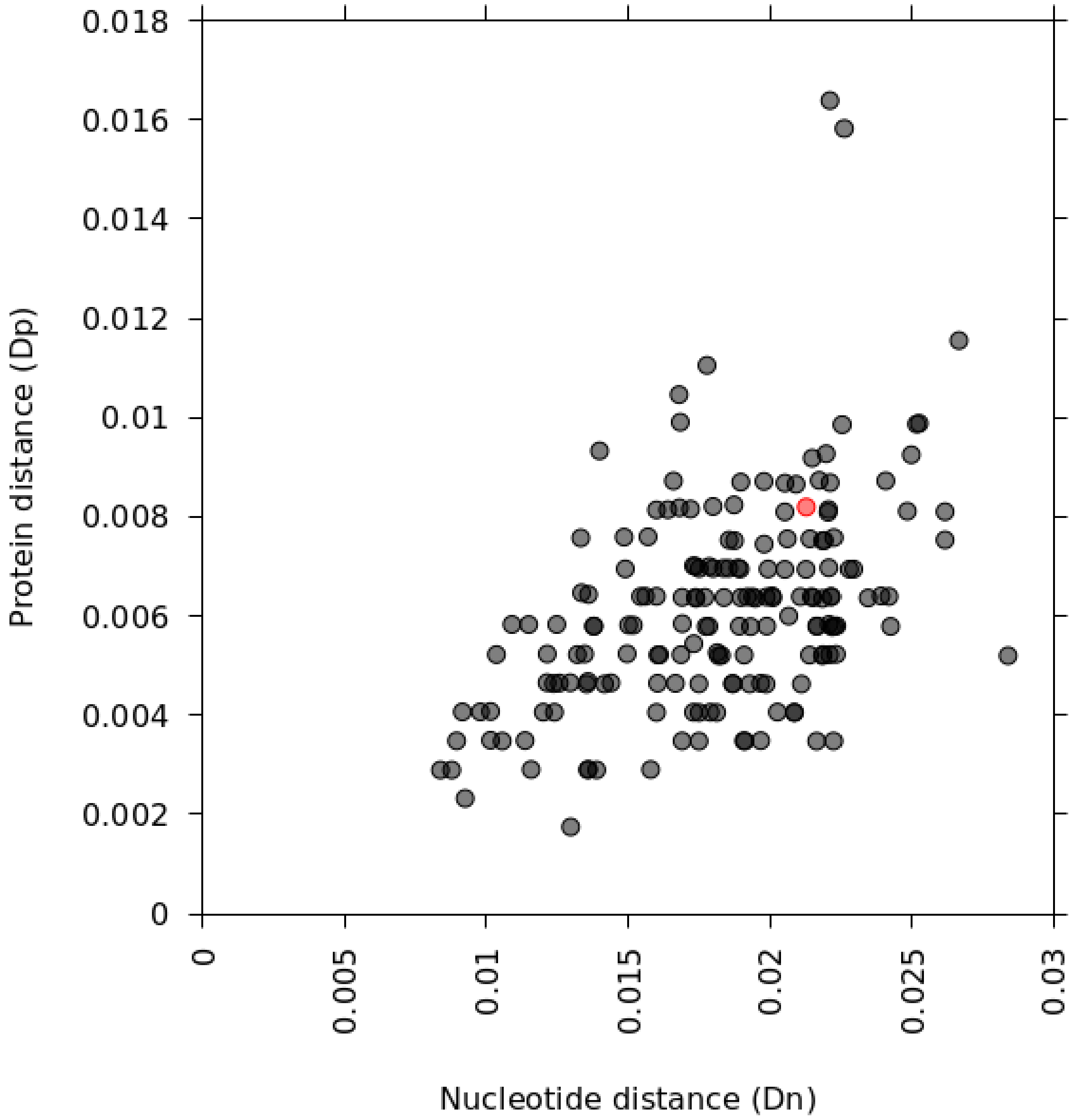

193. NC_043487.1/YP_009667147.1

CDS cluster ID	193
CDS cluster name	NC_043487.1/YP_009667147.1
Total sequences	10
Reference forms	9
Compensatory frameshift forms	1
Virus	Lelystad virus
Protein	RNA polymerase

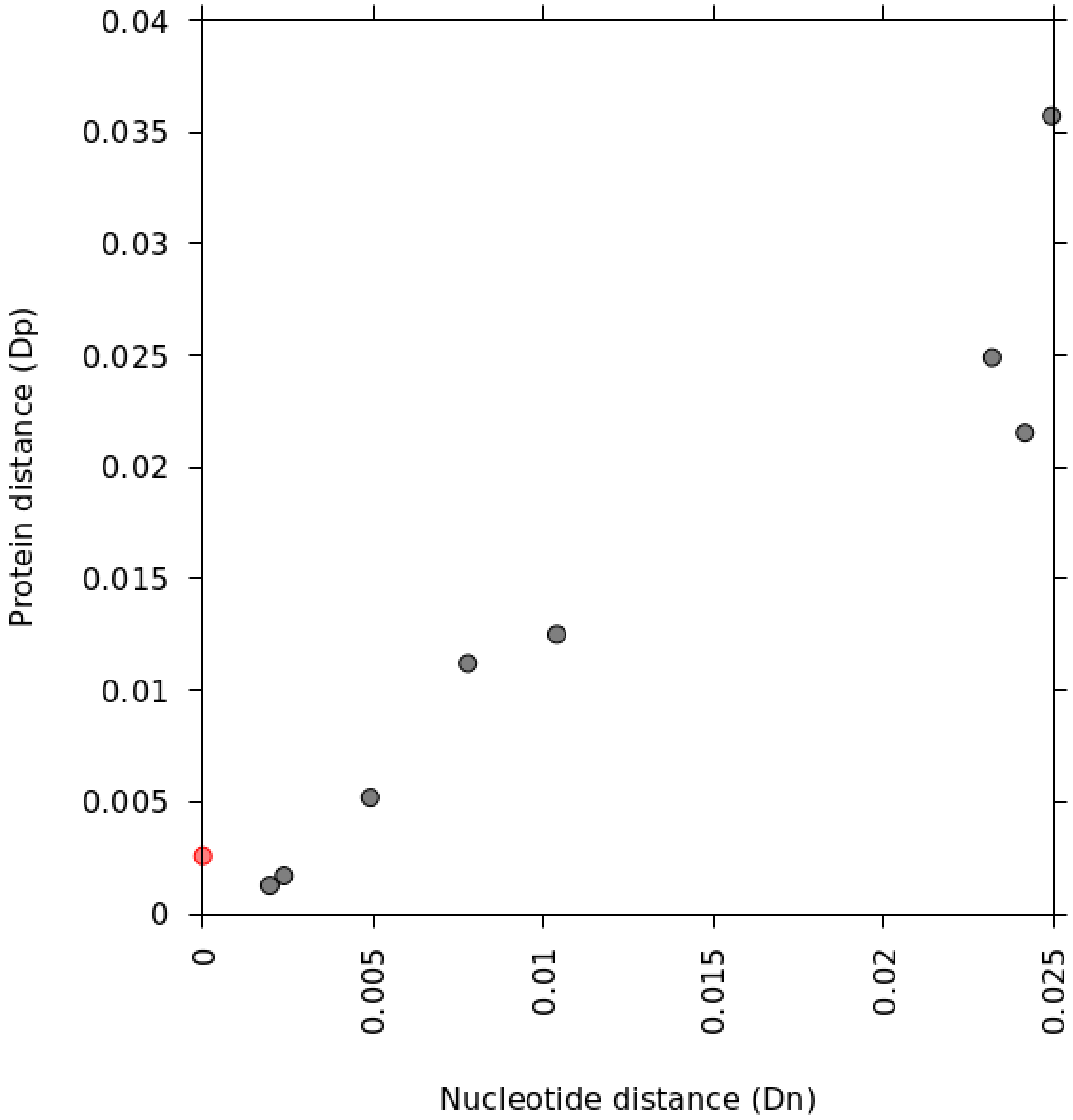

Supplement: Supplementary file 4 — Additional file 4: Supplementary Data S1. [file 12859_2021_4182_MOESM4_ESM.pdf]
